# The stable “F–SO_2_^+^” donor provides a mild and efficient approach to nitriles and amides†

**DOI:** 10.1039/d2ra05890a

**Published:** 2022-11-18

**Authors:** Yin Cui, Yiyong Zhao, Junjie Shen, Guofu Zhang, Chengrong Ding

**Affiliations:** College of Chemical Engineering, Zhejiang University of Technology Hangzhou 310014 P. R. China dingcr@zjut.edu.cn gfzhang@zjut.edu.cn; Zhejiang Ecological Environment Low Carbon Development Center Hangzhou 310014 P. R. China; Zhejiang Kefeng New Material Co. LTD Huzhou 313200 P. R. China

## Abstract

In this update, we developed a mild, efficient and practical method using fluorosulfuryl imidazolium salt A as an environment friendly promoter for conversion of oximes to nitriles or amides *via* β-elimination or Beckmann rearrangement in almost quantitative yield in 10 minutes. The target products were generated in gram-scale and could be collected through crystallization without silica gel column purification in excellent yield.

## Abstract 2

In this update, we developed a mild, efficient and practical method using stable “F–SO_2_^+^” donor A as an environment friendly promoter for conversion of oximes to nitriles or amides *via* β-elimination or Beckmann rearrangement in excellent yields.

## Graphical abstract

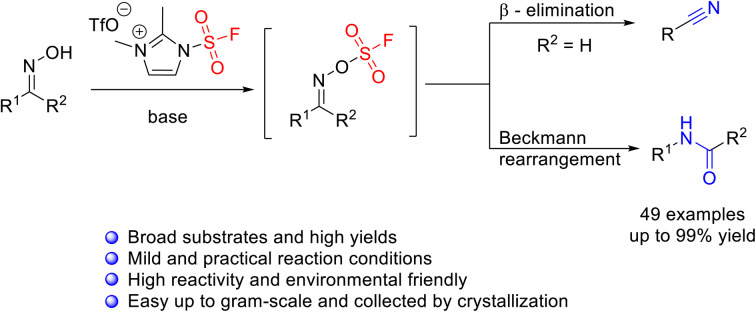

Nitriles and amides are important classes of organonitrogen compounds. Nitrile and amide play an important role in organic synthesis and are core structures of many agrochemicals, bioactive drugs, natural products, fine chemicals and functional materials.^1,2^ Examples include tecovirimat (1), an antiviral indicated for the treatment of smallpox,^3^ which is also effective in treating monkeypox infections;^4^ PF-07321332 (2), which is a nitrile inhibitor of the SARS-CoV-2 main protease;^5^ P5TCN-2F, a polythiophene organic solar cells (OSCs) (3), revealed that the cyano-group leads to high-efficiency OSCs and improved polymer crystallinity (Scheme 1).^6^

**Scheme 1:**
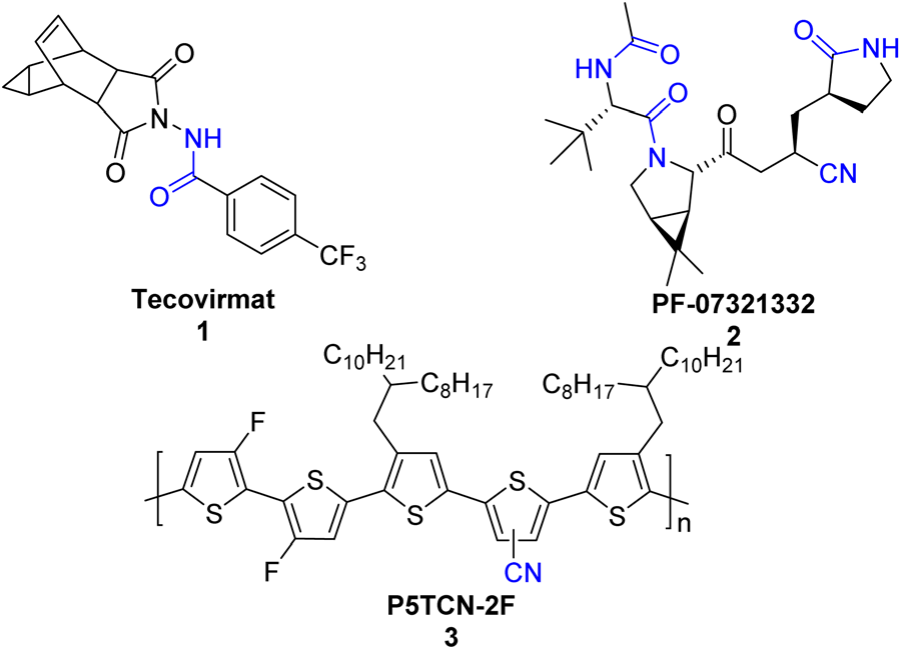
Selected examples for valuable nitriles and amides.

Owing to their widespread applications, there has been a push in recent decades to develop a more efficient, mild, Rosenmund–von Braun and safe approach to nitriles and amides. Sandmeyer and Braun reactions,^7^ formal acid–nitrile exchange,^8^ transition-metal-catalyzed cyanation of halides,^9^ and direct C–H cyanation are examples of traditional cyanide-based processes to nitriles (Scheme 2a(1)).^10^ Meanwhile, many cyanide-free synthetic methods have been developed, such as amide dehydration,^11^ primary amine hydrogenation,^12^ and cyanation with other nitrogen sources.^13^ Amides are commonly formed by reacting carboxylic acid or its derivatives with amines *via* condensation or transition metal-catalyzed coupling (Scheme 2a(2)).^14^ Aside from that, other protocols to amides have been reported, including carbonylative hydroamidation,^15^ nitrile hydrolysis,^16^ nitro-reduction amidation,^17^ and *N*-arylation of activated amides.^18^ The aforementioned strategies, however, were limited to toxic reagents, expensive transition-metal catalysts, complex reaction systems, and harsh conditions, especially when two or more components are used as raw materials, which may result in low atom utilization and more by-products. As a result, developing a solution to the aforementioned difficulties is critical.

**Scheme 2:**
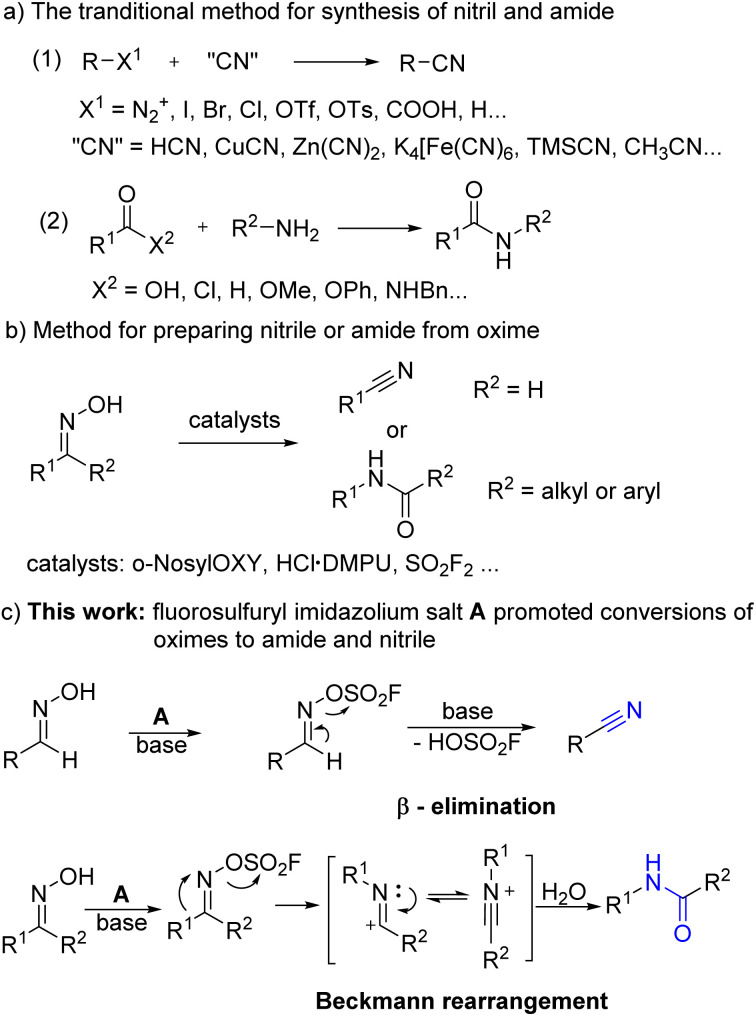
Strategies for preparation of nitriles and amides.

Oximes are simple and easily accessible class of chemical,^18^ particularly aldoximes and ketoximes, which could be converted to nitriles and amides efficiently by β-elimination and Beckman rearrangement,^19^ respectively (Scheme 2b). Various catalysts have been devised in recent years to facilitate the heterolysis of nitrogen–oxygen bond in order to achieve this transformation, but there are still certain drawbacks. Such as *o*-NosylOXY,^20^ required microwave irradiation and high temperature. In 2020, Xu reported that HCl·DMPU assisted conversion of aldehydes into nitriles while HCl–DMPU is a solution emitting fumes.^21^ Recently, Ding, Qin and Fokin groups reported rapid and mild SO_2_F_2_-promoted dehydration of oxime.^22^ However, the use of a greenhouse gas SO_2_F_2_ is not safe as it may leak out in operation.^23^ Although the organoselenium-catalyzed dehydration of aldoximes can produce nitriles under environment friendly conditions, it required for hours up to days.^24^ To some extends, those disadvantages restrict its wide applications.

In 2018, Dong and Sharpless reported a fluorosulfuryl imidazolium salt A, which showned unprecedented reactivity, selectivity, and scope as an “F–SO_2_^+^” donor and is a far more reactive fluorosulfurylating agent than SO_2_F_2_.^25^ Subsequently, it was developed for the crucial precursor of diazotransfer reagent, which enables the preparation of azides from primary amines.^26^ Moreover, fluorosulfuryl imidazolium salt A provides a practical and efficient process to prepare unsymmetrical sulfamides *via* Sulfur(vi)–Fluoride Exchange (SuFEx) click chemistry.^27^ Most recently, Liao and Wang groups reported that fluorosulfuryl imidazolium salt could produce SO_2_F radical and enabled fluorosulfonylation of olefins.^28^

Inspired by the wide application of fluorosulfuryl imidazolium salt and its unprecedented reactivity, and upon viewing the limitations of the preparation of nitriles and amides from oximes, we tried to apply fluorosulfuryl imidazolium salt A for the β-elimination of aldoximes and Beckmann rearrangement of ketoximes after our continuous efforts on the utilization of SO_2_F_2_-promoted transformations.^29^ As predicted, the alkylated imidazolium species served as good leaving groups and delivers the “F–SO_2_^+^” fragment,^27,30^ and aldoximes or ketoximes would react with “F–SO_2_^+^”, with the assistance of the base, to generate the corresponding sulfonyl ester, and further produce the nitriles or amides *via* β-elimination or Beckmann rearrangement. As predicted, aldoximes or ketoximes would react with fluorosulfuryl imidazolium salt A, with the assistance of the base, to generate the corresponding sulfonyl ester, and futher produce the nitriles or amides *via* β-elimination or Beckmann rearrangement (Scheme 2c). When 4-bromobenzaldehyde oxime (1d) or acetophenone oxime (3a) were used as model substrates, 4-bromobenzonitrile (2d) or *N*-phenylacetamide (4a) were obtained in 98% yields under the optimal reaction conditions (the more details please see ESI Tables S1 and S2†).

Having established the optimal reaction conditions, we examined the scope and generality of this protocol from aryl aldoximes into nitriles. As shown in Table 1, a wide range of aryl aldoximes with either electron-drawing or electron-donating functionality were tolerated, such as OMe (2c), Br (2d), Cl (2e), MeCOO (2h), Ph (2i), MeSO_2_ (2j) and NO_2_ (2k) groups at the 4-position, all gave the corresponding products in equivalent yields. Meanwhile, it should be noted that the isolated yields of temperature-sensitive arylnitrile (2a, 2b, 2f, 2g, 2x and 2y) are lower than GC yields due to their low boiling point and high volatility. With the exception of *para*-substituted substrates, substituents at the *meta* and *para* positions, whether carrying halogen, electron-drawing and donating groups, give the target product in satisfactory yields (2l–2t). Moreover, naphthyl and heterocyclic aldoximes including 2-naphthaldoxime (1u), 2-pyridinealdoxime (1v), thiophene-2-carboxaldoxime (1w) and 2-furaldehyde (1x) reacted well under the current reaction conditions in good to excellent yields. Lastly, we tried to useother aliphatic aldoximes (1aa–1ae) to further investigate the applicable scope of this reaction. To our delight, alkenes (2aa), alkynes (2ab) and long-chain alkanes aldoxime (2ae) react well in great yields.

**Table 1:** Scope of the synthesis of the nitriles^a^

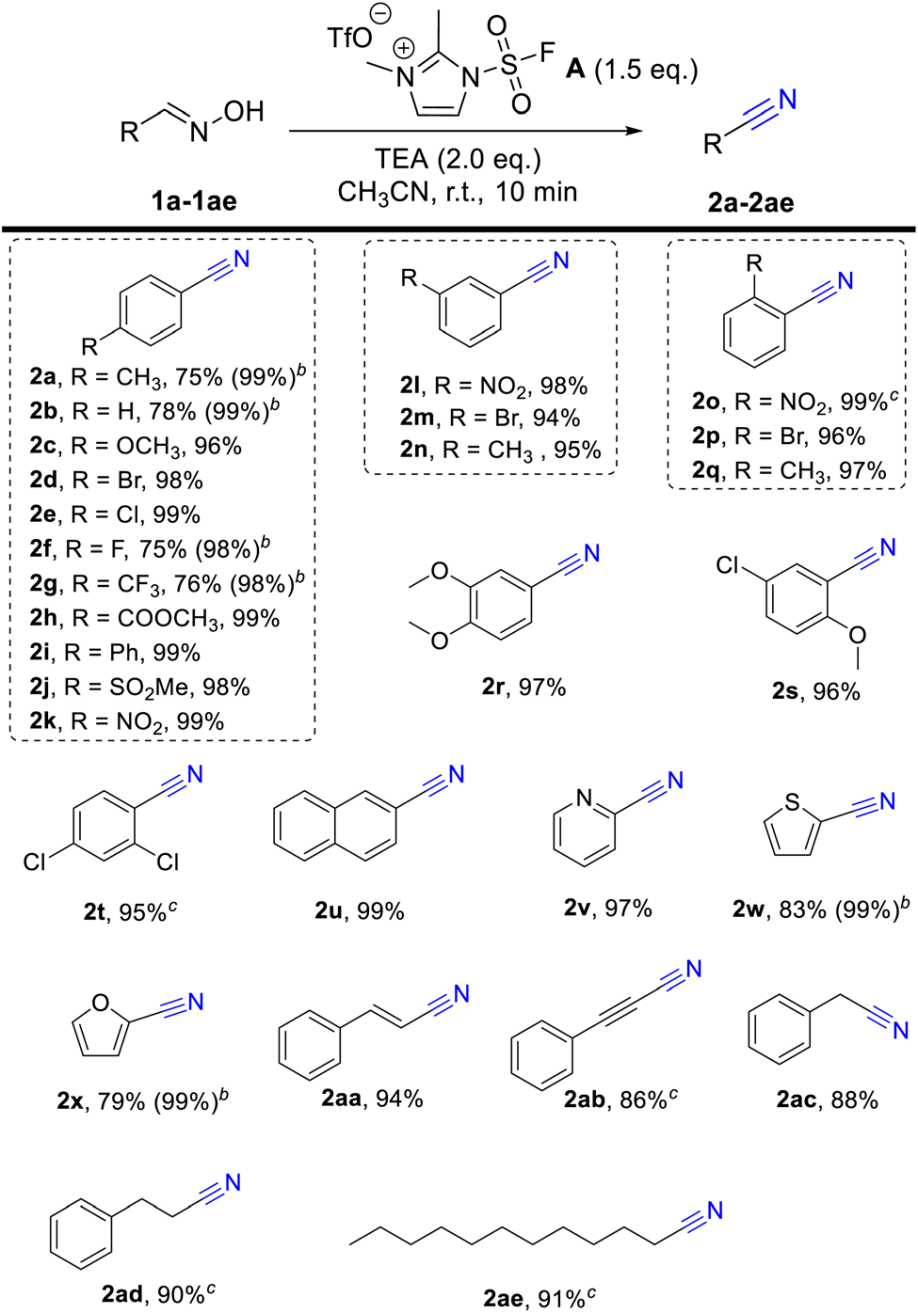

a Reaction conditions: aldoximes 1 (0.5 mmol), fluorosulfuryl imidazolium salt A (0.75 mmol, 1.5 eq.), TEA (1.0 mmol, 2.0 eq.), CH_3_CN (2.0 mL, 0.25 M), room temperature, 10 min, isolated yields.

b GC yields.

c 30 min.

With the encouragement of excellent conversion from aldoxime to nitriles, we further explored the applicability of Beckman rearrangement promoted by fluorosulfuryl imidazolium salt, and the corresponding results are shown in Table 2. Acetophenone oximes both with electron-donating groups (iPr, OMe, OPh) and with electron-withdrawing groups (F, Br, Cl, CN. NO_2_, COOMe) on the benzene ring (4b–4n), all converted into the corresponding products in high yields (81–97%). It indicated that electron and steric hindrance had little effect on this reaction. When 1-(1-naphthalenyl)ethenone oxime (3o) and 1-(2-naphthalenyl)ethenone oxime (3p) were used, the designed products were obtained in 93% and 95% yield, respectively. Moreover, the heteroaryl *N*-2-thienylacetamide (4q) was generated in 81% yield. Expanding the scope, we explored the reaction with cyclic or aliphatic ketoximes. Much to our delight, the rearranged product *N*-(2-phenylethyl)acetamide (4r), 1,3,4,5-tetrahydro-2*H*-1-benzazepin-2-one (4s) and ε-caprolactam (4t) were produced in satisfactory yields (72–88%).

**Table 2:** Scope of the synthesis of the amides^a^

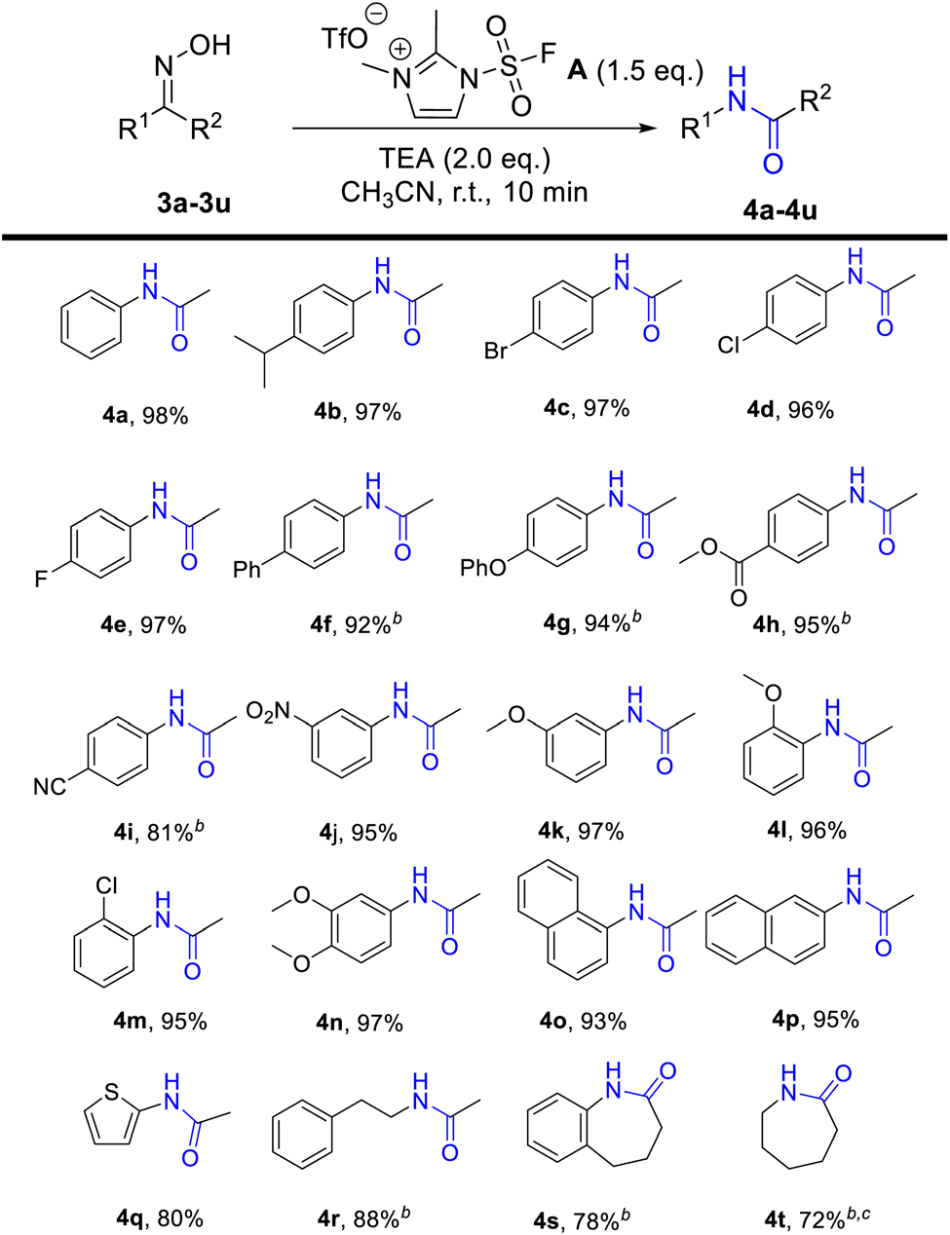

a Reaction conditions: ketoximes 3 (0.5 mmol), fluorosulfuryl imidazolium salt A (0.75 mmol, 1.5 eq.), TEA (1.0 mmol, 2.0 eq.), CH_3_CN (2.0 mL, 0.25 M), room temperature, 10 min, isolated yields.

b 30 min.

c 1.5 equiv. of DBU was used.

Encouraged by the high yields of the aforementioned substrates, we tried further gram-scale reactions and aldehyde or ketone were used as starting material, to confirm that this method was more pragmatic than previous reports. As we can see in the Scheme 3, 4-phenylbenzaldehyde (B) or acetophenone (C) was treated with hydroxylamine, producing 4-phenylbenzaldoxime (1i) and acetophenone oxime (3a), that were concentrated to remove ethanol and reacted with fluorosulfuryl imidazolium salt in the acetonitrile. It was worthy noted that 4-phenylbenzonitrile (2i) and acetanilide (4a) could be obtained in excellent yields through extraction and crystallization without further column purifications. There is no doubt that this is a more efficient and simple strategy for the synthesis of nitriles and amides.

**Scheme 3:**
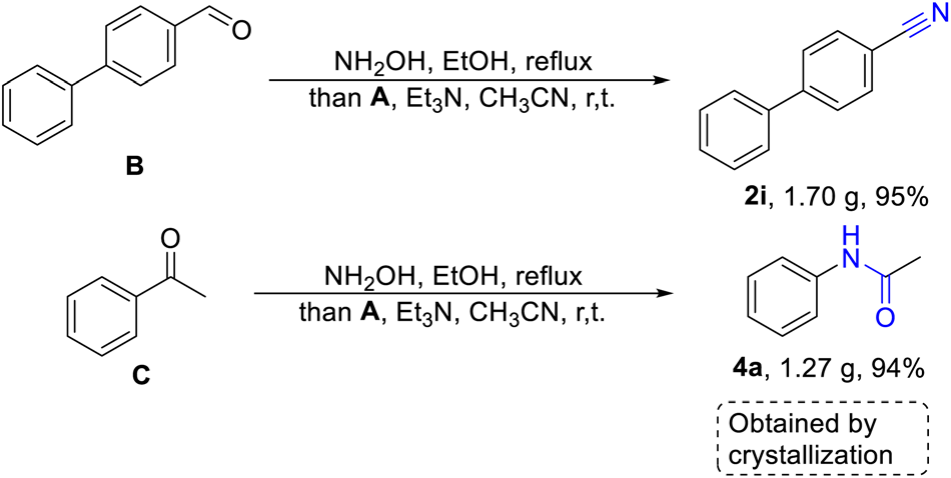
Gram-scale production of 2i and 4a by crystallization *via* cascade process.

To further demonstrate the applicability of this stable SO_2_F donor in the synthesis of complicated molecules, aldehyde D was used as the starting material under standard reaction condition and provided the key precursor for Tarceva in 95% yield (Scheme 4a). We also examined the synthesis of 2-cyano-4′-methylbiphenyl G and obtained the desired precursor for the novel sartan antihypertensive drugs (*e.g.*, Losartan, Valsartan, Eprosartan and Irbesartan) in 97% yield (Scheme 4b).

**Scheme 4:**
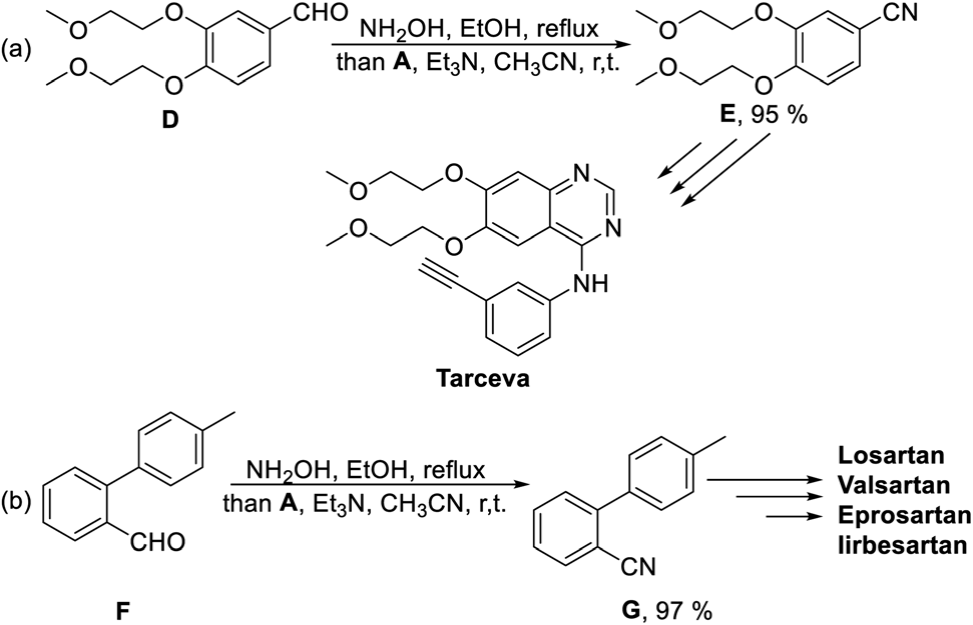
Synthesis of precursors for Tarceva and sartan antihypertensive drugs.

In conclusion, we have successfully applied fluorosulfuryl imidazolium salt to facilitate the heterolysis of nitrogen–oxygen bond, which can provide an expeditious approach to the synthesis of nitriles and amides in almostly quantitative yield at room temperature. The reaction proceeded well with a broad range of aromatic and aliphatic oximes. Furthermore, the gram-scale reaction was carried out without a hitch, and the target product were obtained in excellent yield through crystallization. Moreover, the cascade process was found to be applicable to the synthesis of key precursors for drug molecules in satisfactory yields. Despite the fact that fluorosulfuryl imidazolium salt A is a stable and effective reagent for encouraging nitrogen–oxygen bond breakage, the production of fluorosulfuryl imidazolium salt A necessitates the use of sulfuryl fluoride, a greenhouse gas. Therefore, it is critical to find more environmentally friendly ways to develop a novel “F–SO_2_” donor that also have superior reactivity.

## Conflicts of interest

There are no conflicts to declare.

## Supplementary Material

RA-012-D2RA05890A-s001
