# Supplementary material for: The stable “F–SO2+” donor provides a mild and efficient approach to nitriles and amides
Source: RSC Adv. 2022 Nov 18;12(51):33064–8. doi: 10.1039/d2ra05890a (PMC9672908; doi:10.1039/d2ra05890a)

Electronic Supplementary Material (ESI) for RSC Advances.
This journal is © The Royal Society of Chemistry 2022

Supporting Information

The stable "F−SO_2" donor provides a mild and efficient approach to nitrile and amide

Yin Cui,[a] Yiyong Zhao,[b] Junjie Shen,[c] Guofu Zhang[a]* and Chengrong Ding[a]*

[a]College of Chemical Engineering, Zhejiang University of Technology,

Hangzhou 310014, P. R. China.

E-mail: dingcr@zjut.edu.cn; gfzhang @zjut.edu.cn;

[b]Zhejiang Ecological Environment Low Carbon Development Center, Hangzhou

310014, P. R. China.

[c]Zhejiang Kefeng New Material Co. LTD, Huzhou 313200, P. R. China.

Table of Contents

1. General information S2

2. General procedure for the synthesis of Substrates and fluorosulfuryl imidazolium salt **A**..S3-S4

3. Optimization of reaction conditions S5-S6

4. General procedure for the synthesis of Products **2** S7-S14

5. General procedure for the synthesis of Products **4** S15-S20

6. Gram-scale synthesis of **2i** and **4a** S21-S22

7. Synthesis of precursors for drugs..S23-S24

8. References S24

9. NMR Spectra...S25-S128

Experimental Section

1. General information

All starting materials and reagents were purchased from commercial sources and used as received unless otherwise noted. Reactions were monitored using thin-layer chromatography (TLC) on commercial silica gel plates. Visualization of the developed plates was performed under UV light (254nm). Flash column chromatography was performed on silica gel. GC analysis of determination of conversion was performed on the instrument of Agilent 7890 GC. NMR spectra were recorded on Bruker AVANCE III 400MHz (^{1}H NMR) and 101MHz (^{13}C NMR) instrument in Chloroform-*d* or DMSO-d_6 with TMS as internal standard.

2. General procedure for the synthesis of Substrates and fluorosulfuryl imidazolium salt A

General procedure for the synthesis of Substrates 1

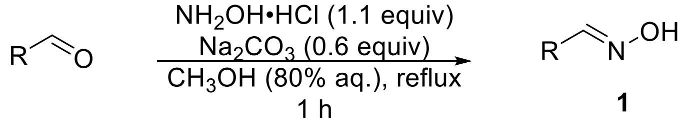

Aldehydes (20 mmol, 1.0 equiv), hydroxylamine hydrochloride (1.53 g, 22 mmol) and Na_2CO_3 (1.27 g, 12 mmol) were dissolved in 50 mL CH_3OH (80 % aq.). The reaction mixture was heated under reflux for 0.5-1.0 h. After the reaction was complete by TLC, the reaction was cooled to room temperature, diluted with water (50 mL) and extracted with ethyl acetate (3×50 mL). And then the combined organic layers were washed with brine, dried over anhydrous Na_2SO_4 and concentrated to dryness. This crude aldoximes **1** was used without further purification.

General procedure for the synthesis of Substrates 3

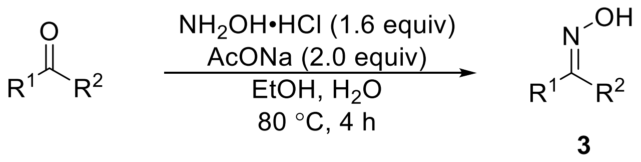

Ketones (20 mmol, 1.0 equiv), hydroxylamine hydrochloride (32 mmol, 1.6 equiv) and AcONa (40 mmol, 2.0 equiv) were dissolved in the mixture of EtOH/H_2O (v/v, 4:1, 50 mL). The reaction mixture was stirred at 80 ℃ until the consumption of the starting material was observed by TLC. After that, the reaction was cooled to room temperature, diluted with water (100 mL), extracted with ethyl acetate (150 mL ×3), dried with anhydrous Na_2SO_4, and concentrated. The residue was purified by recrystallization or flash column chromatography on silica gel to afford the desired substrates **3**.

General procedure for the synthesis of fluorosulfuryl imidazolium salt A

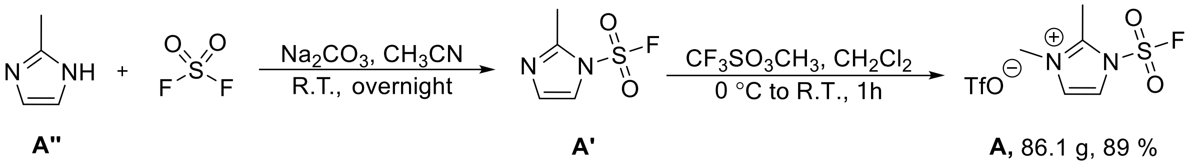

2-Methyl-1H-imidazole (24.7 g, 300 mmol), acetonitrile (600 mL) and sodium carbonate (79.6

g, 750 mmol) were added into a 1L round-bottom flask. Round-bottom flask equipped with magnetic stirrer and rubber stopper. Then the SO_2F_2 gas was introduced into the stirring reaction mixture by slow bubbling through a SO_2F_2 balloon, and the reaction mixture was stirred at room temperature for overnight. The progress of the reaction was monitored by TLC. After completion of the reaction, the reaction mixture was diluted with 600 mL of DCM and washed with water (3000 mL × 3), saturated NaCl solution (600 mL) and dried over anhydrous Na_2SO_4 and the evaporation of the solvent gave crude intermediate **A'** with some residual acetonitrile and dichloromethane. This crude mixture was used directly in the next step. Crude intermediate **A'** and dichloromethane (300mL) were added into a 500mL round-bottom flask. Round-bottom flask equipped with magnetic stirrer. Then methyl trifluoromethanesulfonate (34 mL, 296 mmol) added slowly at 0 °C under N_2. The mixture allowed to warm to room temperature and stirred for 4 hours, while monitoring by TLC. After that time, the mixture was concentrated under rotary evaporation to give a white viscous crude product, to which methyl tertbutyl ether (300 mL) was added. With vigorous stirring, a solid precipitate was formed. The precipitate was washed with methyl tert-butyl ether (300mL × 3) and dried in vacuo to yield the compound **A** as a white solid (86.1 g, overall yield 89 %).

Analytic Data of fluorosulfuryl imidazolium salt A

1-(Fluorosulfuryl)-2,3-dimethyl-1H-imidazol-3-ium trifluoromethanesulfonate (**A**)[1]

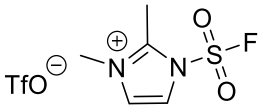

^{1}H NMR (400 MHz, DMSO-d_6) δ 7.44 (d, J = 2.0 Hz, 1H), 7.41 (d, J = 1.9 Hz, 1H), 3.68 (s, 3H), 2.50 (s, 3H). ^{19}F NMR (377 MHz, DMSO-d_6) δ 39.97 (s, 1F), -77.96 (s, 3F).

3. Optimization of reaction conditions

Table 1 Optimization of converting aldoxime to nitrile[a]

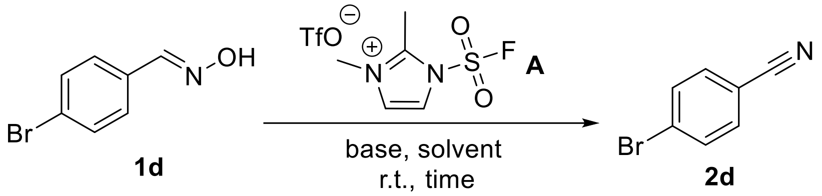

Entry	**A** (eq.)	Base (eq.)	Solvent (mL)	Yield (%)[b]
1	1.0	Et_3N (2.0)	CH_3CN	70
**2**	**1.5**	**Et_3N (2.0)**	**CH_3CN**	**98**
3	2.0	Et_3N (2.0)	CH_3CN	97
4	1.5	DIPEA (2.0)	CH_3CN	87
5	1.5	DBU(2.0)	CH_3CN	81
6	1.5	Pyridine (2.0)	CH_3CN	24
7	1.5	Na_2CO_3 (2.0)	CH_3CN	42
8	1.5	K_2CO_3 (2.0)	CH_3CN	64
9	1.5	CsF (2.0)	CH_3CN	48
10	1.5	NaOH (2.0)	CH_3CN	50
11	1.5	Et_3N (1.0)	CH_3CN	21
12	1.5	Et_3N (1.5)	CH_3CN	53
13[c]	1.5	Et_3N (2.0)	CH_3CN	98
14	1.5	Et_3N (2.0)	CH_2Cl_2	62
15	1.5	Et_3N (2.0)	H_2O	49
16	1.5	Et_3N (2.0)	EtOAc	38
17	1.5	Et_3N (2.0)	DMF	52
18	1.5	Et_3N (2.0)	CH_3OH	37
19	1.5	Et_3N (2.0)	THF	35
20	1.5	Et_3N (2.0)	Heptane	47
21	No **A**	Et_3N (2.0)	CH_3CN	No reaction
22	1.5	No base	CH_3CN	No reaction

[a] Reaction conditions: 4-bromobenzaldehyde oxime **1d** (0.5 mmol), fluorosulfuryl imidazolium salt **A**, base, CH_3CN (2.0 mL, 0.25 M), room temperature, 10 min. [b] Isolated yield. [c] 15 min.

Table 2 Optimization of converting ketoxime to amide[a]

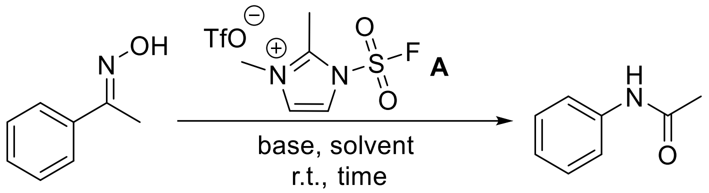

Entry	**A** (eq.)	Base	Solvent	Yield[b] (%)
1	1.0	Et_3N (2.0)	CH_3CN	64
**2**	**1.5**	**Et_3N (2.0)**	**CH_3CN**	**98**
3	2.0	Et_3N (2.0)	CH_3CN	98
4	1.5	DIPEA (2.0)	CH_3CN	90
5	1.5	DBU(2.0)	CH_3CN	70
6	1.5	Pyridine (2.0)	CH_3CN	72
7	1.5	Na_2CO_3 (2.0)	CH_3CN	78
8	1.5	K_2CO_3 (2.0)	CH_3CN	88
9	1.5	$CsCO_3$ (2.0)	CH_3CN	83
10	1.5	Et_3N (1.0)	CH_3CN	80
11	1.5	Et_3N (1.5)	CH_3CN	91
12[c]	1.5	Et_3N (2.0)	CH_3CN	98
13	1.5	Et_3N (2.0)	DMF	80
14	1.5	Et_3N (2.0)	CH_2Cl_2	81
15	1.5	Et_3N (2.0)	THF	83
16	1.5	Et_3N (2.0)	H_2O	35
17	1.5	Et_3N (2.0)	EtOAc	62
18	1.5	Et_3N (2.0)	CH_3OH	31
19	1.5	Et_3N (2.0)	n-hexane	46
20	No **A**	Et_3N (2.0)	CH_3CN	No reaction
21	1.5	No base	CH_3CN	44

[a] Reaction conditions: acetophenone oxime **3a** (0.5 mmol), fluorosulfuryl imidazolium salt **A**, base, CH_3CN (2.0 mL, 0.25 M), room temperature, 10 min. [b] Isolated yield. [c] 15 min.

4. General procedure for the synthesis of Products 2

General procedure for the synthesis of Products 2

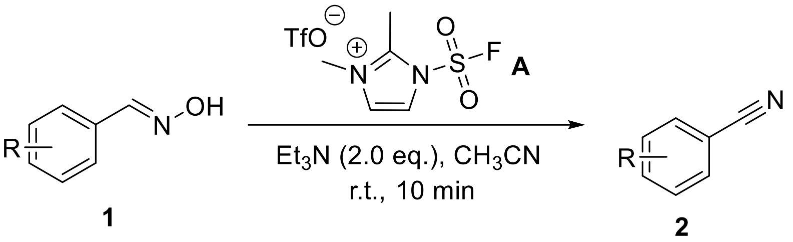

Aldoxime **1** (0.5 mmol), CH_3CN (1.0 mL) and Et_3N (139 μL, 1.0 mmol) were added into a 15 mL Schlenk flask equipped with magnetic stirrer and rubber stopper. Then the fluorosulfuryl imidazolium salt **A** (246 mg, 0.75 mmol) was dissolved in acetonitrile (1.0 mL) and that was injected slowly with a syringe into Schlenk flask, and the reaction mixture was stirred at room temperature for 10 minutes. The progress of the reaction was monitored by TLC or GC. After completion of the reaction, the reaction mixture was diluted with 10 mL of DCM and washed with 5% HCl (2×8 mL), 5% $NaHCO_3$ (2×8 mL), saturated NaCl solution (2×8 mL) and dried over anhydrous Na_2SO_4 and the evaporation of the solvent gave a residue that was purified on silica gel column chromatography using petroleum ether and ethyl acetate.

Analytic Data of Products 2

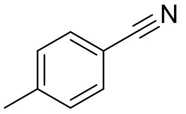

4-Methylbenzonitrile (2a)[2]

White solid (43.9 mg, 75%). ^{1}H NMR (400 MHz, Chloroform-*d*) δ 7.55 (d, *J* = 8.2 Hz, 2H), 7.28 (d, *J* = 8.0 Hz, 2H), 2.44 (s, 3H). ^{13}C NMR (101 MHz, Chloroform-*d*) δ 143.72, 132.05 , 129.85 , 119.17 , 109.32 , 21.84 .

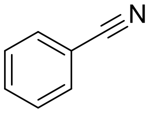

Benzonitrile (2b)[2]

Colorless oil (40.2 mg, 78%).^{1}H NMR (400 MHz, Chloroform-*d*) δ 7.65 – 7.52 (m, 3H), 7.43 (t, *J* = 7.8 Hz, 2H). ^{13}C NMR (101 MHz, Chloroform-*d*) δ 132.83 , 132.09 , 129.16 , 118.84 , 112.33.

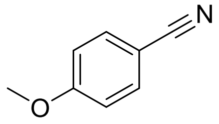

4-Methoxybenzonitrile (2c)[2]

White solid (63.9 mg, 96%). ^{1}H NMR (400 MHz, Chloroform-*d*) δ 7.57 – 7.38 (m, 2H), 6.88 – 6.77 (m, 2H), 3.76 (s, 3H). ^{13}C NMR (101 MHz, Chloroform-*d*) δ 162.83, 133.85, 119.20, 114.76, 103.65, 55.51.

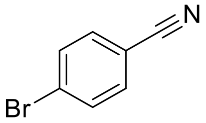

4-Bromobenzonitrile (2d)[3]

White solid (89.2 mg, 98%). ^{1}H NMR (400 MHz, Chloroform-*d*) δ 7.62 – 7.54 (m, 2H), 7.52 – 7.45 (m, 2H). ^{13}C NMR (101 MHz, Chloroform-*d*) δ 133.46 , 132.62 , 127.97 , 118.07 , 111.19 .

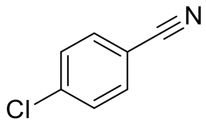

4-Chlorobenzonitrile (2e)[2]

White solid (68.0 mg, 99%). ^{1}H NMR (400 MHz, Chloroform-*d*) δ 7.58 (d, *J* = 8.6 Hz, 2H), 7.43 (d, *J* = 8.7 Hz, 2H). ^{13}C NMR (101 MHz, Chloroform-*d*) δ 139.47 , 133.41 , 129.68 , 117.96 , 110.77 .

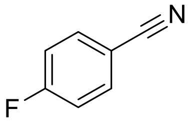

1,3-bis(4-bromophenyl)urea (2f)[3]

White solid (45.4 mg, 75%).^{1}H NMR (400 MHz, Chloroform-*d*) δ 7.60 (ddd, *J* = 9.5, 5.0, 2.4 Hz, 2H), 7.18 – 7.01 (m, 2H). ^{13}C NMR (101 MHz, Chloroform-*d*) δ 164.92 (d, *J* = 255.9 Hz), 134.69 (d, *J* = 9.4 Hz), 117.98 , 116.76 (d, *J* = 22.6 Hz), 108.47 (d, *J* = 3.6 Hz).

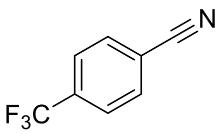

4-Fluorobenzonitrile (2g)[4]

Colorless oil (63.4 mg, 84%). ^{1}H NMR (400 MHz, Chloroform-*d*) δ 7.89 – 7.71 (m, 4H). ^{13}C NMR (101 MHz, Chloroform-*d*) δ 134.70 , 134.37 , 132.71 , 126.21 , 126.18 , 126.14 , 124.42 , 121.71 , 117.46 , 116.05 .

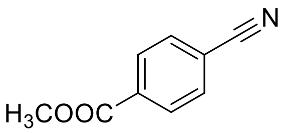

Methyl 4-cyanobenzoate (2h)[2]

Colorless oil (79.7 mg, 99%).^{1}H NMR (400 MHz, Chloroform-*d*) δ 8.07 – 7.96 (m, 2H), 7.71 – 7.57 (m, 2H), 3.86 (s, 3H). ^{13}C NMR (101 MHz, Chloroform-*d*) δ 165.27 , 133.81 , 132.18 , 129.99 , 117.88 , 116.23 , 52.63 .

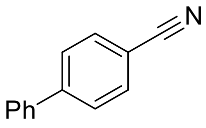

[1,1'-biphenyl]-4-Carbonitrile (2i)[3]

White solid (88.7 mg, 99%). ^{1}H NMR (400 MHz, Chloroform-*d*) δ 7.61 (q, *J* = 8.5, 7.7 Hz, 4H), 7.56 (d, *J* = 7.2 Hz, 2H), 7.51 – 7.37 (m, 3H). ^{13}C NMR (101 MHz, Chloroform-*d*) δ 145.41 , 138.98 , 132.61 , 129.23 , 128.81 , 127.66 , 127.24 , 119.06 , 110.85 .

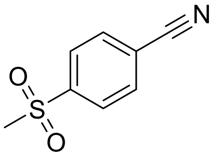

4-(methylsulfonyl)Benzonitrile (2j)[3]

White solid (88.8 mg, 98%). ^{1}H NMR (400 MHz, Chloroform-*d*) δ 8.12 – 8.01 (m, 2H), 7.98 – 7.83 (m, 2H), 3.10 (s, 3H). ^{13}C NMR (101 MHz, Chloroform-*d*) δ 144.44 , 133.27 , 128.22 , 117.51 , 117.16 , 44.19 .

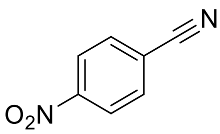

4-Nitrobenzonitrile (2k)[2]

Yellow solid (73.3 mg, 99%). ^{1}H NMR (400 MHz, Chloroform-*d*) δ 8.36 (dt, *J* = 9.0, 2.0 Hz, 2H), 7.91 (dt, *J* = 9.0, 2.0 Hz, 2H). ^{13}C NMR (101 MHz, Chloroform-*d*) δ 150.03 , 133.58 , 124.31 , 118.28 , 116.90 .

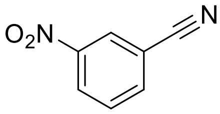

3-Nitrobenzonitrile (2l)[3]

White solid (72.5 mg, 98%). ^{1}H NMR (400 MHz, Chloroform-*d*) δ 8.59 – 8.53 (m, 1H), 8.50 (ddd, *J* = 8.4, 2.1, 1.0 Hz, 1H), 8.03 (d, *J* = 7.7 Hz, 1H), 7.77 (t, *J* = 8.0 Hz, 1H). ^{13}C NMR (101 MHz, Chloroform-*d*) δ 148.23 , 137.68 , 130.74 , 127.57 , 127.24 , 116.58 , 114.11 .

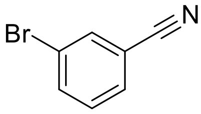

3-Bromobenzonitrile (2m)[3]

White solid (85.5 mg, 94%). ^{1}H N MR (400 MHz, Chloroform-*d*) δ 7.80 – 7.69 (m, 2H), 7.60 (d, *J* = 7.8 Hz, 1H), 7.36 (t, *J* = 7.9 Hz, 1H). ^{13}C NMR (101 MHz, Chloroform-*d*) δ 136.14 , 134.72 , 130.75 , 130.70 , 122.89 , 117.30 , 114.18 .

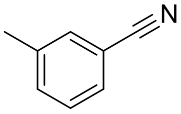

3-Methylbenzonitrile (2n)[5]

White solid (55.6 mg, 95%). ^{1}H NMR (400 MHz, Chloroform-*d*) δ 7.42 – 7.26 (m, 4H), 2.33 (s, 3H). ^{13}C NMR (101 MHz, Chloroform-*d*) δ 139.24 , 133.67 , 132.36 , 129.17 , 129.02 , 118.96 , 112.13 , 21.04 .

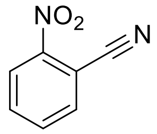

2-Nitrobenzonitrile (2o)[6]

White solid (73.3 mg, 99%).^{1}H NMR (400 MHz, Chloroform-*d*) δ 7.52 (d, *J* = 7.9 Hz, 1H), 7.47 – 7.39 (m, 1H), 7.30 – 7.19 (m, 2H), 2.49 (s, 3H). ^{13}C NMR (101 MHz, Chloroform-*d*) δ 141.80 , 132.67 , 132.40 , 130.23 , 126.24 , 118.07 , 112.66 , 20.35 .

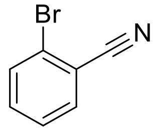

2-Bromobenzonitrile (2p)[3]

White solid (87.3 mg, 96%). ^{1}H NMR (400 MHz, Chloroform-*d*) δ 7.75 – 7.65 (m, 2H), 7.53 – 7.41 (m, 2H). ^{13}C NMR (101 MHz, Chloroform-*d*) δ 134.35 , 133.95 , 133.23 , 127.69 , 125.32 , 117.16 , 115.86 .

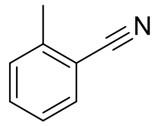

2-Methylbenzonitrile (2q)[5]

Colorless oil (56.8 mg, 97%). ^{1}H NMR (400 MHz, Chloroform-*d*) δ 7.52 (d, *J* = 7.9 Hz, 1H), 7.47 – 7.39 (m, 1H), 7.30 – 7.19 (m, 2H), 2.49 (s, 3H). ^{13}C NMR (101 MHz, Chloroform-*d*) δ 141.80 , 132.67 , 132.40 , 130.23 , 126.24 , 118.07 , 112.66 , 20.35 .

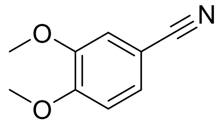

3,4-Dimethoxybenzonitrile (2r)[5]

White solid (79.1 mg, 97%). ^{1}H NMR (400 MHz, Chloroform-*d*) δ 7.25 (d, *J* = 8.3 Hz, 1H), 7.04 (s, 1H), 6.88 (d, *J* = 8.4 Hz, 1H), 3.90 (s, 3H), 3.86 (s, 3H). ^{13}C NMR (101 MHz, Chloroform-*d*) δ 152.86 , 149.15 , 126.46 , 119.24 , 113.88 , 111.27 , 103.76 , 56.12 .

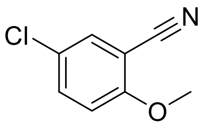

5-Chloro-2-methoxybenzonitrile (2s)[7]

White solid (84.4 mg, 96%). ^{1}H NMR (400 MHz, Chloroform-*d*) δ 7.50 (dd, *J* = 7.4, 2.5 Hz, 2H), 7.01 – 6.86 (m, 1H), 3.94 (s, 3H). ^{13}C NMR (101 MHz, Chloroform-*d*) δ 159.93 , 134.40 , 132.97 , 125.64 , 115.14 , 112.73 , 103.10 , 56.44 .

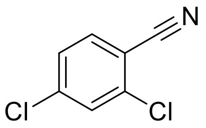

2,4-Dichlorobenzonitrile (2t)[6]

White solid (81.7 mg, 95%). ^{1}H NMR (400 MHz, Chloroform-*d*) δ 7.63 (d, *J* = 8.4 Hz, 1H), 7.56 (d, *J* = 1.9 Hz, 1H), 7.39 (dd, *J* = 8.4, 2.0 Hz, 1H). ^{13}C NMR (101 MHz, Chloroform-*d*) δ 140.11 , 137.82 , 134.62 , 130.28 , 127.89 , 115.24 , 111.90 .

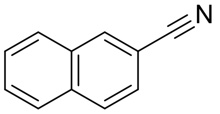

2-Naphthonitrile (2u)[2]

White solid (75.8 mg, 99%). ^{1}H NMR (400 MHz, Chloroform-*d*) δ 8.15 (s, 1H), 7.85 (t, *J* = 9.1 Hz, 3H), 7.67 – 7.51 (m, 3H). ^{13}C NMR (101 MHz, Chloroform-*d*) δ 134.60 , 134.11 , 132.18 , 129.20 , 129.09 , 128.40 , 128.06 , 127.69 , 126.27 , 119.31 , 109.27 .

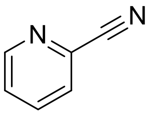

Picolinonitrile (2v)[3]

Yellow oil (50.5 mg, 97%). ^{1}H NMR (400 MHz, Chloroform-*d*) δ 8.64 (d, *J* = 4.8 Hz, 1H), 7.83 (td, *J* = 7.8, 1.7 Hz, 1H), 7.69 – 7.63 (m, 1H), 7.51 (ddd, *J* = 7.8, 4.8, 1.1 Hz, 1H). ^{13}C NMR (101 MHz, Chloroform-*d*) δ 151.10 , 137.26 , 133.70 , 128.62 , 127.16 , 117.27 .

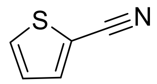

Thiophene-2-carbonitrile (2w)[3]

Colorless oil (45.3 mg, 83%). ^{1}H NMR (400 MHz, Chloroform-*d*) δ 7.74 – 7.51 (m, 2H), 7.13 (dd, *J* = 4.9, 3.9 Hz, 1H). ^{13}C NMR (101 MHz, Chloroform-*d*) δ 137.51 , 132.76 , 127.75 , 114.30 , 109.78 .

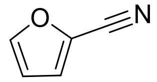

Furan-2-carbonitrile (2x)[6]

Colorless oil (36.8 mg, 79%). ^{1}H NMR (400 MHz, Chloroform-*d*) δ 7.56 (d, *J* = 1.8 Hz, 1H), 7.08 (d, *J* = 3.7 Hz, 1H), 6.51 (dd, *J* = 3.7, 1.9 Hz, 1H). ^{13}C NMR (101 MHz, Chloroform-*d*) δ 147.58 , 126.04 , 122.18 , 111.57 , 111.47 .

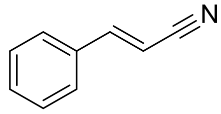

Cinnamonitrile (2aa)[2]

Colorless oil (60.7 mg, 94%) ^{1}H NMR (400 MHz, Chloroform-*d*) δ 7.42 (q, *J* = 5.3, 4.8 Hz, 5H), 7.34 (d, *J* = 16.6 Hz, 1H), 5.85 (d, *J* = 16.7 Hz, 1H). ^{13}C NMR (101 MHz, Chloroform-*d*) δ 150.54 , 133.55 , 131.27 , 129.16 , 127.47 , 118.33 , 96.36 .

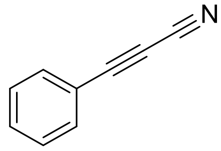

3-Phenylpropiolonitrile (2ab)[3]

White solid (54.6 mg, 86%) ^{1}H NMR (400 MHz, Chloroform-*d*) δ 7.64 – 7.57 (m, 2H), 7.53 (t, *J* = 7.6 Hz, 1H), 7.41 (t, *J* = 7.7 Hz, 2H). ^{13}C NMR (101 MHz, Chloroform-*d*) δ 133.49 , 131.94 , 128.90 , 117.45 , 105.48 , 83.06 , 63.03 .

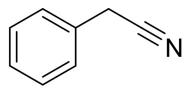

2-Phenylacetonitrile (2ac)[2]

Colorless oil (51.5 mg, 88%) ^{1}H NMR (400 MHz, Chloroform-*d*) δ 7.53 – 7.30 (m, 5H), 3.73 (s, 2H). ^{13}C NMR (101 MHz, Chloroform-*d*) δ 130.12 , 129.17 , 128.06 , 128.00 , 118.10 , 23.55 .

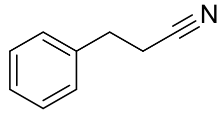

3-Phenylpropanenitrile (2ad)[6]

Colorless oil (59.0 mg, 90%) ^{1}H NMR (400 MHz, Chloroform-*d*) δ 7.43 – 7.24 (m, 5H), 2.95 (t, *J* = 7.4 Hz, 2H), 2.60 (t, *J* = 7.4 Hz, 2H). ^{13}C NMR (101 MHz, Chloroform-*d*) δ 138.28 , 128.91 , 128.40 , 127.25 , 119.38 , 31.51 , 19.28.

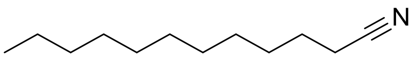

Dodecanenitrile (2ae)[8]

White solid (82.5 mg, 91%) ^{1}H NMR (400 MHz, Chloroform-*d*) δ 2.30 (t, *J* = 7.1 Hz, 2H), 1.62 (p, *J* = 7.2 Hz, 2H), 1.49 – 1.35 (m, 2H), 1.35 – 1.11 (m, 14H), 0.85 (t, *J* = 6.8 Hz, 3H). ^{13}C NMR (101 MHz, Chloroform-*d*) δ 119.73 , 31.86 , 29.52 , 29.48 , 29.28 , 28.74 , 28.62 , 25.36 , 22.63 , 17.02 , 14.03 .

5. General procedure for the synthesis of Products 4

General procedure for the synthesis of Products 4

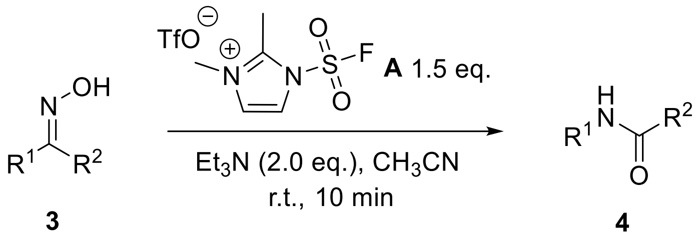

Aldoxime **1** (0.5 mmol), CH_3CN (1.0 mL) and Et_3N (139 μL, 1.0 mmol) were added into a 15 mL Schlenk flask equipped with magnetic stirrer and rubber stopper. Then the fluorosulfuryl imidazolium salt **A** (246 mg, 0.75 mmol) was dissolved in acetonitrile (1.0 mL) and that was injected slowly with a syringe into Schlenk flask, and the reaction mixture was stirred at room temperature for 10 minutes. The progress of the reaction was monitored by TLC. After completion of the reaction, the reaction mixture was diluted with 10 mL of DCM and washed with 5% HCl (2×8 mL), 5% $NaHCO_3$ (2×8 mL), saturated NaCl solution (2×8 mL) and dried over anhydrous Na_2SO_4 and the evaporation of the solvent gave a residue that was purified on silica gel column chromatography using petroleum ether and ethyl acetate.

Analytic Data of Products 4

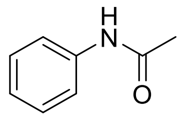

***N*-phenylacetamide (4a)**[9]

White solid (66.2 mg, 98%). ^{1}H NMR (400 MHz, Chloroform-*d*) δ 8.49 (s, 1H), 7.53 (d, *J* = 7.7 Hz, 2H), 7.30 (t, *J* = 7.9 Hz, 2H), 7.11 (t, *J* = 7.4 Hz, 1H), 2.17 (s, 3H). ^{13}C NMR (101 MHz, Chloroform-*d*) δ 169.63 , 137.88 , 128.91 , 124.54 , 120.45 , 24.15 .

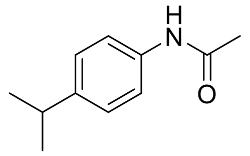

***N*-(4-isopropylphenyl)acetamide (4b)**[10]

White solid (85.9, 97%). ^{1}H NMR (400 MHz, Chloroform-*d*) δ 7.81 (s, 1H), 7.43 (d, *J* = 8.5 Hz, 2H), 7.17 (d, *J* = 8.4 Hz, 2H), 2.89 (p, *J* = 6.9 Hz, 1H), 2.16 (s, 3H), 1.24 (d, *J* = 6.9 Hz, 6H). ^{13}C NMR (101 MHz, Chloroform-*d*) δ 168.83 , 145.02 , 135.67 , 126.82 , 120.36 , 33.60 ,

24.38 , 24.04 .

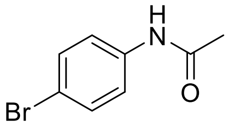

N-(4-bromophenyl)acetamide (4c)[11]

White solid (103.8 mg, 97%). ^{1}H NMR (400 MHz, Chloroform-*d*) δ 7.48 (s, 1H), 7.43 (s, 4H), 2.19 (s, 3H). ^{13}C NMR (101 MHz, Chloroform-*d*) δ 168.48 , 136.97 , 131.96 , 121.42 , 116.88 , 24.61 .

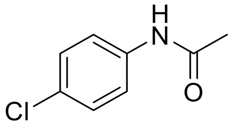

N-(4-chlorophenyl)acetamide (4d)[12]

White solid (81.4 mg, 96%). ^{1}H NMR (400 MHz, DMSO-d_6) δ 10.08 (s, 1H), 7.64 – 7.56 (m, 2H), 7.38 – 7.29 (m, 2H), 2.05 (s, 3H). ^{13}C NMR (101 MHz, DMSO-d_6) δ 168.91, 138.74, 129.03, 126.94, 120.91, 24.45.

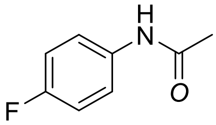

N-(4-fluorophenyl)acetamide (4e)[13]

White solid (91.8 mg, 97%).^{1}H NMR (400 MHz, Chloroform-*d*) δ 7.71 (s, 1H), 7.47 (dd, J = 8.7, 4.8 Hz, 2H), 7.01 (t, J = 8.5 Hz, 2H), 2.17 (s, 3H). ^{13}C NMR (101 MHz, Chloroform-*d*) δ 168.61, 159.39 (d, J = 243.5 Hz), 135.10 – 132.25 (m), 121.91 (d, J = 7.9 Hz), 115.61 (d, J = 22.4 Hz), 24.43.

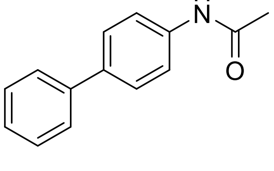

N-([1,1'-biphenyl]-4-yl)acetamide (4f)[14]

White solid (97.2 mg, 92%). ^{1}H NMR (400 MHz, DMSO-d_6) δ 10.05 (s, 1H), 7.68 (d, *J* = 8.6 Hz, 2H), 7.61 (t, *J* = 8.5 Hz, 4H), 7.43 (t, *J* = 7.6 Hz, 2H), 7.31 (t, *J* = 7.3 Hz, 1H), 2.07 (s, 3H). ^{13}C NMR (101 MHz, DMSO-d_6) δ 168.82 , 140.20 , 139.28 , 135.10 , 129.35 , 127.42 , 127.34 , 126.67 , 119.77 , 24.51 .

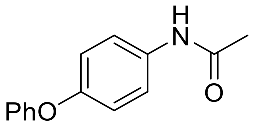

***N*-(4-phenoxyphenyl)acetamide (4g)**[10]

White solid (106.8 mg, 94%). ^{1}H NMR (400 MHz, Chloroform-*d*) δ 7.78 (d, *J* = 9.8 Hz, 1H), 7.48 (d, *J* = 8.9 Hz, 2H), 7.33 (t, *J* = 7.9 Hz, 2H), 7.10 (t, *J* = 7.4 Hz, 1H), 6.99 (dd, *J* = 8.2, 4.8 Hz, 4H), 2.18 (s, 3H). ^{13}C NMR (101 MHz, Chloroform-*d*) δ 168.70 , 157.49 , 153.51 , 133.43 , 129.76 , 123.13 , 121.88 , 119.55 , 118.46 , 24.38 .

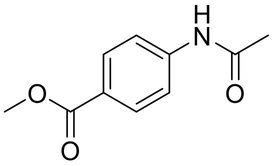

Methyl 4-acetamidobenzoate (4h)[15]

White solid (91.7 mg, 95%). ^{1}H NMR (400 MHz, Chloroform-*d*) δ 8.39 (s, 1H), 7.98 (d, *J* = 8.1 Hz, 2H), 7.64 (d, *J* = 8.4 Hz, 2H), 3.90 (s, 3H), 2.21 (s, 3H). ^{13}C NMR (101 MHz, Chloroform-*d*) δ 169.21 , 166.84 , 142.44 , 130.77 , 125.38 , 118.95 , 52.11 , 24.67 .

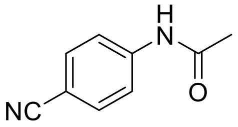

***N*-(4-cyanophenyl)acetamide (4i)**[16]

White solid (64.8 mg, 81%). ^{1}H NMR (400 MHz, DMSO-d_6) δ 10.39 (s, 1H), 7.76 (s, 4H), 2.09 (s, 3H). ^{13}C NMR (101 MHz, DMSO-d_6) δ 169.66 , 143.94 , 133.71 , 119.57 , 119.37 , 105.14 , 24.67.

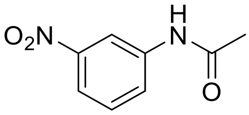

***N*-(3-nitrophenyl)acetamide (4j)**[13]

Yellow solid (85.6 mg, 95%). ^{1}H NMR (400 MHz, DMSO-*d*$_6$) δ 10.44 (s, 1H), 8.62 (t, *J* = 2.0 Hz, 1H), 7.88 (dt, *J* = 8.1, 2.5 Hz, 2H), 7.59 (t, *J* = 8.2 Hz, 1H), 2.10 (s, 3H). ^{13}C NMR (101 MHz, DMSO-*d*$_6$) δ 169.55 , 148.40 , 140.85 , 130.59 , 125.32 , 117.99 , 113.42 , 24.52 .

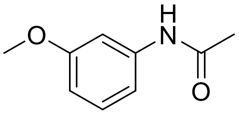

***N*-(3-methoxyphenyl)acetamide (4k)**[13]

White solid (80.1 mg, 97%). ^{1}H NMR (400 MHz, Chloroform-*d*) δ 8.12 (s, 1H), 7.19 (t, *J* = 8.1 Hz, 1H), 7.02 (d, *J* = 7.9 Hz, 1H), 6.71 – 6.59 (m, 1H), 3.77 (s, 3H), 2.16 (s, 3H). ^{13}C NMR (101 MHz, Chloroform-*d*) δ 169.00 , 160.06 , 139.26 , 129.64 , 112.25 , 109.98 , 105.89 , 55.28 , 24.56 .

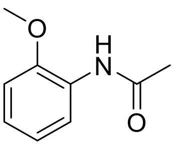

***N*-(2-methoxyphenyl)acetamide (4l)**[15]

White solid (79.3 mg, 96%). ^{1}H NMR (400 MHz, Chloroform-*d*) δ 8.36 (d, *J* = 9.4 Hz, 1H), 7.82 (s, 1H), 7.04 (td, *J* = 7.8, 1.6 Hz, 1H), 6.96 (td, *J* = 7.7, 1.3 Hz, 1H), 6.87 (dd, *J* = 8.1, 1.1 Hz, 1H), 3.87 (s, 3H), 2.20 (s, 3H). ^{13}C NMR (101 MHz,) δ 168.27 , 147.71 , 127.69 , 123.64 , 121.04 , 119.80 , 109.89 , 55.64 , 24.93 .

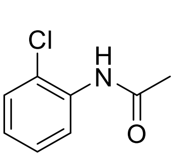

***N*-(2-chlorophenyl)acetamide (4m)**[13]

White solid (81.5 mg, 95%). ^{1}H NMR (400 MHz, Chloroform-*d*) δ 8.37 (d, *J* = 8.1 Hz, 1H), 7.67 (s, 1H), 7.43 – 7.34 (m, 1H), 7.32 – 7.24 (m, 1H), 7.05 (t, *J* = 7.3 Hz, 1H), 2.26 (s, 3H). ^{13}C NMR (101 MHz, Chloroform-*d*) δ 168.32 , 134.60 , 128.99 , 127.75 , 124.65 , 122.57 , 121.68 , 24.90 .

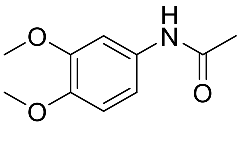

***N*-(3,4-dimethoxyphenyl)acetamide (4n)**[13]

White solid (94.6 mg, 97%). ^{1}H NMR (400 MHz, DMSO-d_6) δ 9.82 (s, 1H), 7.29 (d, J = 2.3 Hz, 1H), 7.09 (dd, J = 8.7, 2.4 Hz, 1H), 6.86 (d, J = 8.7 Hz, 1H), 3.71 (s, 3H), 3.70 (s, 3H), 2.01 (s, 3H). ^{13}C NMR (101 MHz, DMSO-d_6) δ 168.37 , 148.92 , 145.09 , 133.44 , 112.43 , 111.40 , 104.73 , 56.11 , 55.75 , 24.30 .

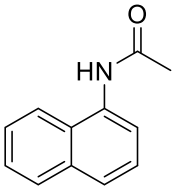

***N*-(naphthalen-1-yl)acetamide (4o)**[10]

White solid (86.1 mg, 93%). ^{1}H NMR (400 MHz, DMSO-d_6) δ 9.96 (s, 1H), 8.13 – 8.05 (m, 1H), 7.94 (dd, J = 6.2, 3.2 Hz, 1H), 7.76 (d, J = 8.2 Hz, 1H), 7.71 (d, J = 7.3 Hz, 1H), 7.55 (td, J = 6.9, 6.0, 3.6 Hz, 2H), 7.52 – 7.45 (m, 1H), 2.20 (s, 3H). ^{13}C NMR (101 MHz, DMSO-d_6) δ 169.42 , 134.17 , 128.58 , 128.14 , 126.44 , 126.21 , 126.04 , 125.51 , 123.22 , 122.01 , 23.97 .

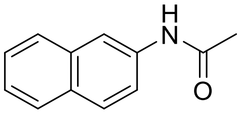

***N*-(naphthalen-2-yl)acetamide (4p)**[12]

White solid (88.0 mg, 95%). ^{1}H NMR (400 MHz, Chloroform-d) δ 8.21 (s, 1H), 7.95 (s, 1H), 7.77 (dt, J = 8.5, 4.3 Hz, 3H), 7.51 – 7.38 (m, 3H), 2.23 (s, 3H). ^{13}C NMR (101 MHz, Chloroform-d) δ 168.99 , 135.44 , 133.81 , 130.65 , 128.73 , 127.67 , 127.56 , 126.49 , 125.04 , 120.05 , 116.84 , 24.66 .

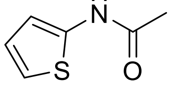

***N*-(thiophen-2-yl)acetamide (4q)**[17]

White solid (56.5 mg, 80%). ^{1}H NMR (400 MHz, DMSO-d_6) δ 11.14 (s, 1H), 6.94 – 6.86 (m, 1H), 6.83 (dd, J = 5.5, 3.7 Hz, 1H), 6.63 (dd, J = 3.7, 1.4 Hz, 1H), 2.06 (s, 3H). ^{13}C NMR (101 MHz, DMSO-d_6) δ 166.67 , 140.36 , 124.32 , 117.03 , 110.69 , 22.98 .

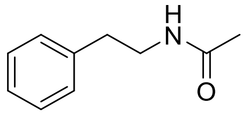

***N*-phenethylacetamide (4r)**[15]

White solid (71.8 mg, 88 %). ^{1}H NMR (400 MHz, Chloroform-*d*) δ 7.33 (t, *J* = 7.3 Hz, 2H), 7.24 (dd, *J* = 19.8, 7.2 Hz, 3H), 5.66 (s, 1H), 3.53 (q, *J* = 6.4 Hz, 2H), 2.84 (t, *J* = 6.9 Hz, 2H), 1.96 (s, 3H). ^{13}C NMR (101 MHz, Chloroform-*d*) δ 170.22 , 138.88 , 128.77 , 128.68 , 126.55 , 40.72 , 35.63 , 23.37 .

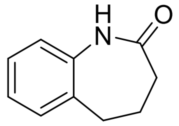

1,3,4,5-tetrahydro-2H-1-benzazepin-2-one (4s)[15]

White solid (62.8 mg, 78%). ^{1}H NMR (400 MHz, Chloroform-*d*) δ 7.85 (s, 1H), 7.25 (d, *J* = 8.3 Hz, 2H), 7.20 – 7.12 (m, 1H), 7.01 (d, *J* = 7.7 Hz, 1H), 2.83 (t, *J* = 7.2 Hz, 2H), 2.39 (t, *J* = 7.2 Hz, 2H), 2.27 (q, *J* = 7.0 Hz, 2H). ^{13}C NMR (101 MHz, Chloroform-*d*) δ 175.24 , 137.76 , 134.39 , 129.91 , 127.50 , 125.79 , 121.84 , 32.74 , 30.33 , 28.49 .

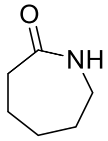

ε-caprolactam (4t)[12]

White solid (40.7 mg, 72%). ^{1}H NMR (400 MHz, Chloroform-*d*) δ 7.17 (s, 1H), 3.25 – 3.04 (m, 2H), 2.40 (d, *J* = 5.4 Hz, 2H), 1.73 – 1.54 (m, 6H). ^{13}C NMR (101 MHz, Chloroform-*d*) δ 179.53 , 42.72 , 36.73 , 30.58 , 29.67 , 23.19 .

6. Gram-scale production of 2i and 4a by crystallization

Procedure for the synthesis of 2i

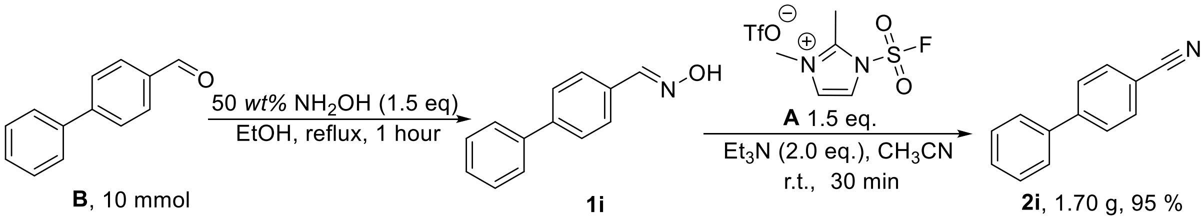

4-phenylbenzaldehyde **B** (1.82 g, 10 mmol), EtOH (100 mL, 0.1 M) and 50 wt% aqueous hydroxylamine solution (0.77 g, 15 mmol) were added into a 250 mL round-bottom flask equipped with magnetic stirrer, then the mixture was heated at reflux for an hour. After 4-phenylbenzaldehyde **B** was completely consumed (monitored by TLC) and the reaction mixture was concentrated under reduced pressure to give the crude 4-phenylbenzaldoxime **1i**. The crude 4-phenylbenzaldoxime **1i** (without any purification), CH_3CN (50 mL) and Et_3N (2.8 mL, 20 mmol) were added into a 250 mL round-bottom flaske quipped with magnetic stirrer and rubber stopper. Then the fluorosulfuryl imidazolium salt **A** (4.92 g, 15 mmol) was dissolved in acetonitrile (50 mL) and that was injected slowly with a syringe into flask, and the reaction mixture was stirred at room temperature for 30 minutes. The progress of the reaction was monitored by TLC. After completion of the reaction, the reaction mixture was concentrated under reduced pressure to remove acetonitrile and diluted with 100 mL of DCM and washed with 5% HCl (100 mL), saturated NaCl solution (100 mL) and dried over anhydrous Na_2SO_4 and the evaporation of the appropriate amount solvent. While white soild was crystallized and washed with petroleum ether to give 4-phenylbenzonitrile **2i** (1.70 g, 95 %).

Procedure for the synthesis of 4a

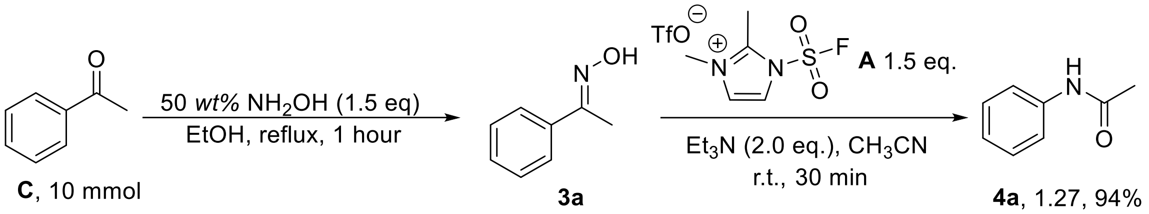

Acetophenone **C** (1.20 g, 10 mmol), EtOH (100 mL, 0.1 M) and 50 wt% aqueous hydroxylamine solution (0.77 g, 15 mmol) were added into a 250 mL round-bottom flask equipped with magnetic stirrer, then the mixture was heated at reflux for an hour. After Acetophenone **C** was completely consumed (monitored by TLC) and the reaction mixture was

concentrated under reduced pressure to give the crude acetophenone oxime **3a**. The crude acetophenone oxime **3a** (without any purification), CH_3CN (50 mL) and Et_3N (2.8 mL, 20 mmol) were added into a 250 mL round-bottom flaske quipped with magnetic stirrer and rubber stopper. Then the fluorosulfuryl imidazolium salt **A** (4.92 g, 15 mmol) was dissolved in acetonitrile (50 mL) and that was injected slowly with a syringe into flask, and the reaction mixture was stirred at room temperature for 30 minutes. The progress of the reaction was monitored by TLC. After completion of the reaction, the reaction mixture was concentrated under reduced pressure to remove appropriate amount acetonitrile. While white soild was crystallized and washed with petroleum ether to give acetanilide **4a** (1.70 g, 94 %).

7. Synthesis of precursors for drugs

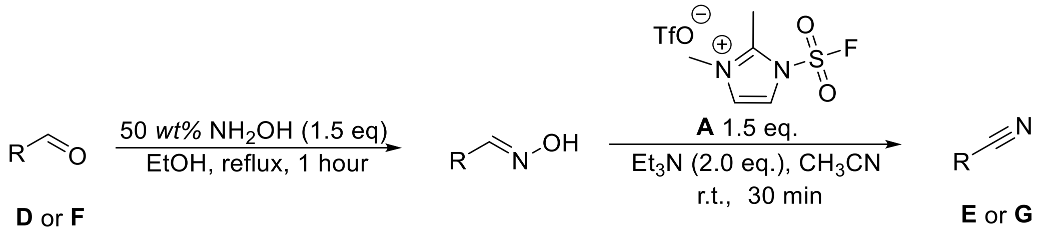

Aldehyde **D** or **F** (0.5 mmol), EtOH (5 mL, 0.1 M) and 50 wt% aqueous hydroxylamine solution (24.7 μL, 0.75 mmol) were added into a 10 mL round-bottom flask equipped with magnetic stirrer, then the mixture was heated at reflux for an hour. After aldehyde was completely consumed (monitored by TLC) and the reaction mixture was concentrated under reduced pressure to give the crude aldoxime. The crude aldoxime (without any purification), CH_3CN (3 mL) and Et_3N (139 μL, 1 mmol) were added into a 10 mL round-bottom flaske quipped with magnetic stirrer and rubber stopper. Then the fluorosulfuryl imidazolium salt **A** (246 mg, 0.75 mmol) was dissolved in acetonitrile (2 mL) and that was injected slowly with a syringe into flask, and the reaction mixture was stirred at room temperature for 30 minutes. The progress of the reaction was monitored by TLC. After completion of the reaction, the reaction mixture was diluted with 20 mL of DCM and washed with 5% HCl (2×20 mL), 5% $NaHCO_3$ (2×20 mL), saturated NaCl solution (2×20 mL) and dried over anhydrous Na_2SO_4 and the evaporation of the solvent gave a residue that was purified on silica gel column chromatography using petroleum ether and ethyl acetate.

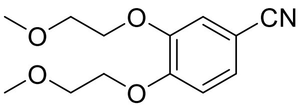

3,4-Bis(2-methoxyethoxy)benzaldehyde (E)[3]

yellow oil (119.3 mg, 95%).^{1}H NMR (400 MHz, Chloroform-*d*) δ 7.30 – 7.25 (m, 1H), 7.16 (d, *J* = 1.7 Hz, 1H), 6.94 (d, *J* = 8.4 Hz, 1H), 4.19 (dt, *J* = 15.5, 4.7 Hz, 4H), 3.80 (q, *J* = 5.5 Hz, 4H), 3.46 (s, 6H). ^{13}C NMR (101 MHz, Chloroform-*d*) δ 152.95 , 148.92 , 126.85 , 119.18 , 117.08 , 113.53 , 104.20 , 70.82 , 70.71 , 69.08 , 68.64 , 59.32 , 59.28 .

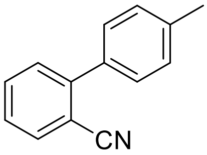

4'-methyl-2-biphenylcarbonitrile (**G**)[18]

White solid (93.7 mg, 97%). ^{1}H NMR (400 MHz, Chloroform-*d*) δ 7.81 – 7.74 (m, 1H), 7.66 (td, *J* = 7.7, 1.4 Hz, 1H), 7.52 (ddd, *J* = 14.5, 7.2, 1.2 Hz, 3H), 7.44 (td, *J* = 7.6, 1.2 Hz, 1H), 7.33 (d, *J* = 7.9 Hz, 2H), 2.45 (s, 3H). ^{13}C NMR (101 MHz, Chloroform-*d*) δ 145.56 , 138.73 , 135.30 , 133.75 , 132.81 , 130.01 , 129.48 , 128.65 , 127.32 , 118.93 , 111.21 , 21.29 .

8. References

[1] P. Wang, H. Zhang, X. Nie, T. Xu and S. Liao, *Nat. Commun.*, 2022, **13,** 3370.
[2] S. R. Mudshinge, C. S. Potnis, B. Xu and G. B. Hammond, *Green Chem.*, 2020, **22**, 4161.
[3] W.-Y. Fang and H.-L. Qin, *J. Org. Chem.*, 2019, **84**, 5803.
[4] K. Hyodo, K. Togashi, N. Oishi, G. Hasegawa and K. Uchida, *Org. Lett.*, 2017, **19**, 3005.
[5] Z. Shu, Y. Ye, Y. Deng, Y. Zhang and J. Wang, *Angew. Chem., Int. Ed.*, 2013, **52**, 10573.
[6] Y. Zhao, G. Mei, H. Wang, G. Zhang and C. Ding, *Synlett*, 2019, **30**, 1484.
[7] L. Zhang and X. Hu, *Chem. Sci.*, 2017, **8**, 7009.
[8] R. Y. Liu, M. Bae and S. L. Buchwald, *J. Am. Chem. Soc.*, 2018, **140**, 1627.
[9] N. Tandon, S. M. Patil, R. Tandon and P. Kumar, *RSC Adv.*, 2021, **11**, 21291.
[10] L. Tang, Z. L. Wang, H. L. Wan, Y. H. He and Z. Guan, *Org. Lett.*, 2020, **22**, 6182.
[11] D. Dev, T. Kalita, T. Mondal and B. Mandal, *Adv. Synth. Catal.*, 2021, **363**, 1427.
[12] Y. Gao, J. Liu, Z. Li, T. Guo, S. Xu, H. Zhu, F. Wei, S. Chen, H. Gebru and K. Guo, *J. Org. Chem.*, 2018, **83**, 2040.
[13] G. Zhang, Y. Zhao, L. Xuan and C. Ding, *Eur. J. Org. Chem.*, 2019, **2019**, 4911.
[14] R. J. Tang, T. Milcent and B. Crousse, *J. Org. Chem.*, 2018, **83**, 930.
[15] J. Gurjar and V. V. Fokin, *Chem. Eur. J.*, 2020, **26**, 10402.
[16] Z. Zand, F. Kazemi and A. Partovi, *J. Photoch. Boi. B,* 2015, **152**, 58.
[17] S.-K. Xiang, D.-X. Zhang, H. Hu, J.-L. Shi, L.-G. Liao, C. Feng, B.-Q. Wang, K.-Q. Zhao, P. Hu, H. Yang and W.-H. Yu, *Adv. Synth. Catal.*, 2013, **355**, 1495.
[18] K. L. Wilson, J. Murray, C. Jamieson and A. J. Watson, *Synlett*, 2018, **29**, 650.

^{1}H NMR Spectra of A

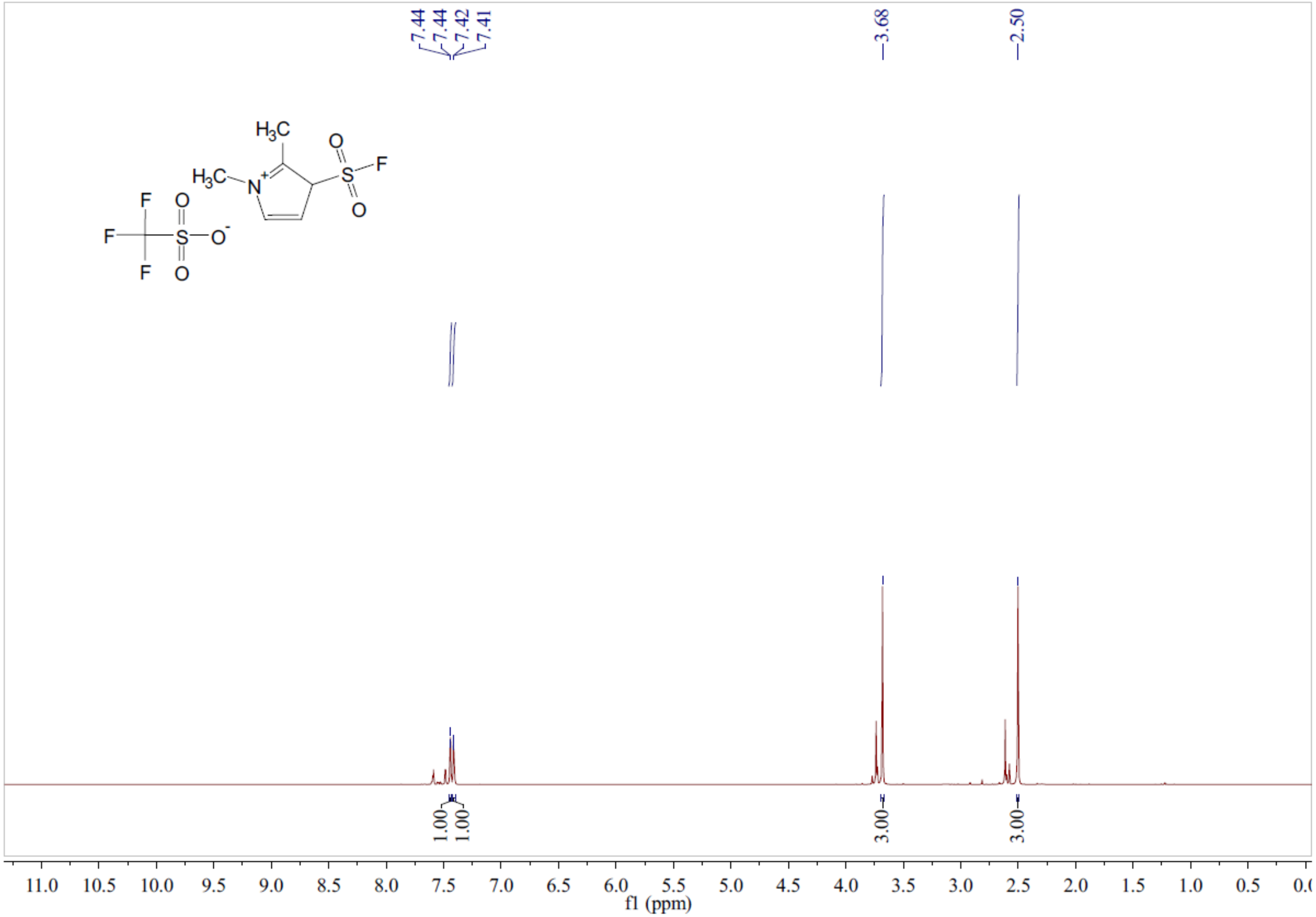

^{19}F NMR Spectra of A

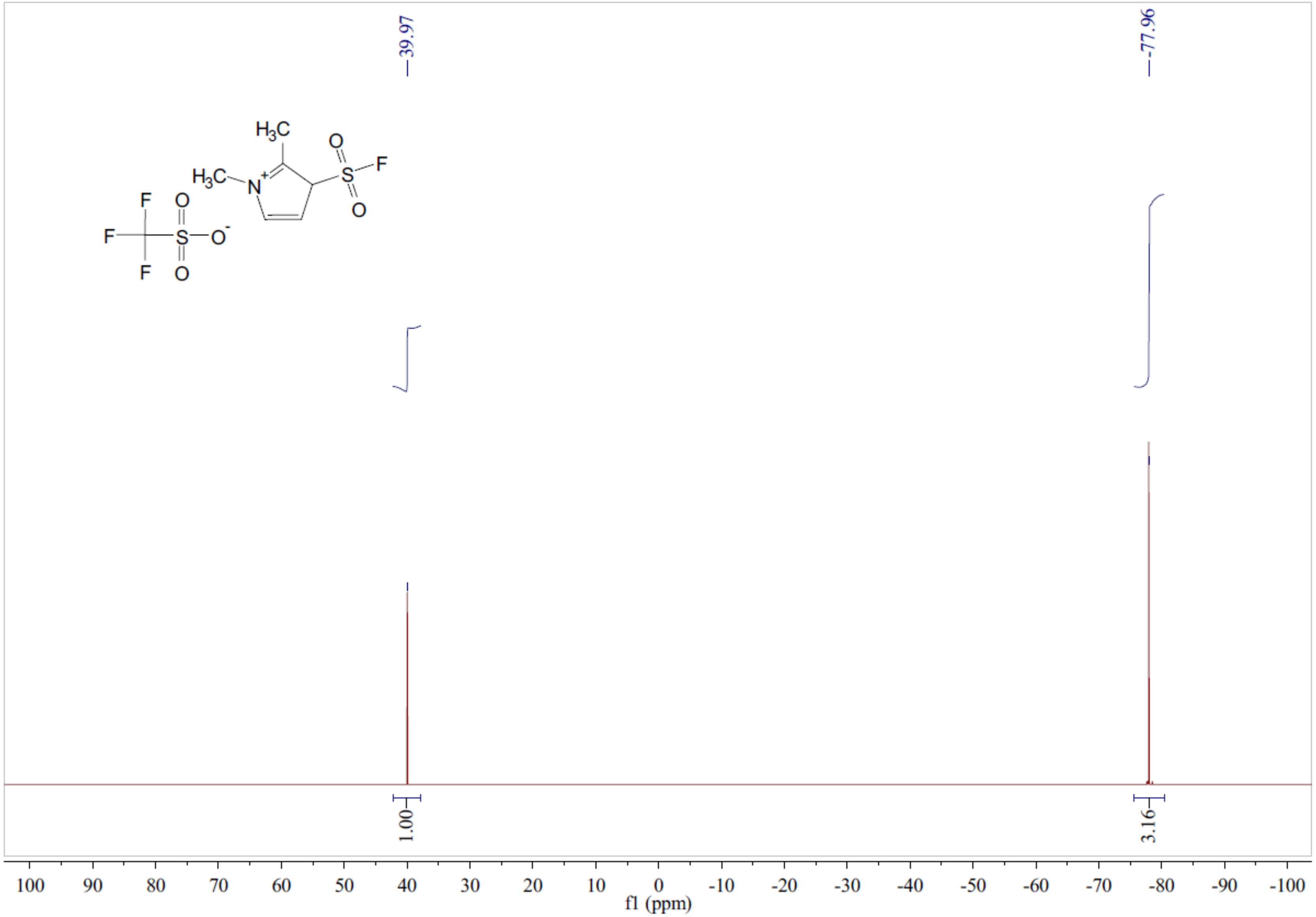

^{1}H NMR Spectra of 2a

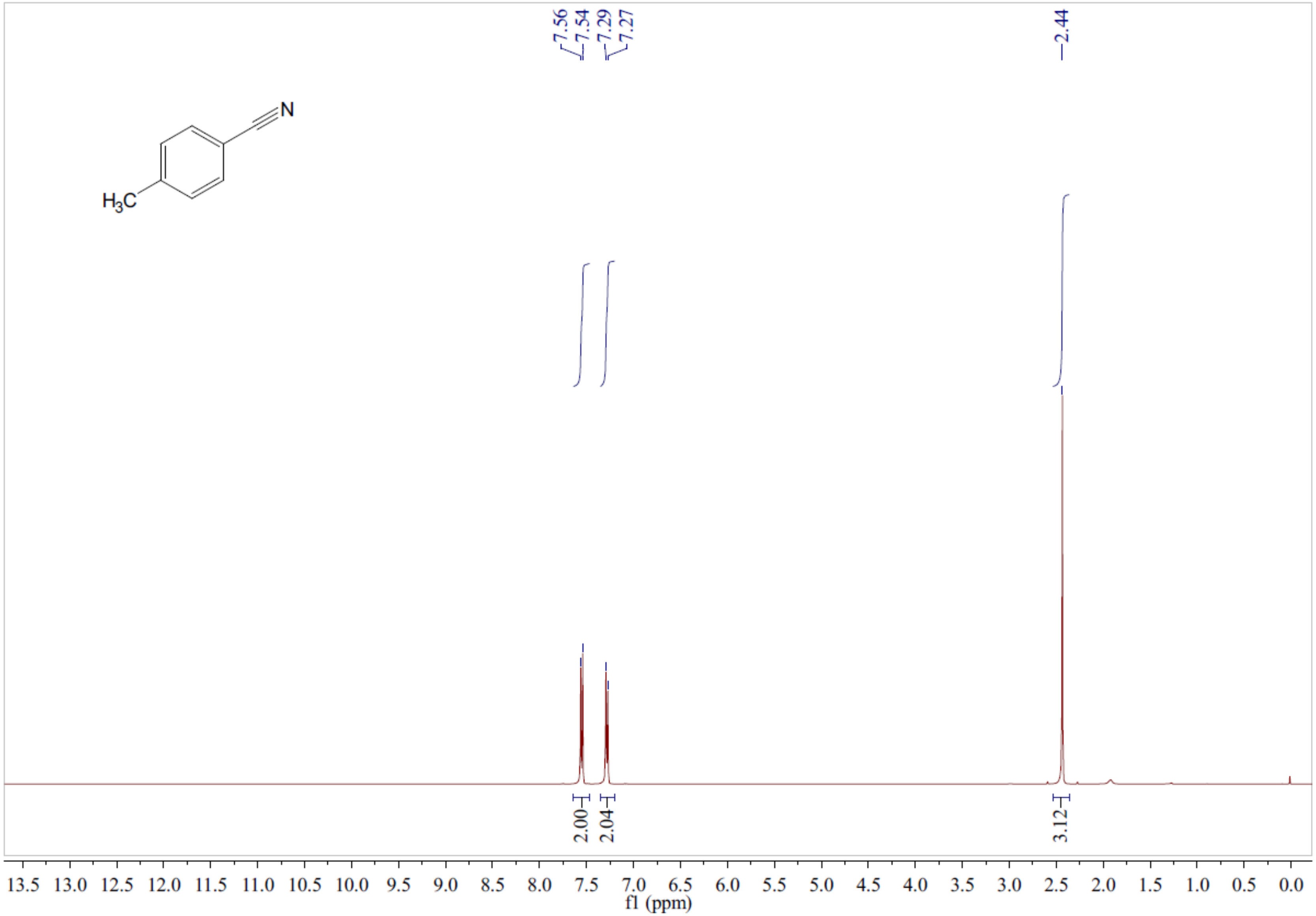

^{13}C NMR Spectra of 2a

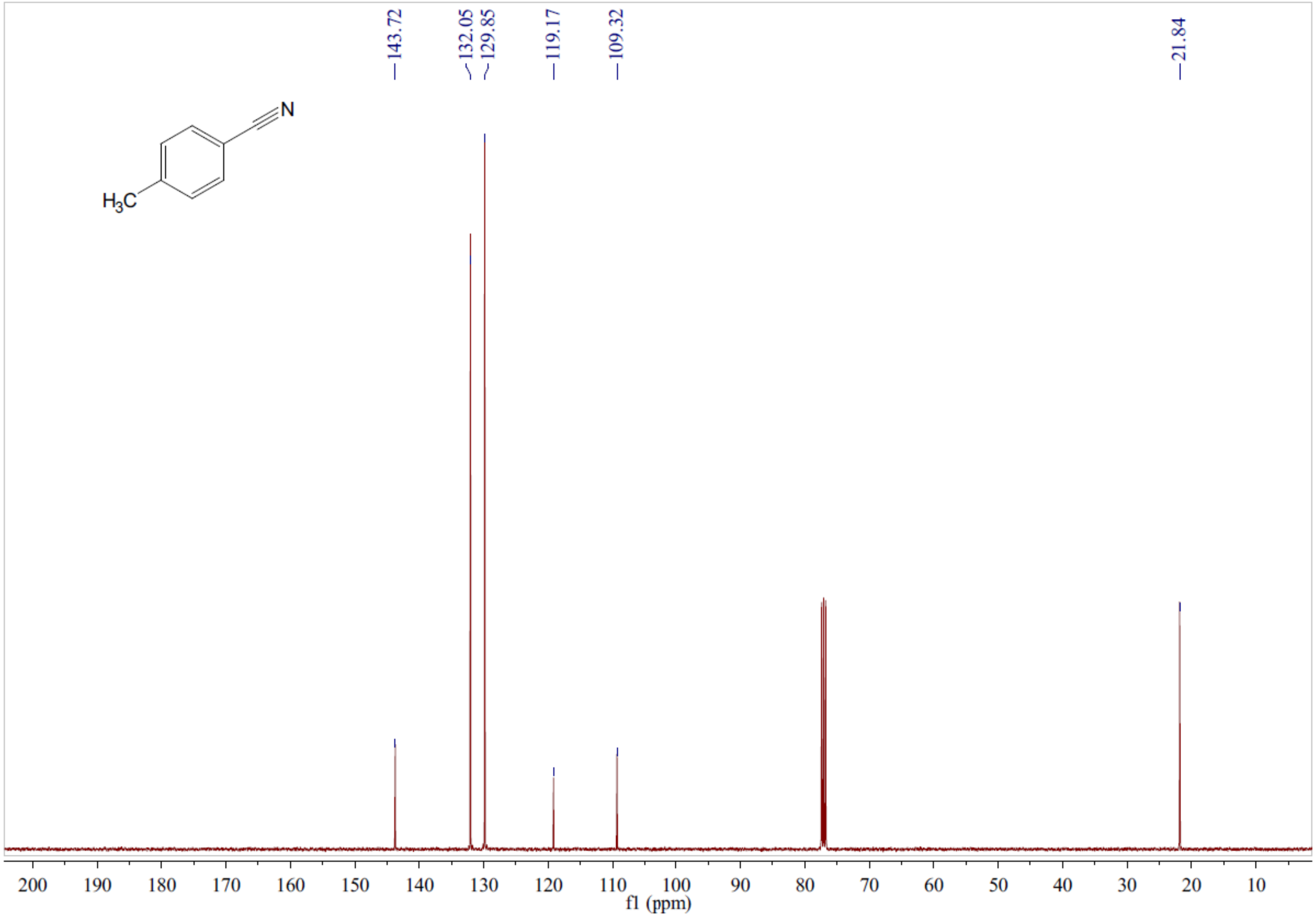

^{1}H NMR Spectra of 2b

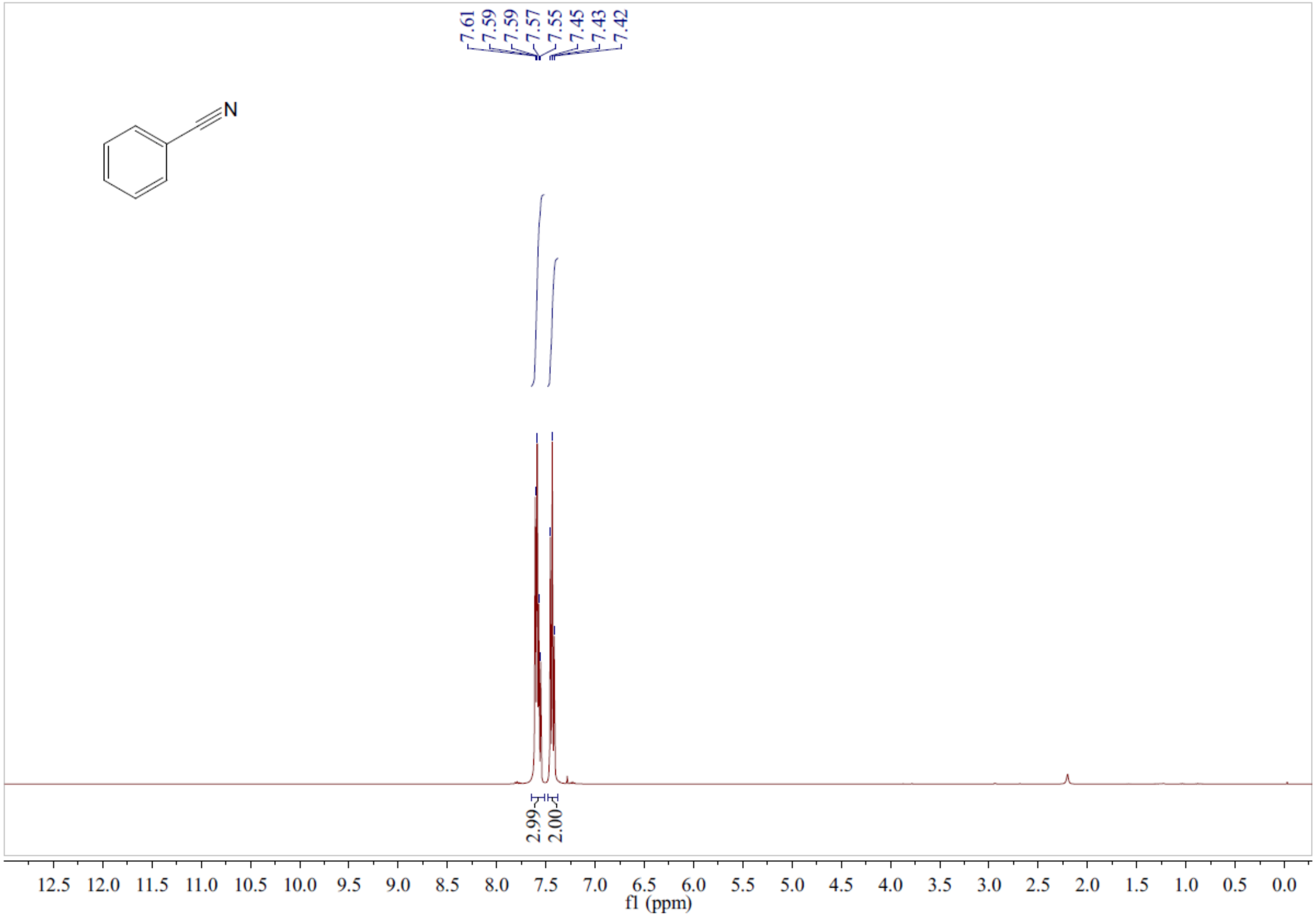

^{13}C NMR Spectra of 2b

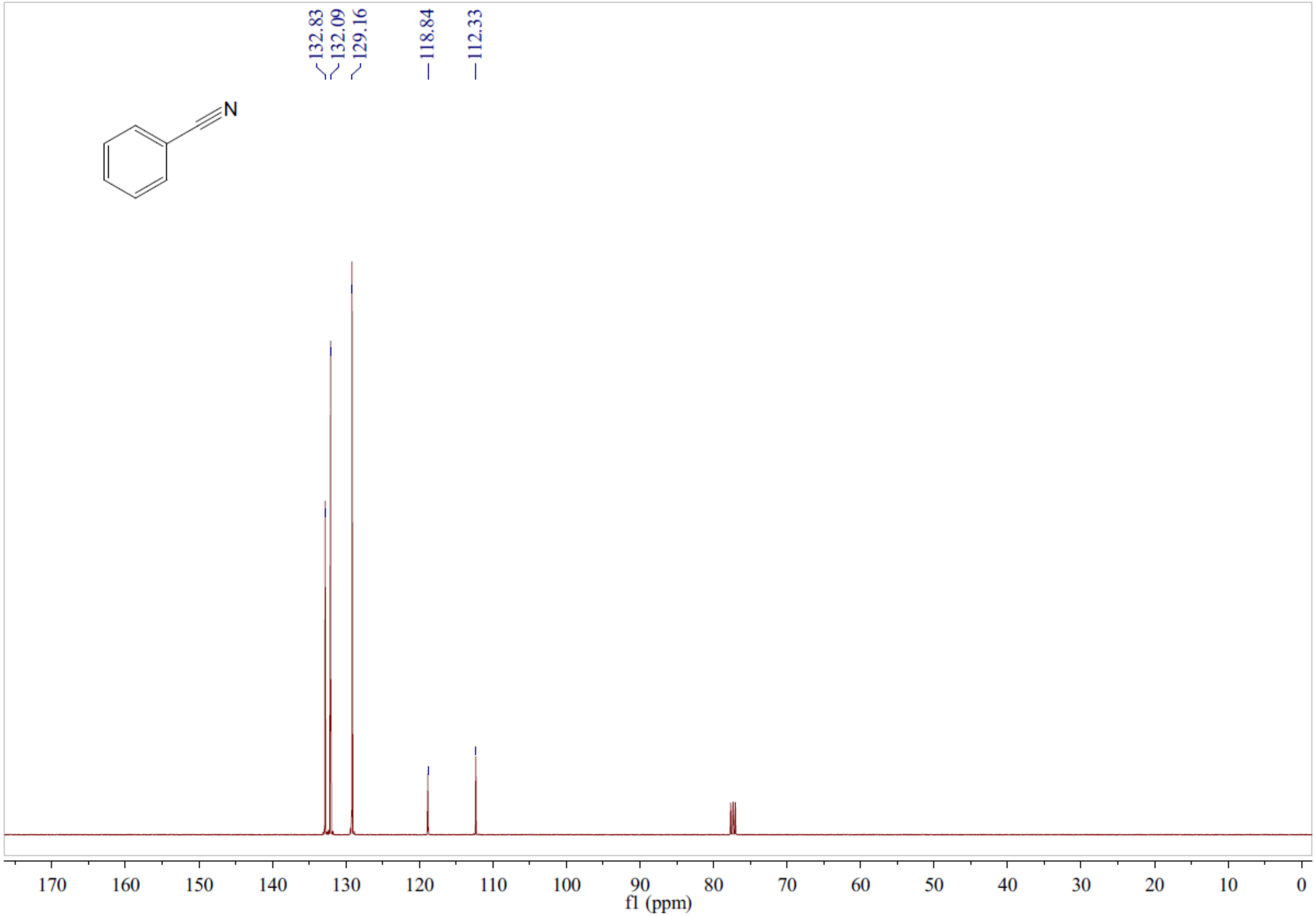

¹H NMR Spectra of 2c

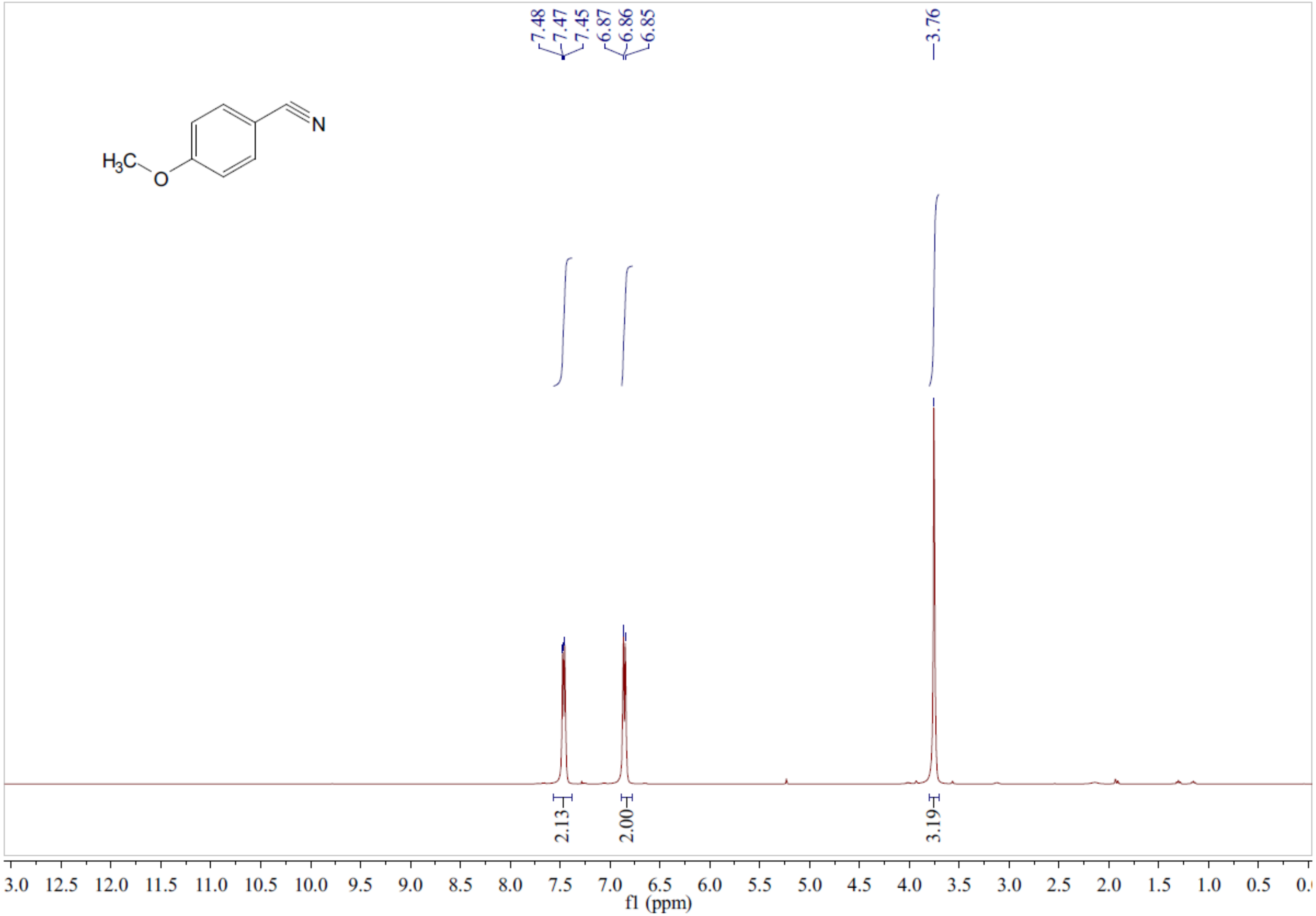

^{13}C NMR Spectra of 2c

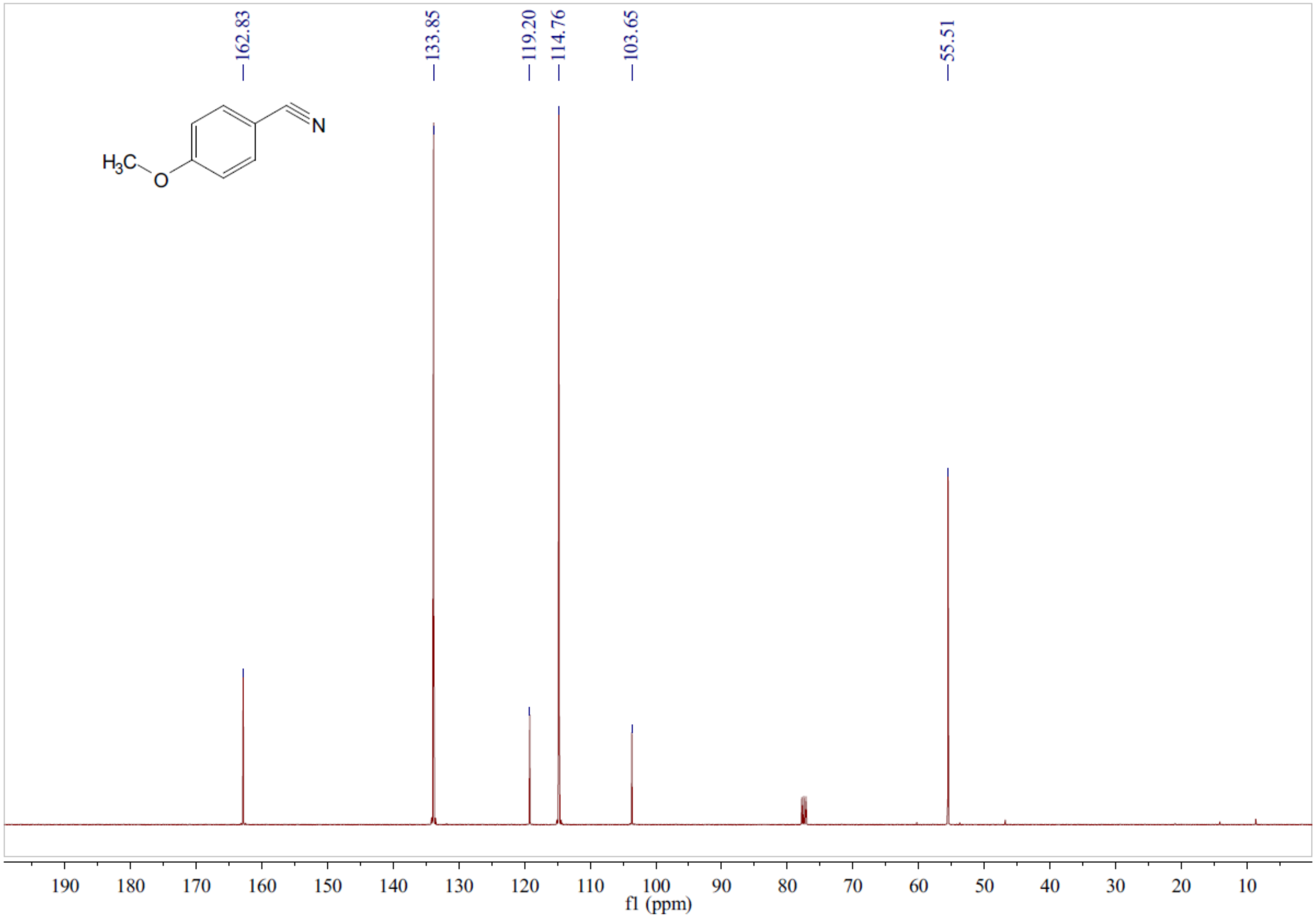

^{1}H NMR Spectra of 2d

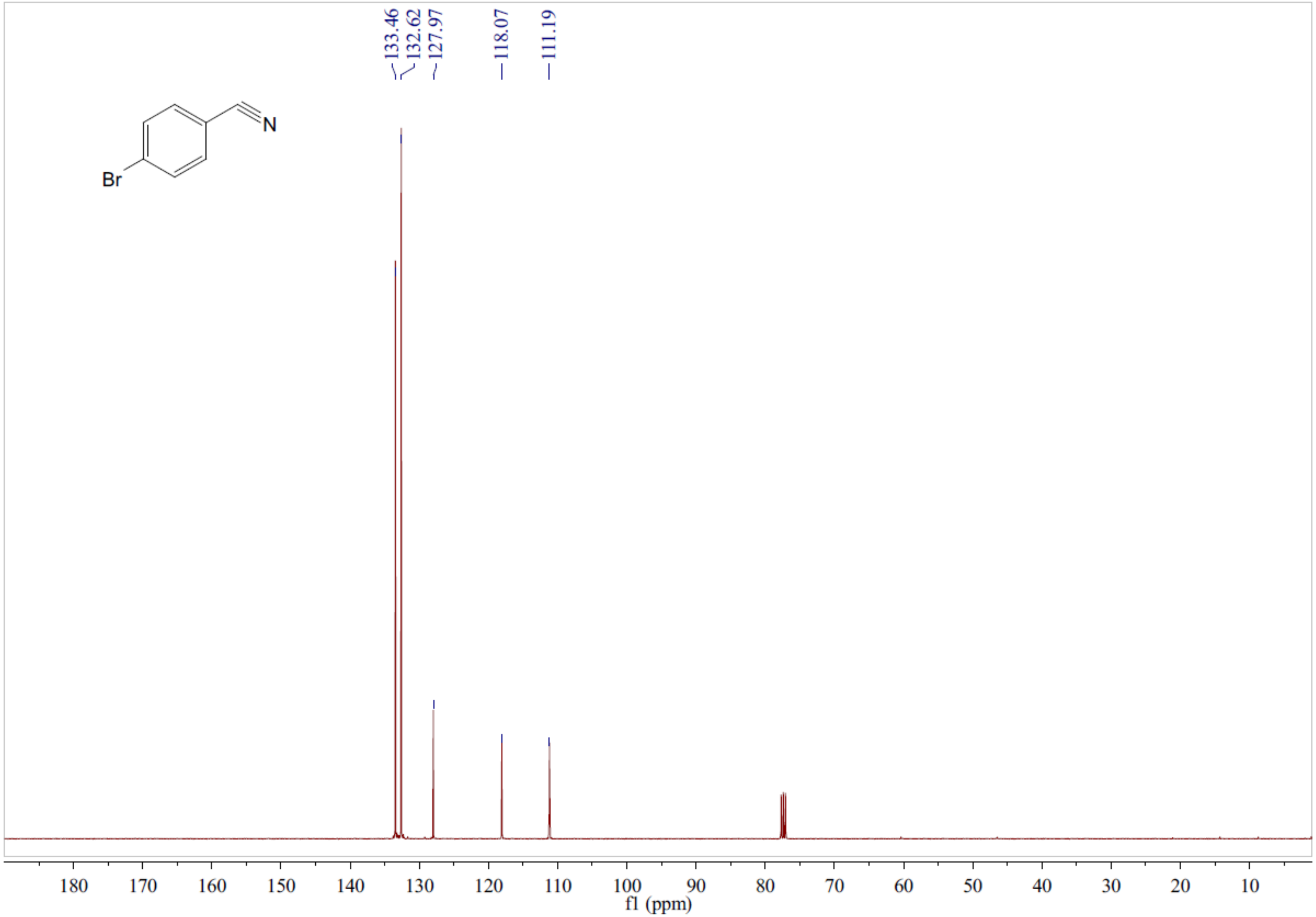

^{13}C NMR Spectra of 2d

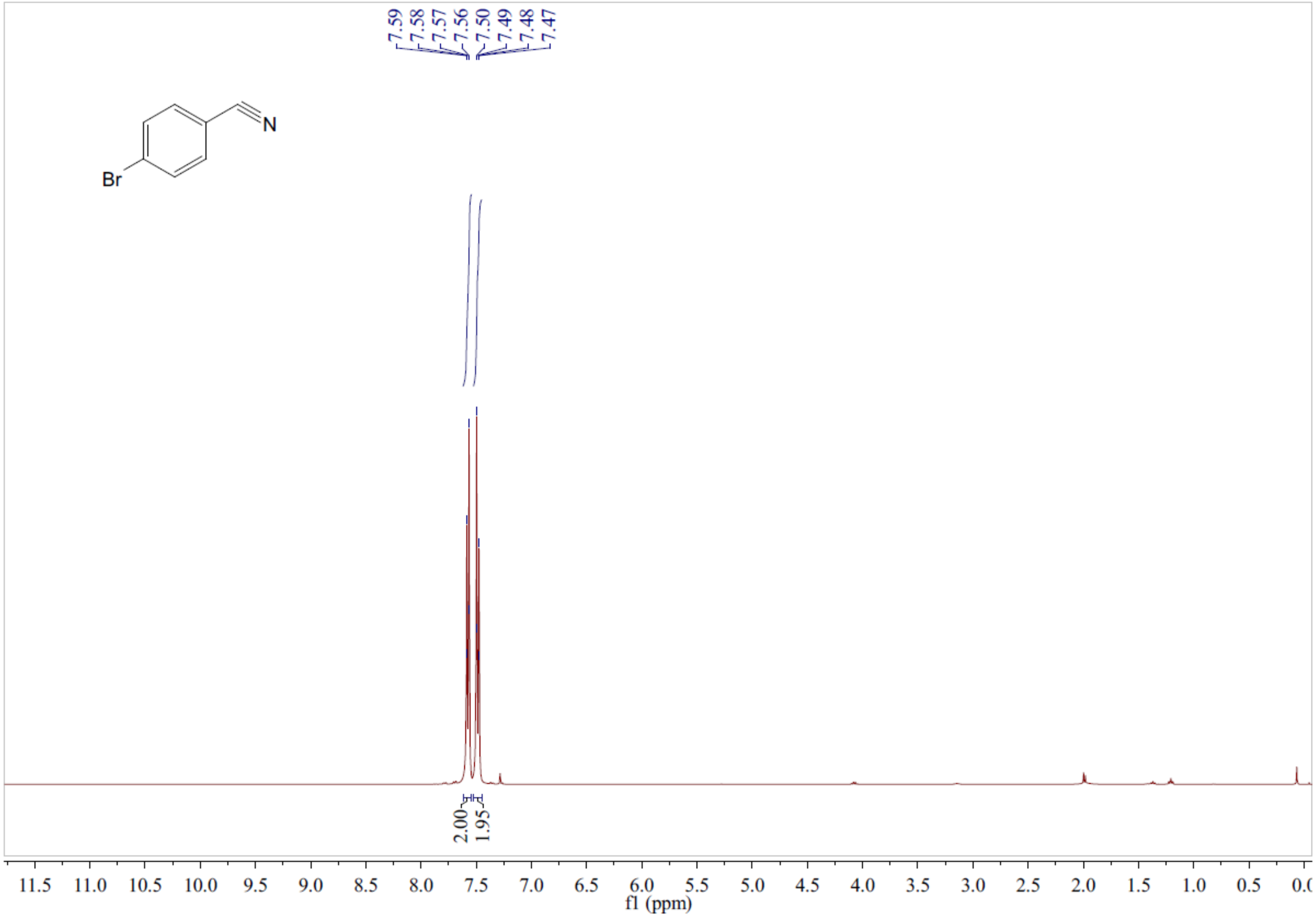

^{1}H NMR Spectra of 2e

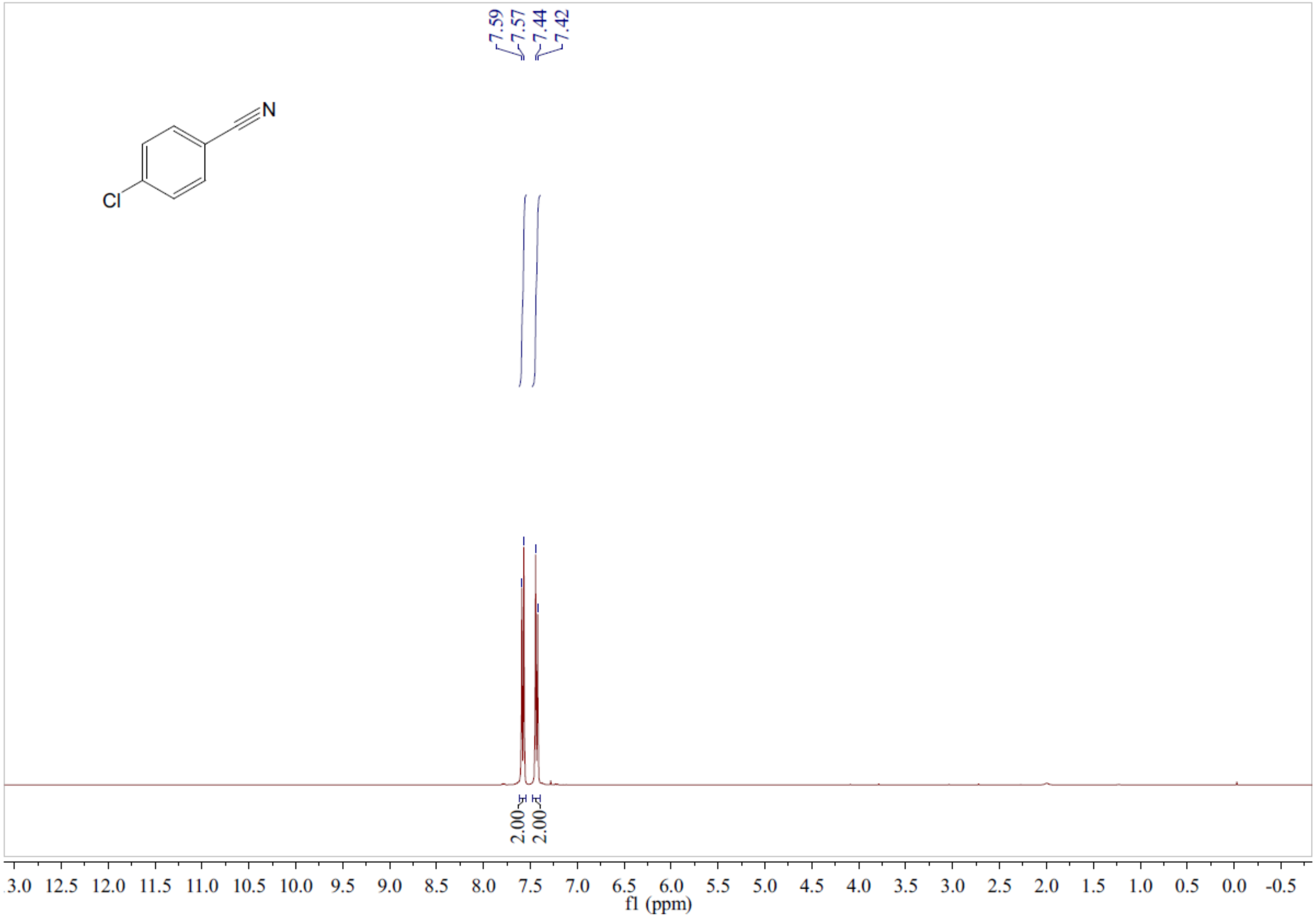

^{13}C NMR Spectra of 2e

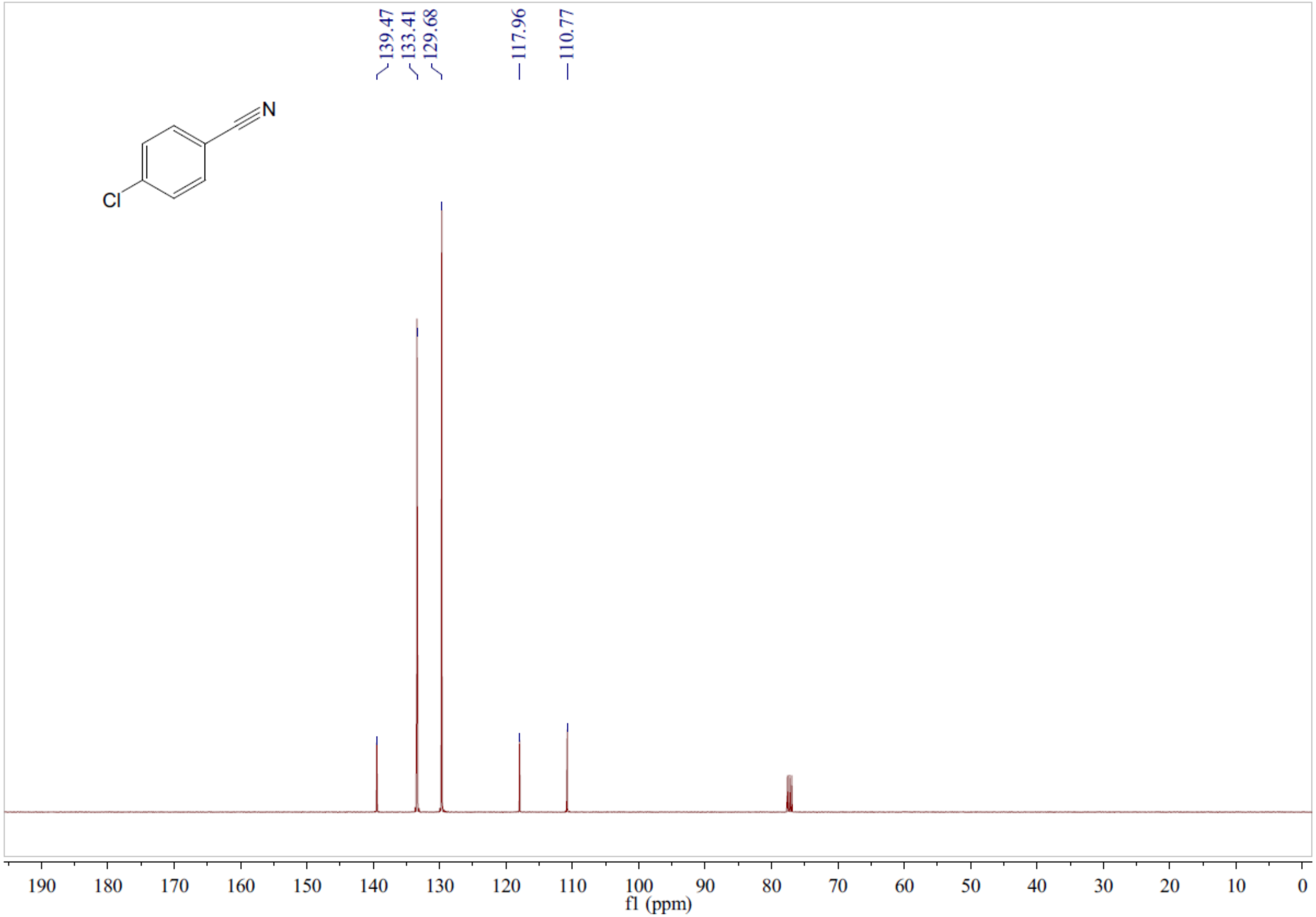

^{1}H NMR Spectra of 2f

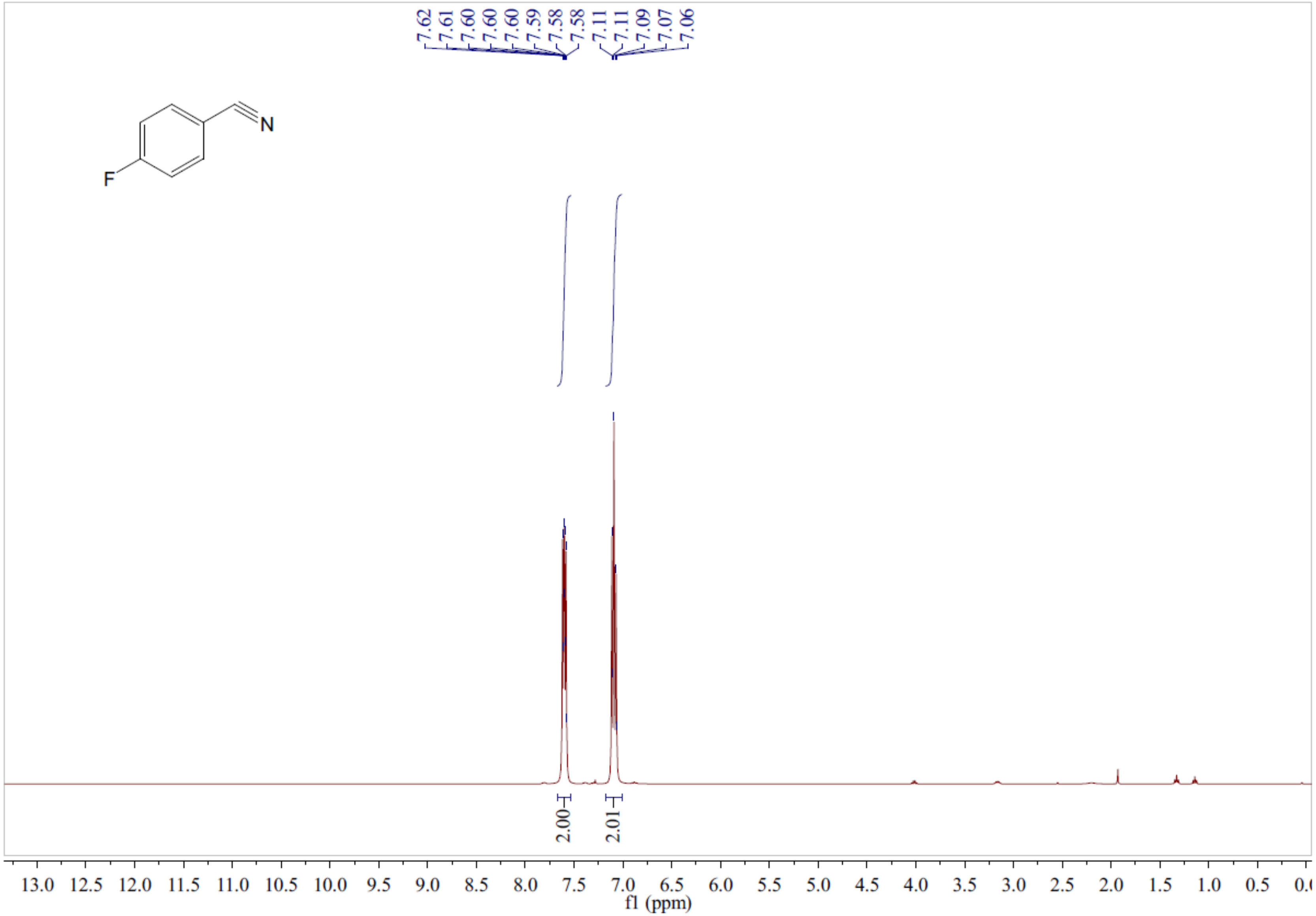

^{13}C NMR Spectra of 2f

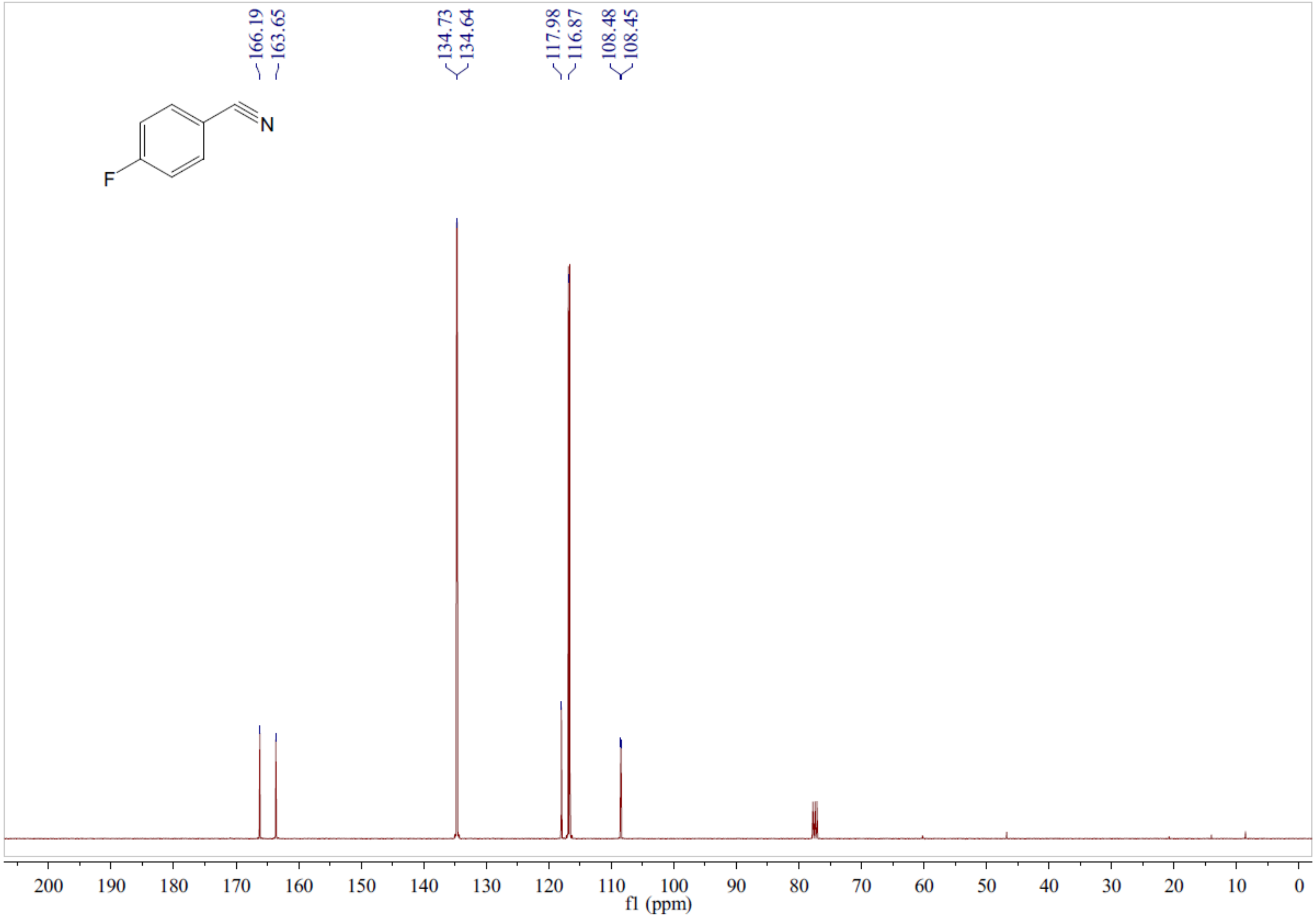

^{1}H NMR Spectra of 2g

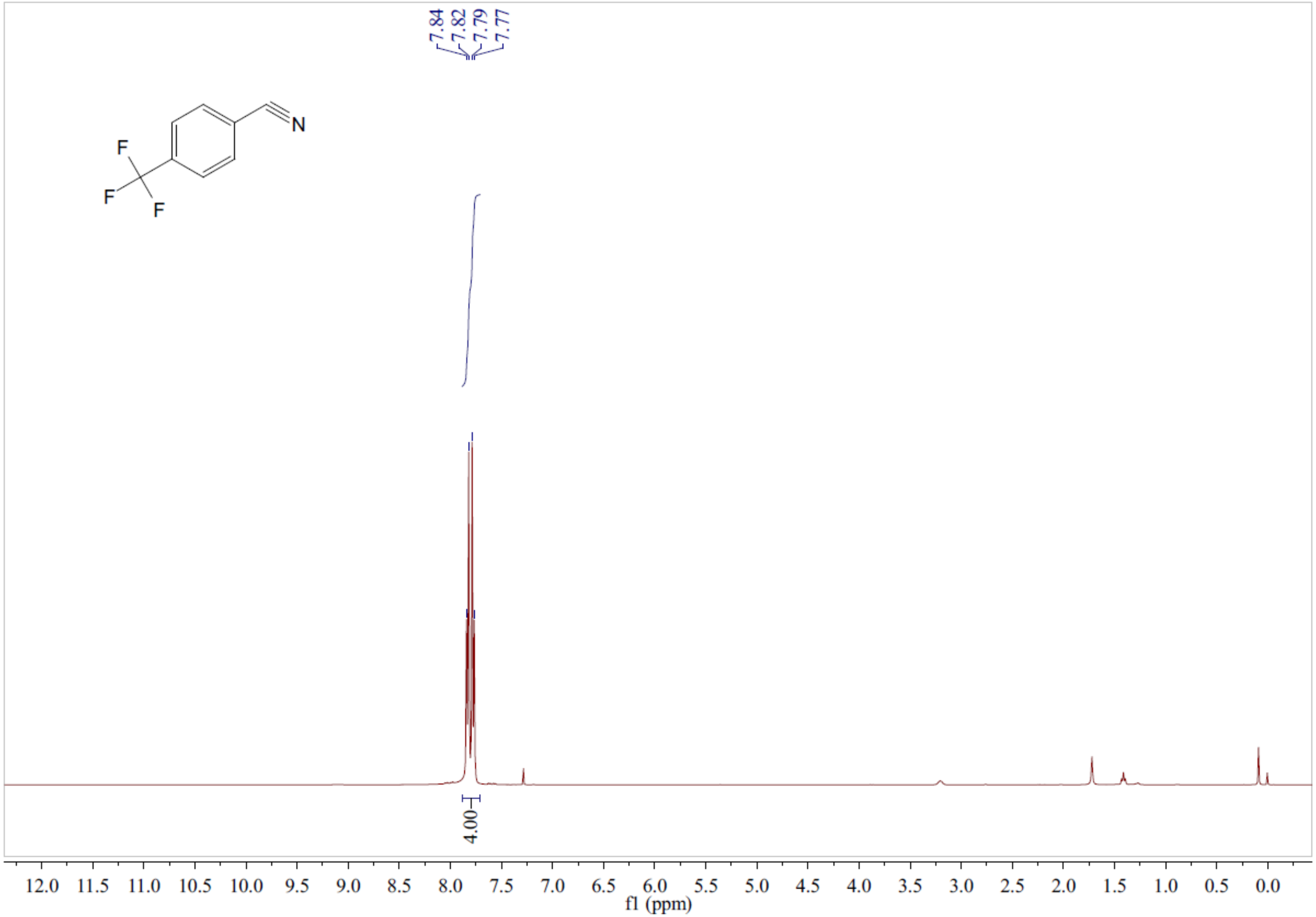

^{13}C NMR Spectra of 2g

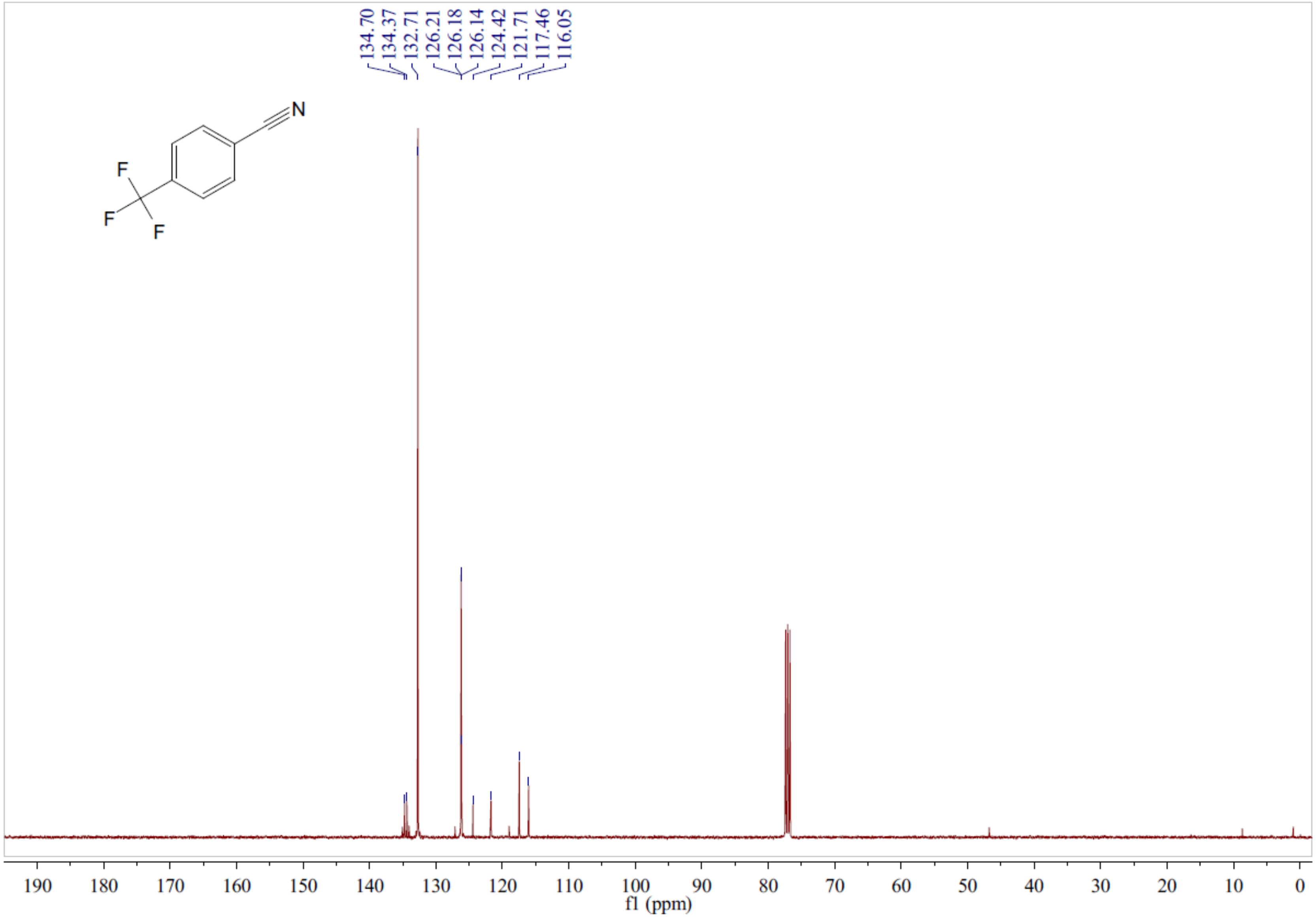

^{1}H NMR Spectra of 2h

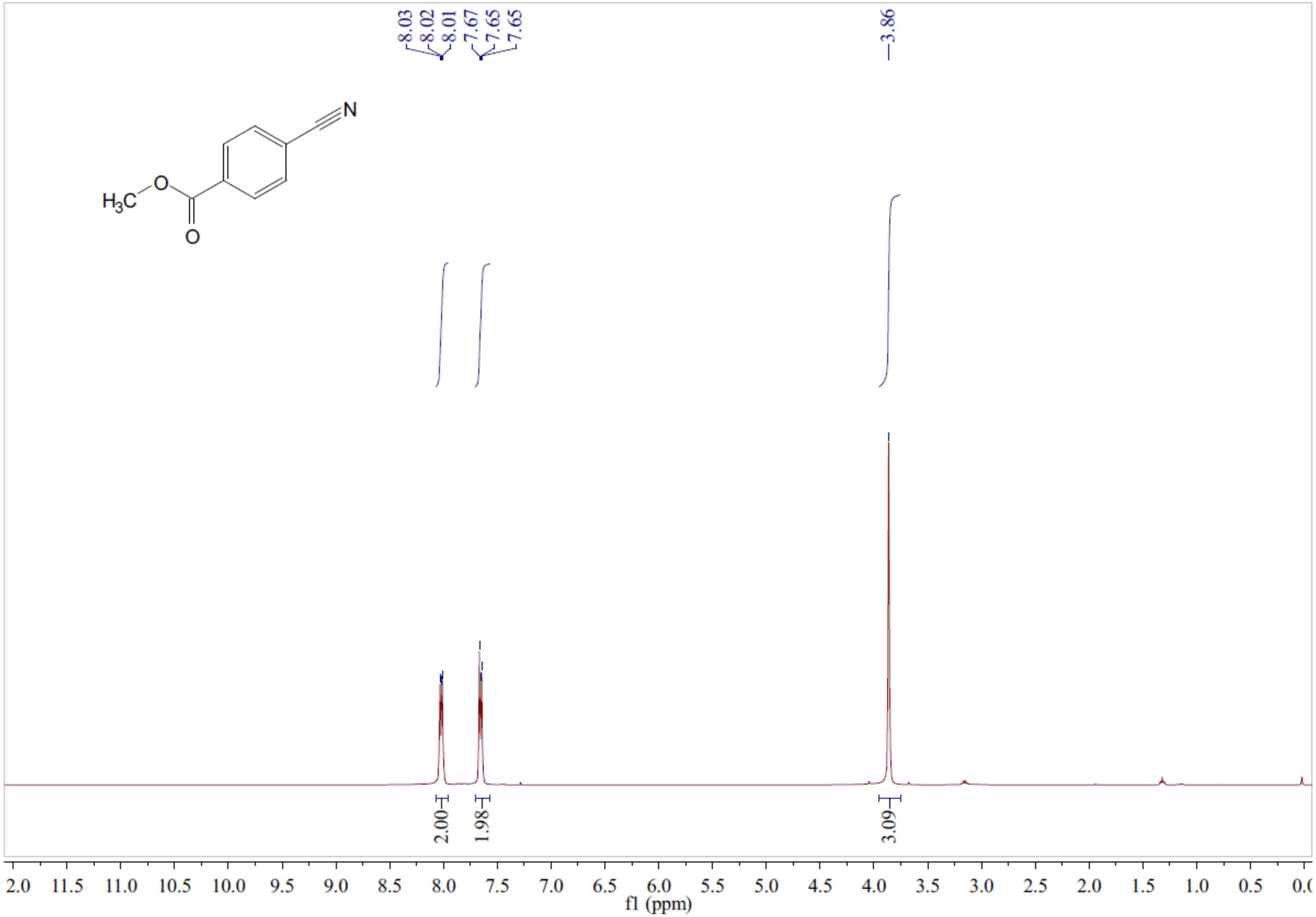

^{13}C NMR Spectra of 2h

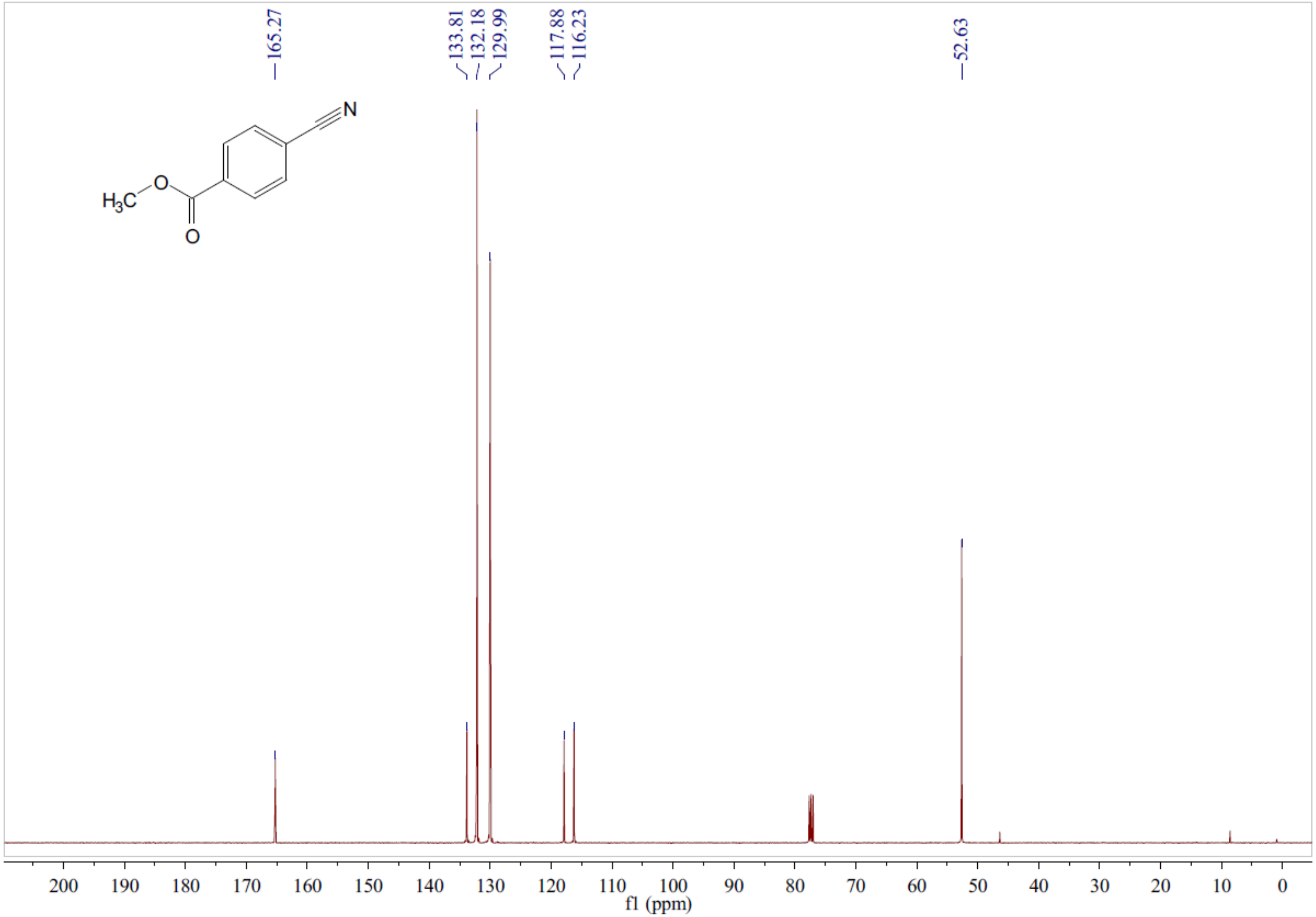

^{1}H NMR Spectra of 2i

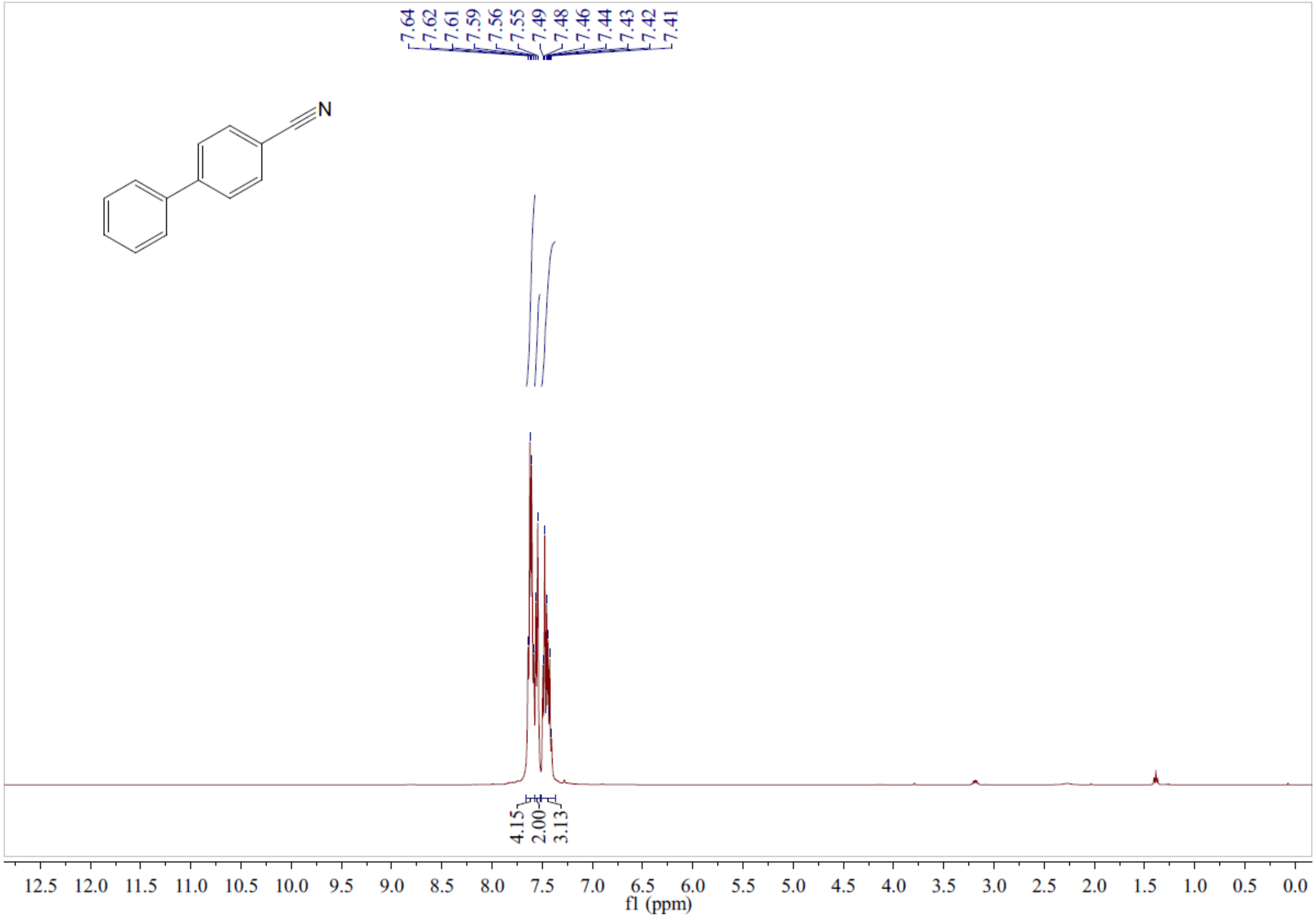

^{13}C NMR Spectra of 2i

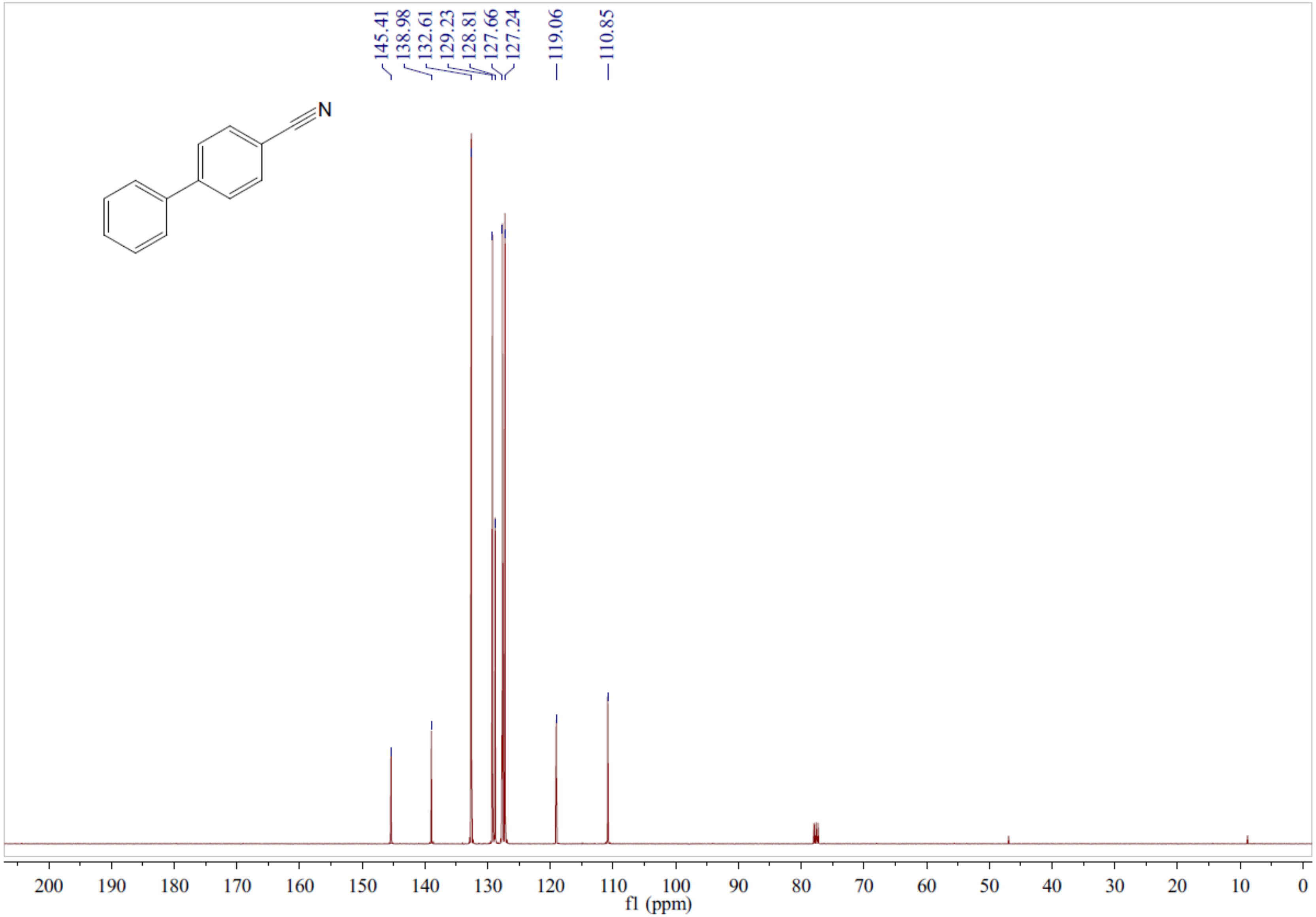

^{1}H NMR Spectra of 2j

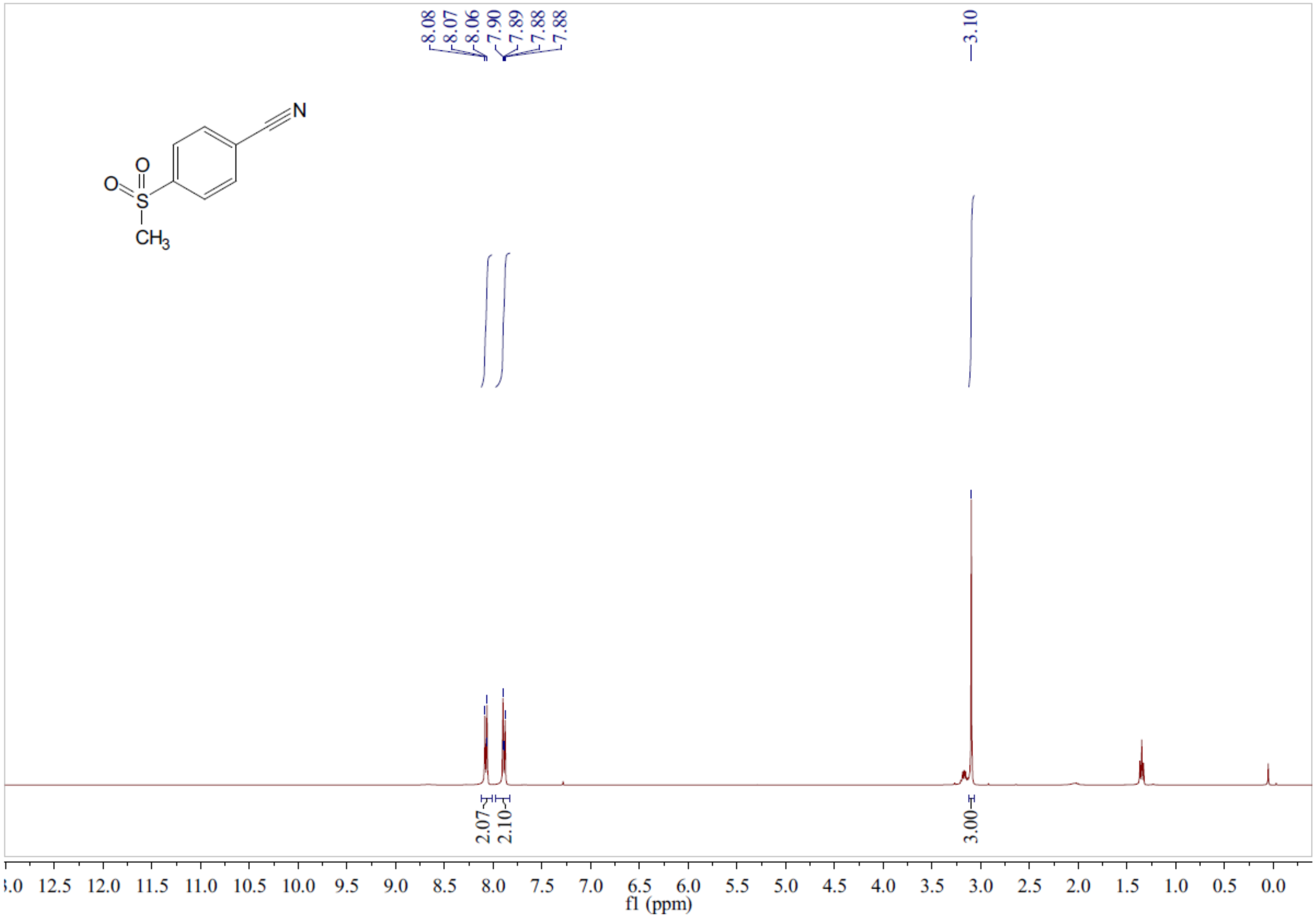

^{13}C NMR Spectra of 2j

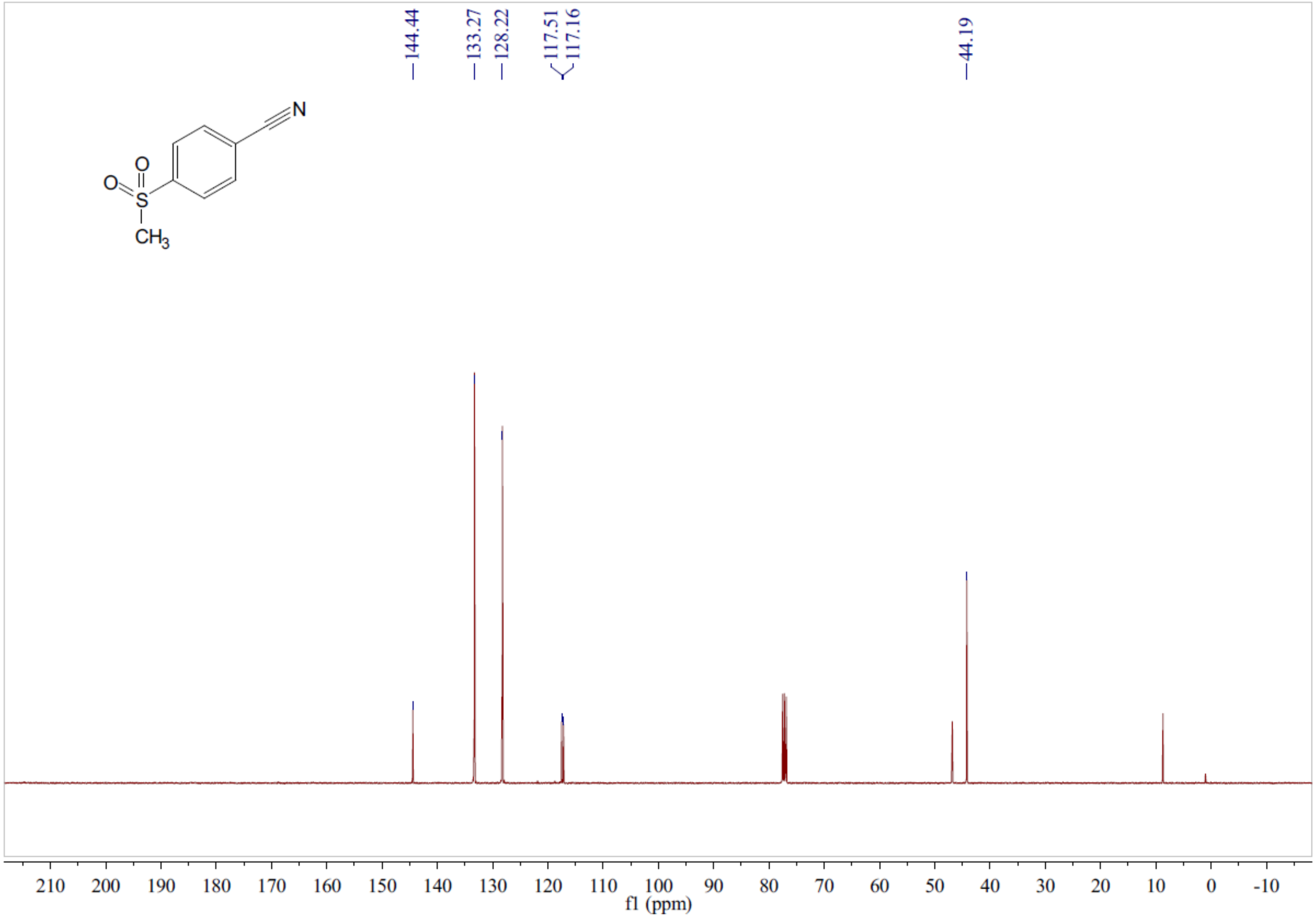

^{1}H NMR Spectra of 2k

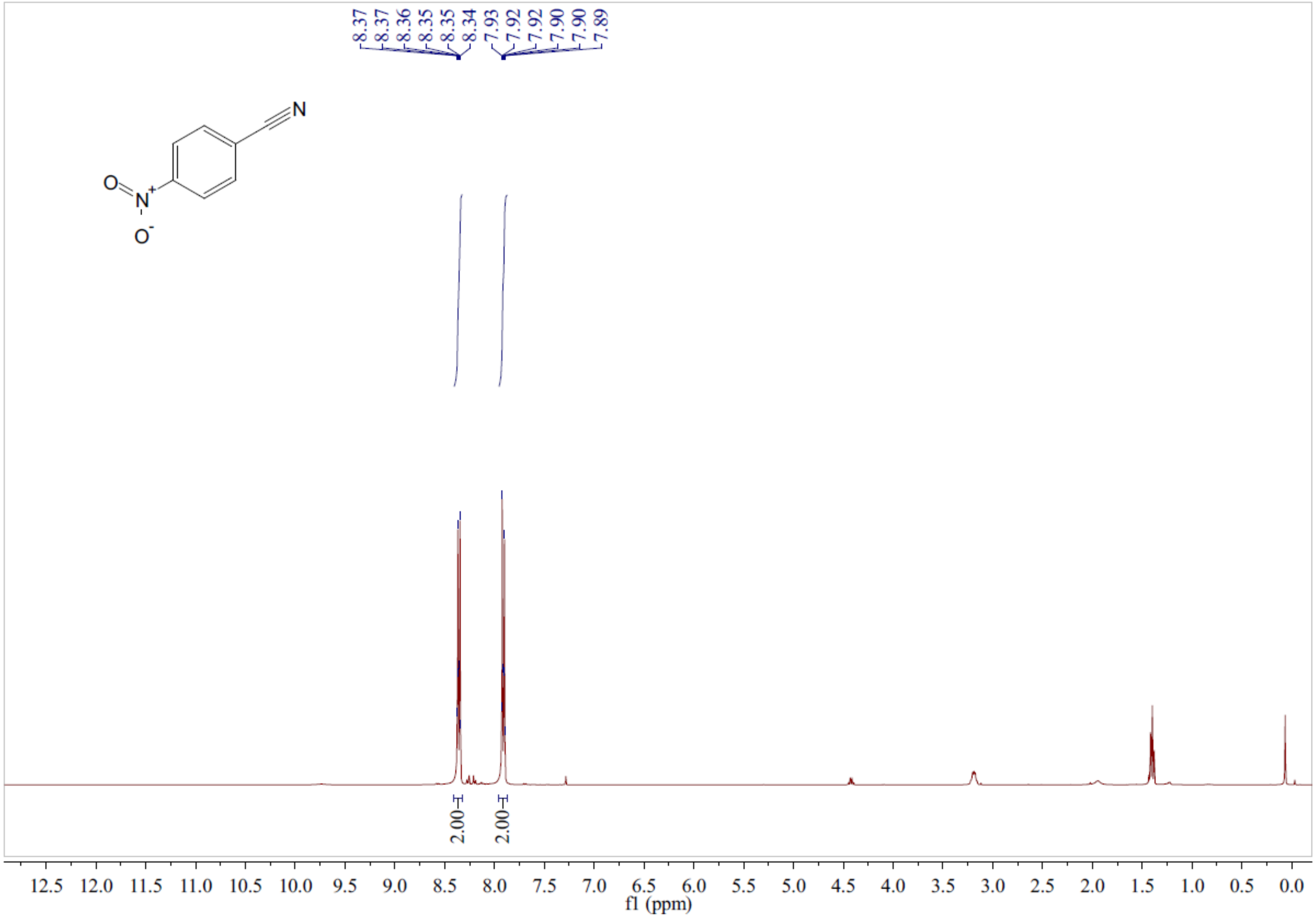

^{13}C NMR Spectra of 2k

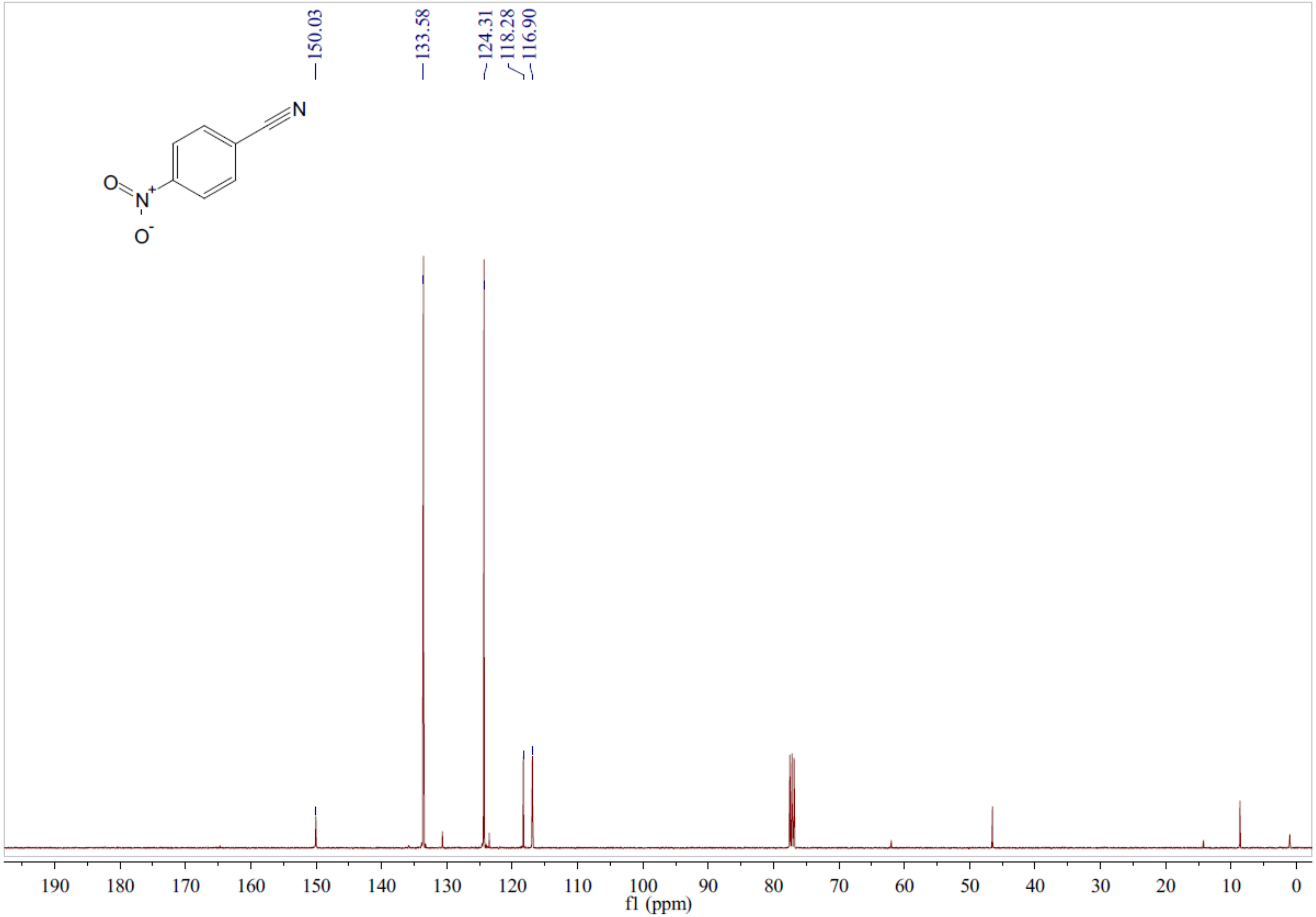

^{1}H NMR Spectra of 2l

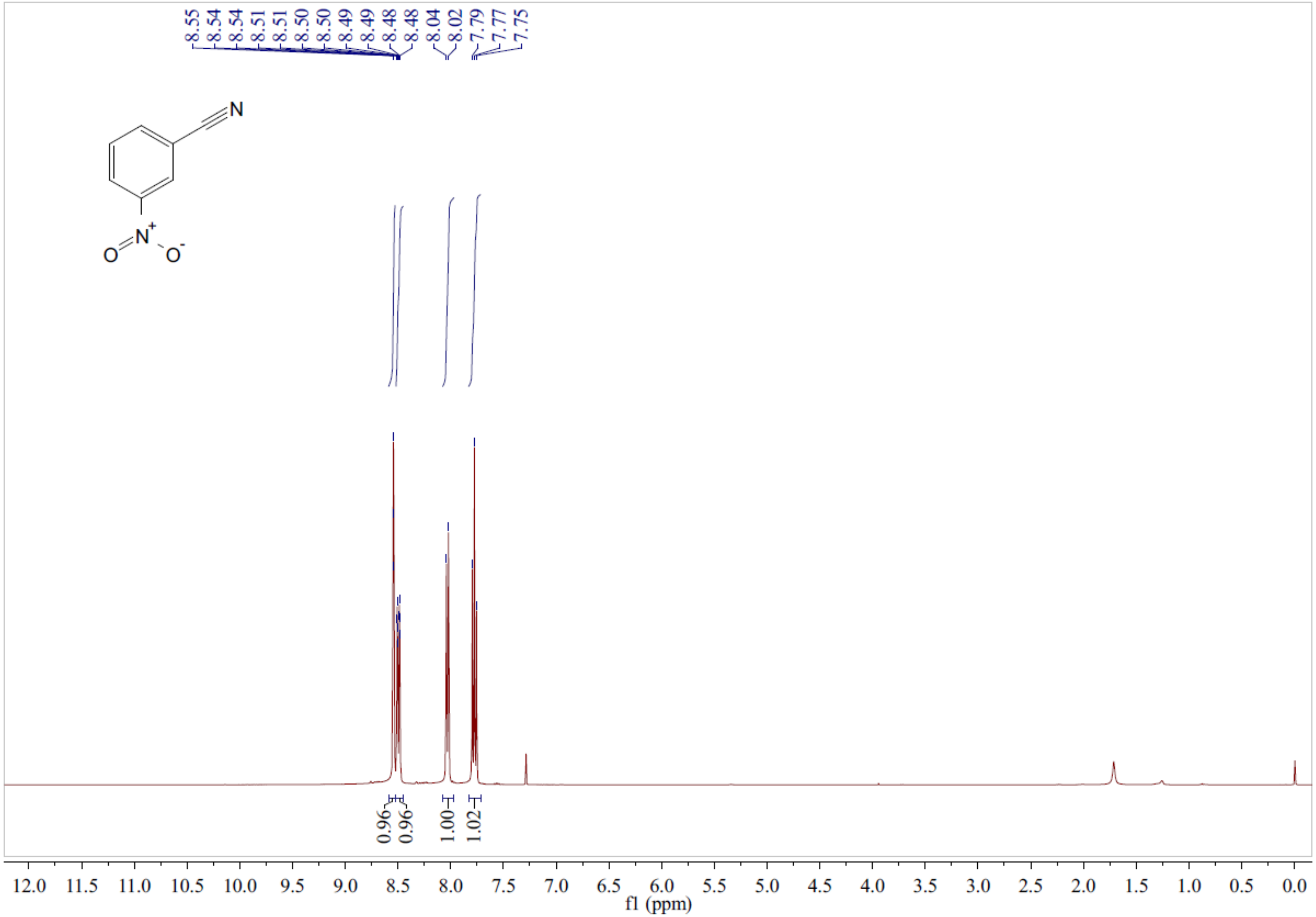

^{13}C NMR Spectra of 2l

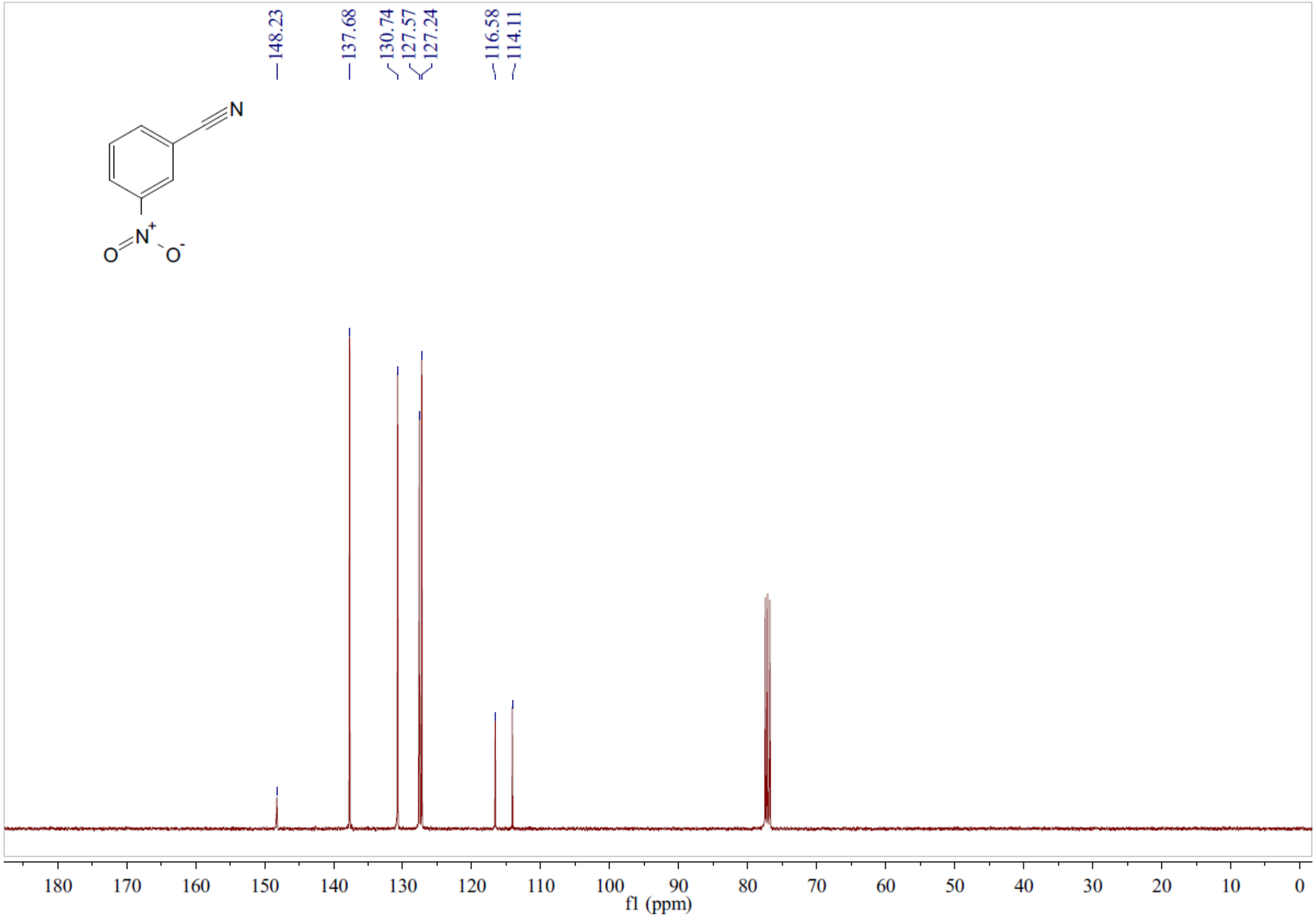

^{1}H NMR Spectra of 2m

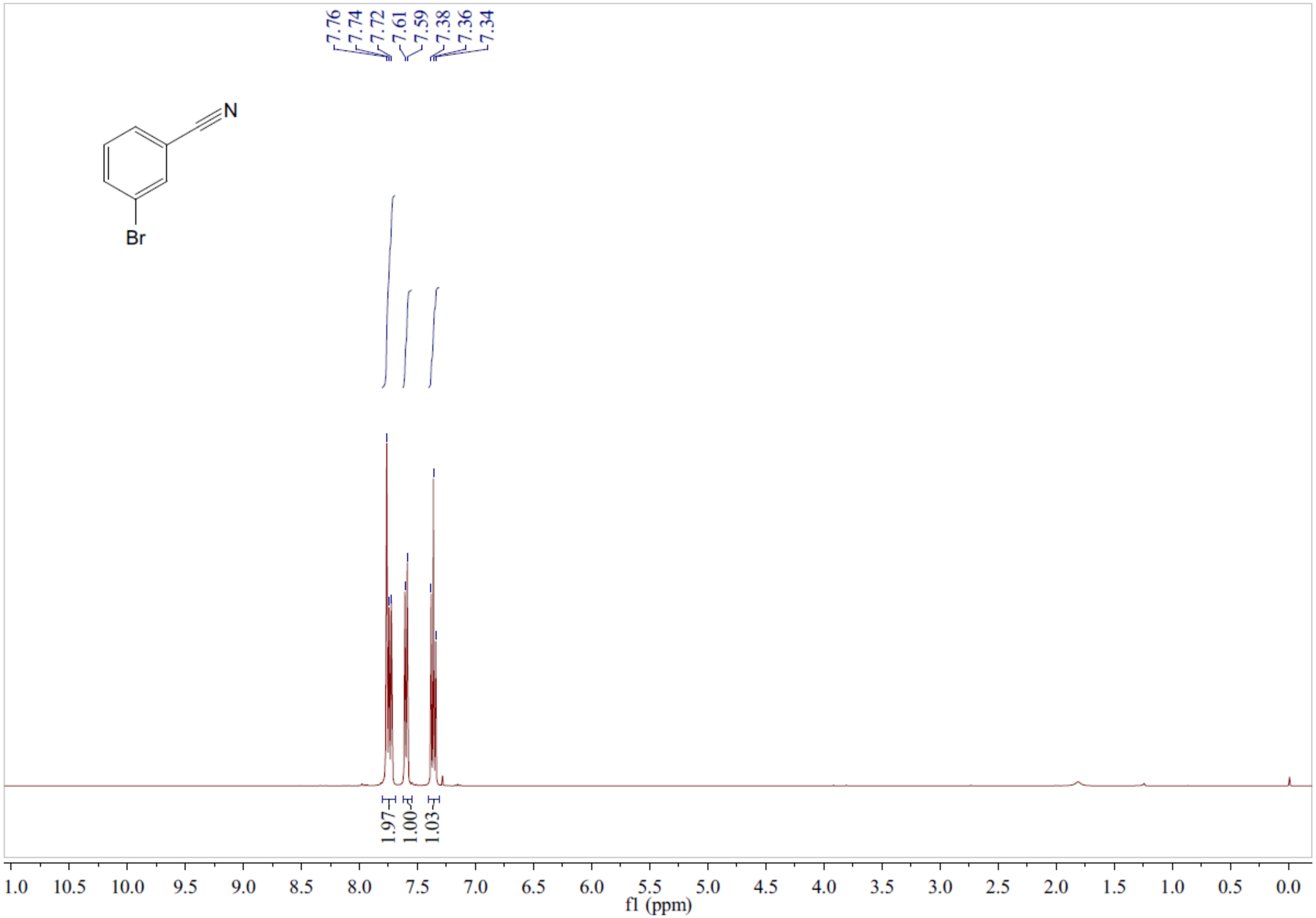

^{13}C NMR Spectra of 2m

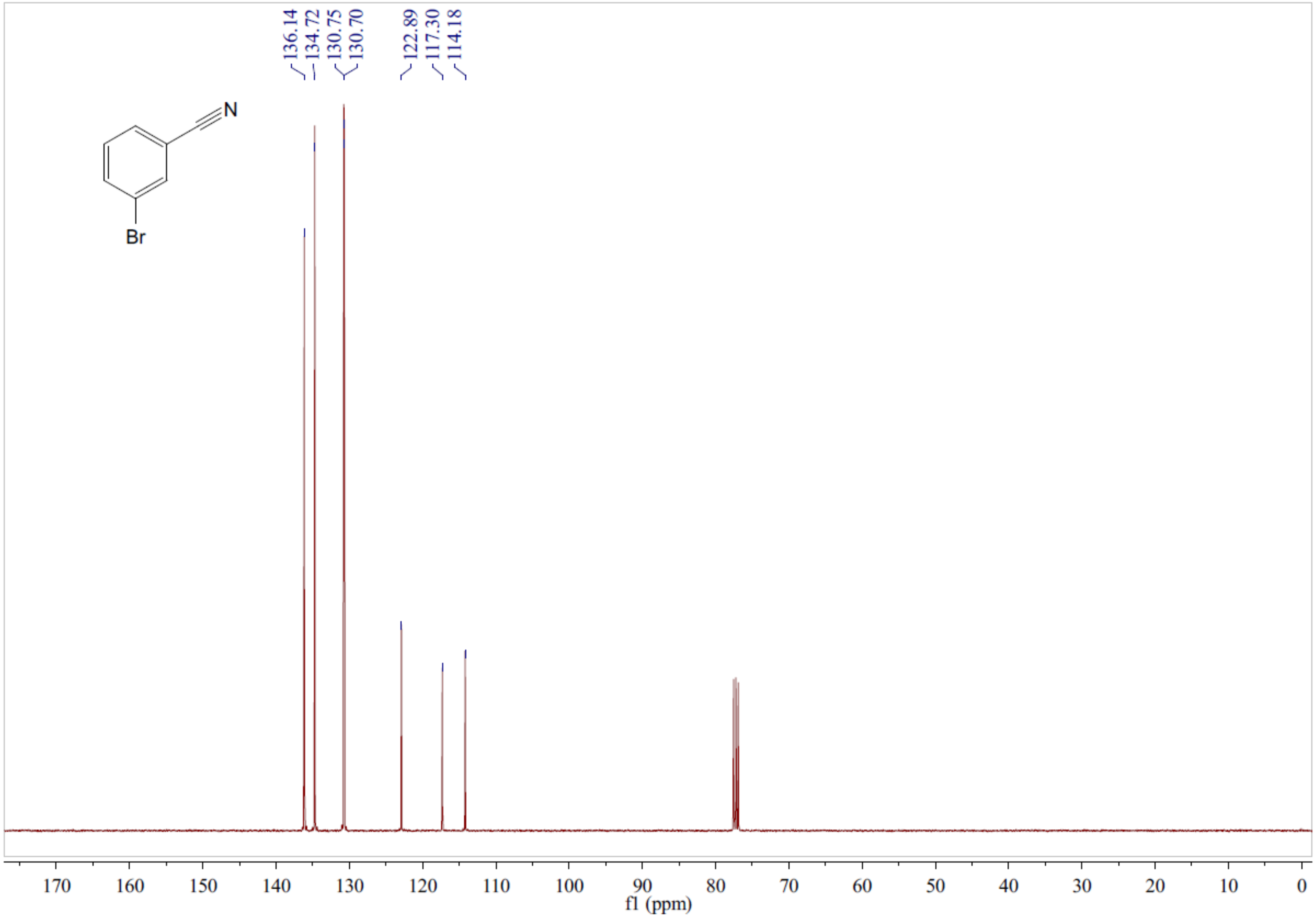

^{1}H NMR Spectra of 2n

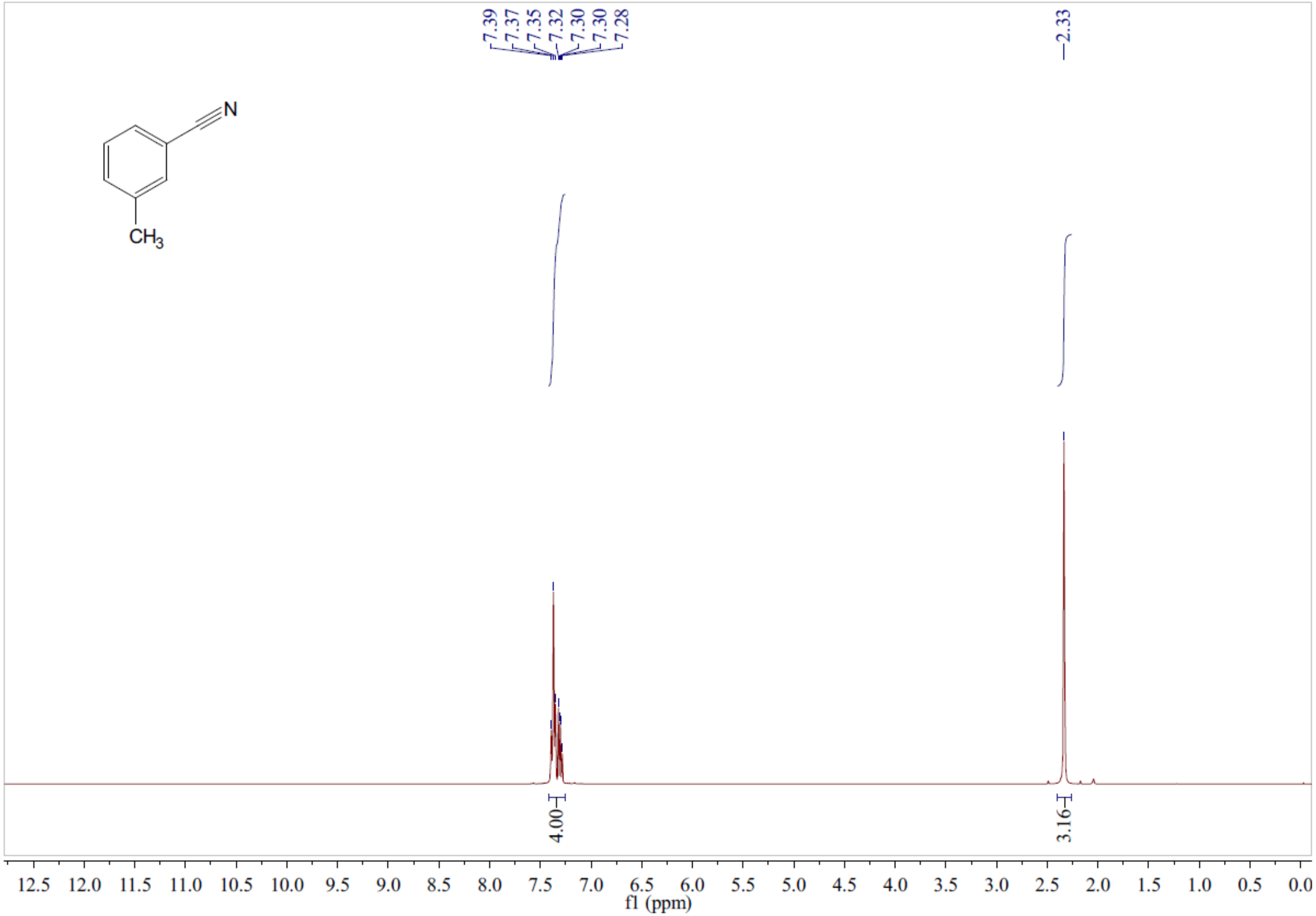

^{13}C NMR Spectra of 2n

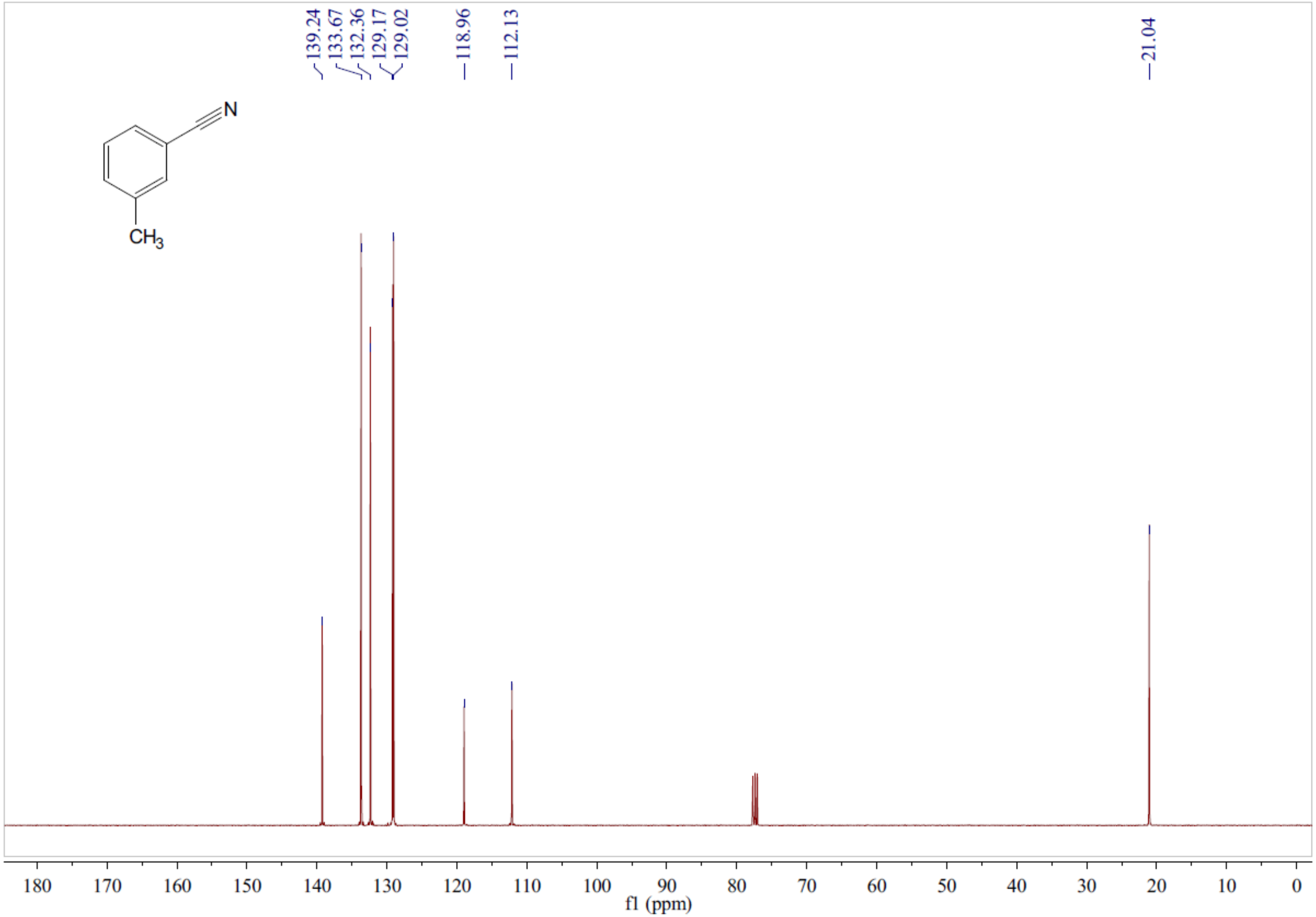

^{1}H NMR Spectra of 2o

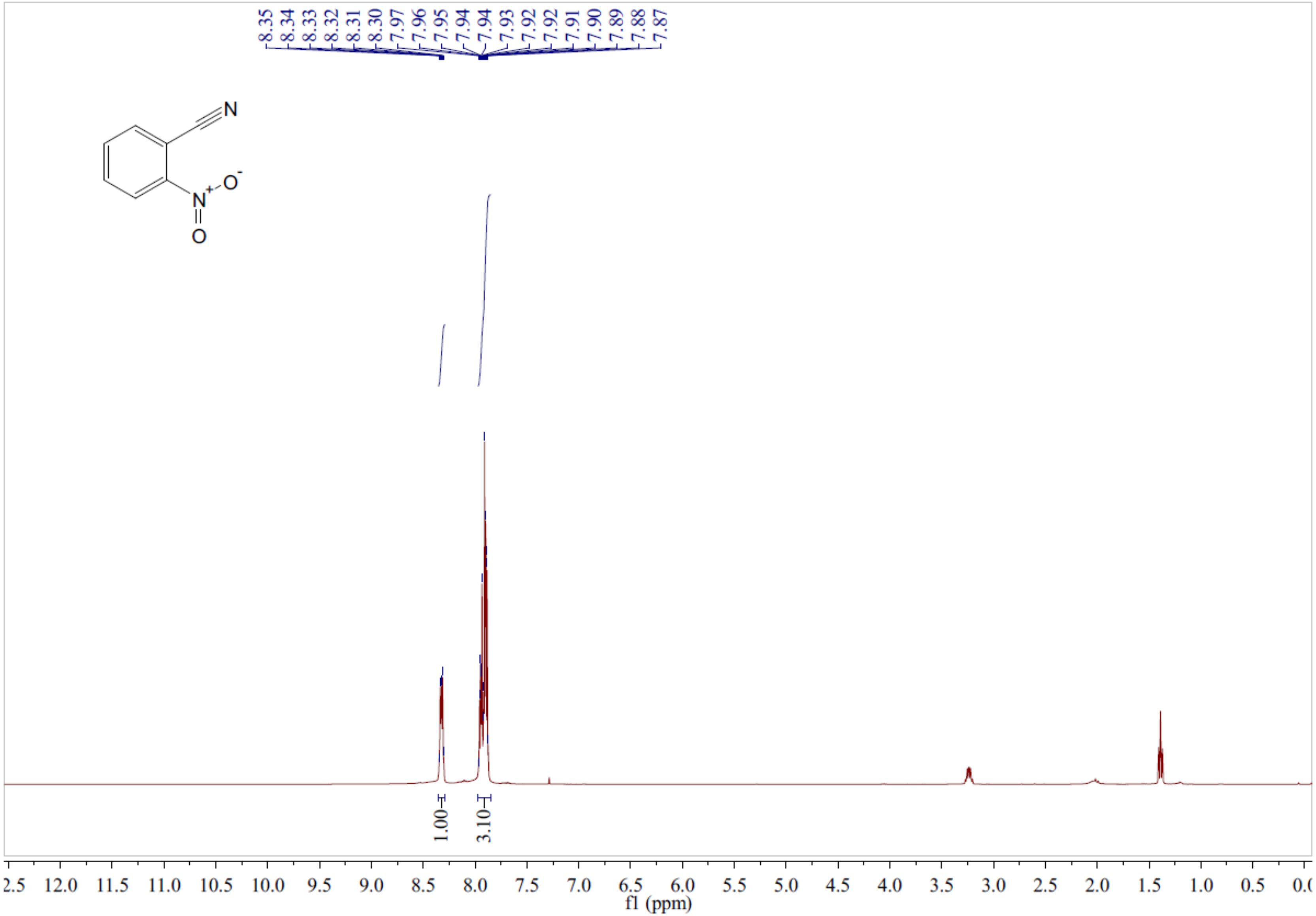

^{13}C NMR Spectra of 2o

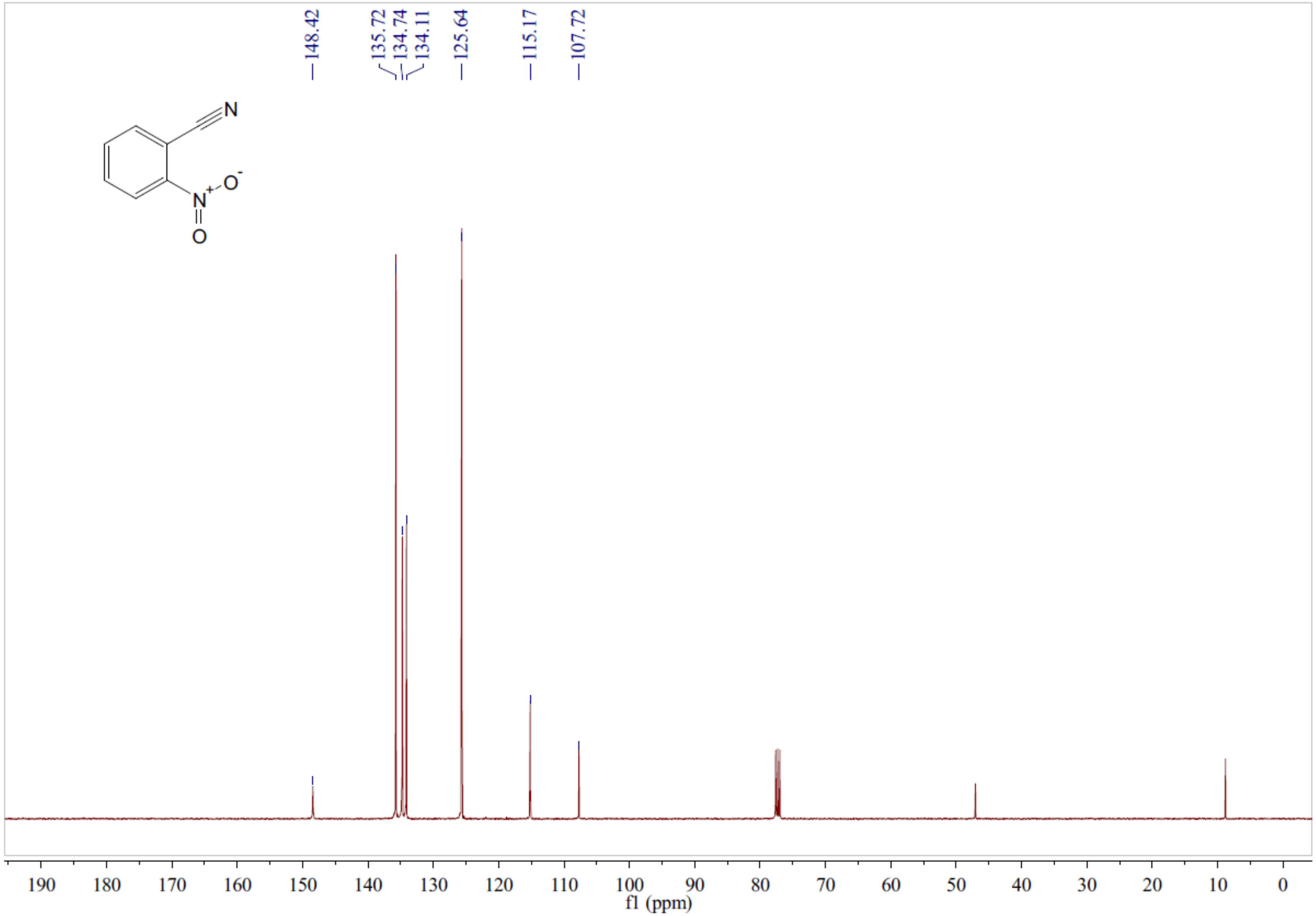

^{1}H NMR Spectra of 2p

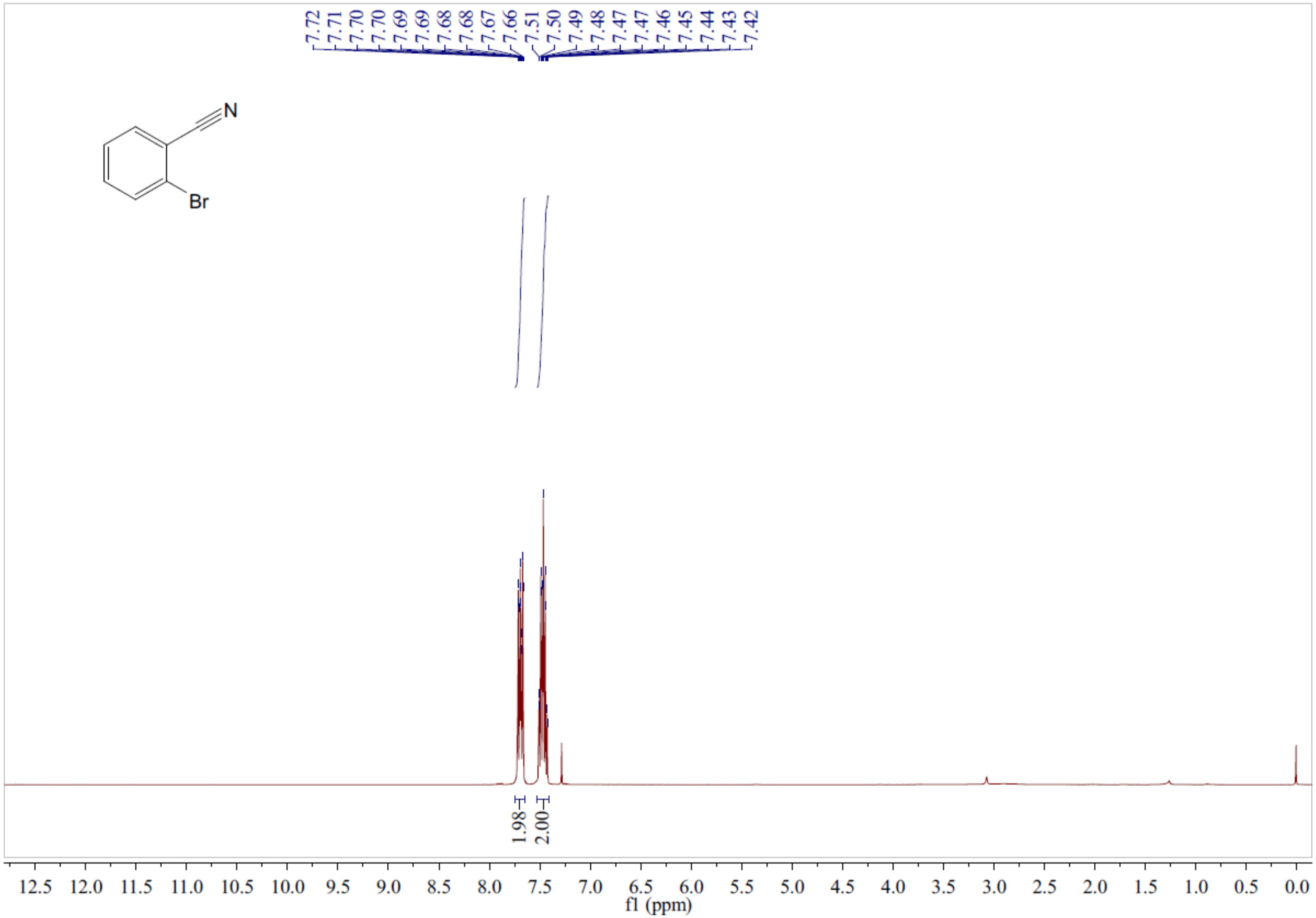

^{13}C NMR Spectra of 2p

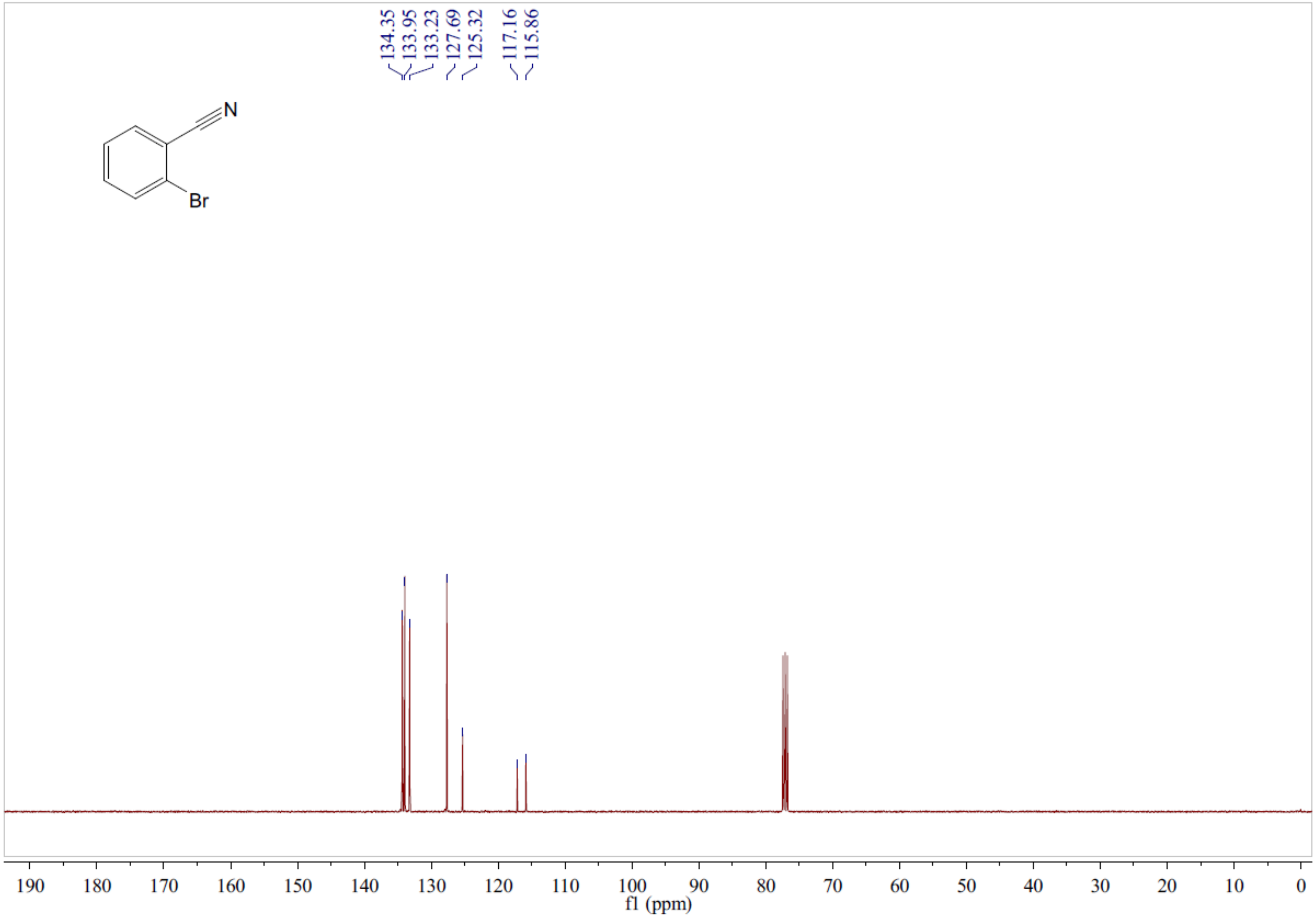

^{1}H NMR Spectra of 2q

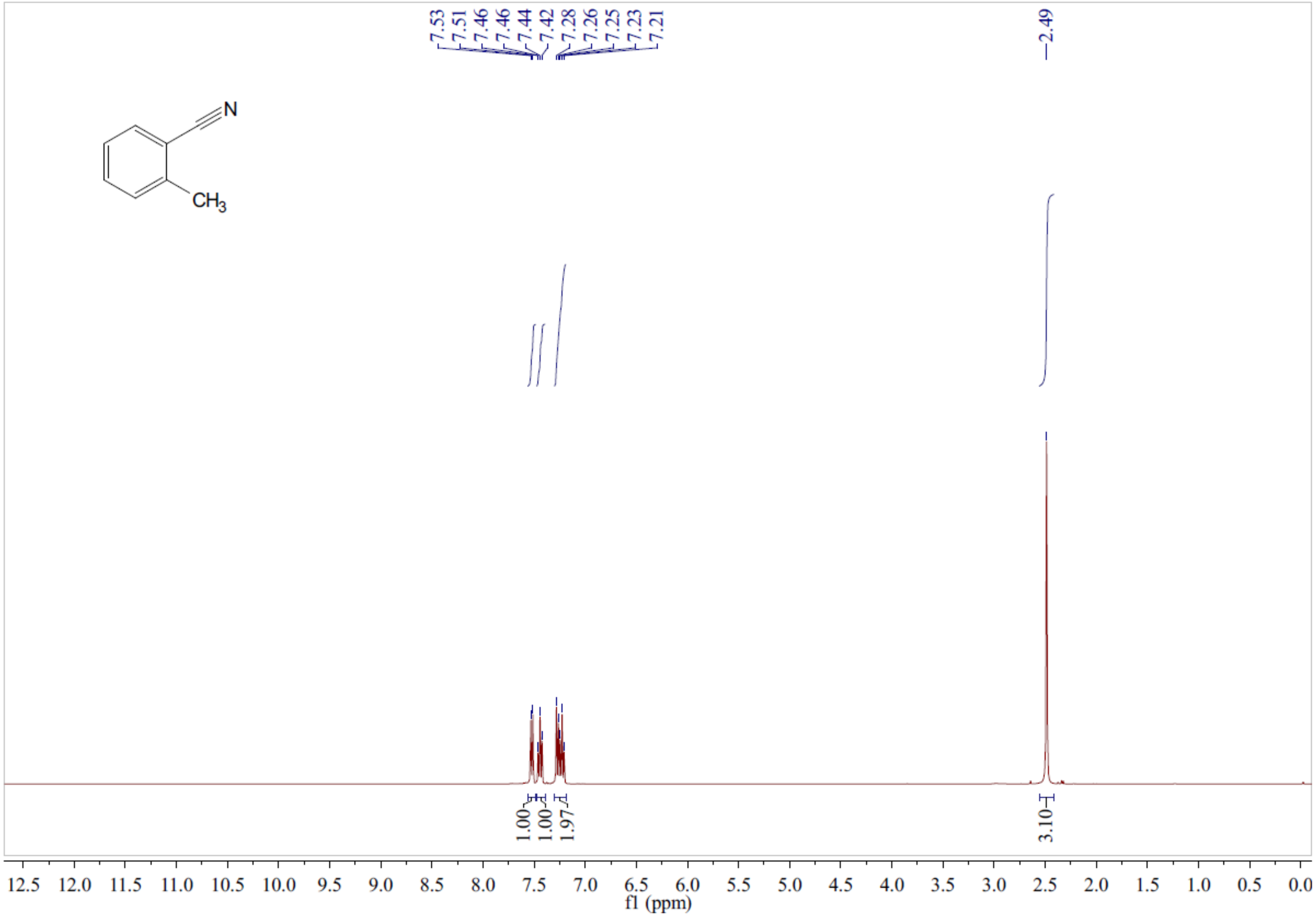

^{13}C NMR Spectra of 2q

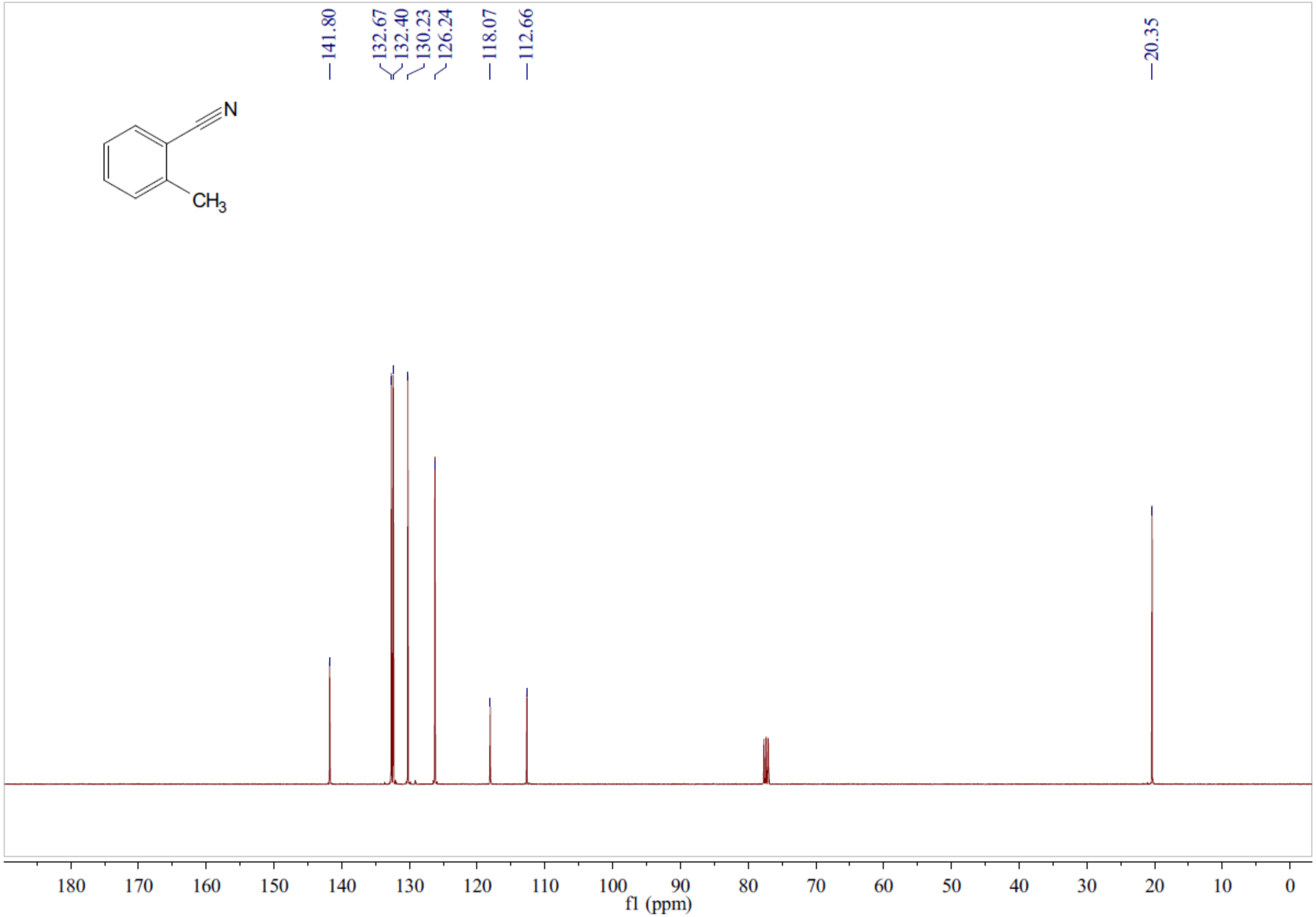

^{1}H NMR Spectra of 2r

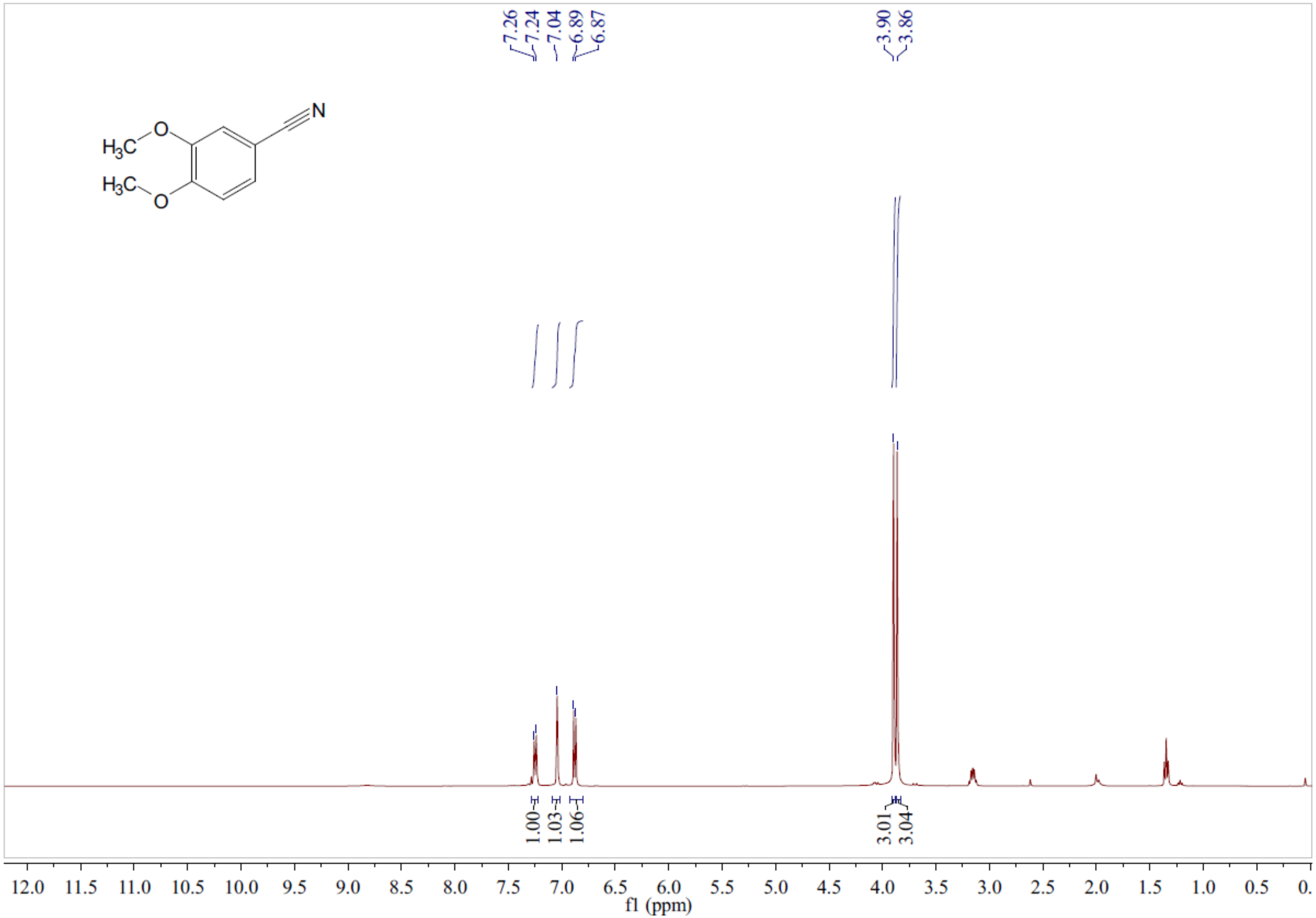

^{13}C NMR Spectra of 2r

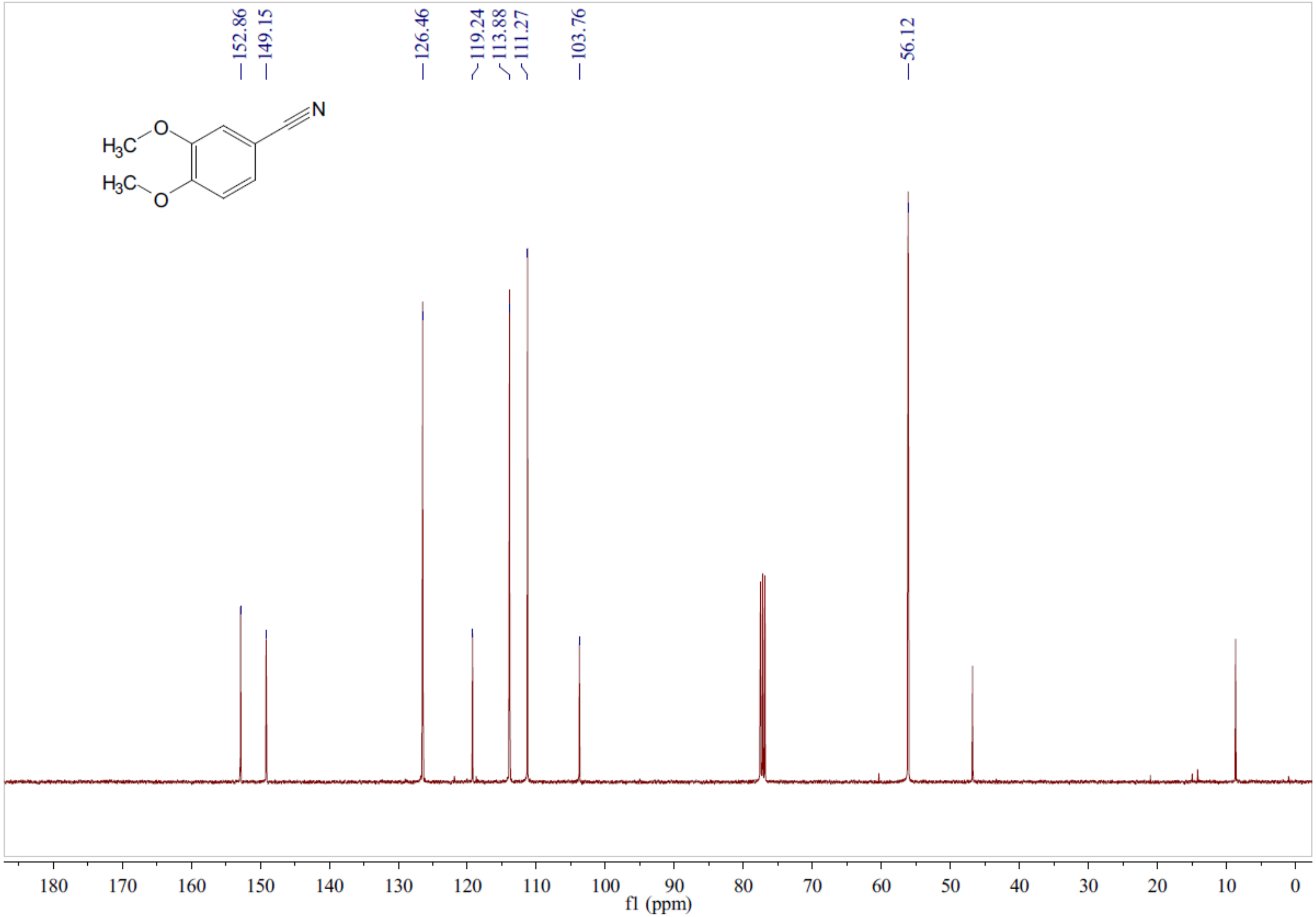

^{1}H NMR Spectra of 2s

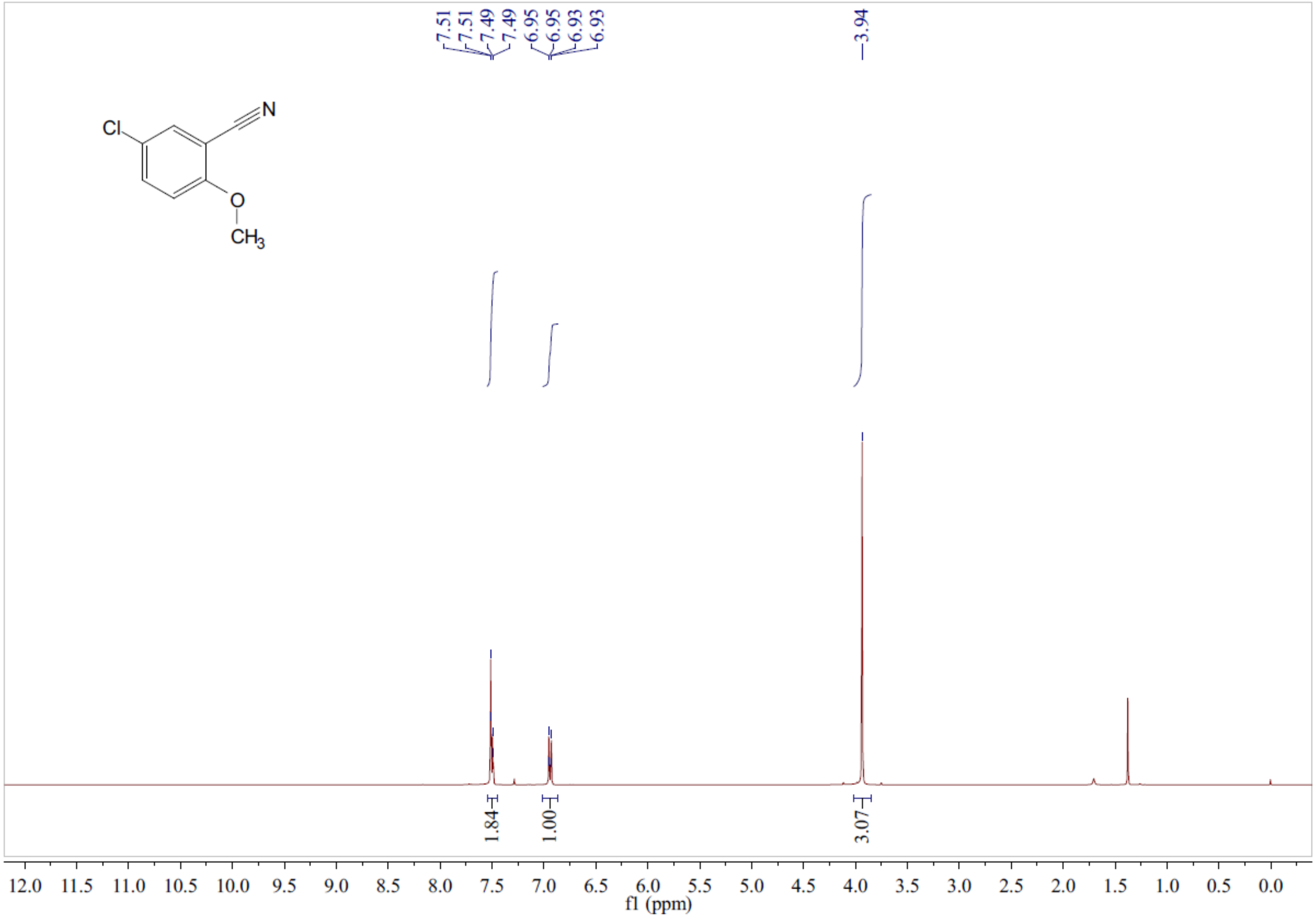

^{13}C NMR Spectra of 2s

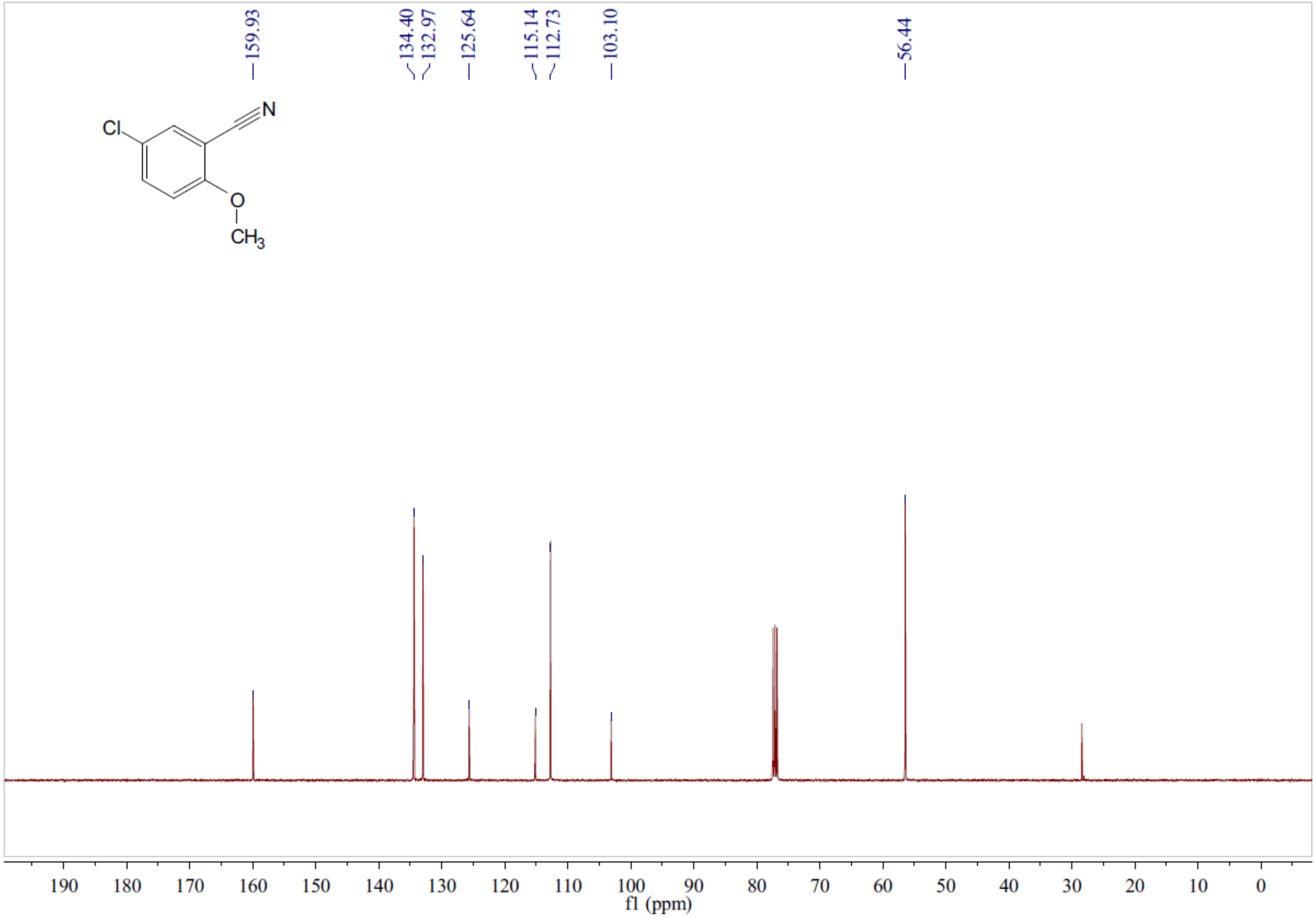

^{1}H NMR Spectra of 2t

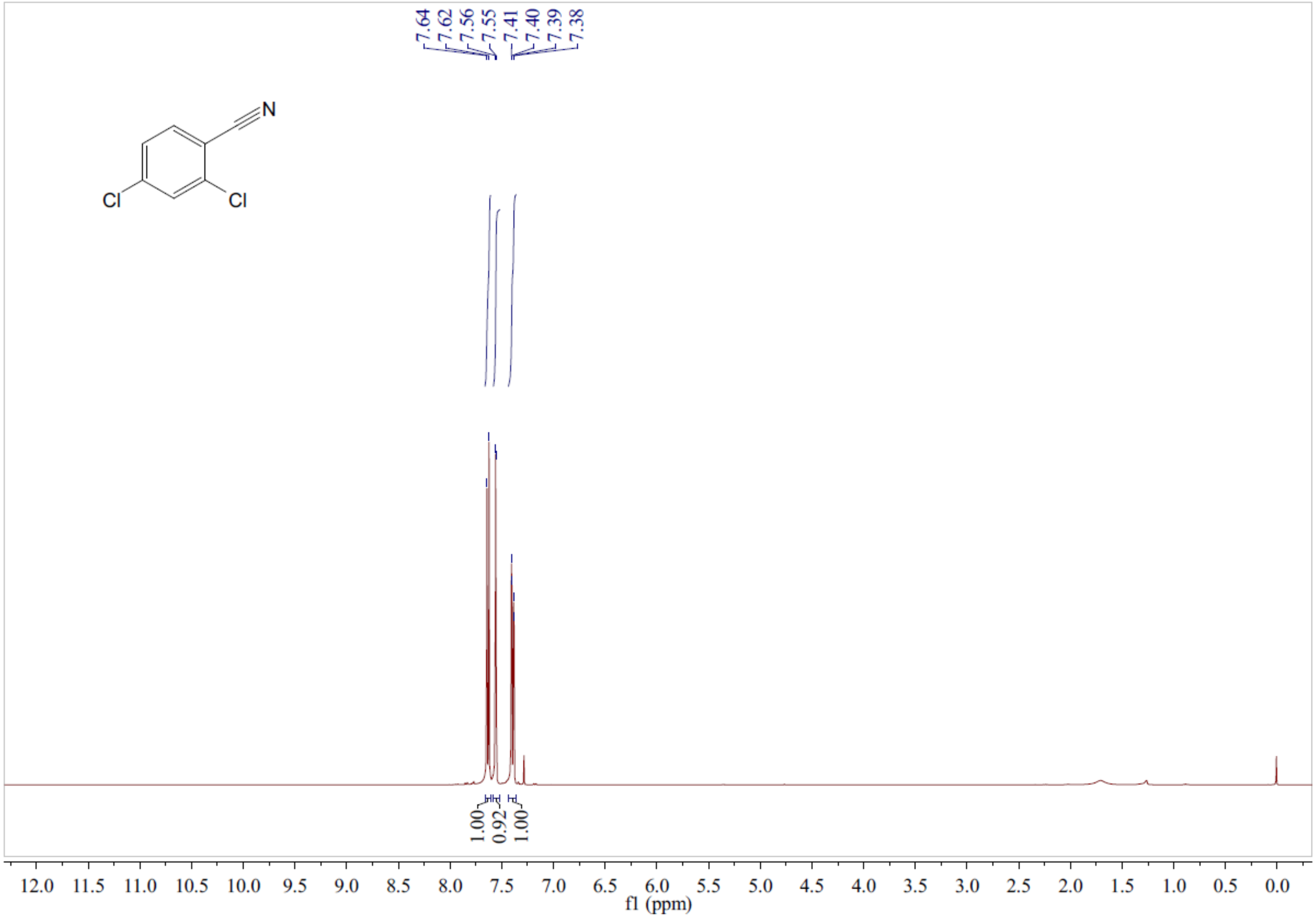

^{13}C NMR Spectra of 2t

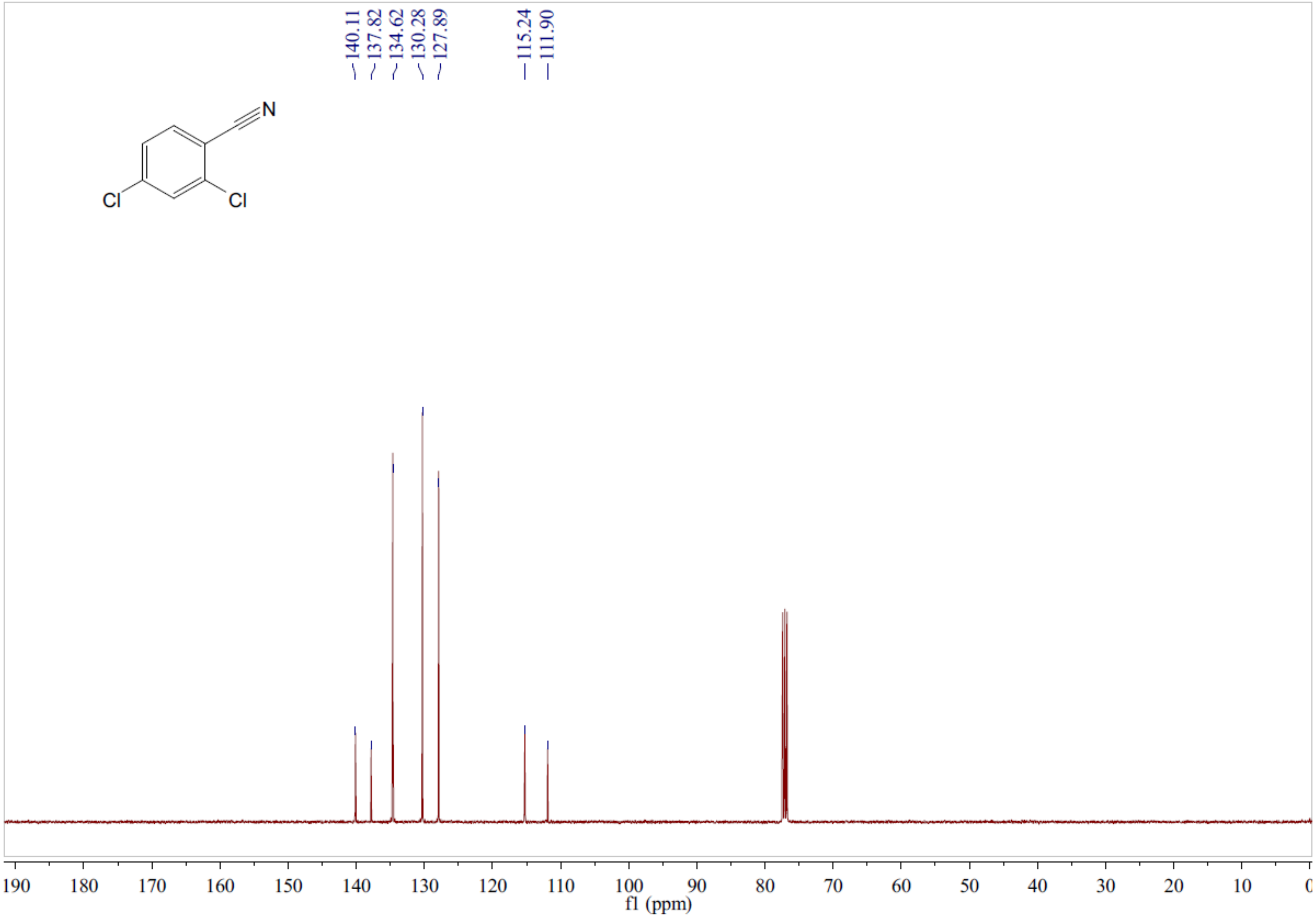

^{1}H NMR Spectra of 2u

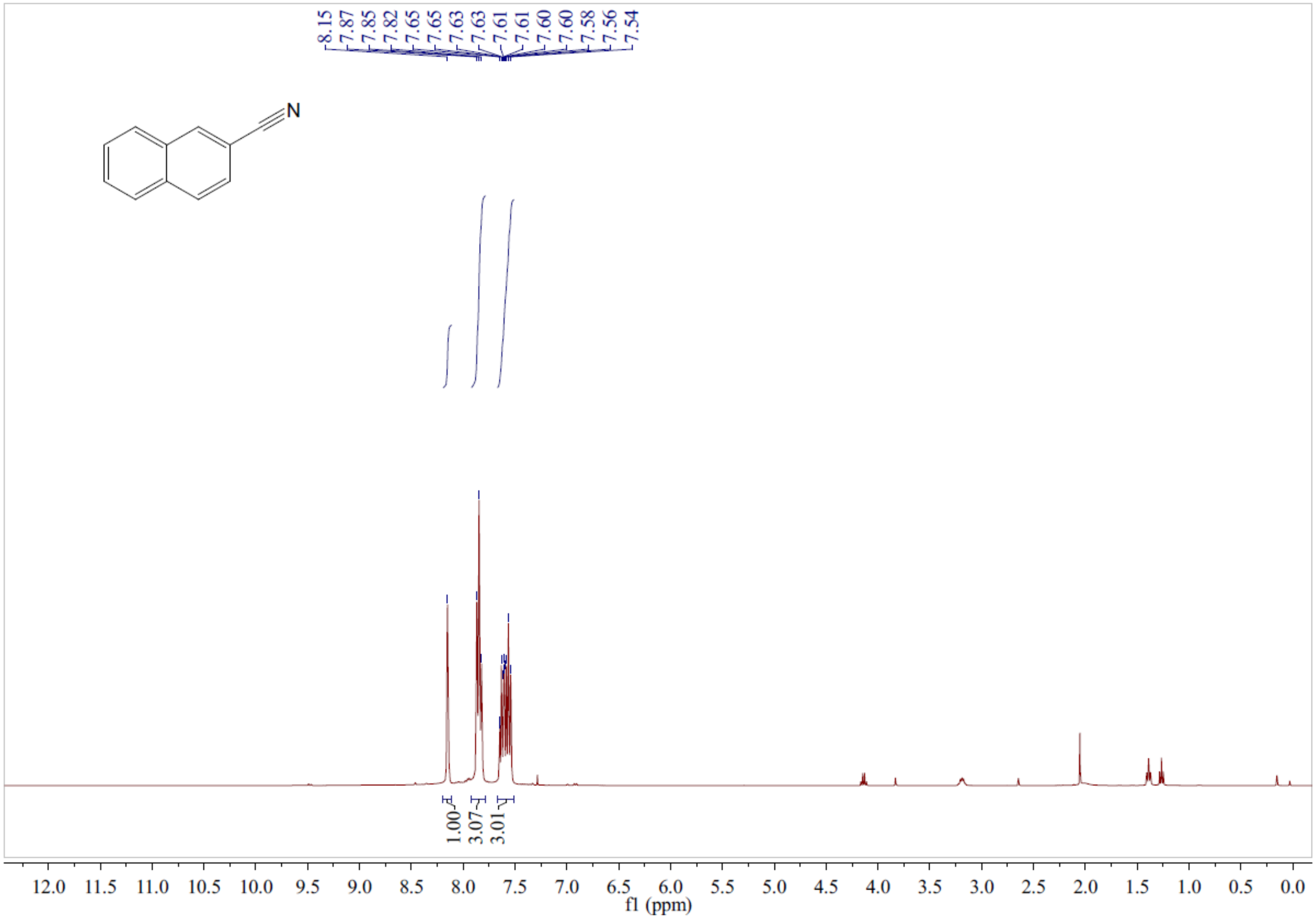

^{13}C NMR Spectra of 2u

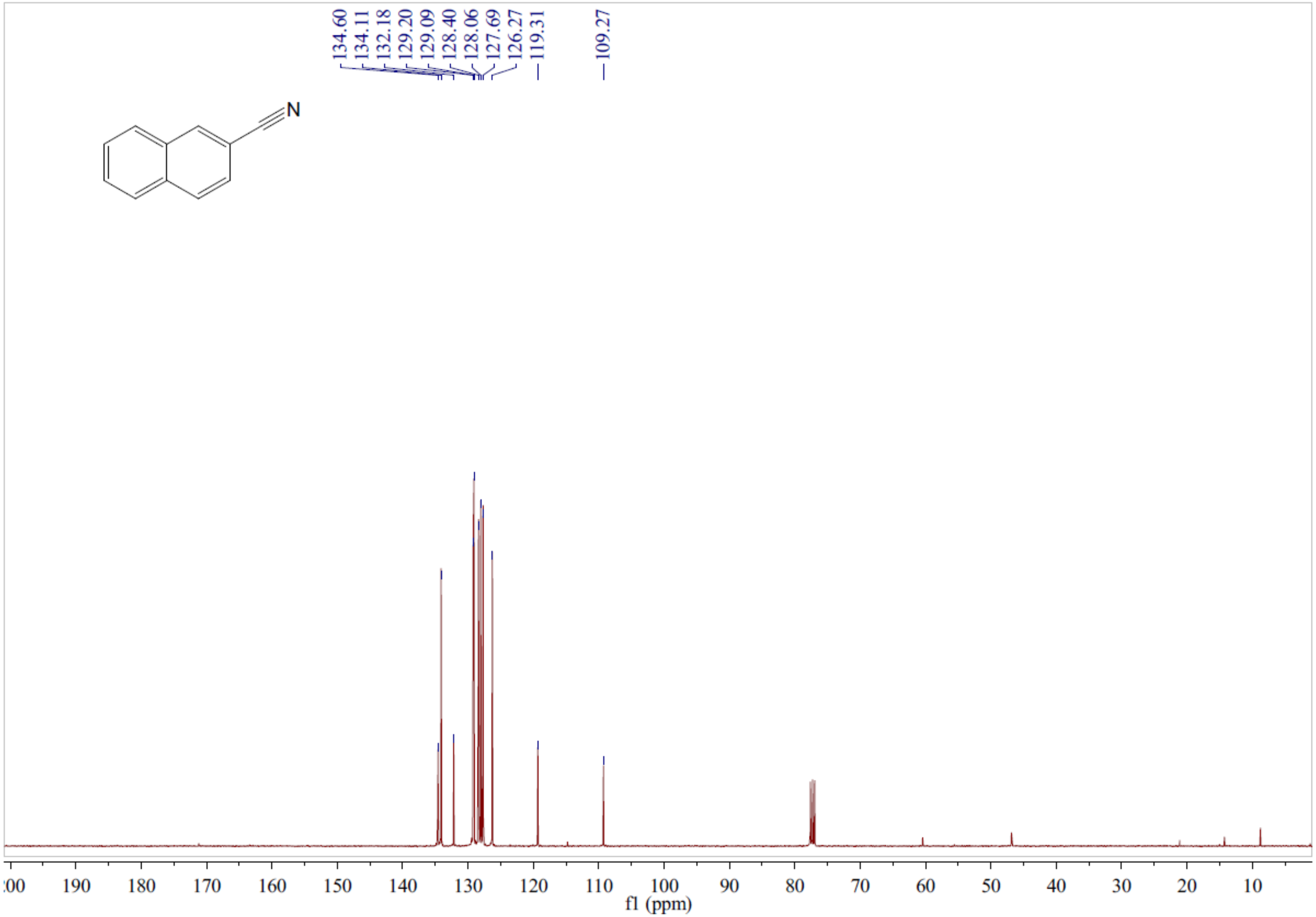

^{1}H NMR Spectra of 2v

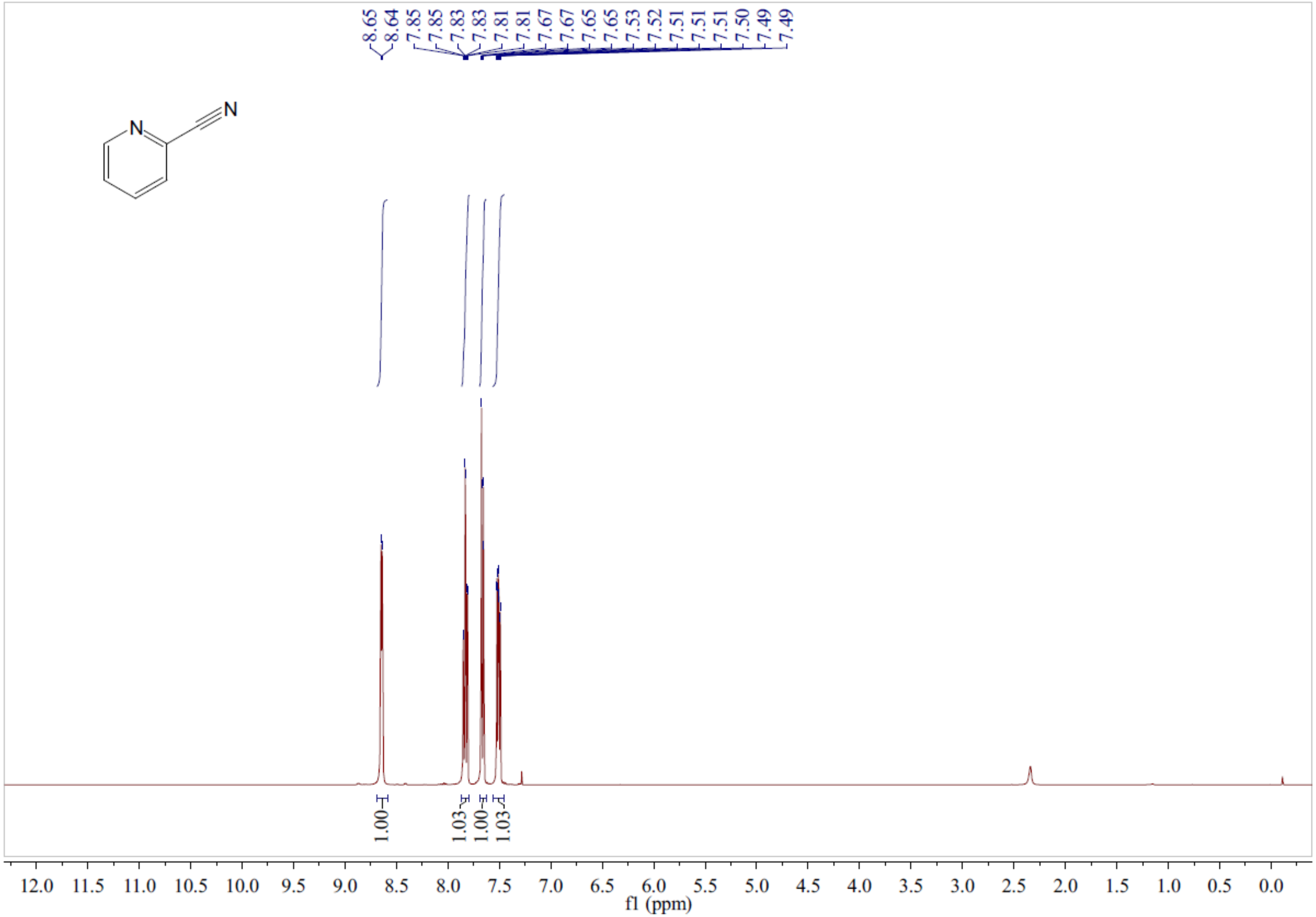

^{13}C NMR Spectra of 2v

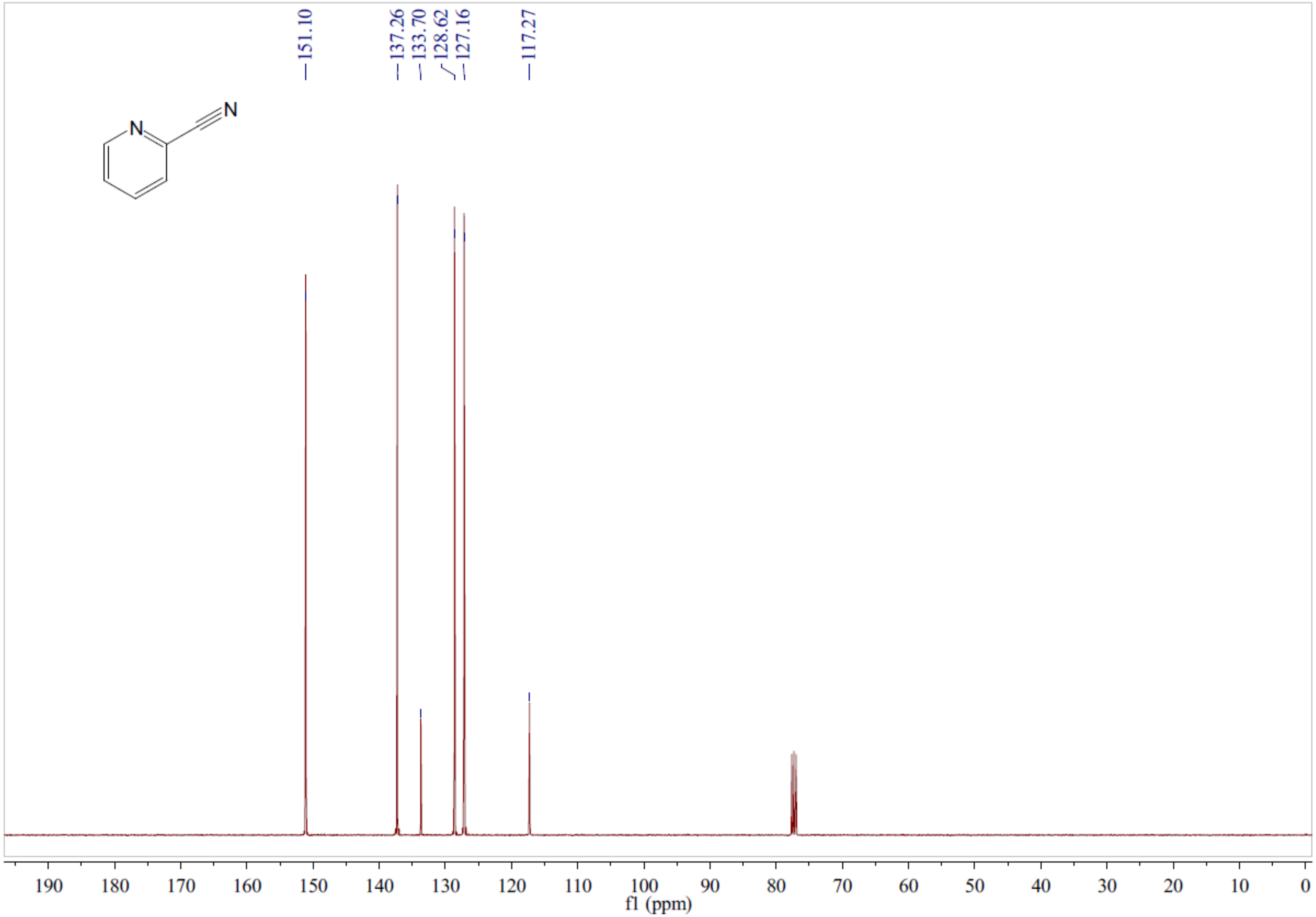

^{1}H NMR Spectra of 2w

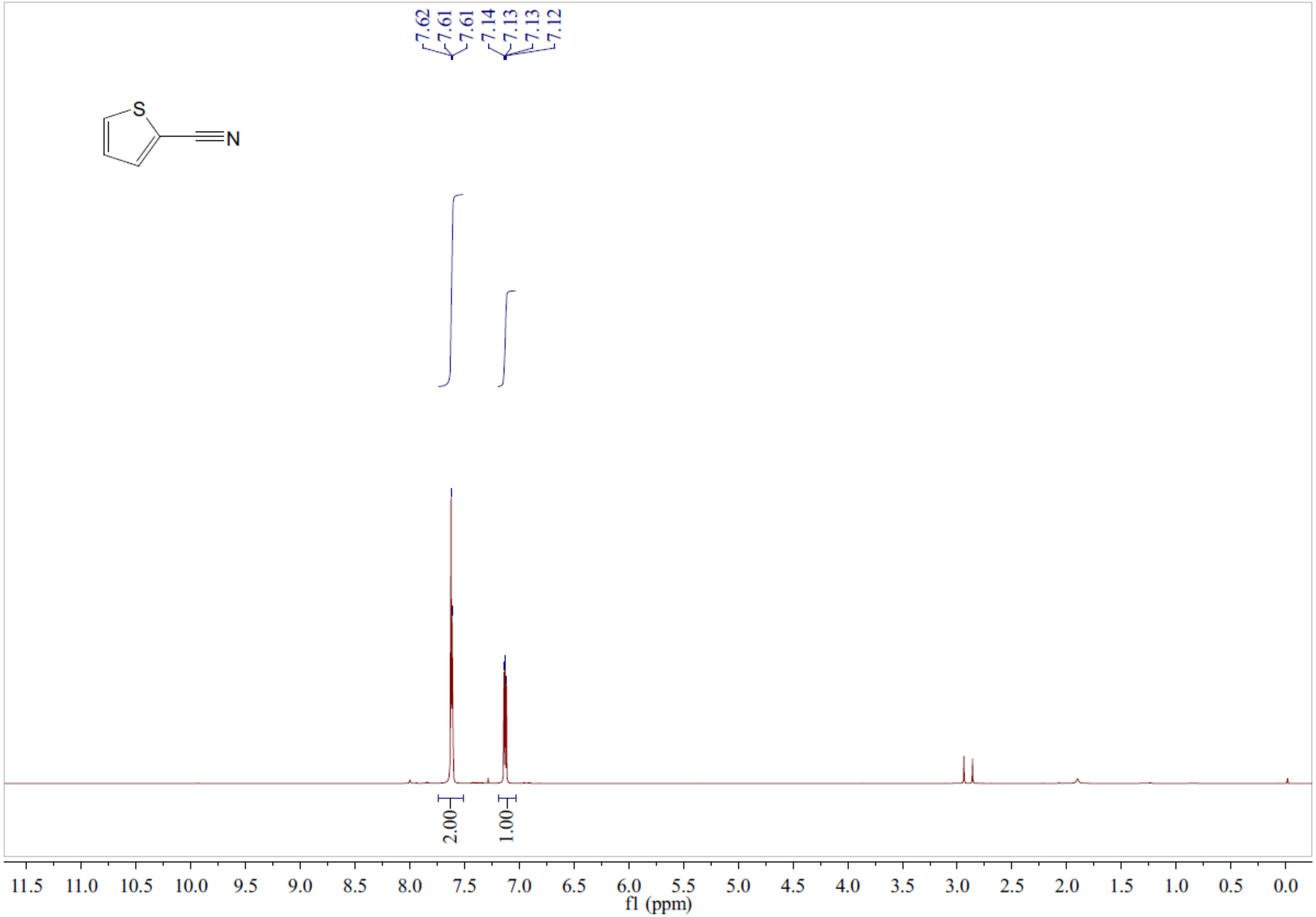

^{13}C NMR Spectra of 2w

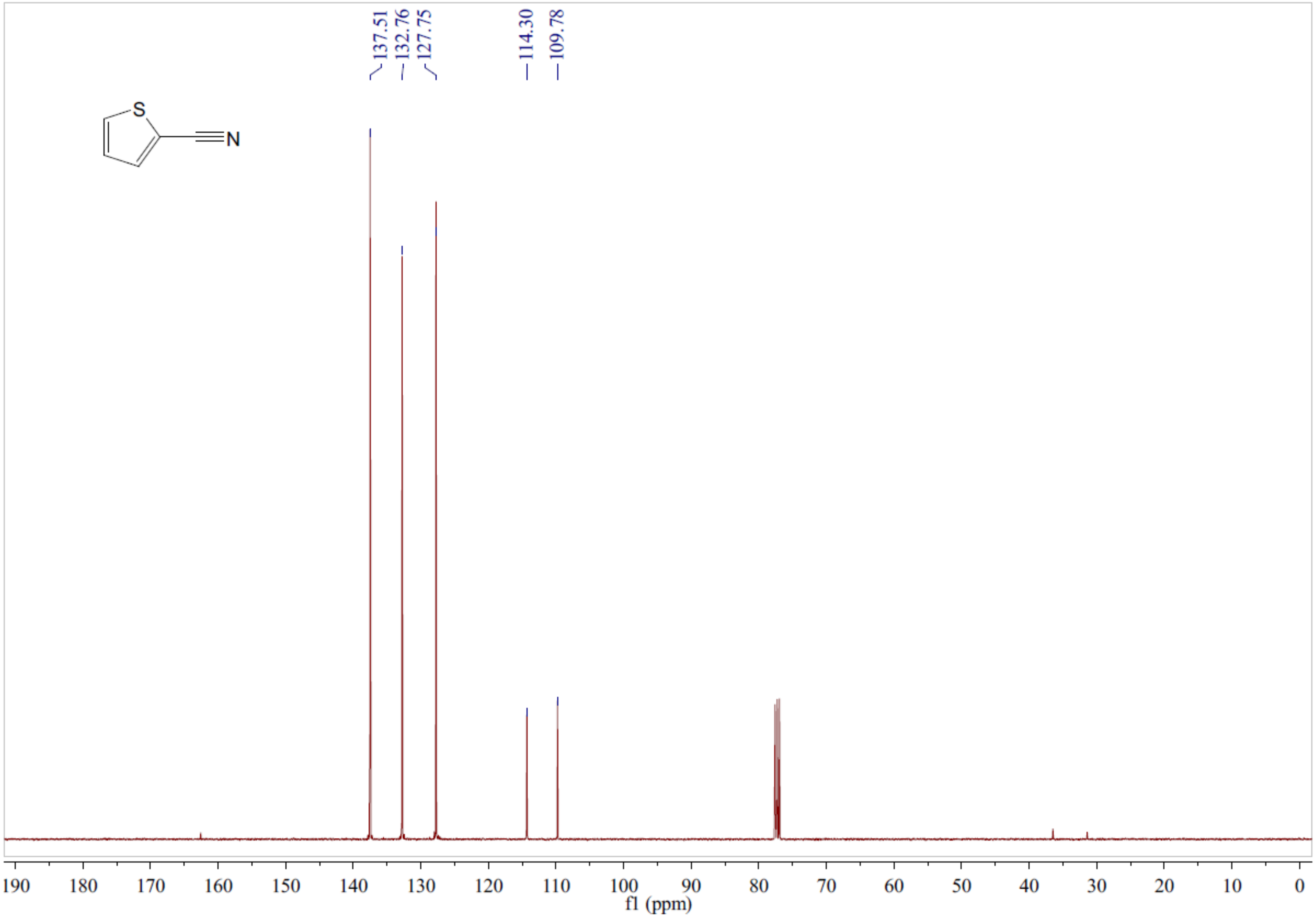

^{1}H NMR Spectra of 2x

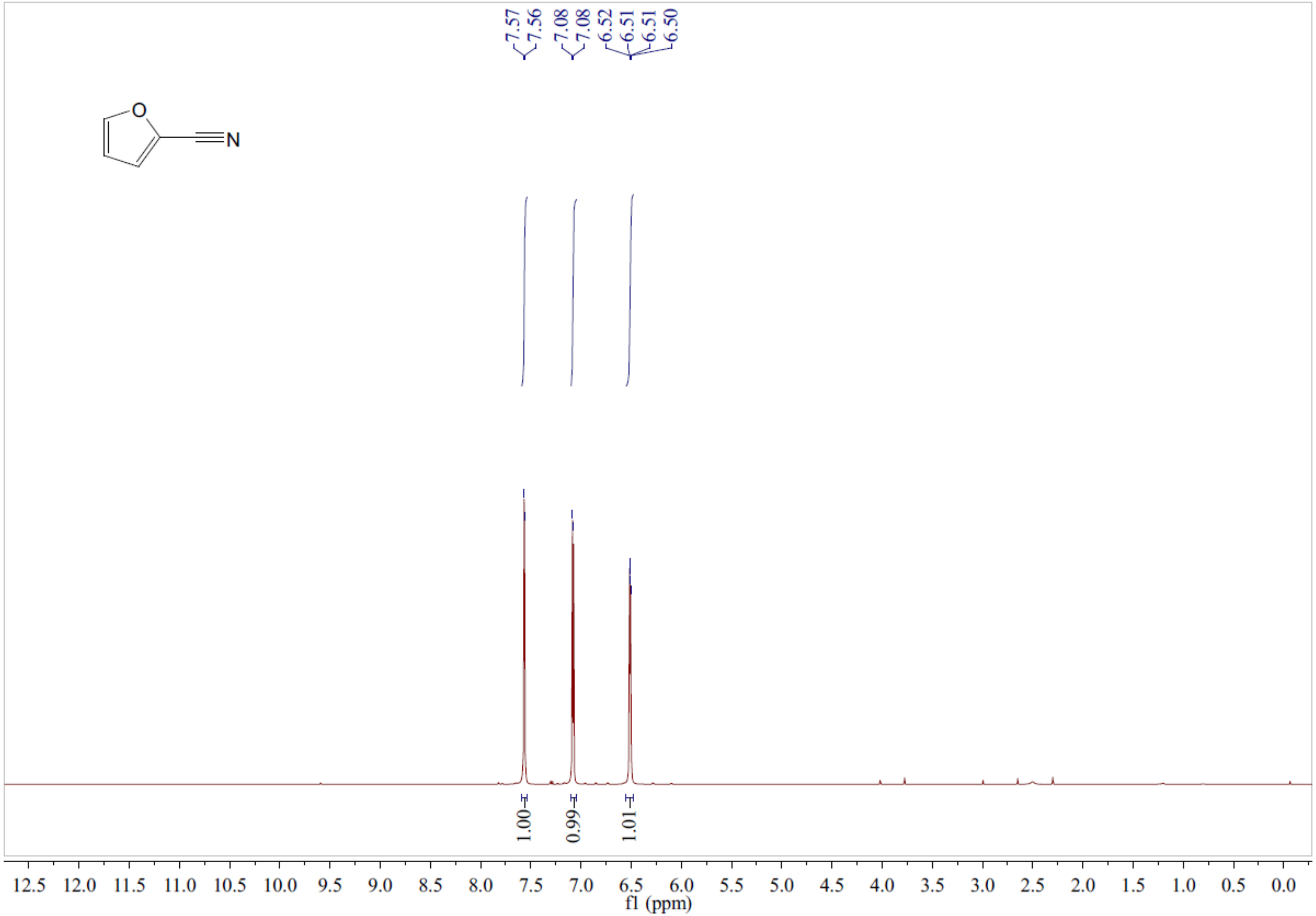

^{13}C NMR Spectra of 2x

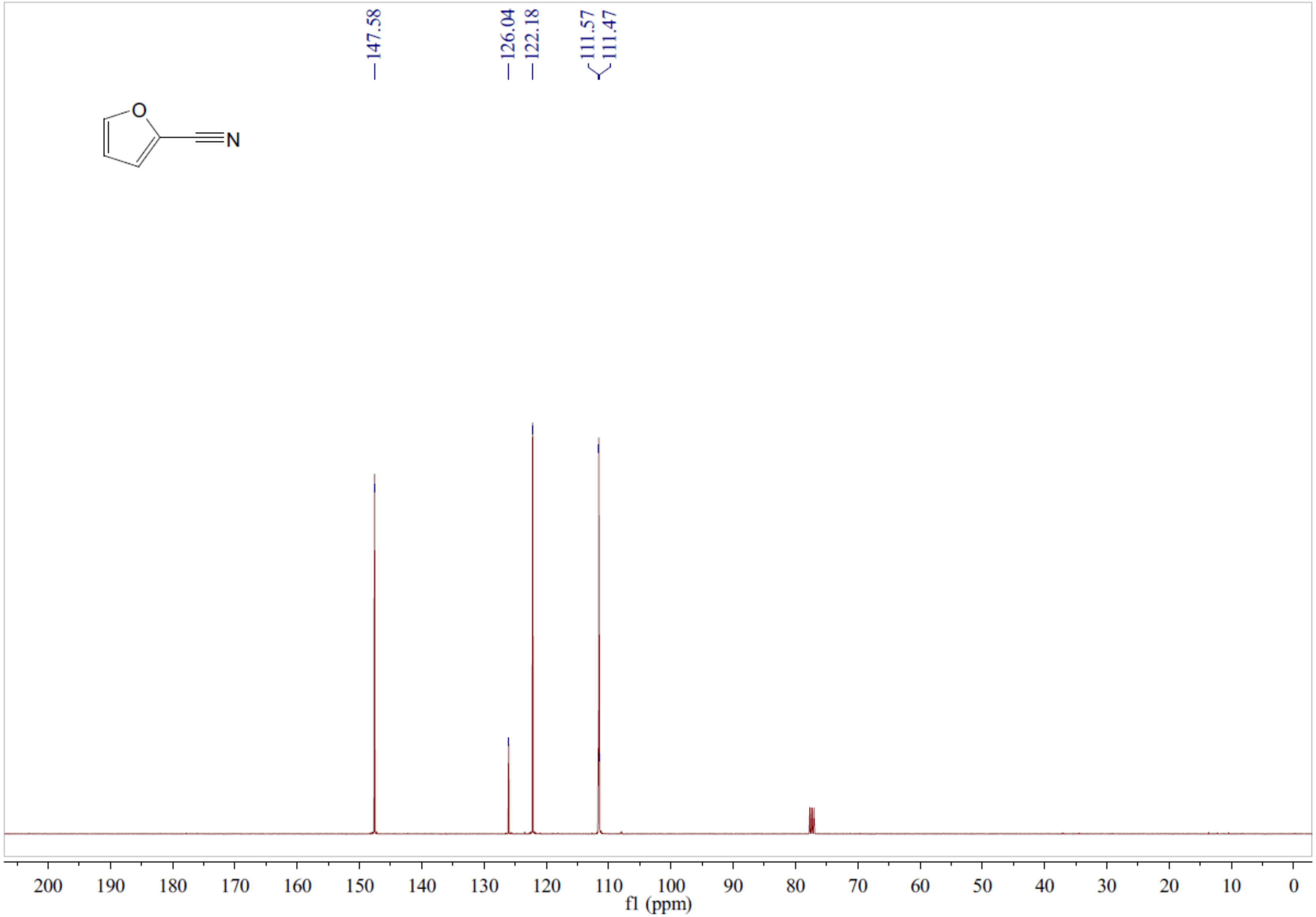

^{1}H NMR Spectra of 2aa

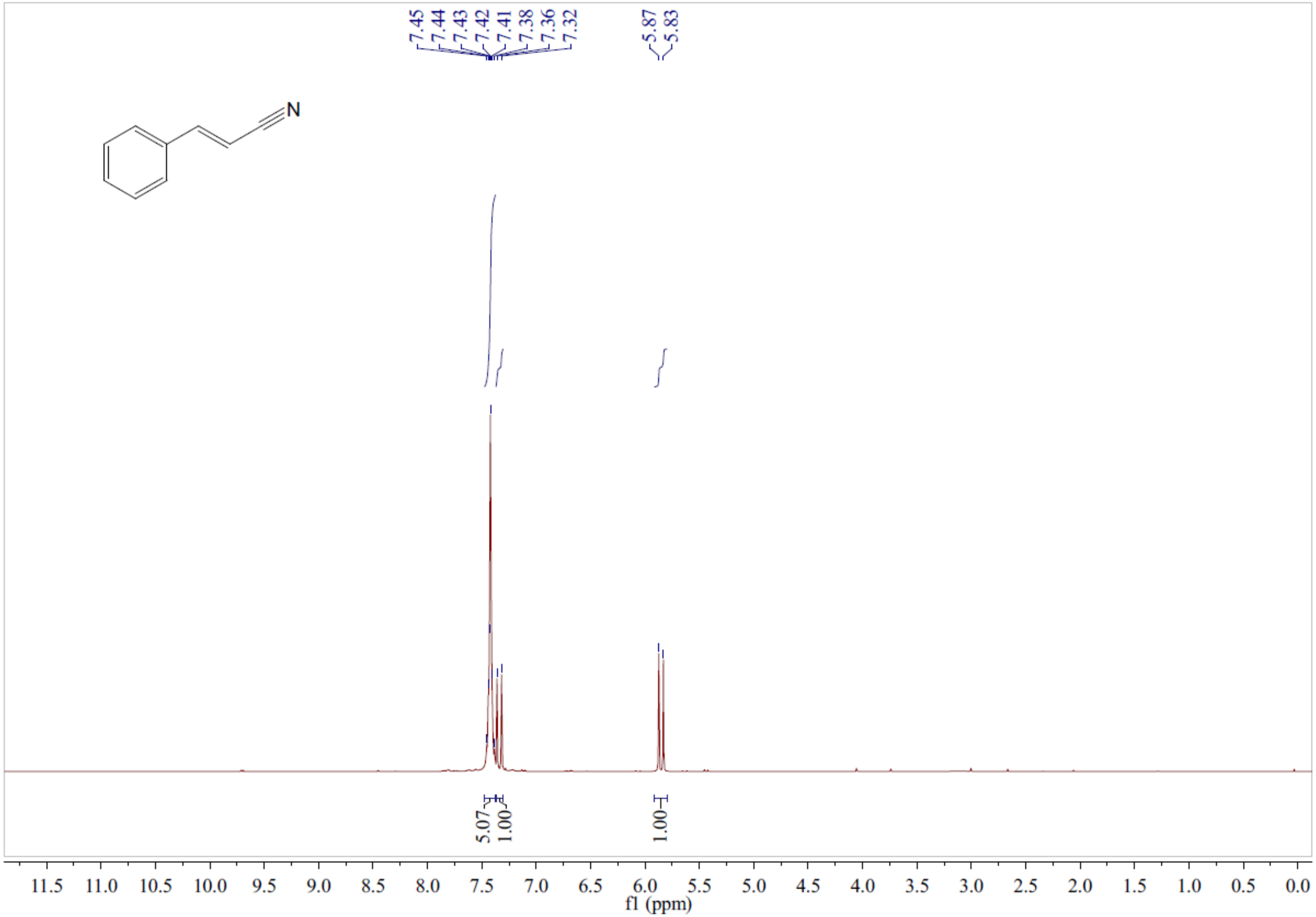

^{13}C NMR Spectra of 2aa

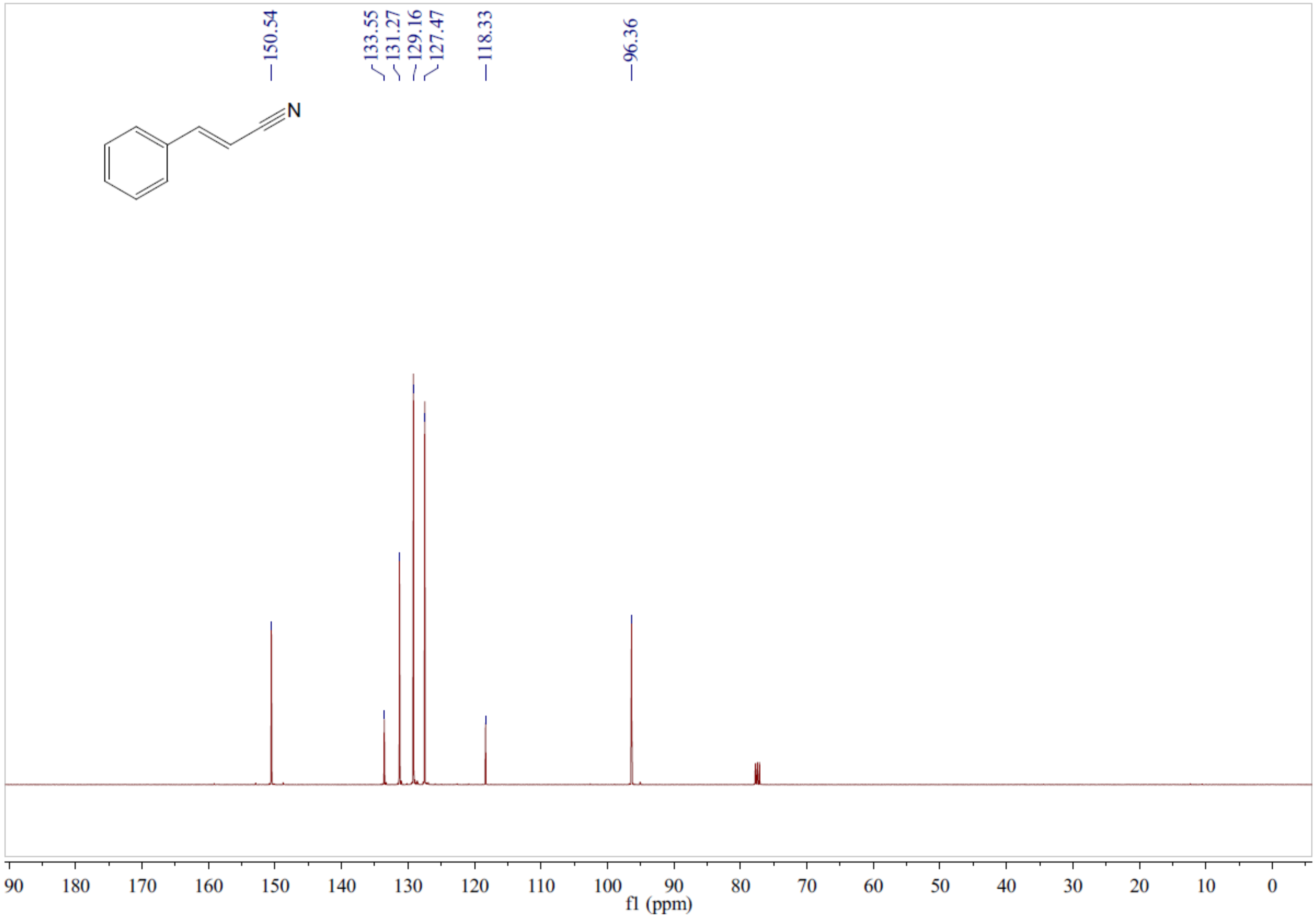

^{1}H NMR Spectra of 2ab

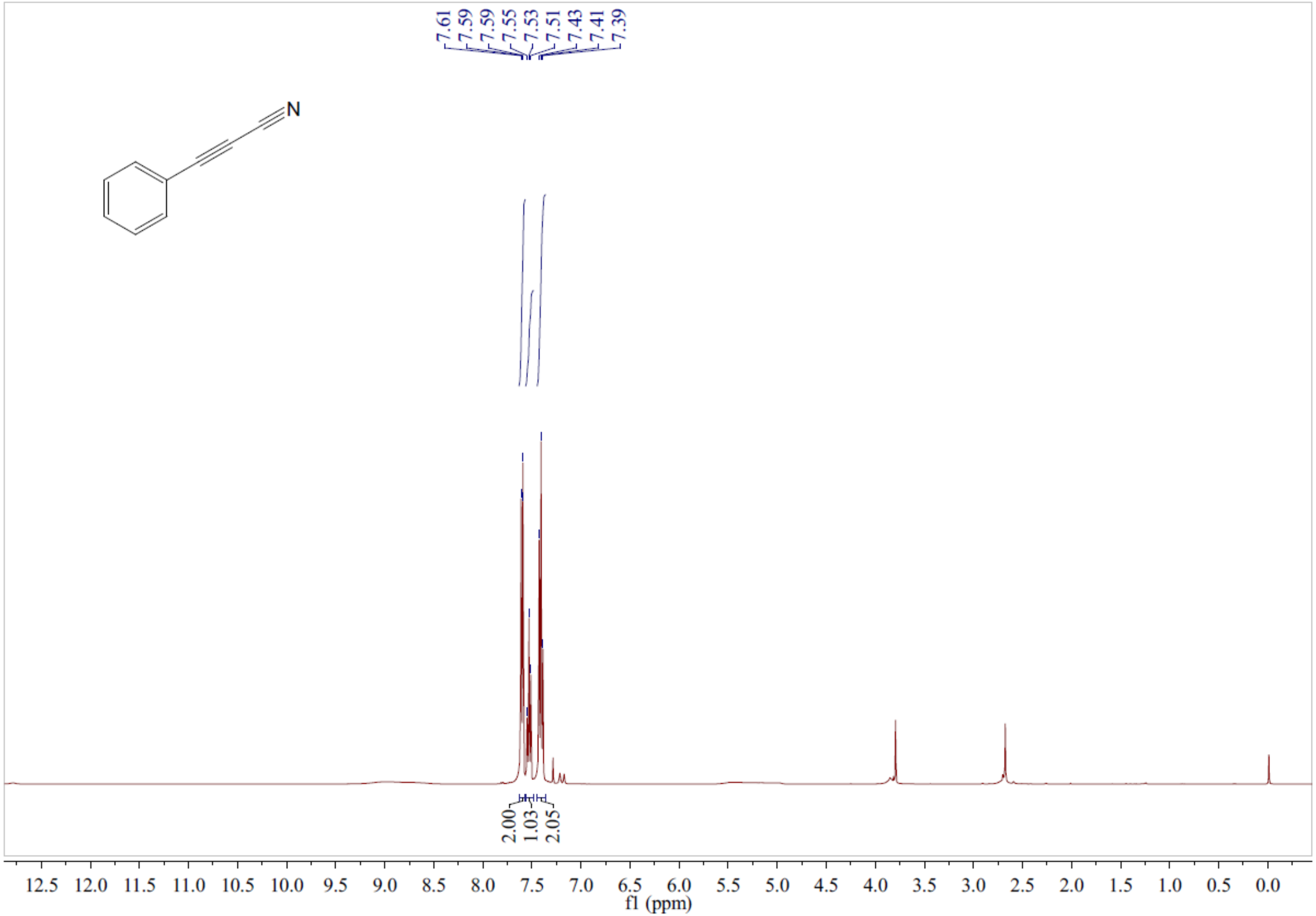

^{13}C NMR Spectra of 2ab

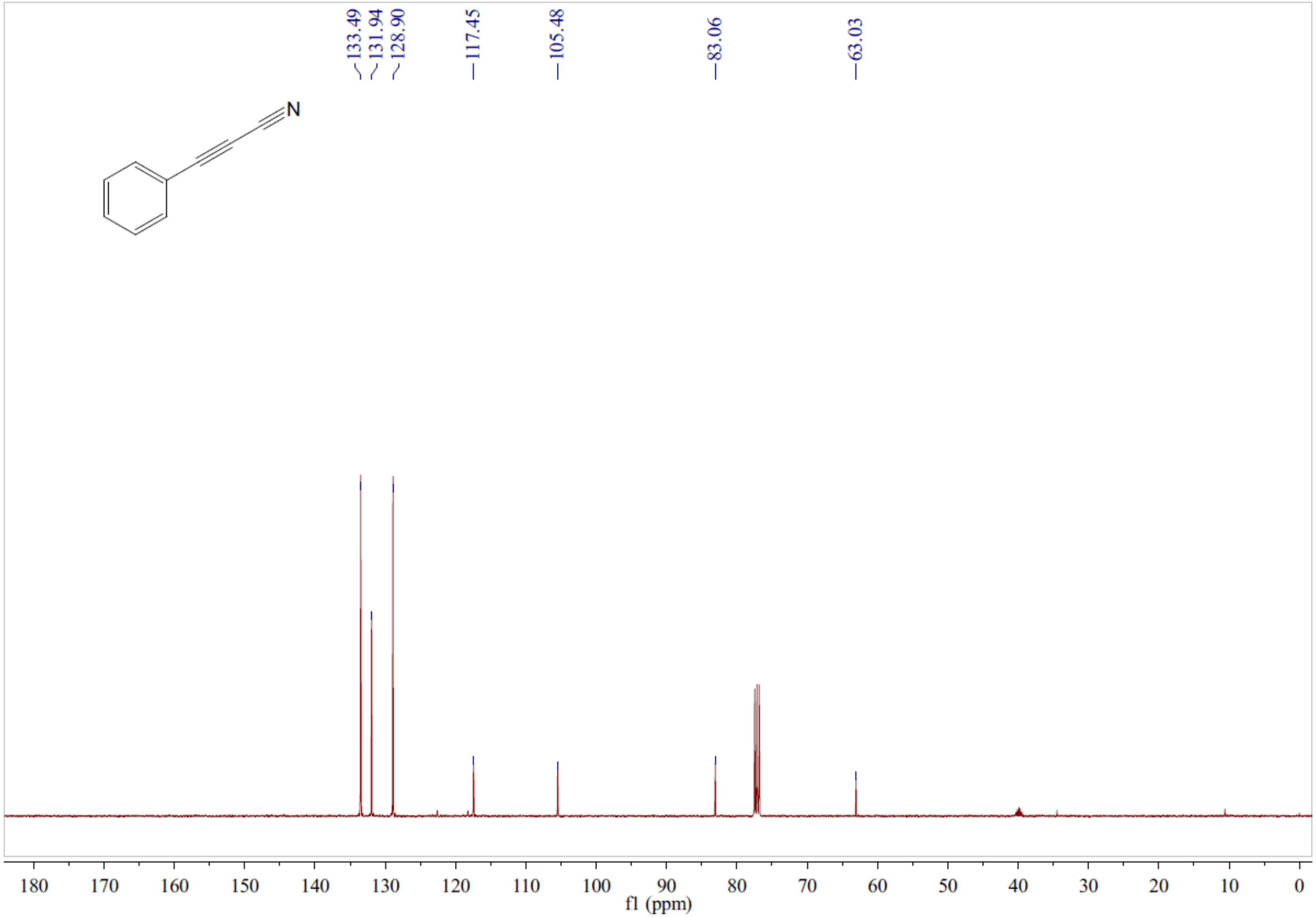

^{1}H NMR Spectra of 2ac

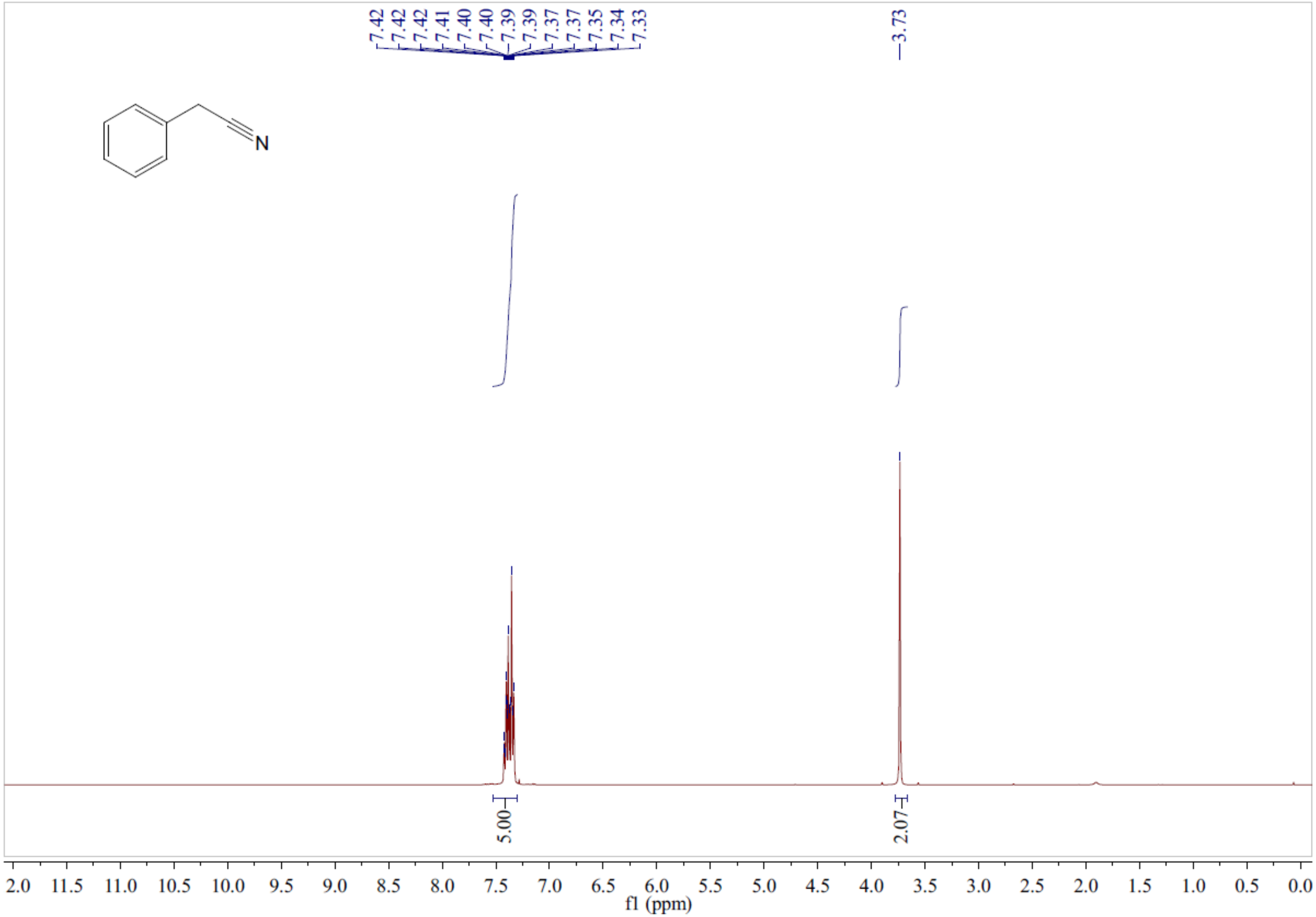

^{13}C NMR Spectra of 2ac

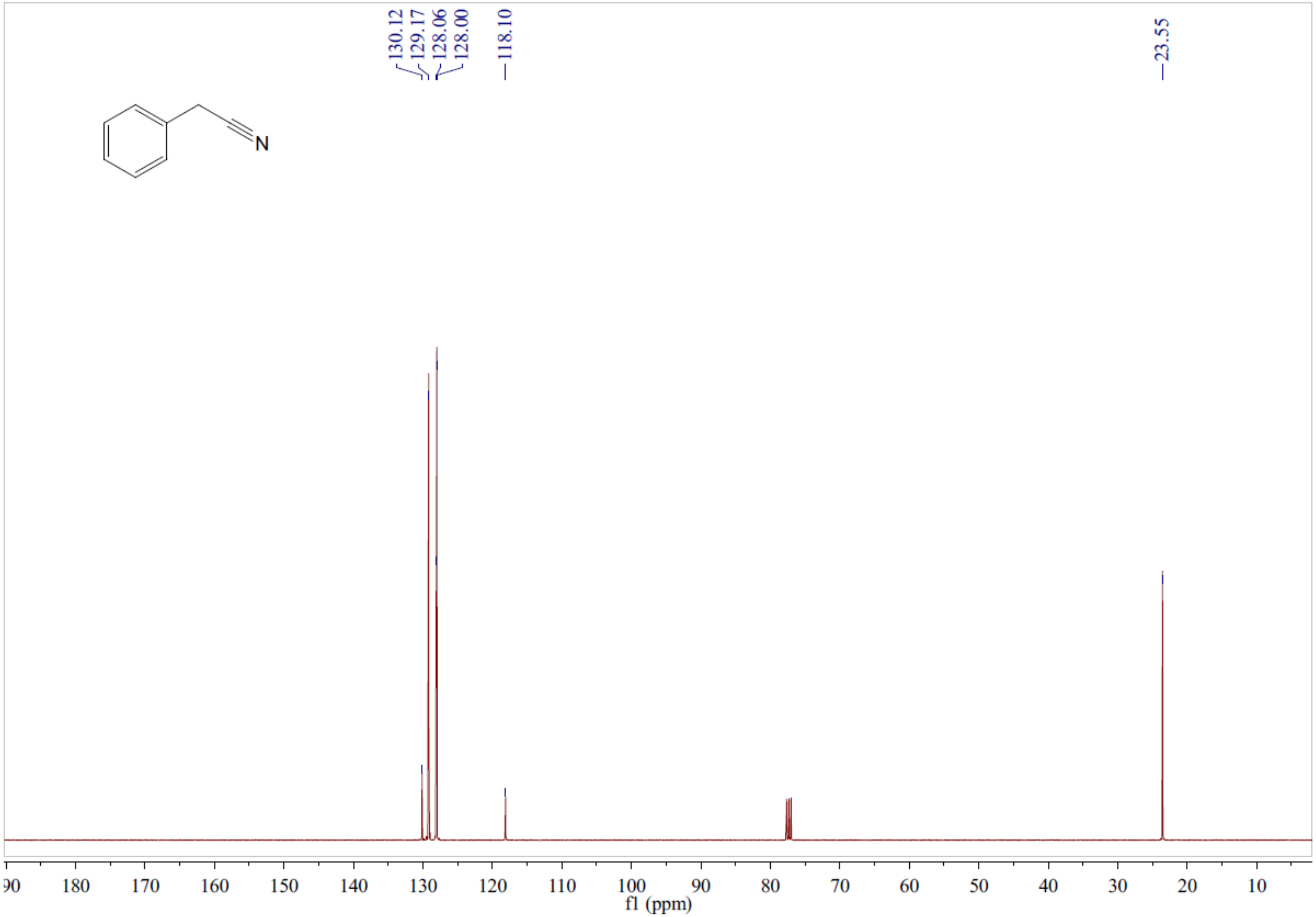

^{1}H NMR Spectra of 2ad

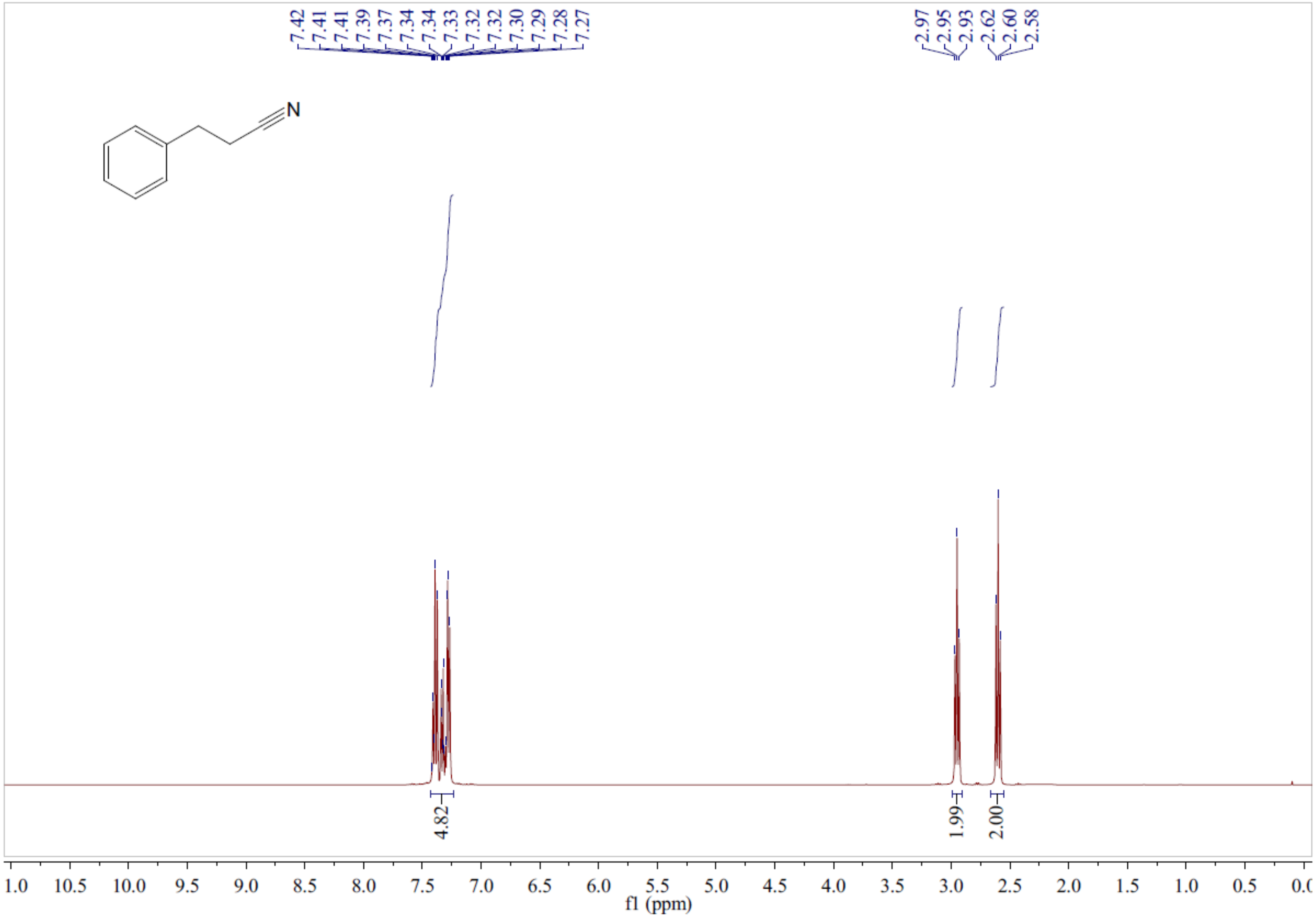

^{13}C NMR Spectra of 2ad

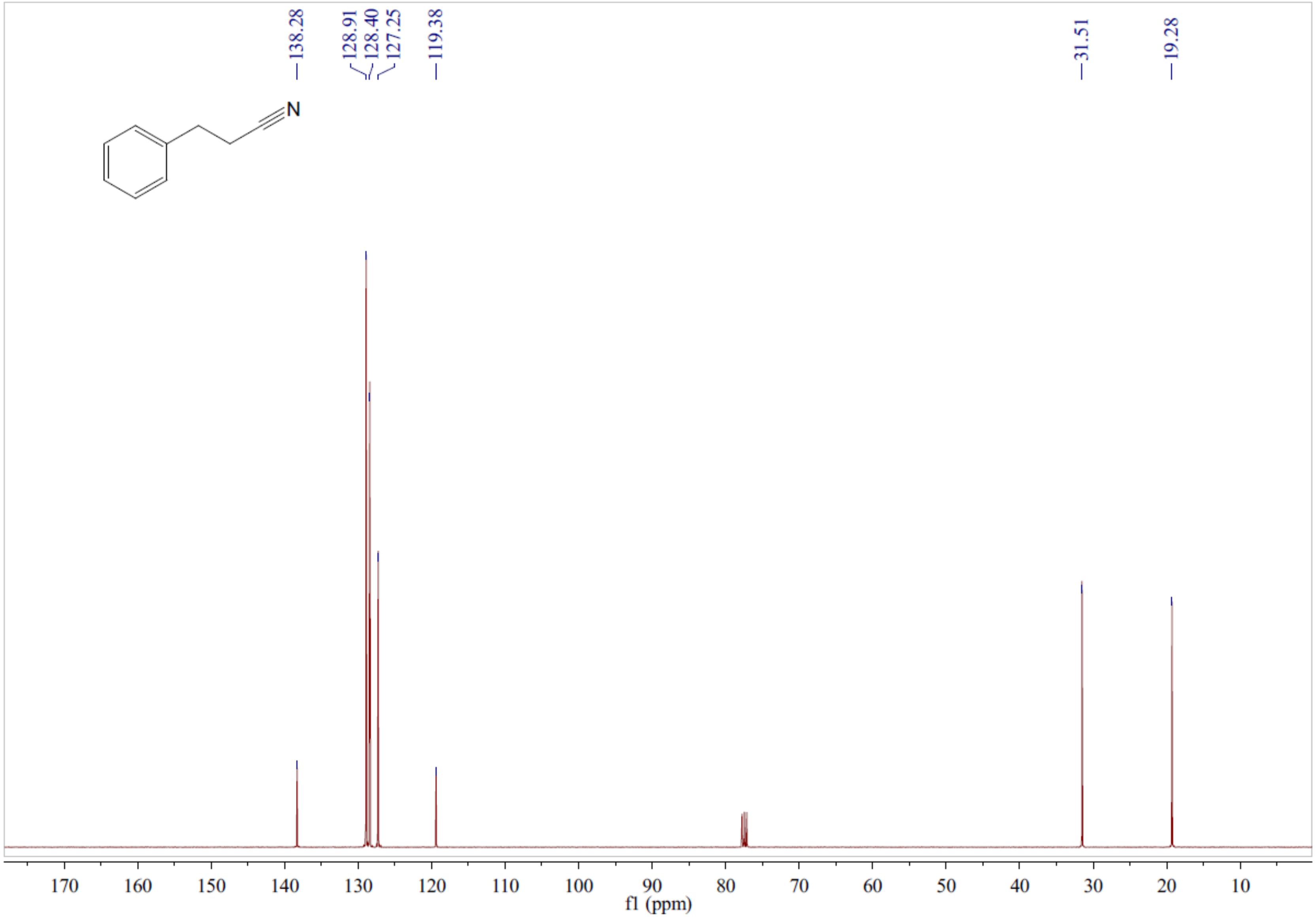

^{1}H NMR Spectra of 2ae

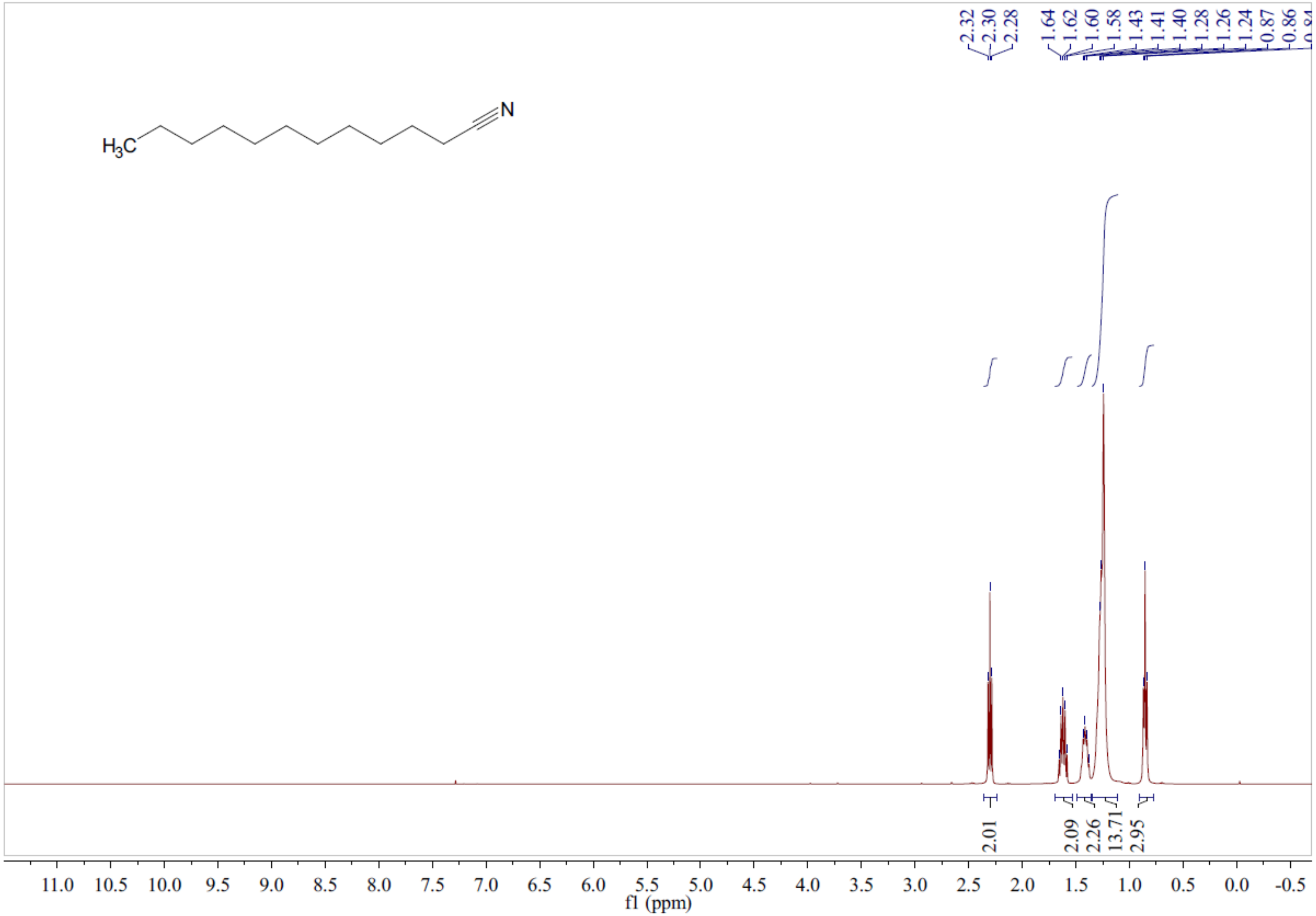

^{13}C NMR Spectra of 2ae

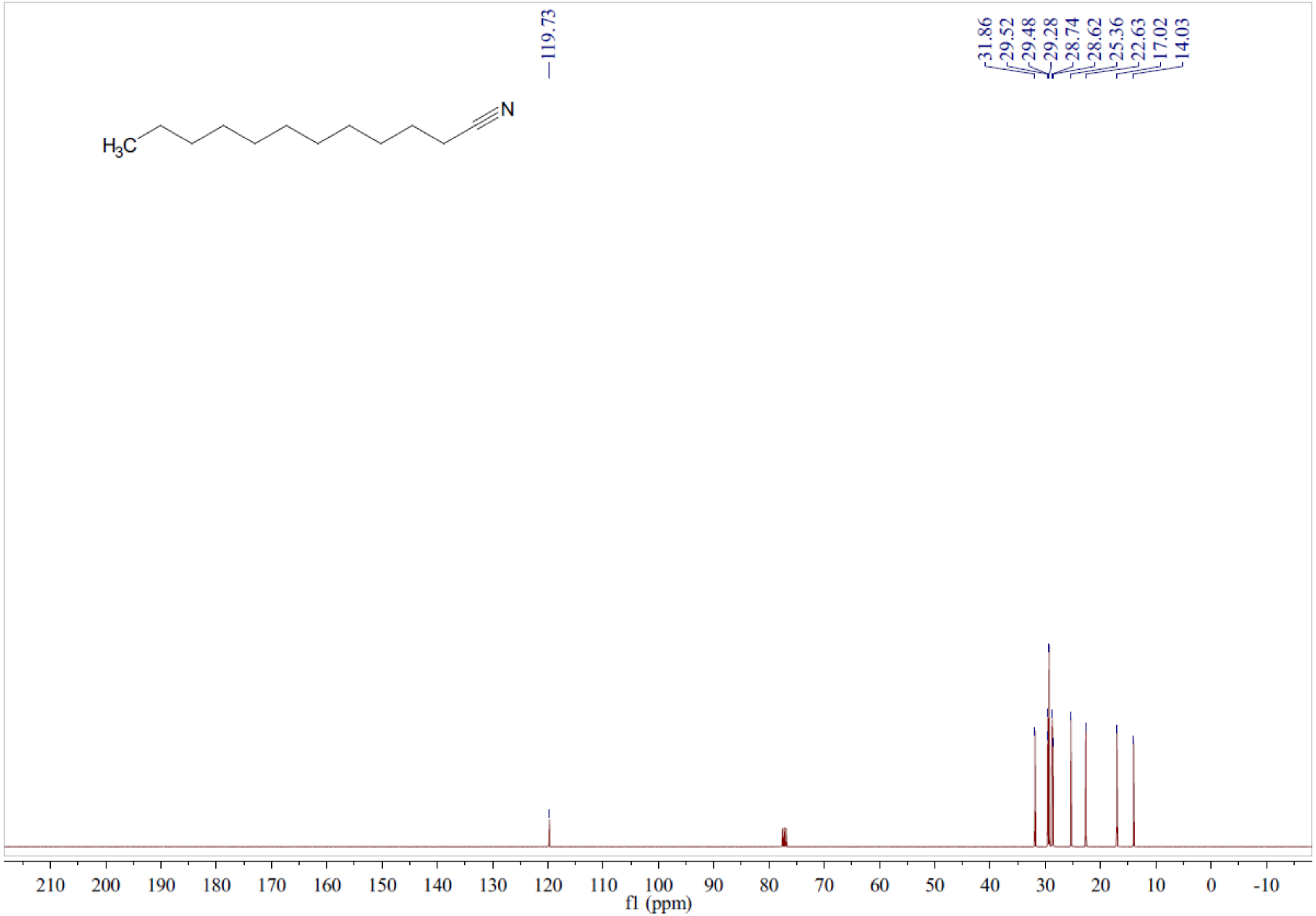

^{1}H NMR Spectra of 4a

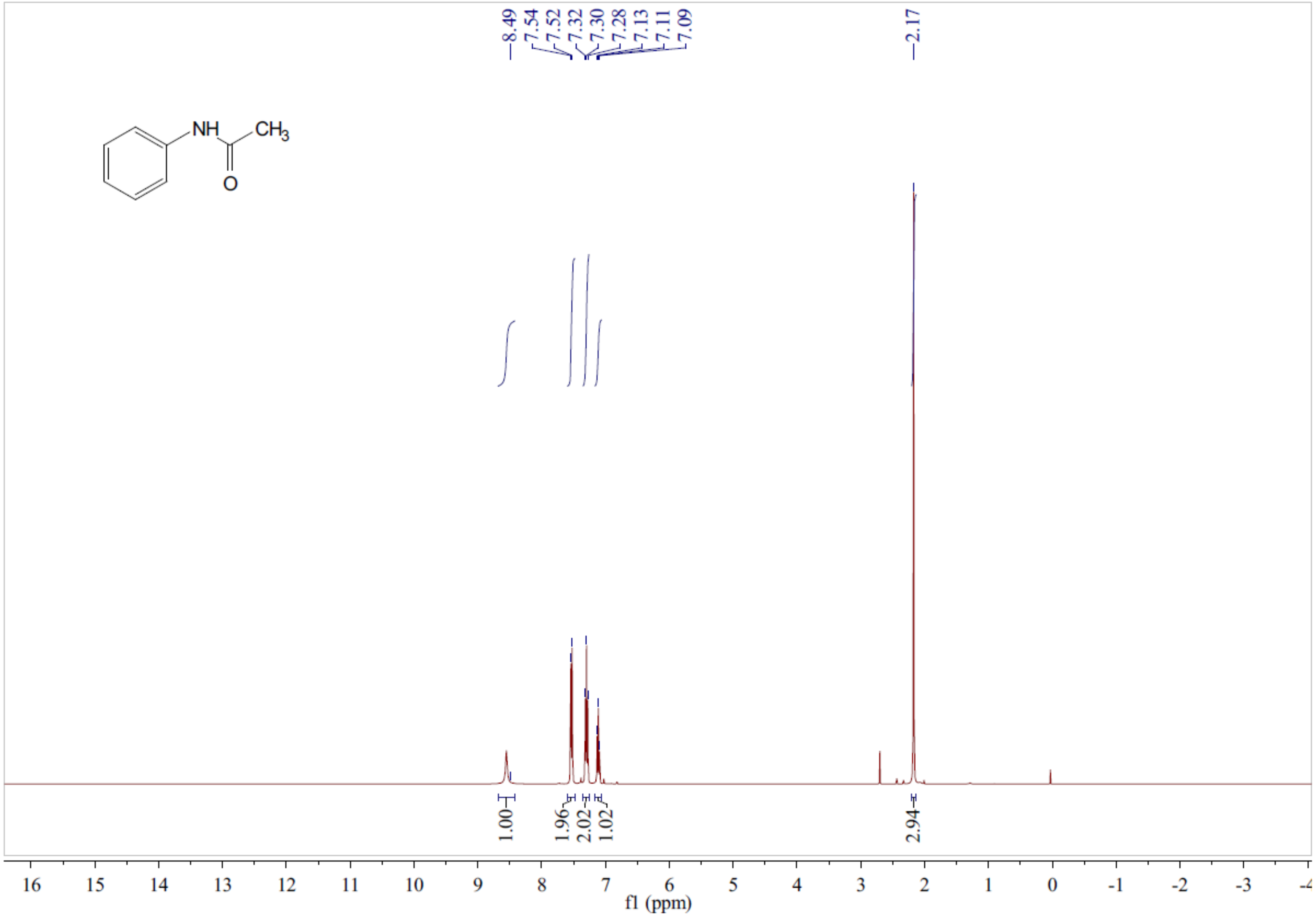

^{13}C NMR Spectra of 4a

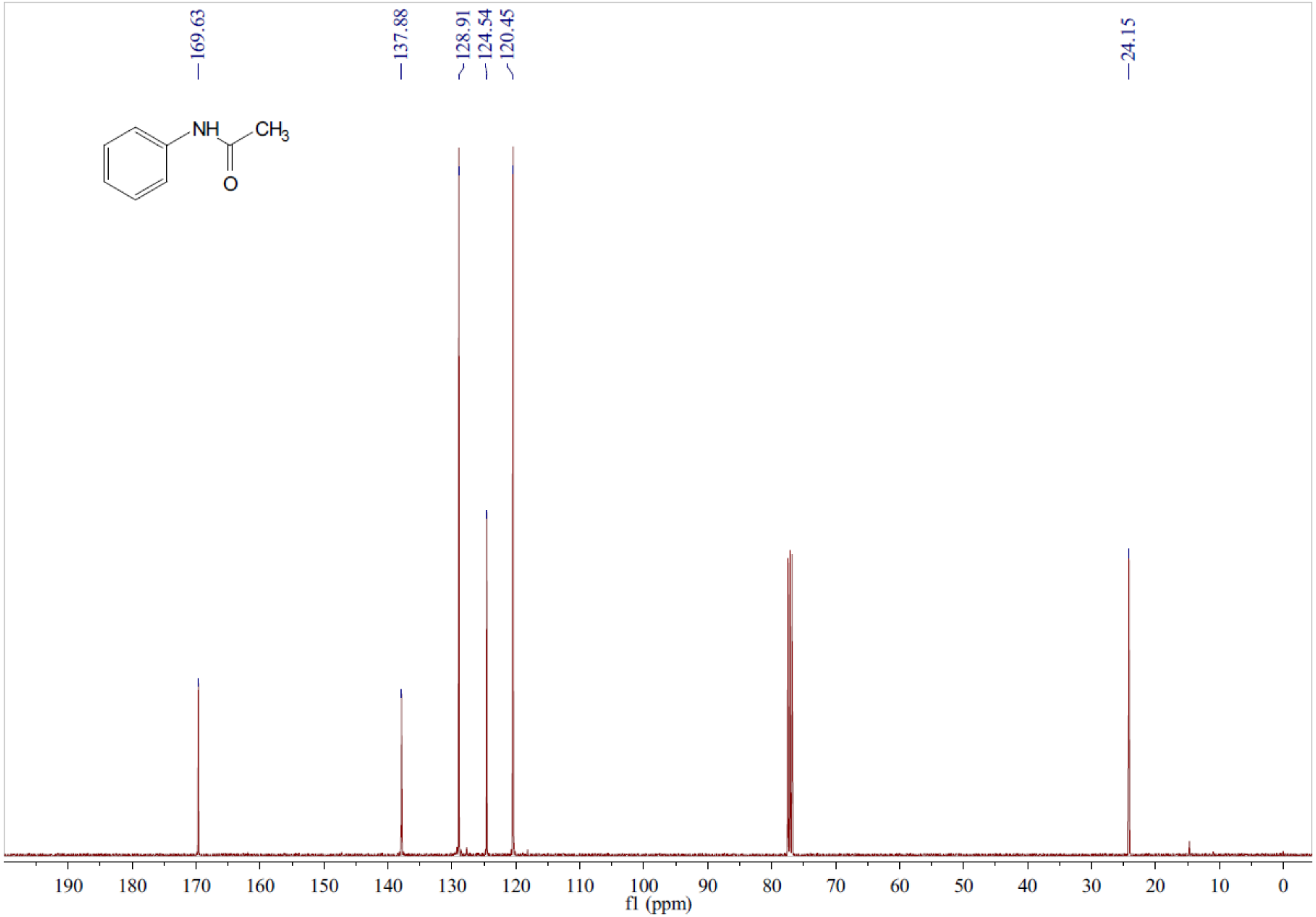

^{1}H NMR Spectra of 4b

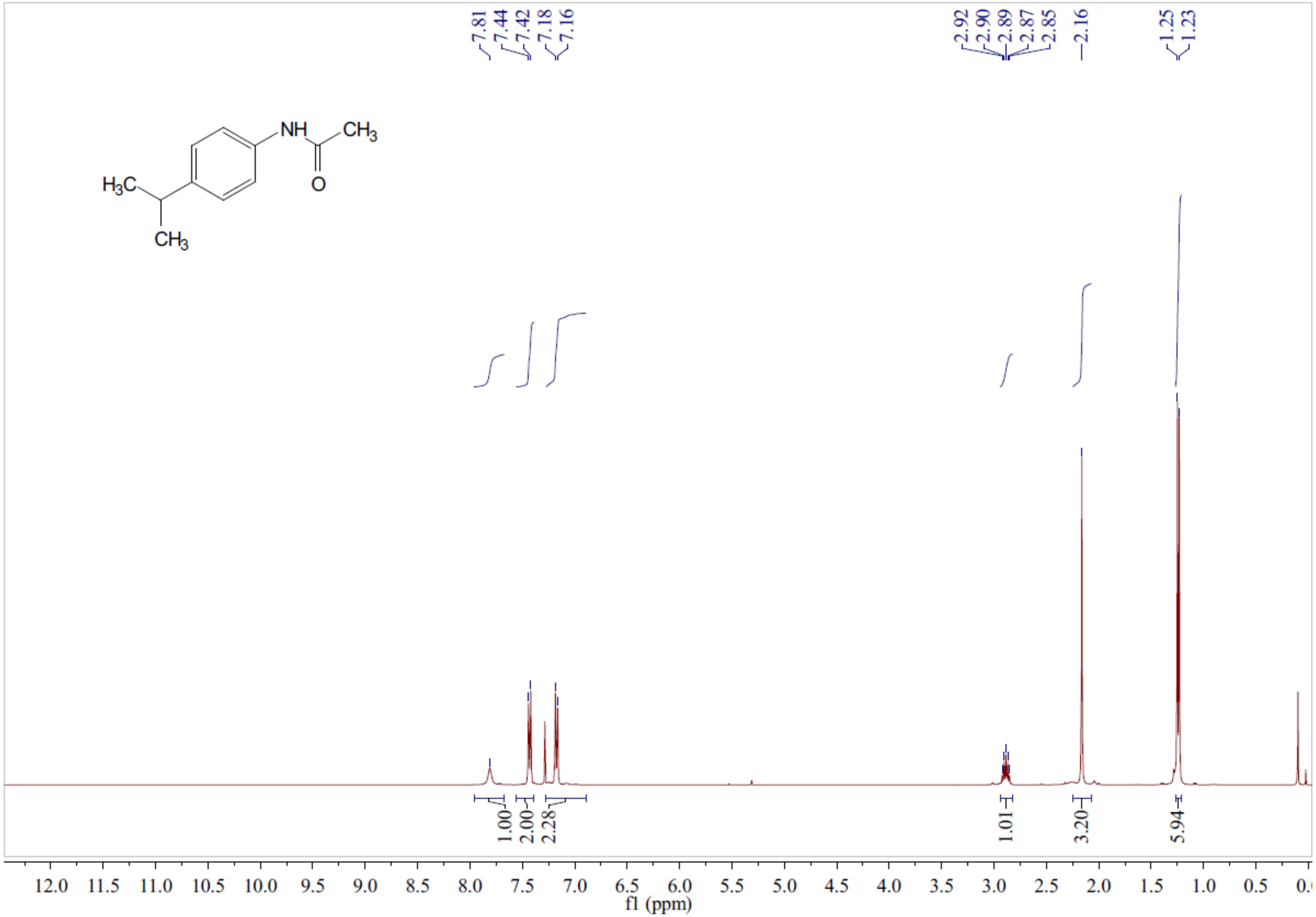

^{13}C NMR Spectra of 4b

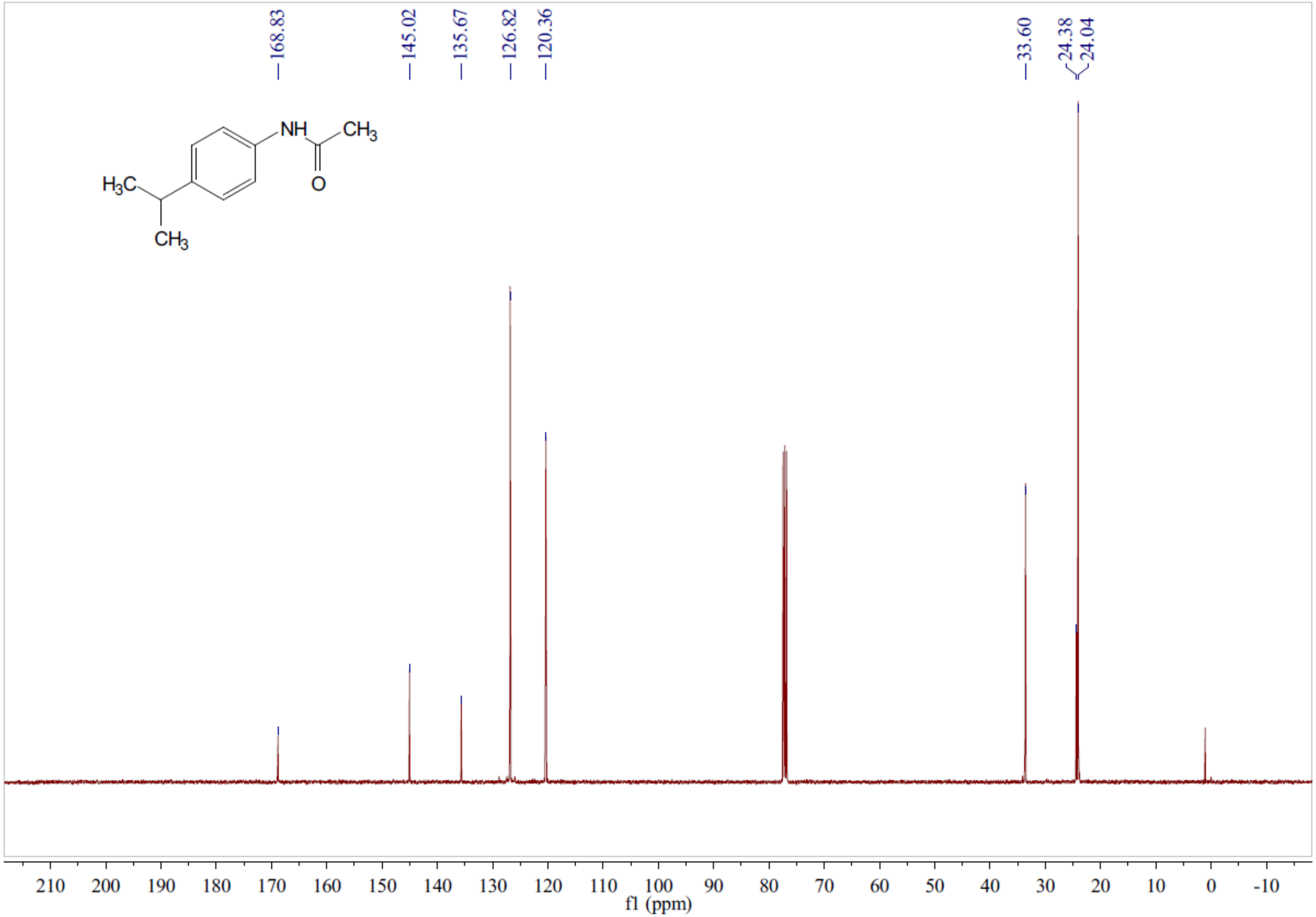

^{1}H NMR Spectra of 4c

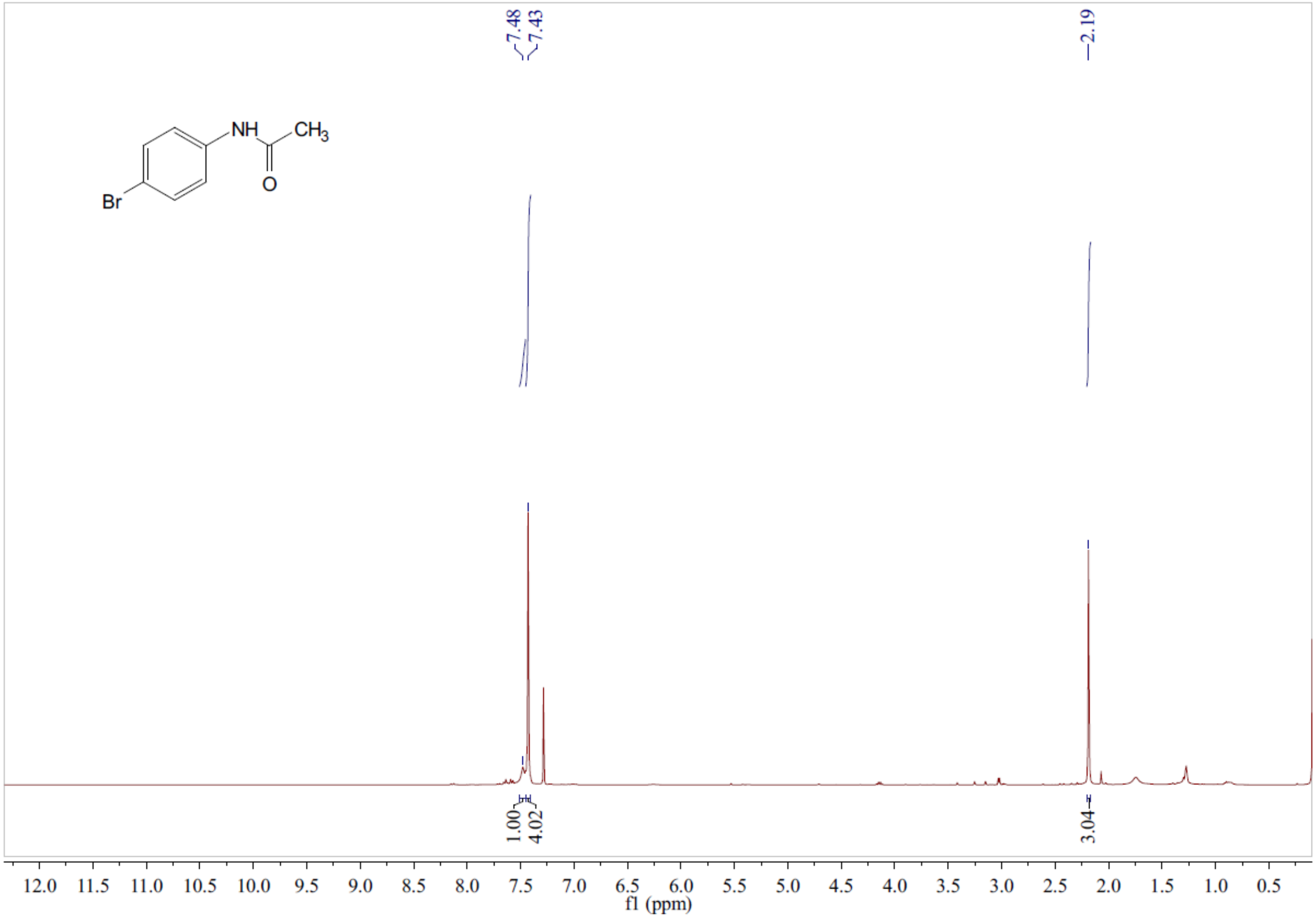

^{13}C NMR Spectra of 4c

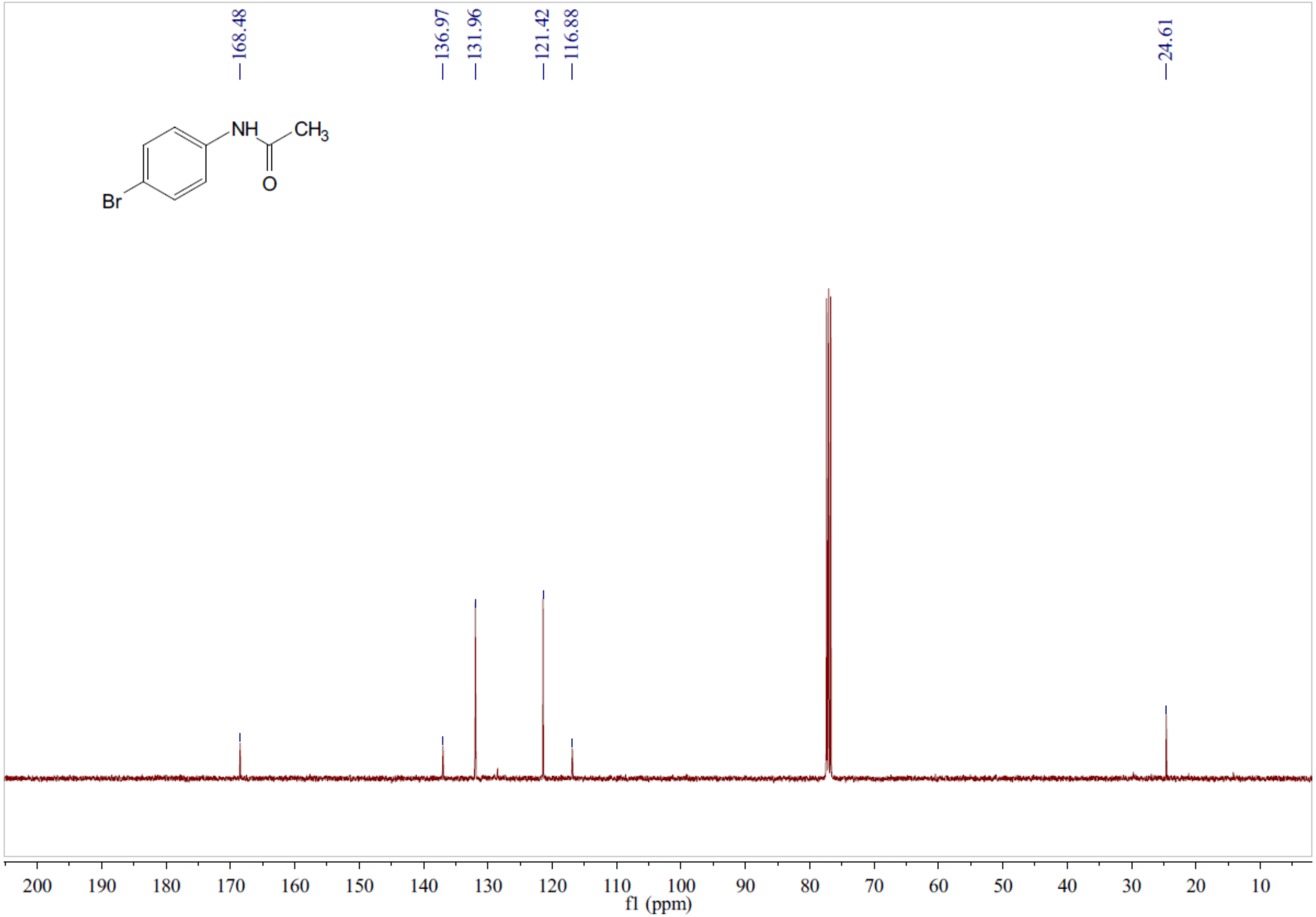

^{1}H NMR Spectra of 4d

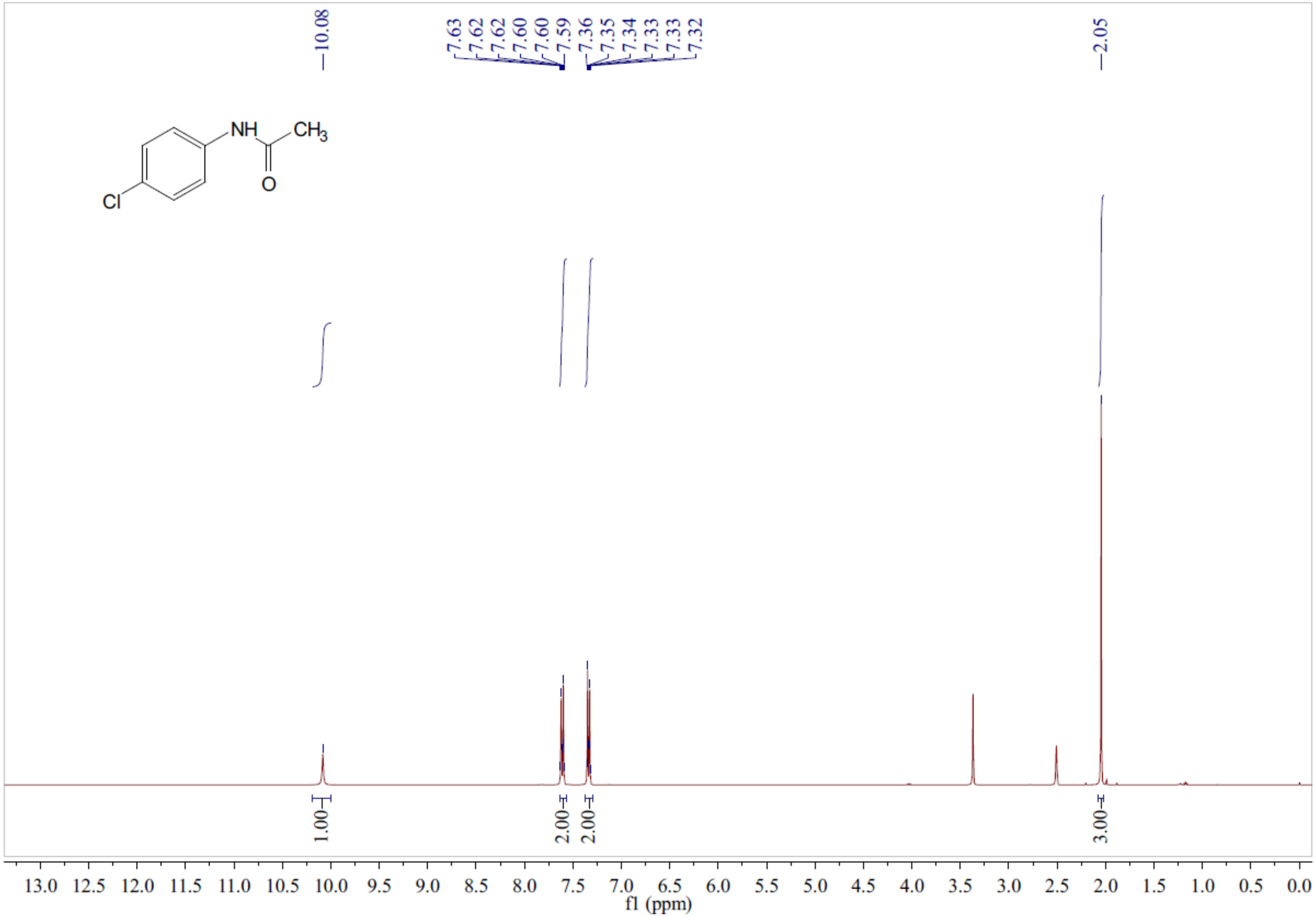

^{13}C NMR Spectra of 4d

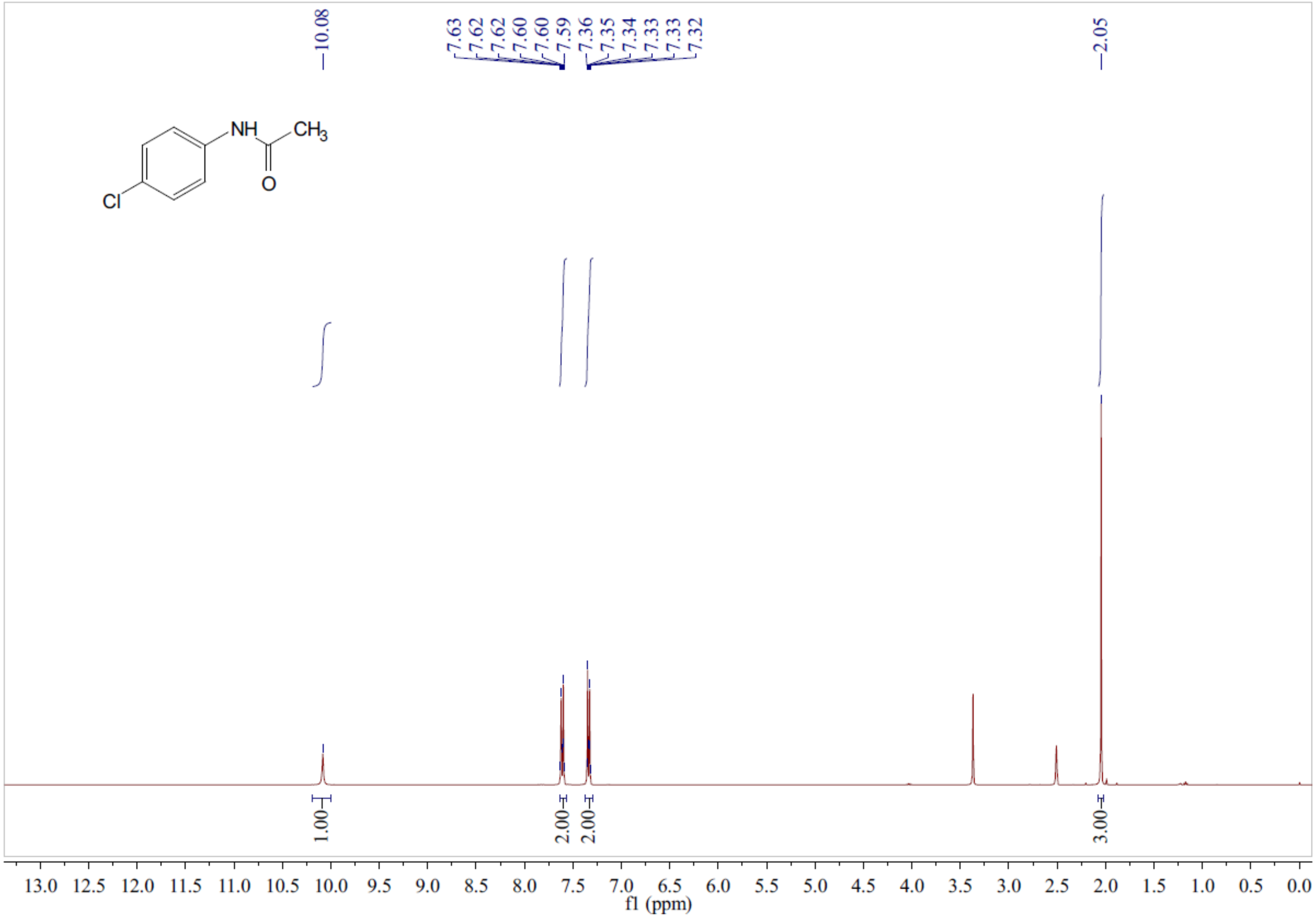

^{1}H NMR Spectra of 4e

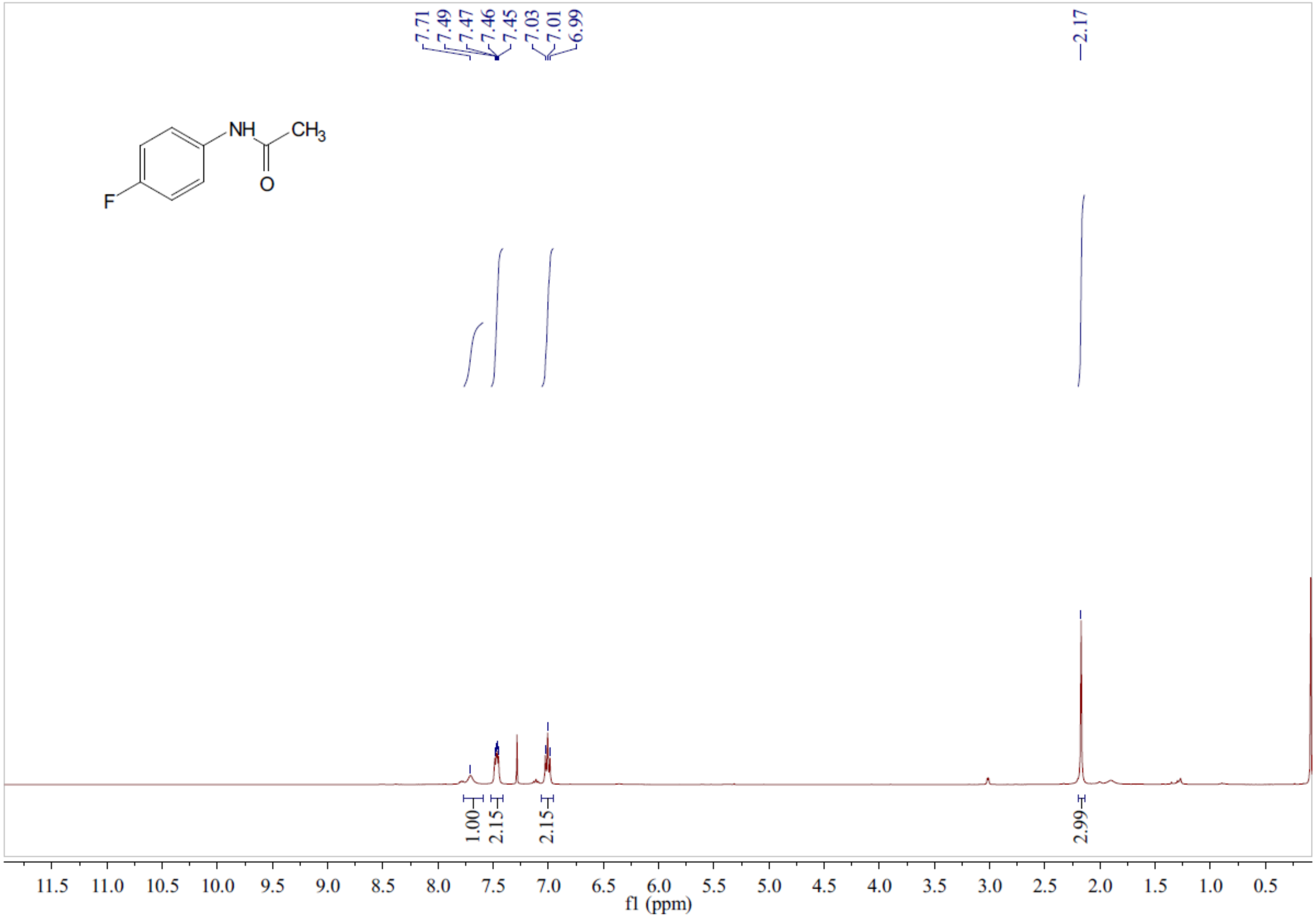

^{13}C NMR Spectra of 4e

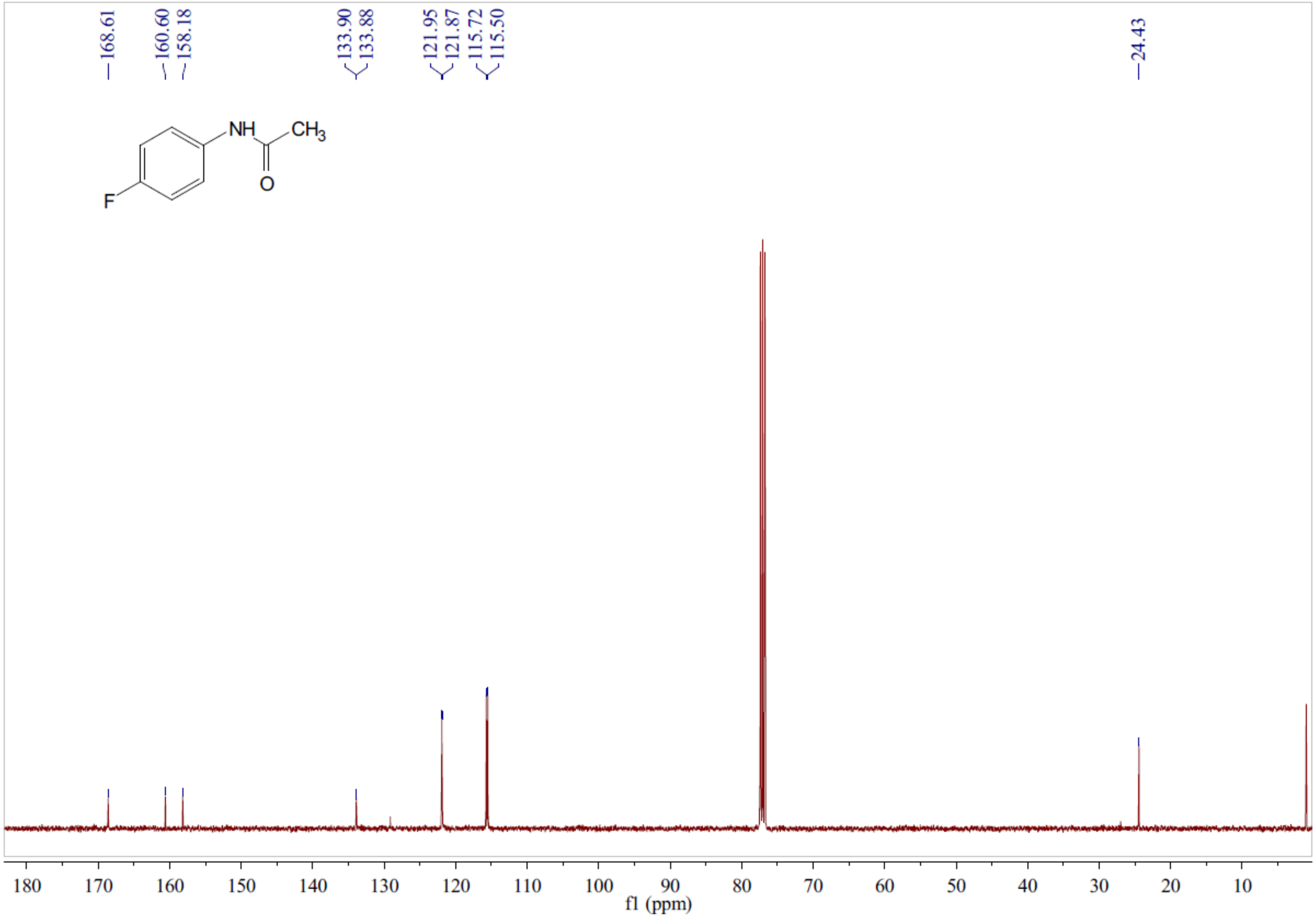

^{1}H NMR Spectra of 4f

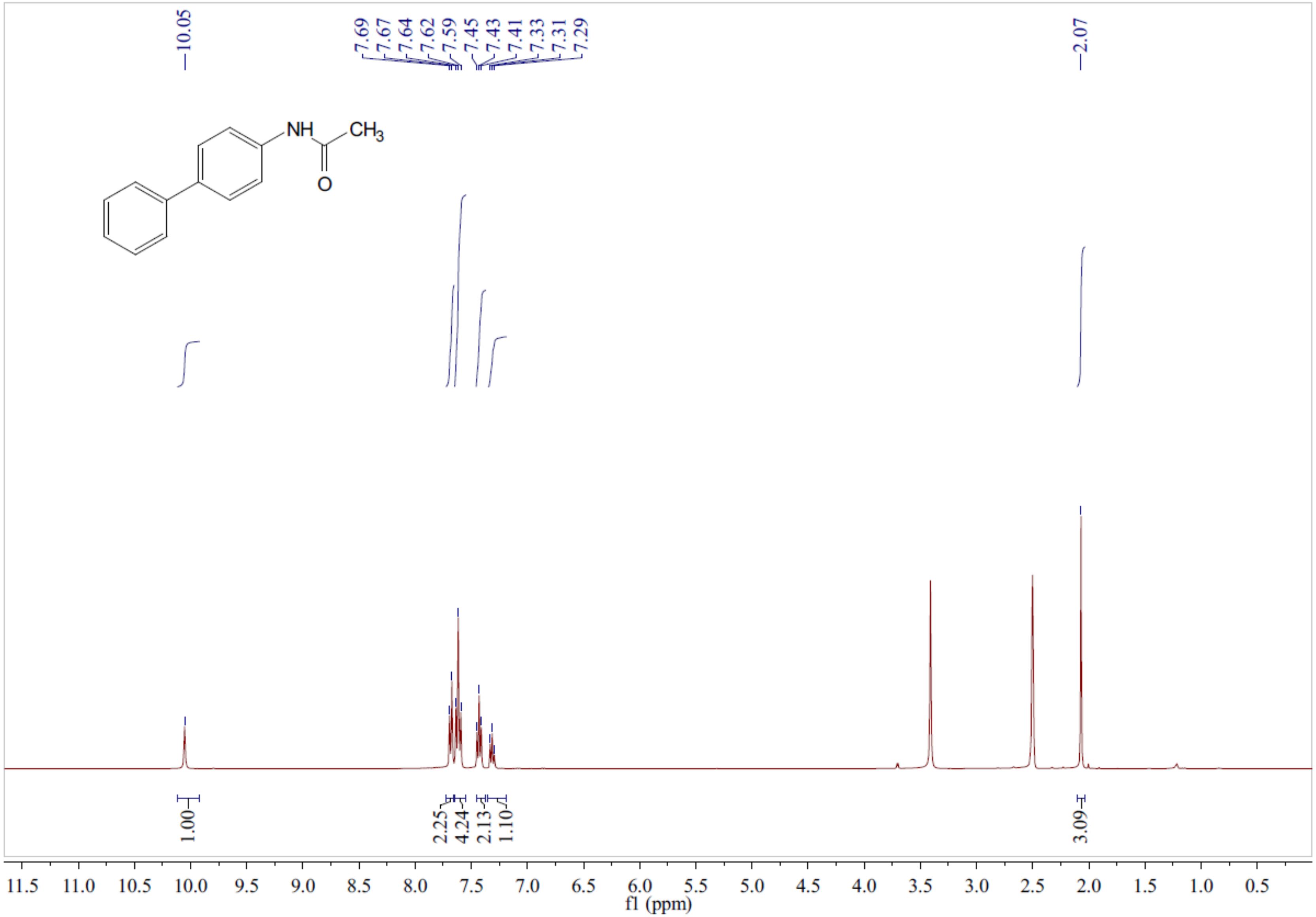

^{13}C NMR Spectra of 4f

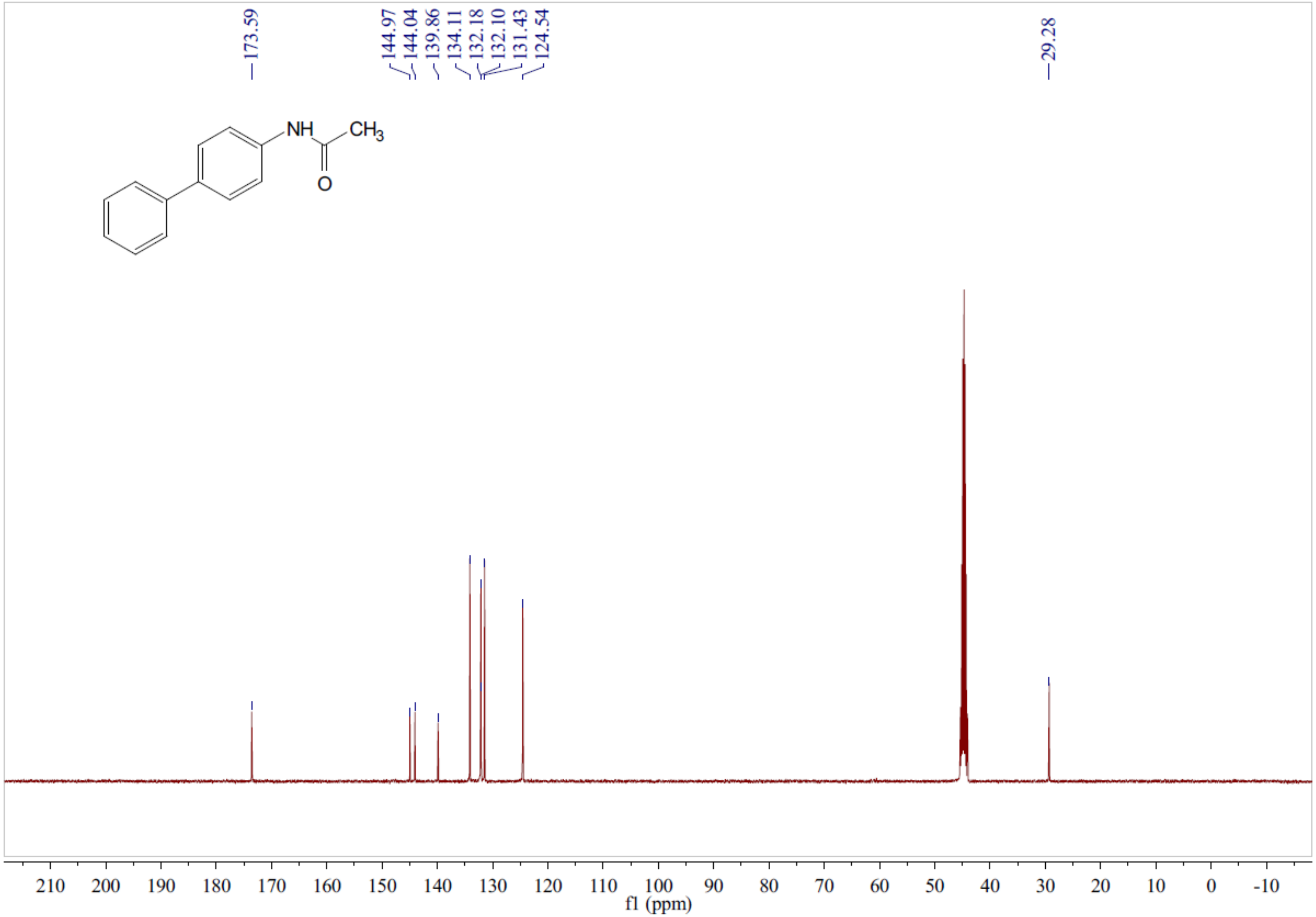

^{1}H NMR Spectra of 4g

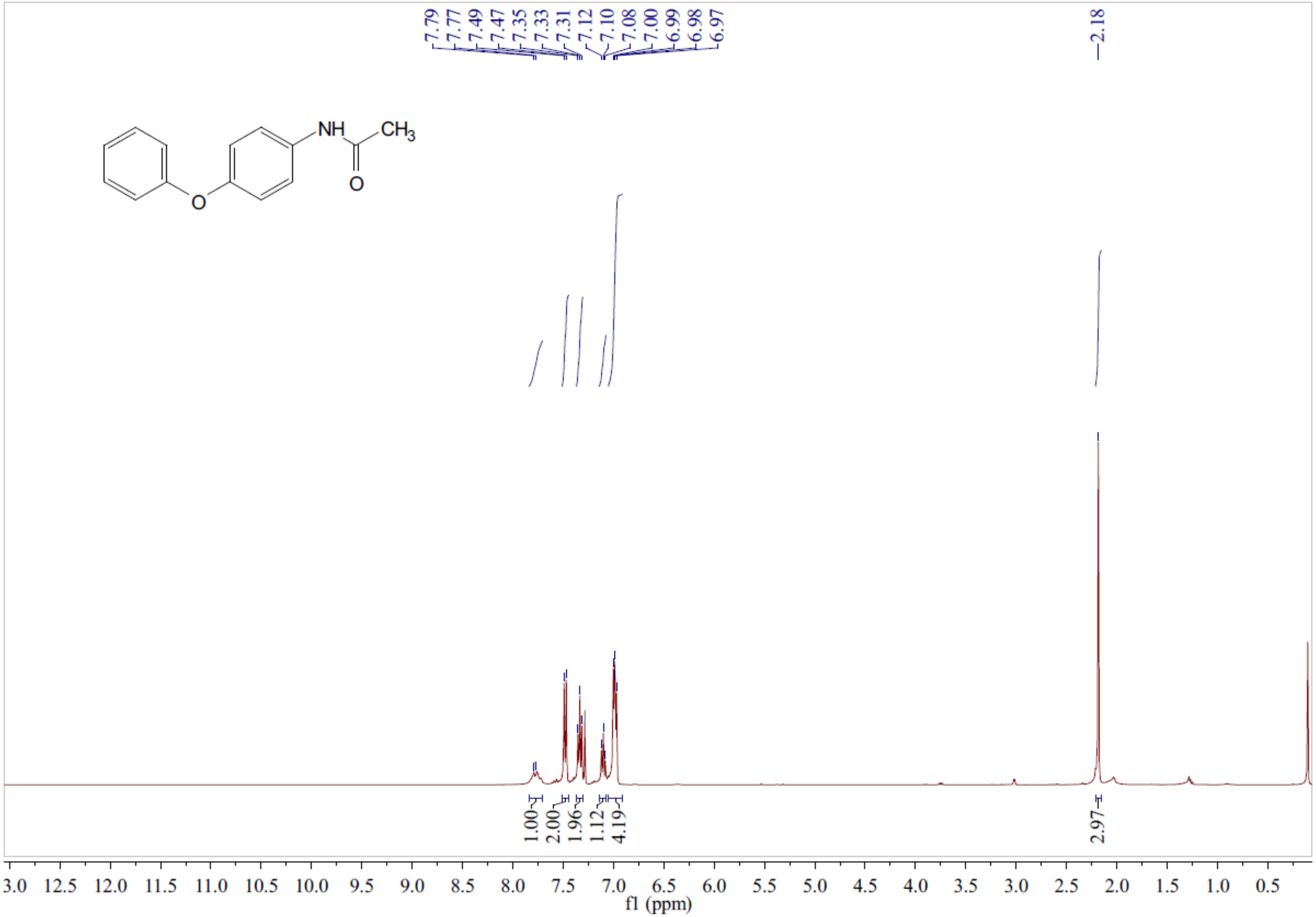

^{13}C NMR Spectra of 4g

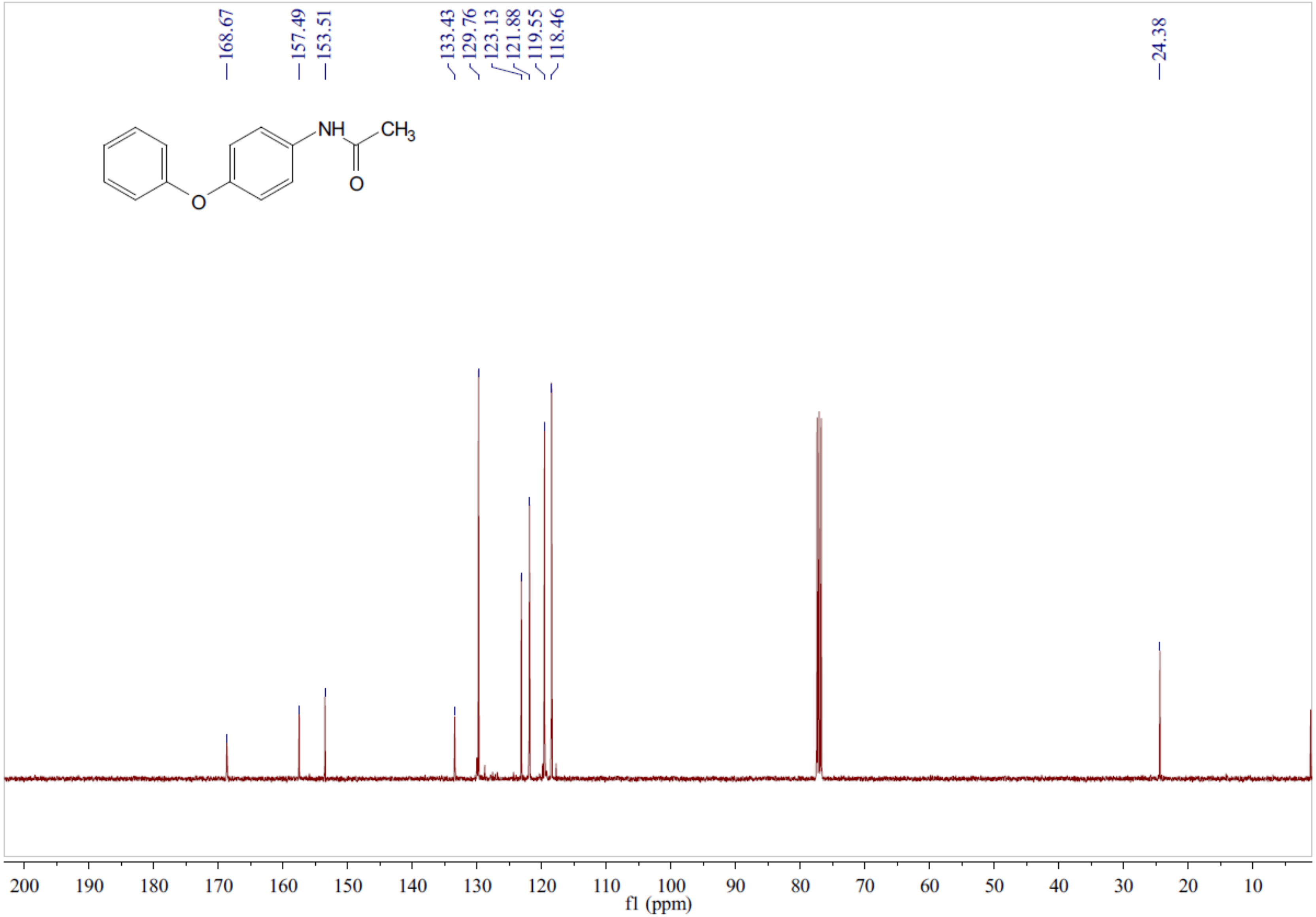

^{1}H NMR Spectra of 4h

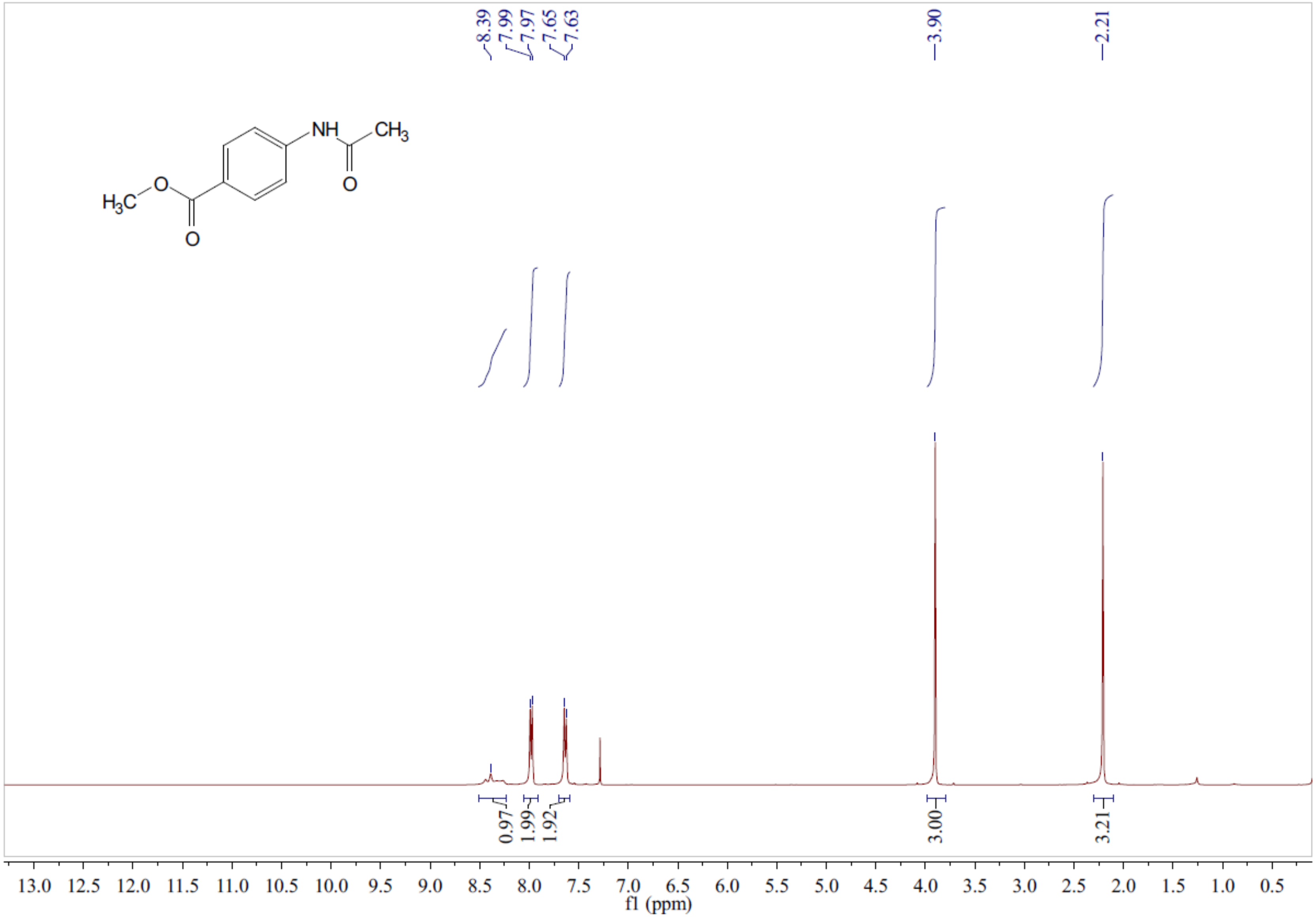

^{13}C NMR Spectra of 4h

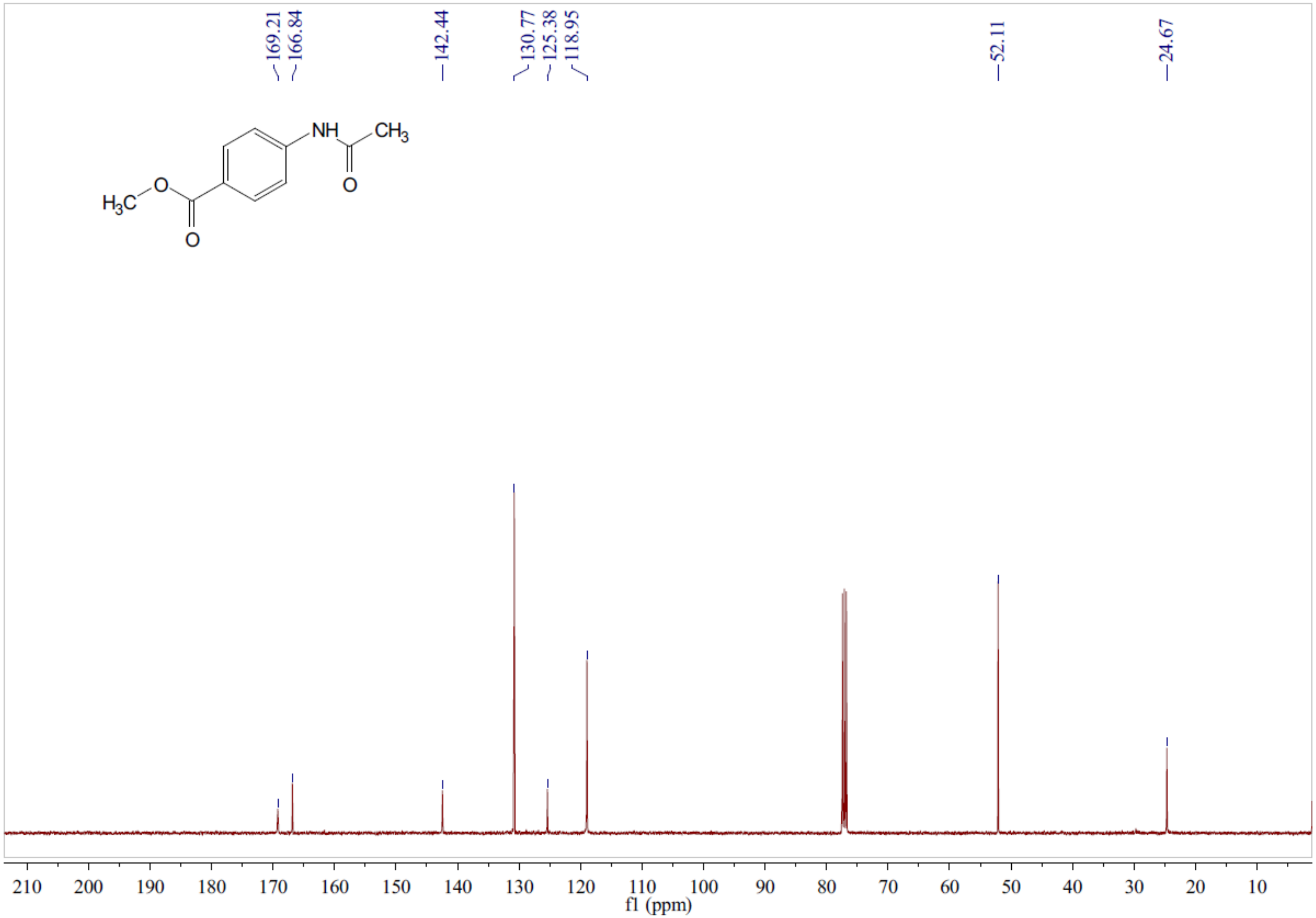

^{1}H NMR Spectra of 4i

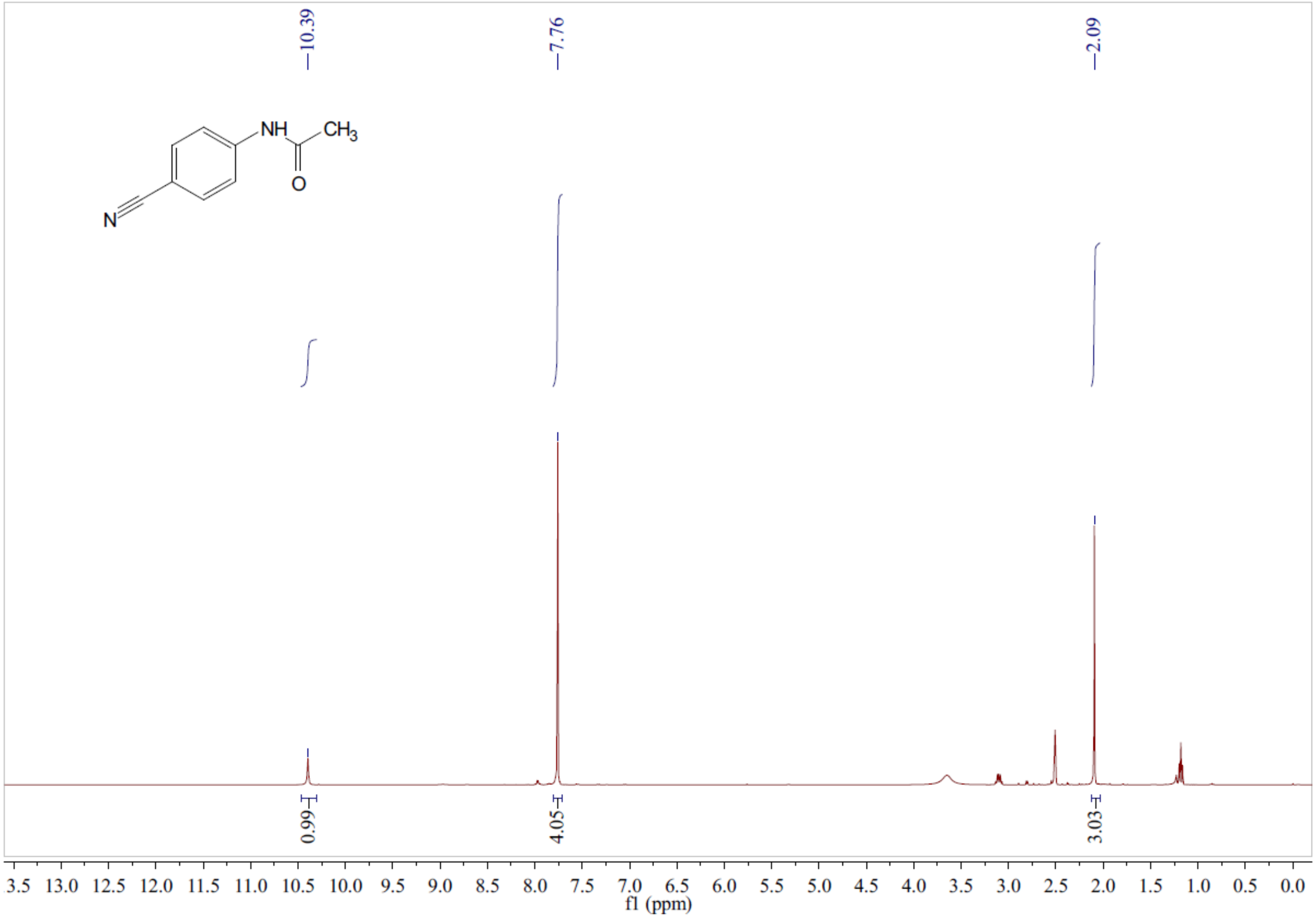

^{13}C NMR Spectra of 4i

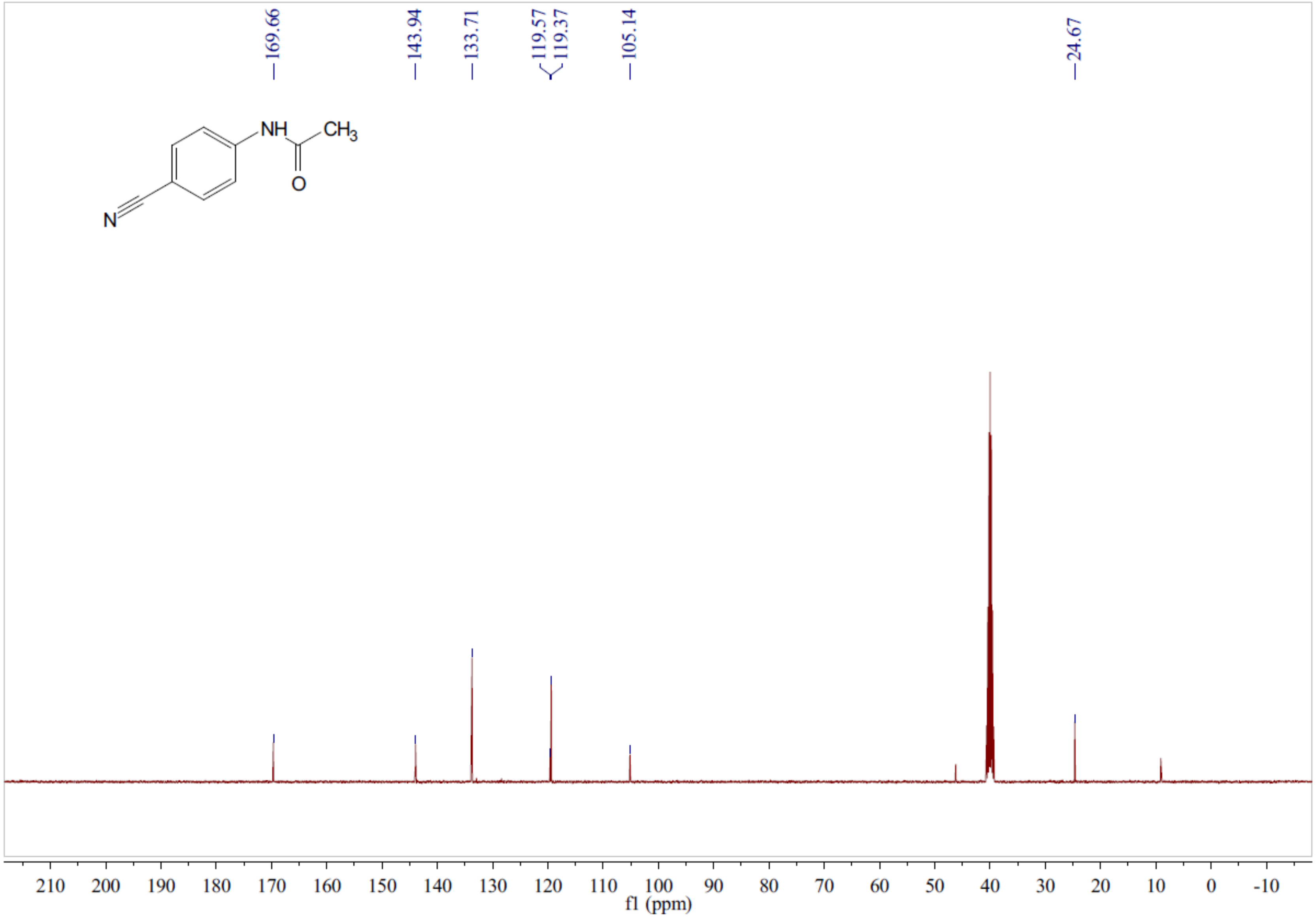

^{1}H NMR Spectra of 4j

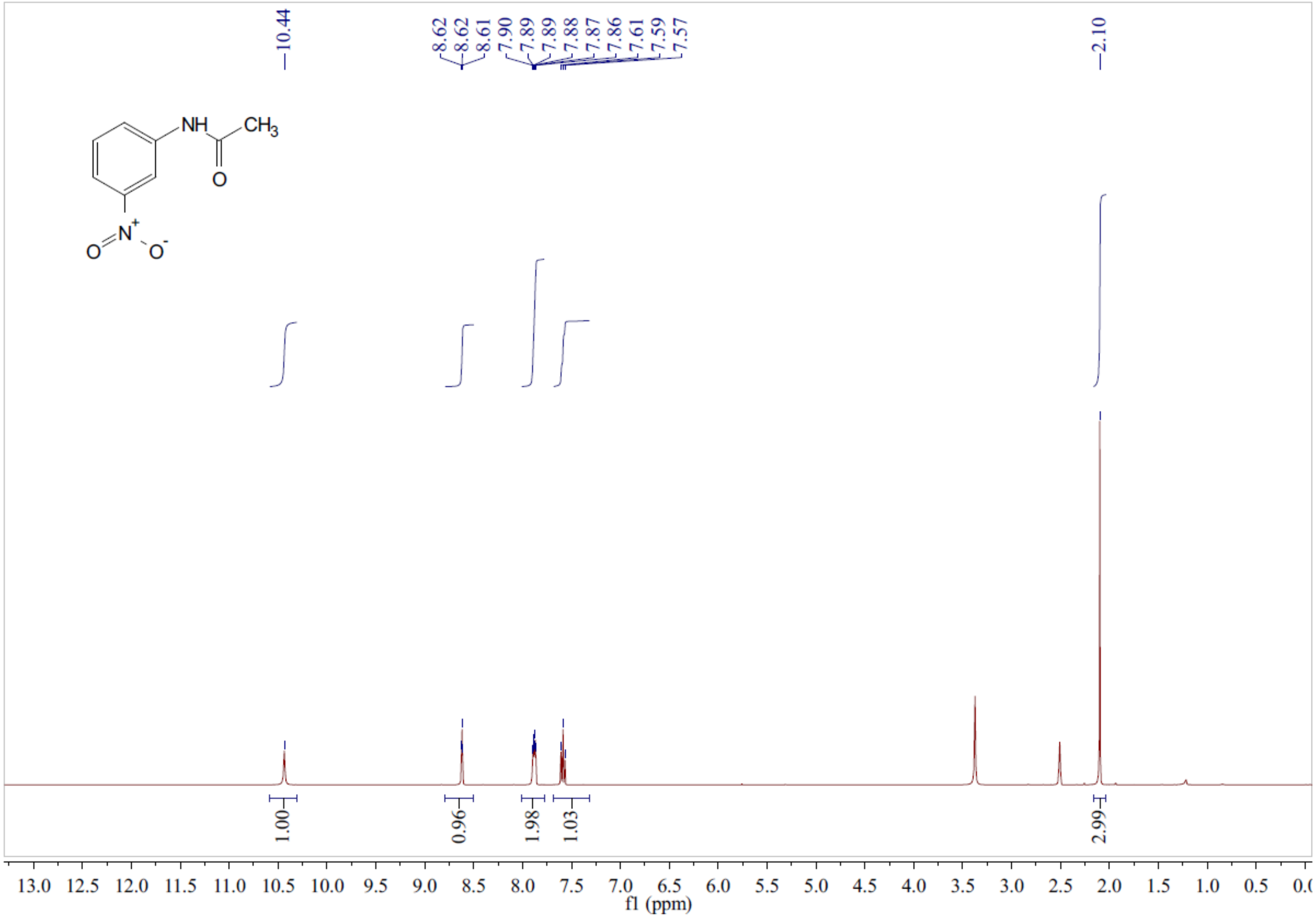

^{13}C NMR Spectra of 4j

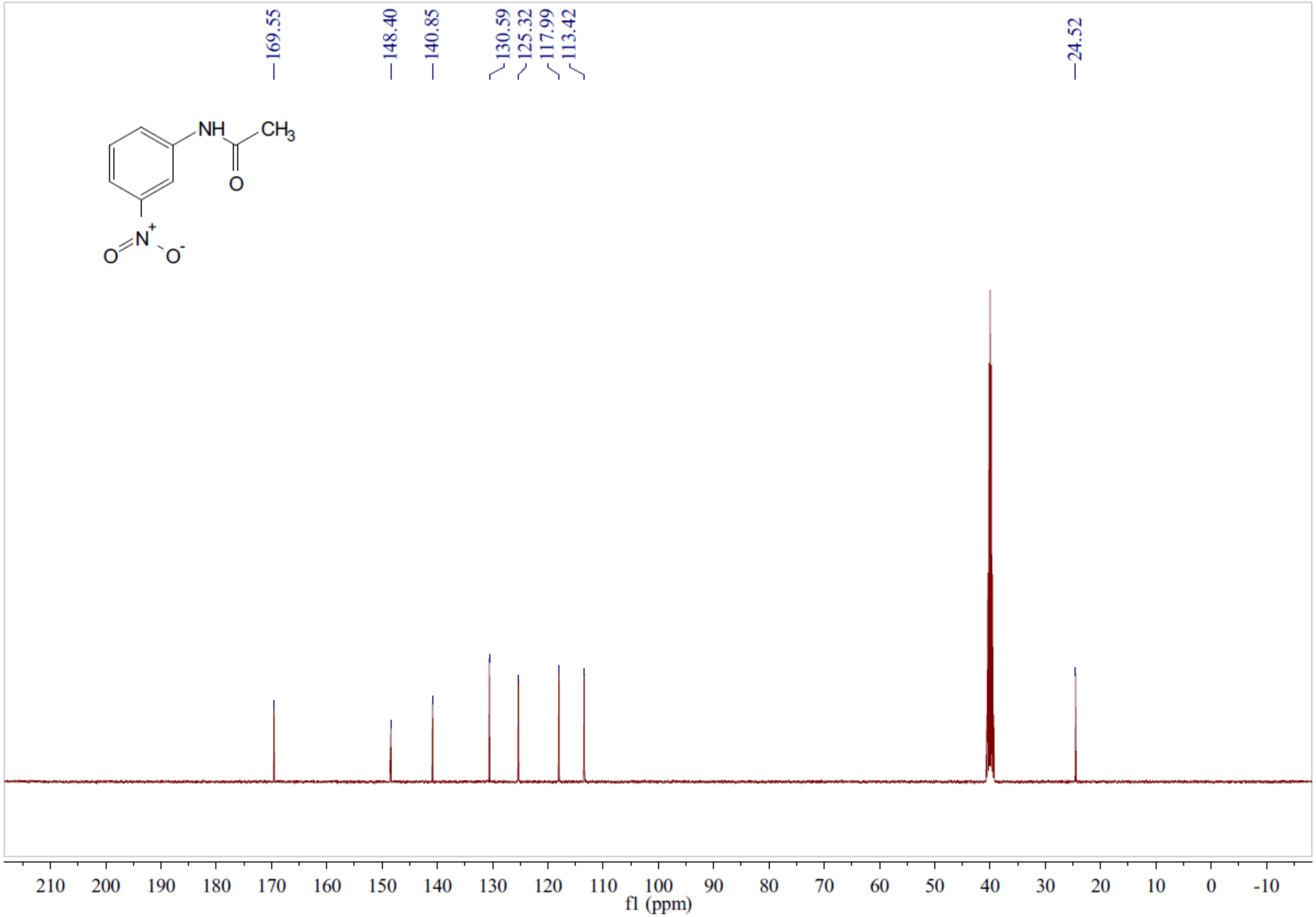

^{1}H NMR Spectra of 4k

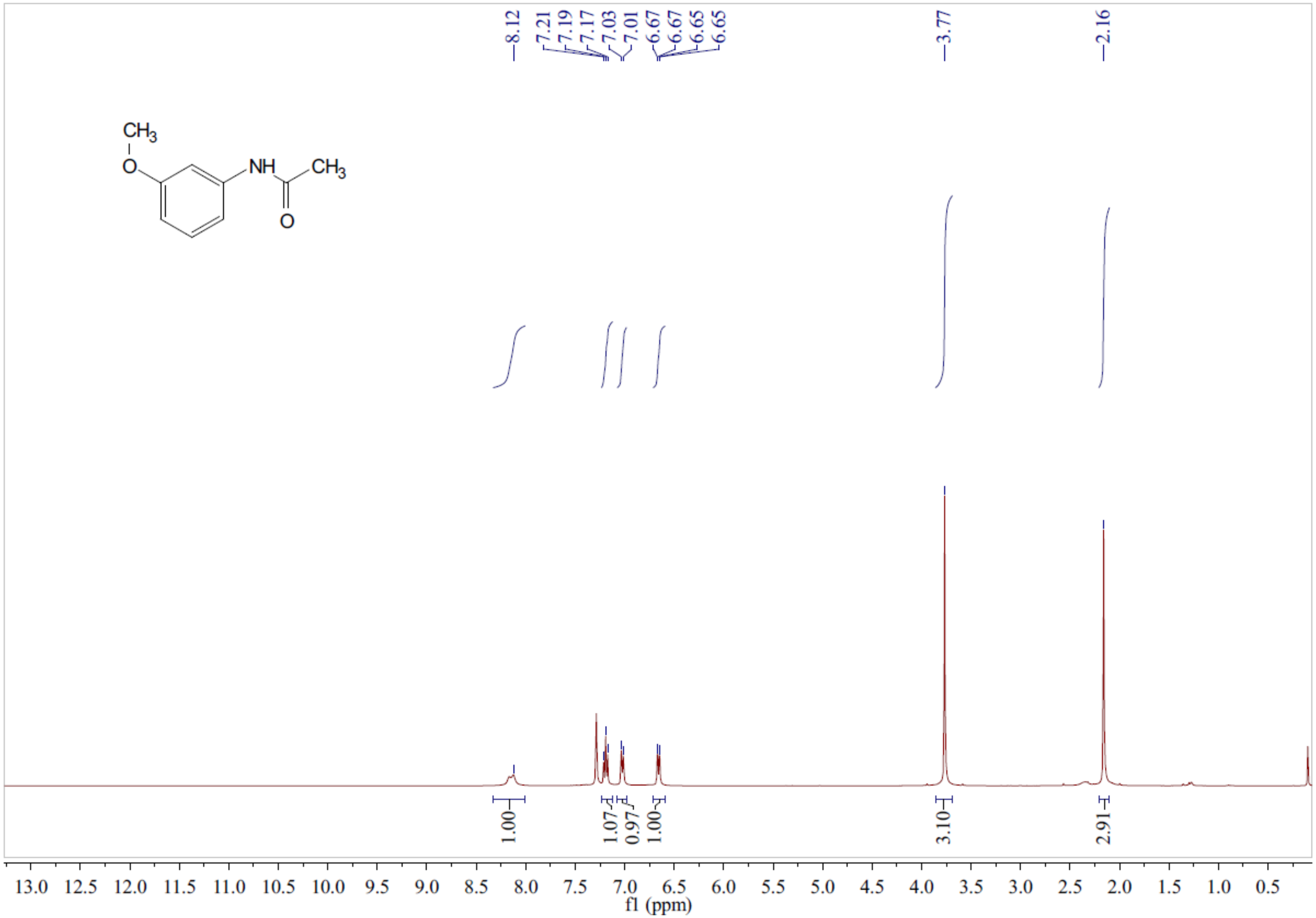

^{13}C NMR Spectra of 4k

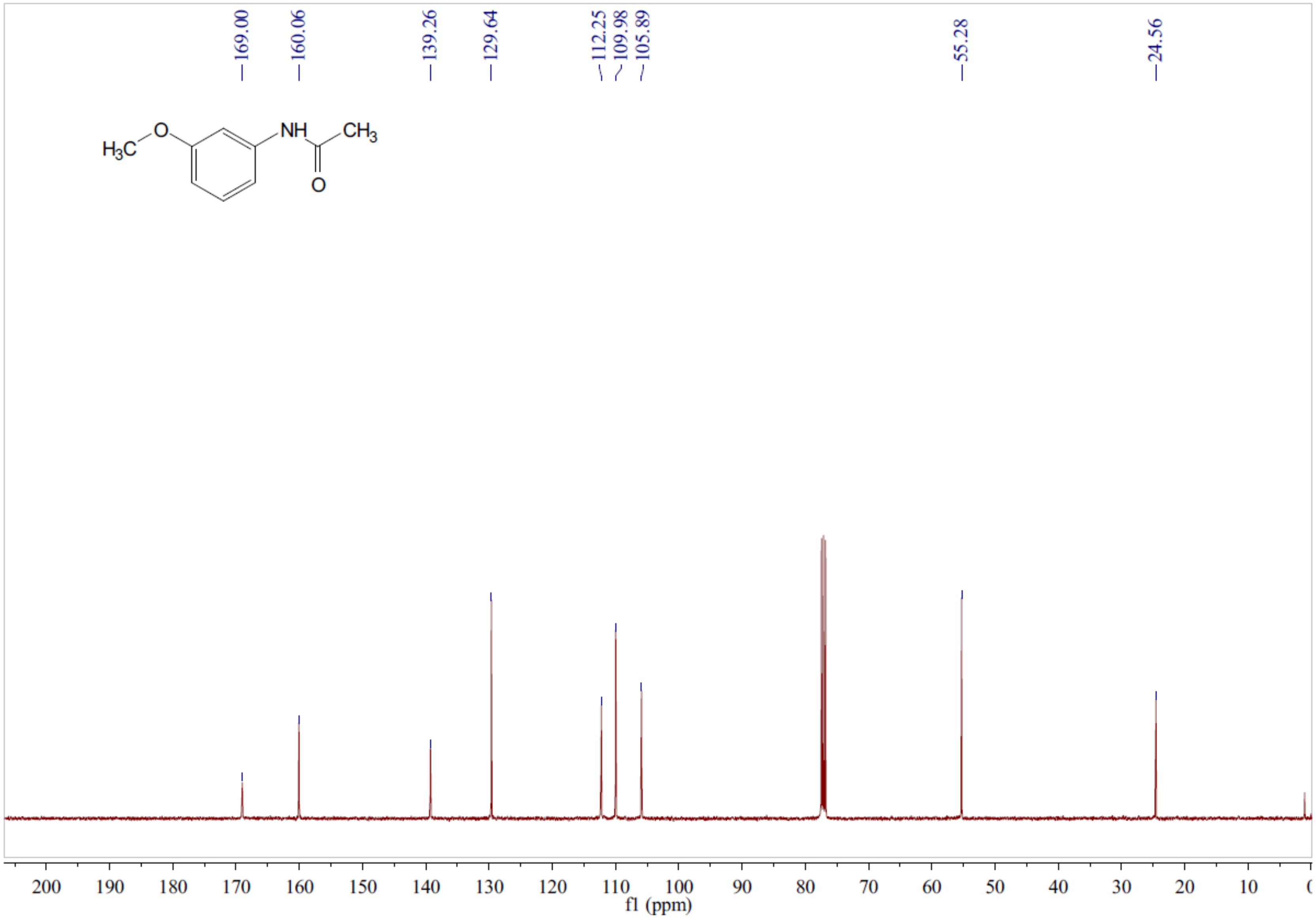

^{1}H NMR Spectra of 4l

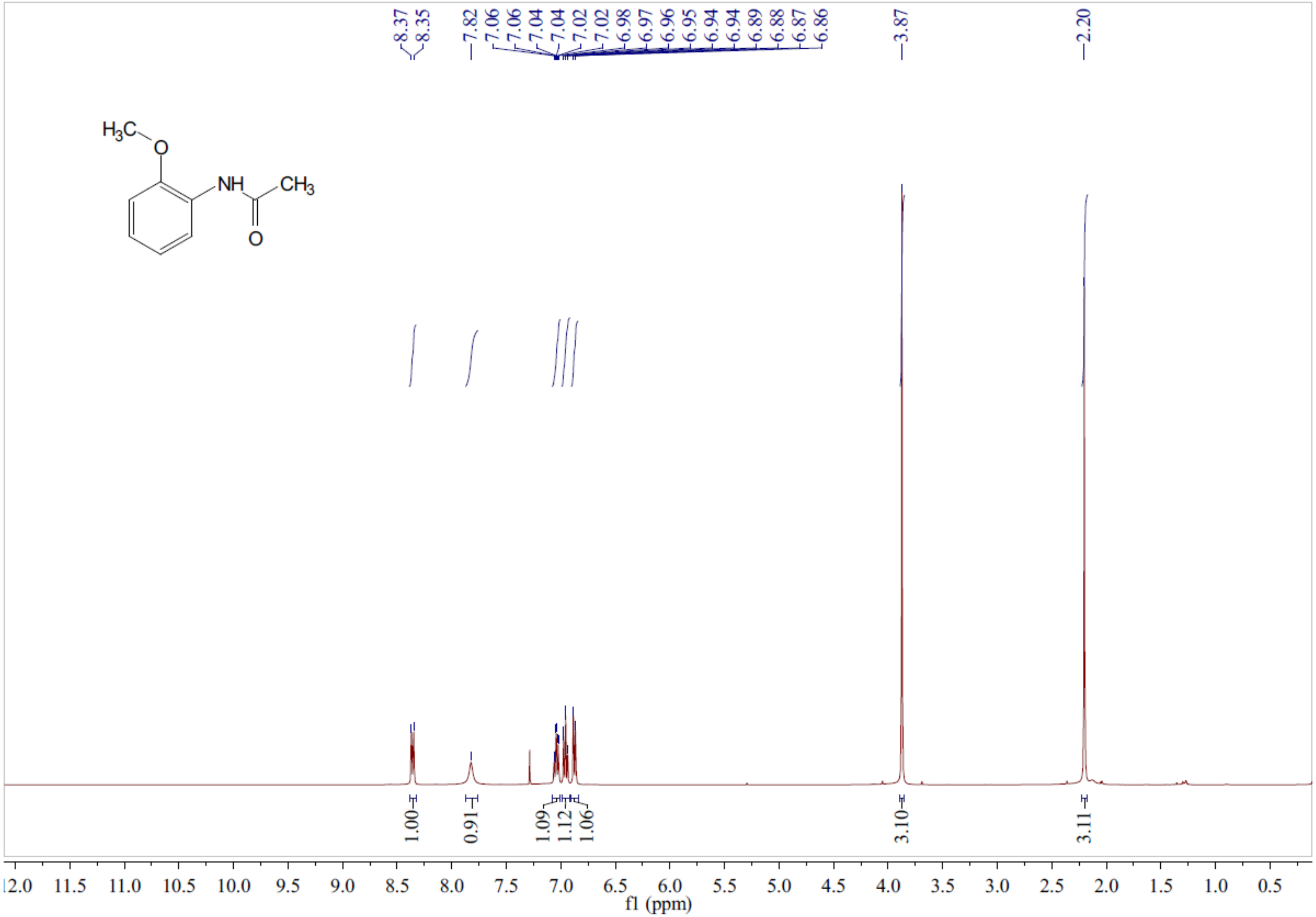

^{13}C NMR Spectra of 4l

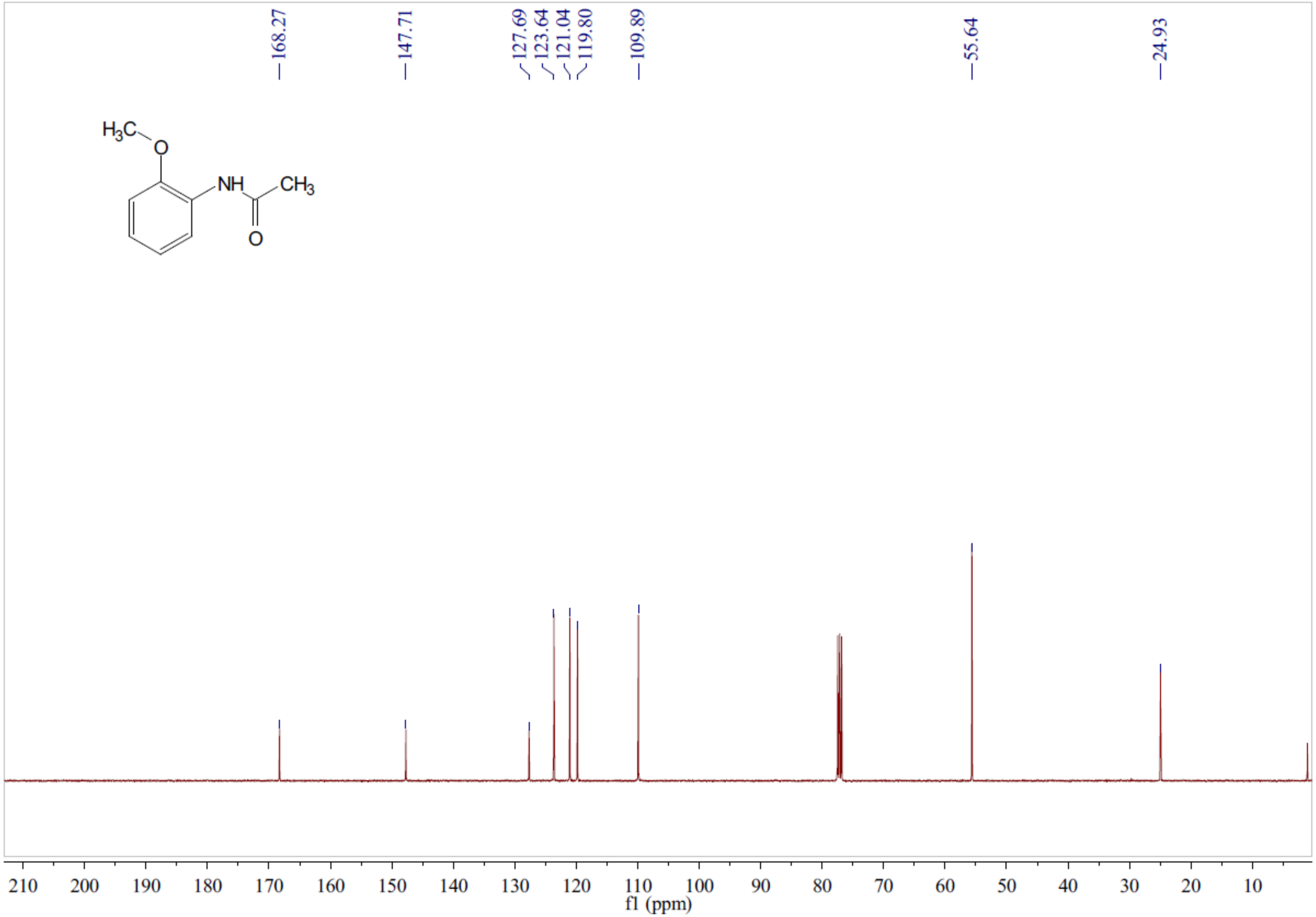

^{1}H NMR Spectra of 4m

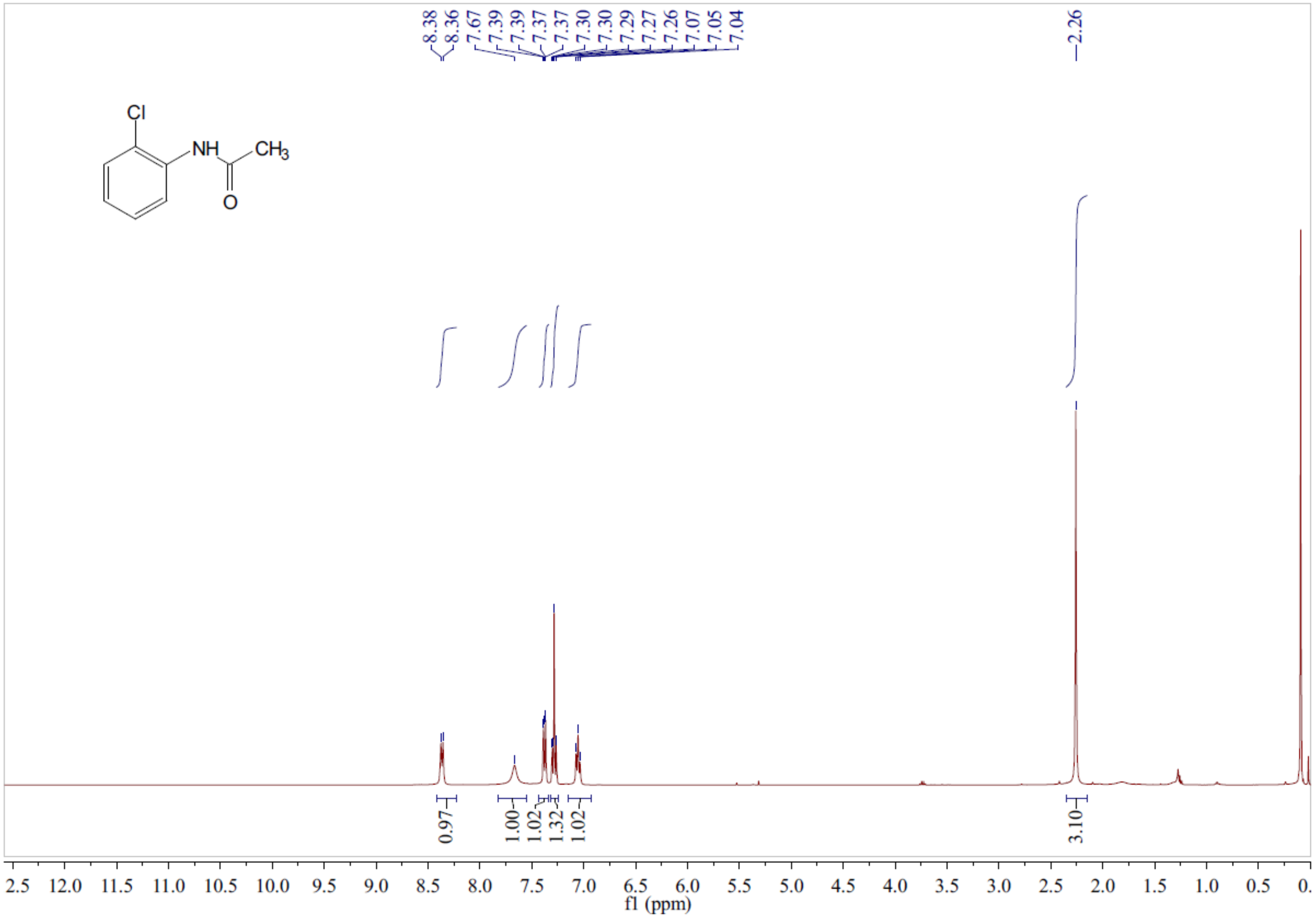

^{13}C NMR Spectra of 4m

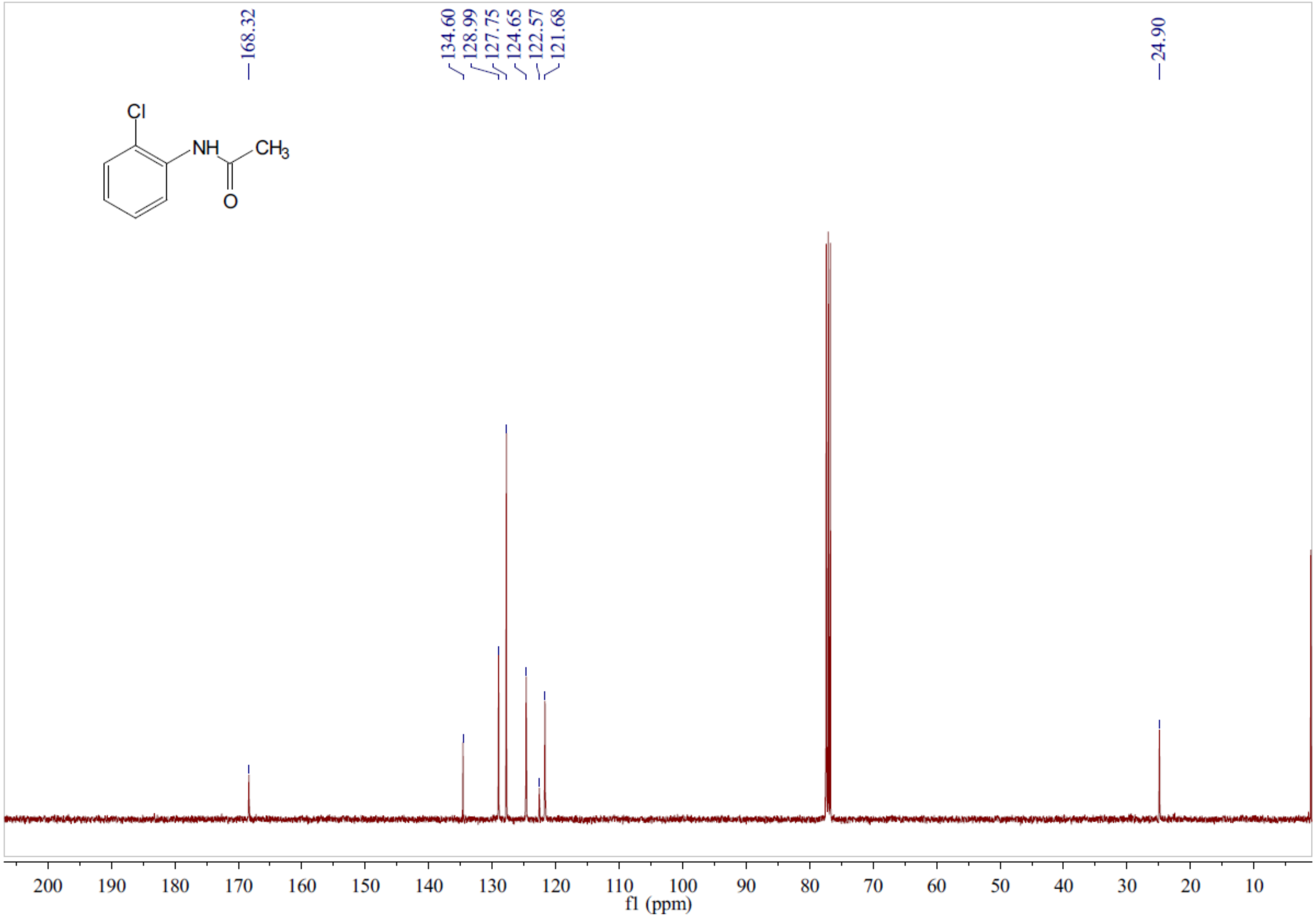

¹H NMR Spectra of 4n

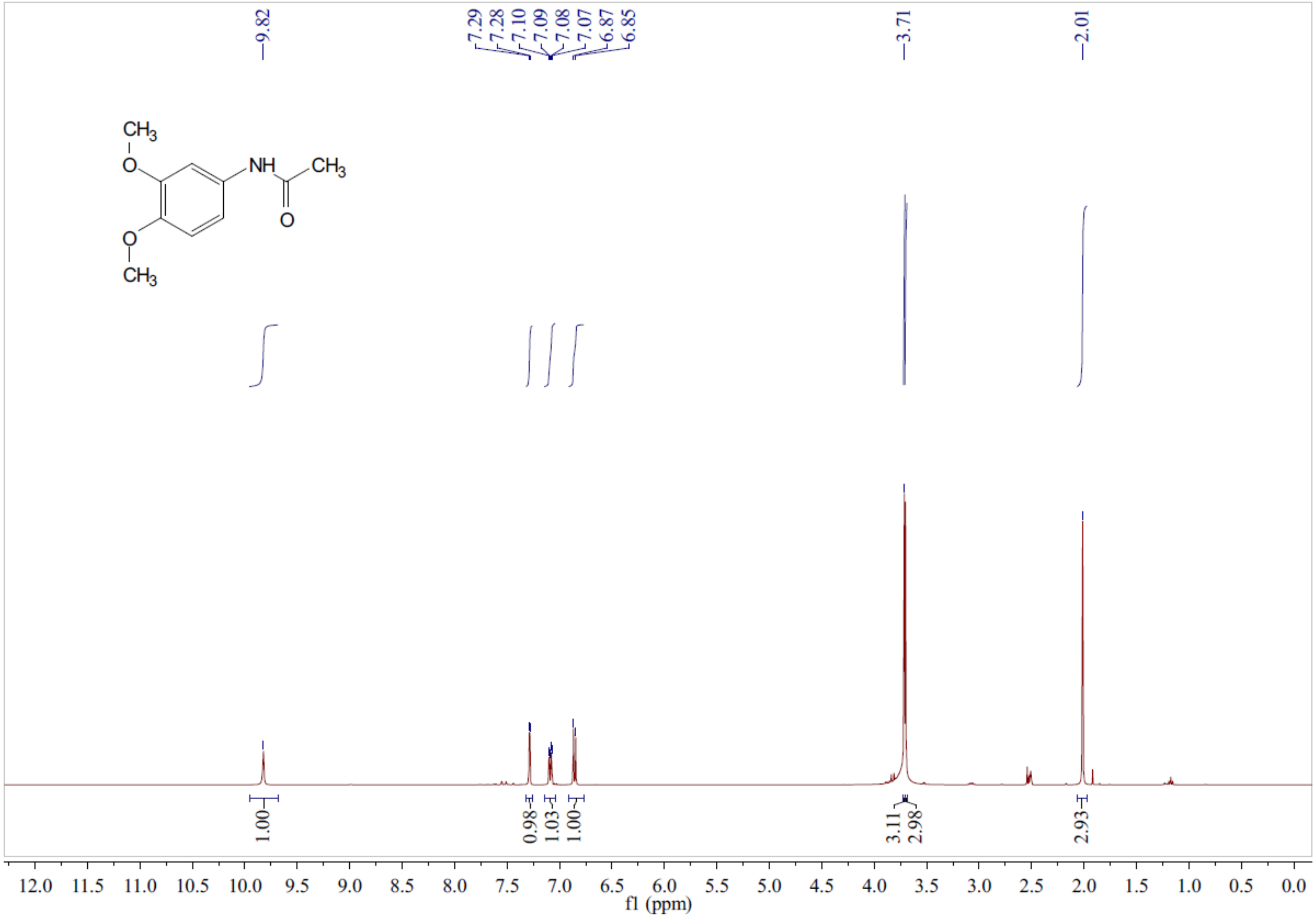

^{13}C NMR Spectra of 4n

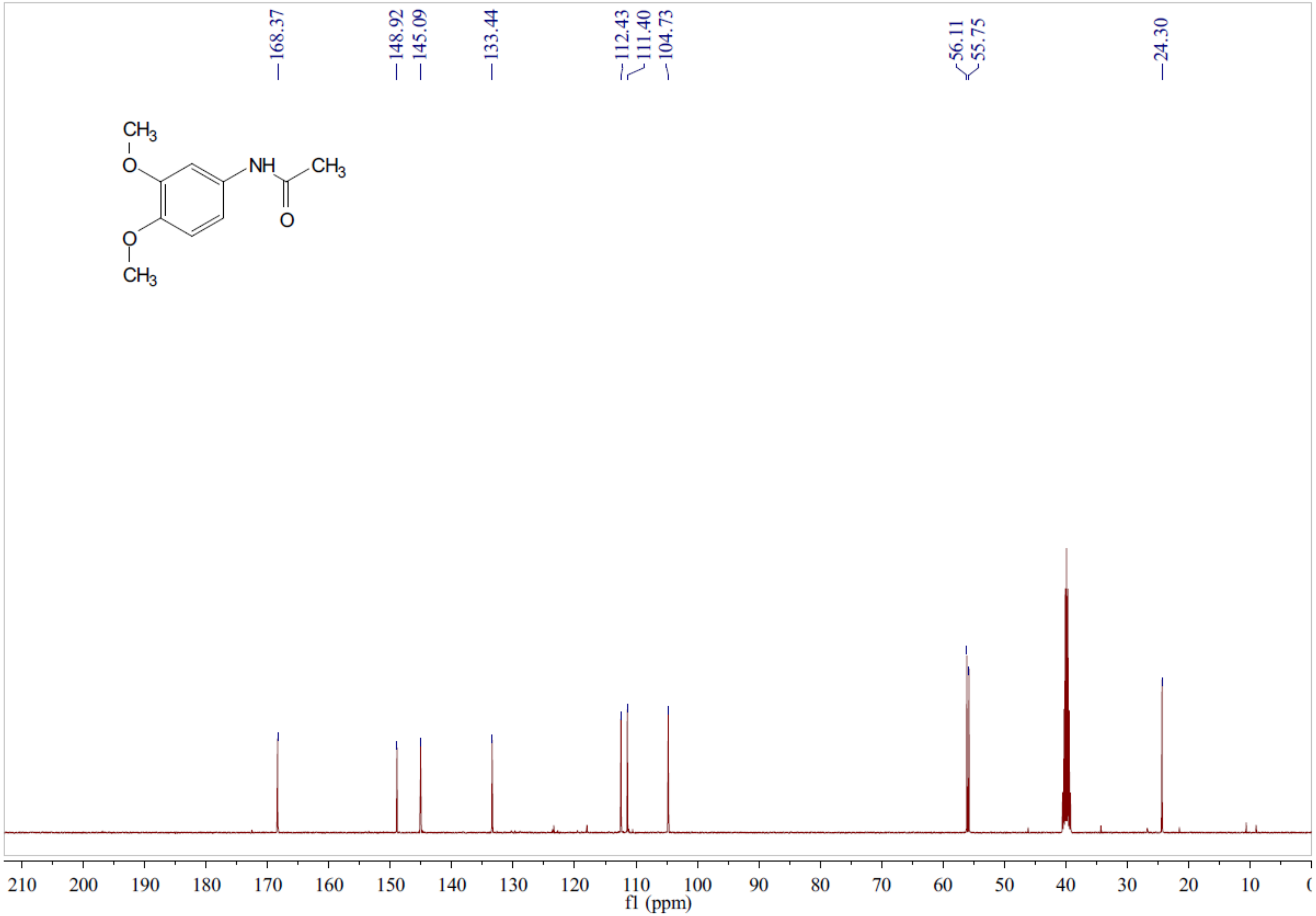

^{1}H NMR Spectra of 4o

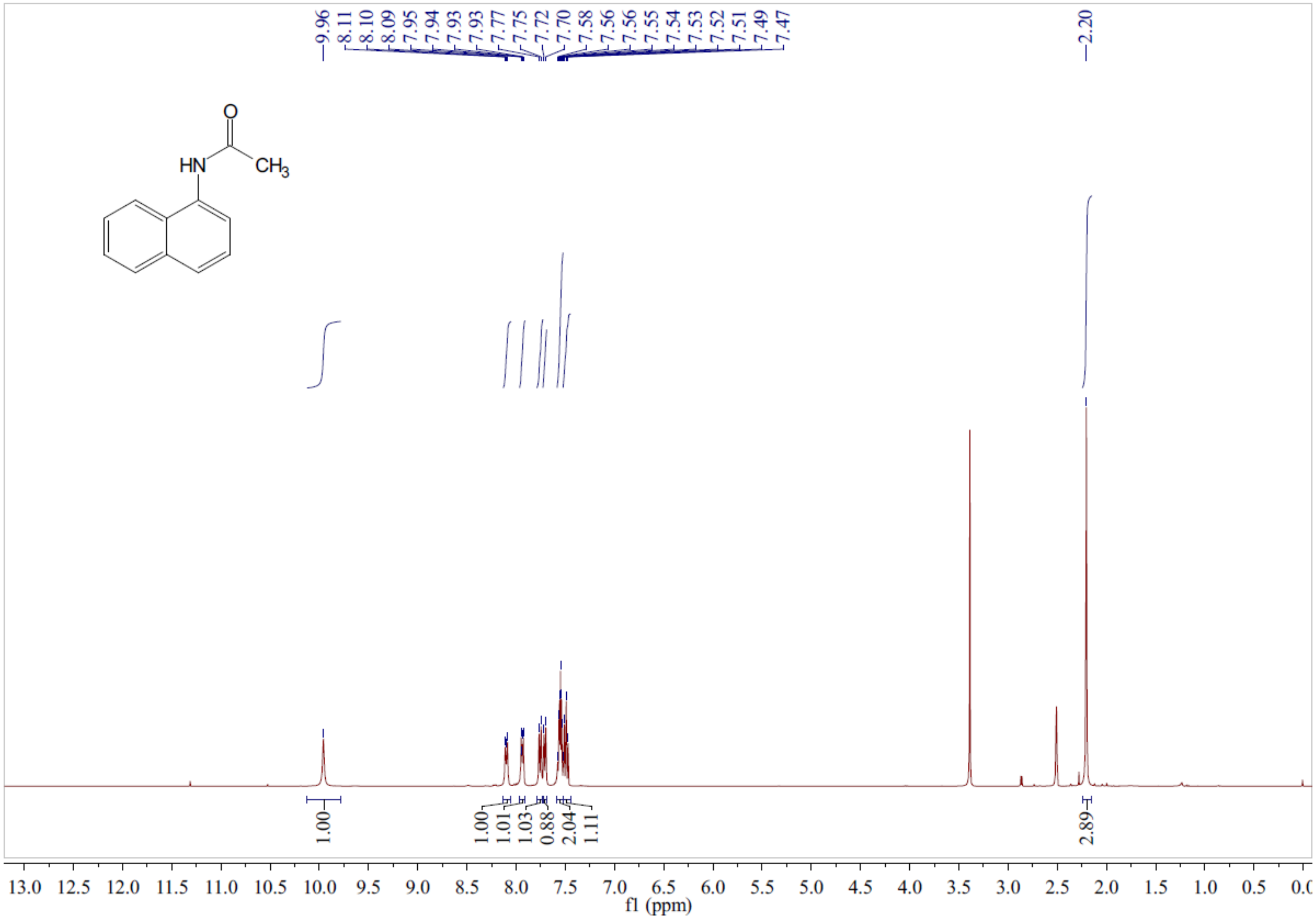

^{13}C NMR Spectra of 4o

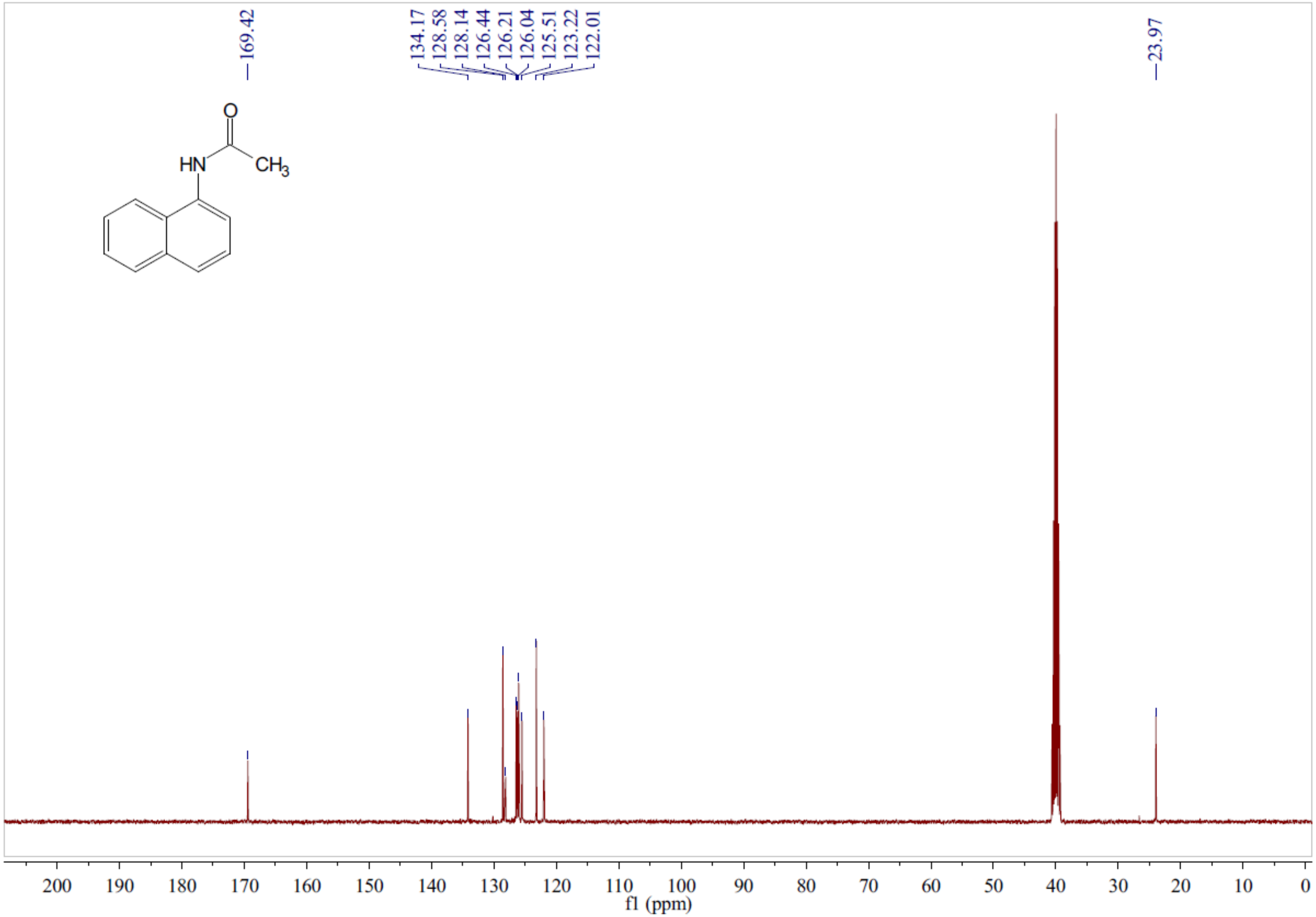

^{1}H NMR Spectra of 4p

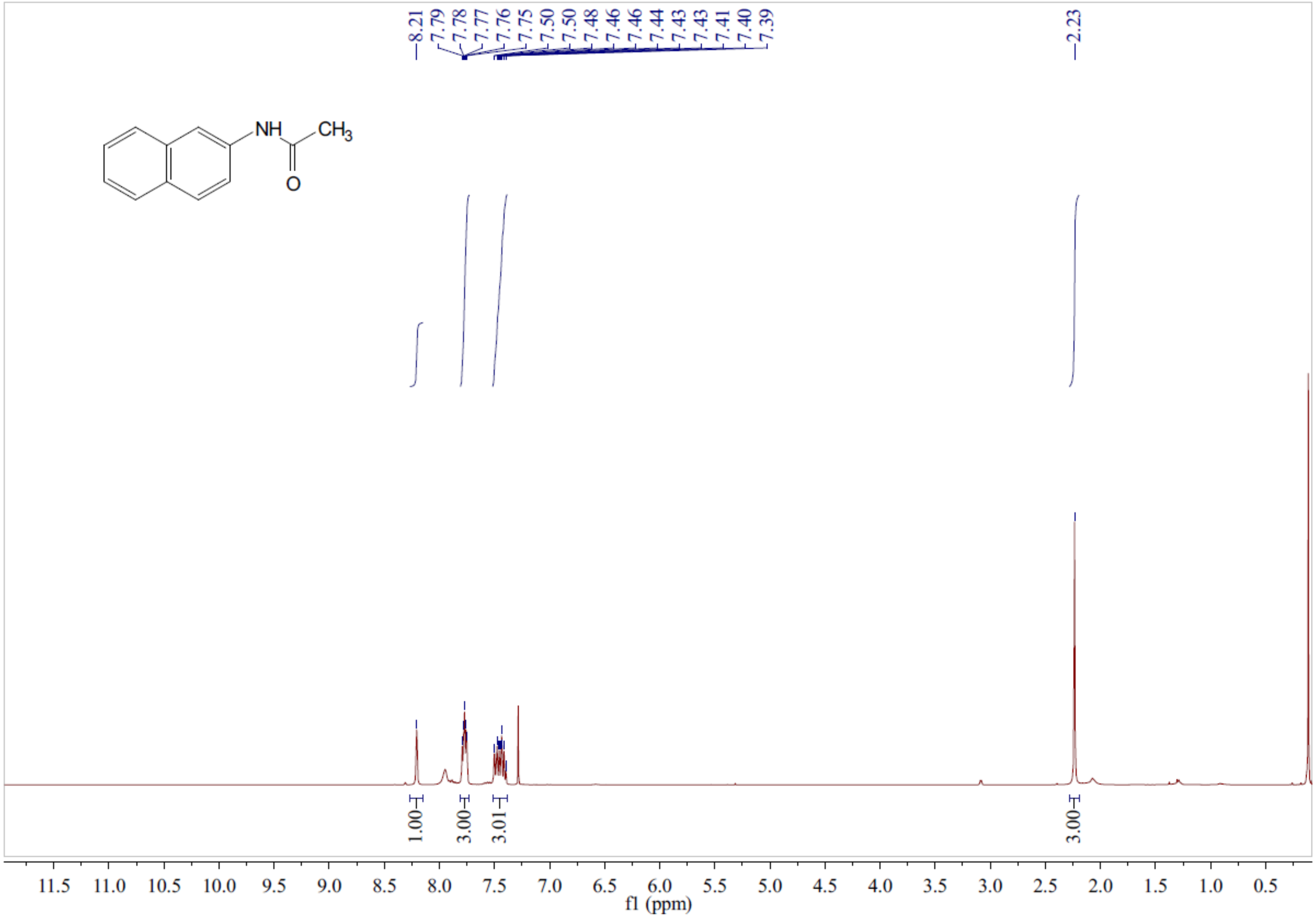

^{13}C NMR Spectra of 4p

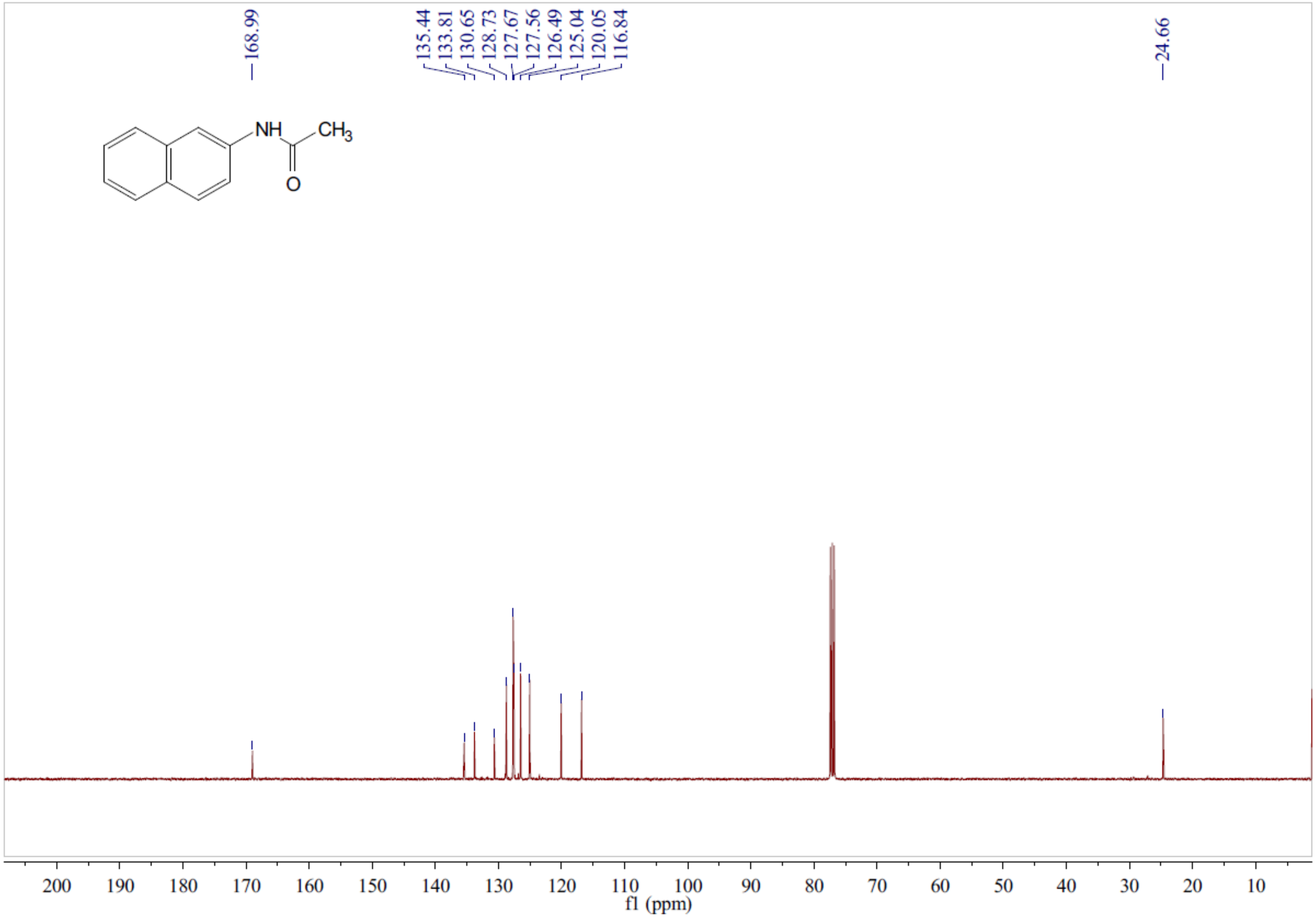

^{1}H NMR Spectra of 4q

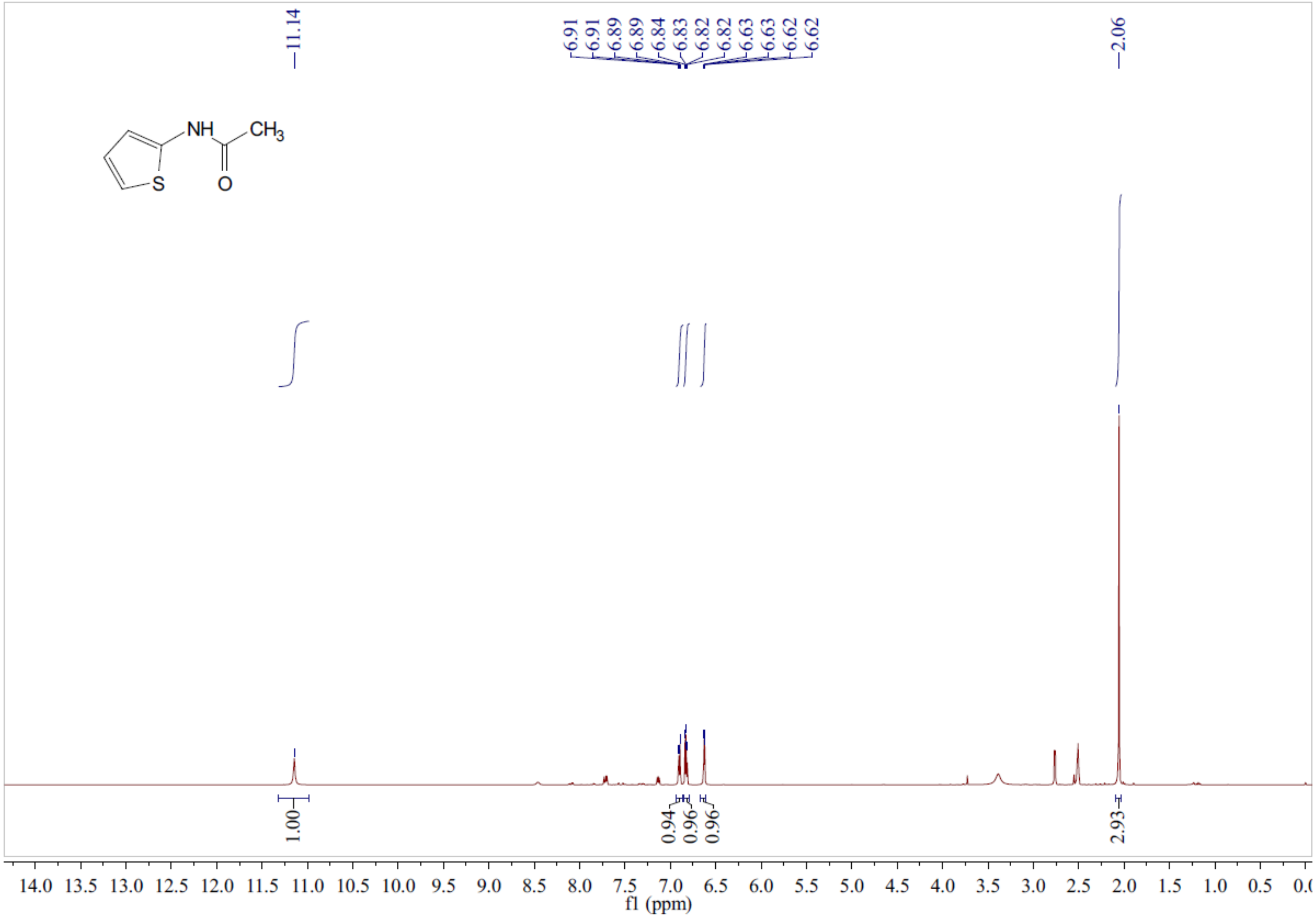

^{13}C NMR Spectra of 4q

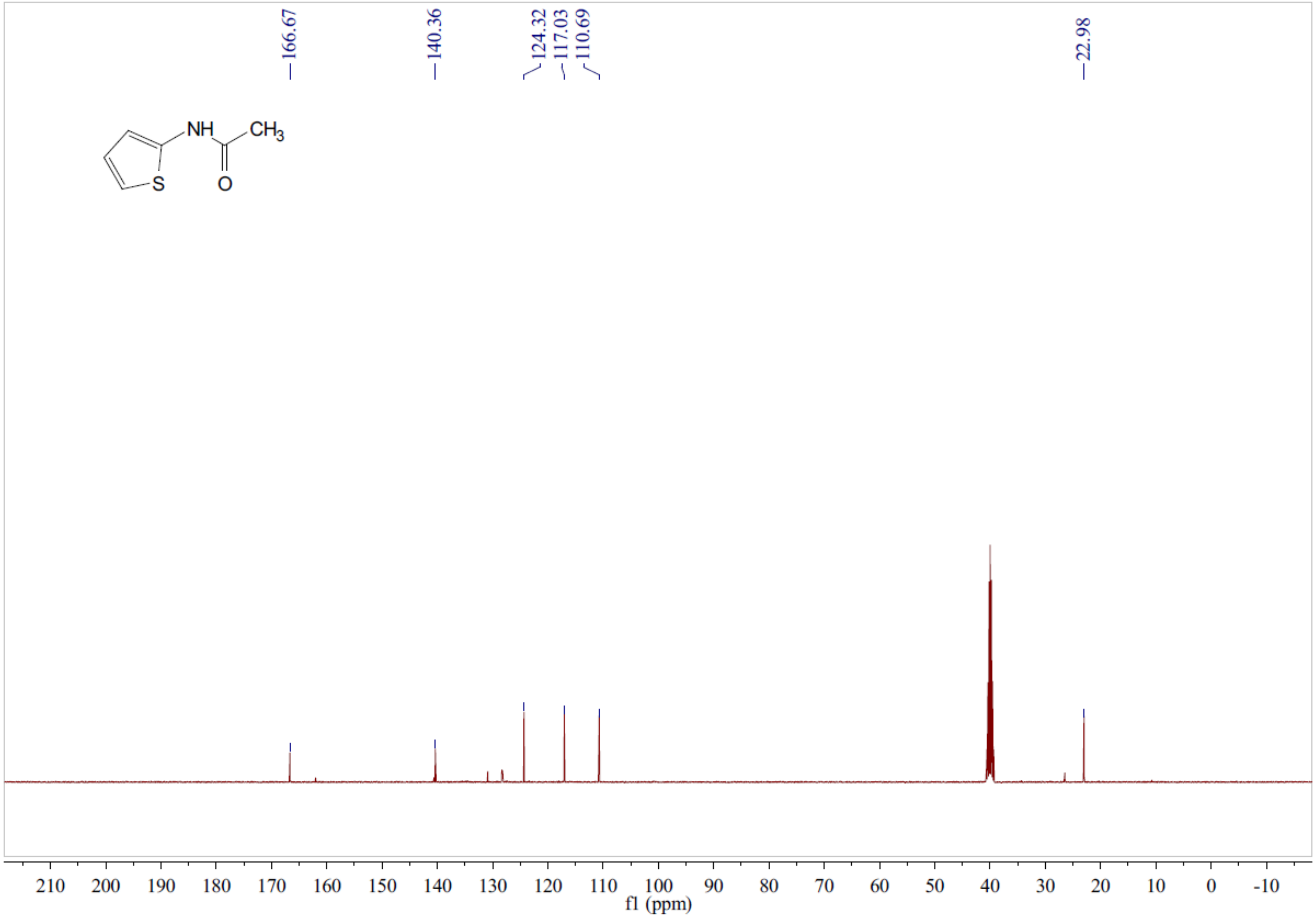

^{1}H NMR Spectra of 4r

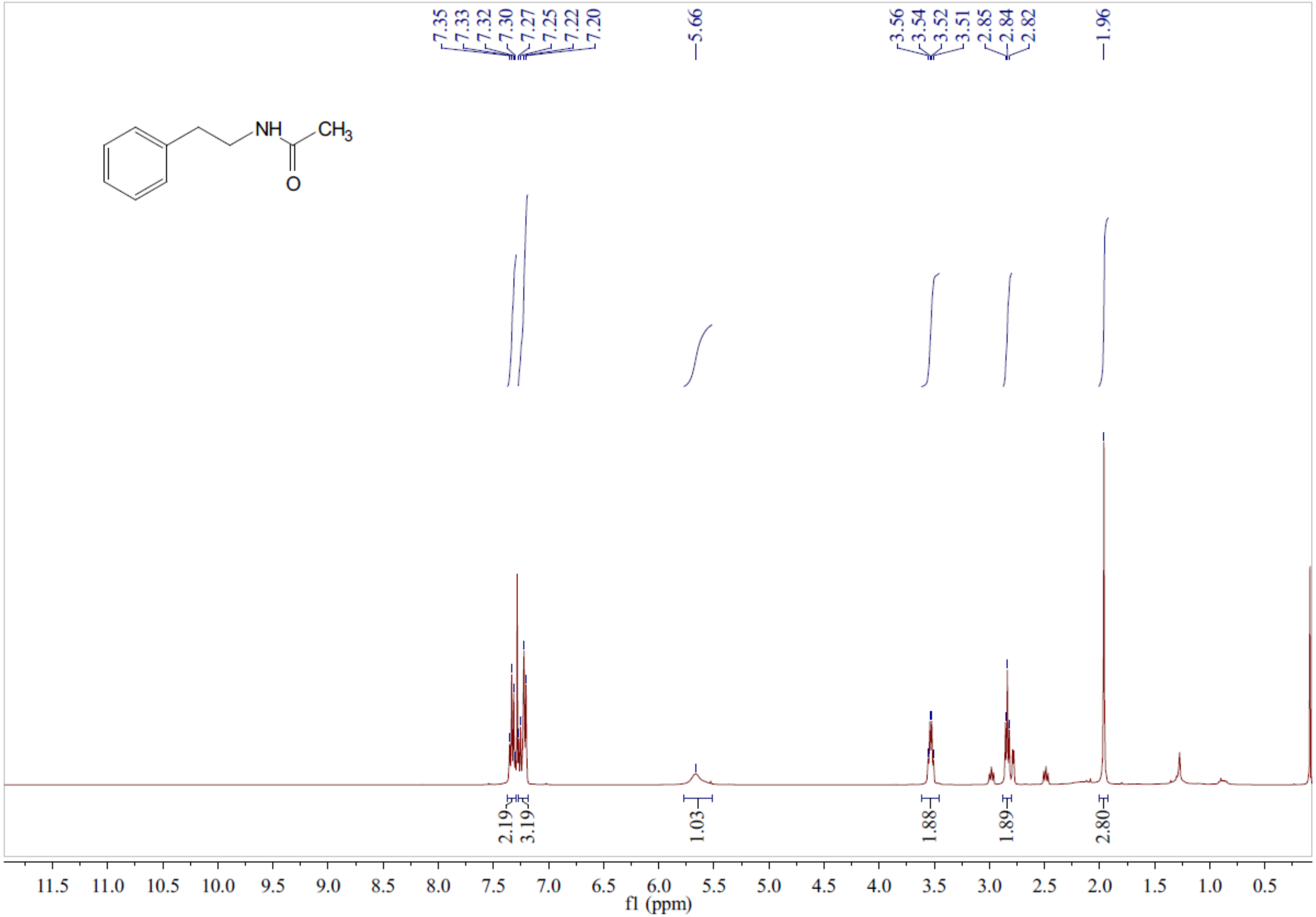

^{13}C NMR Spectra of 4r

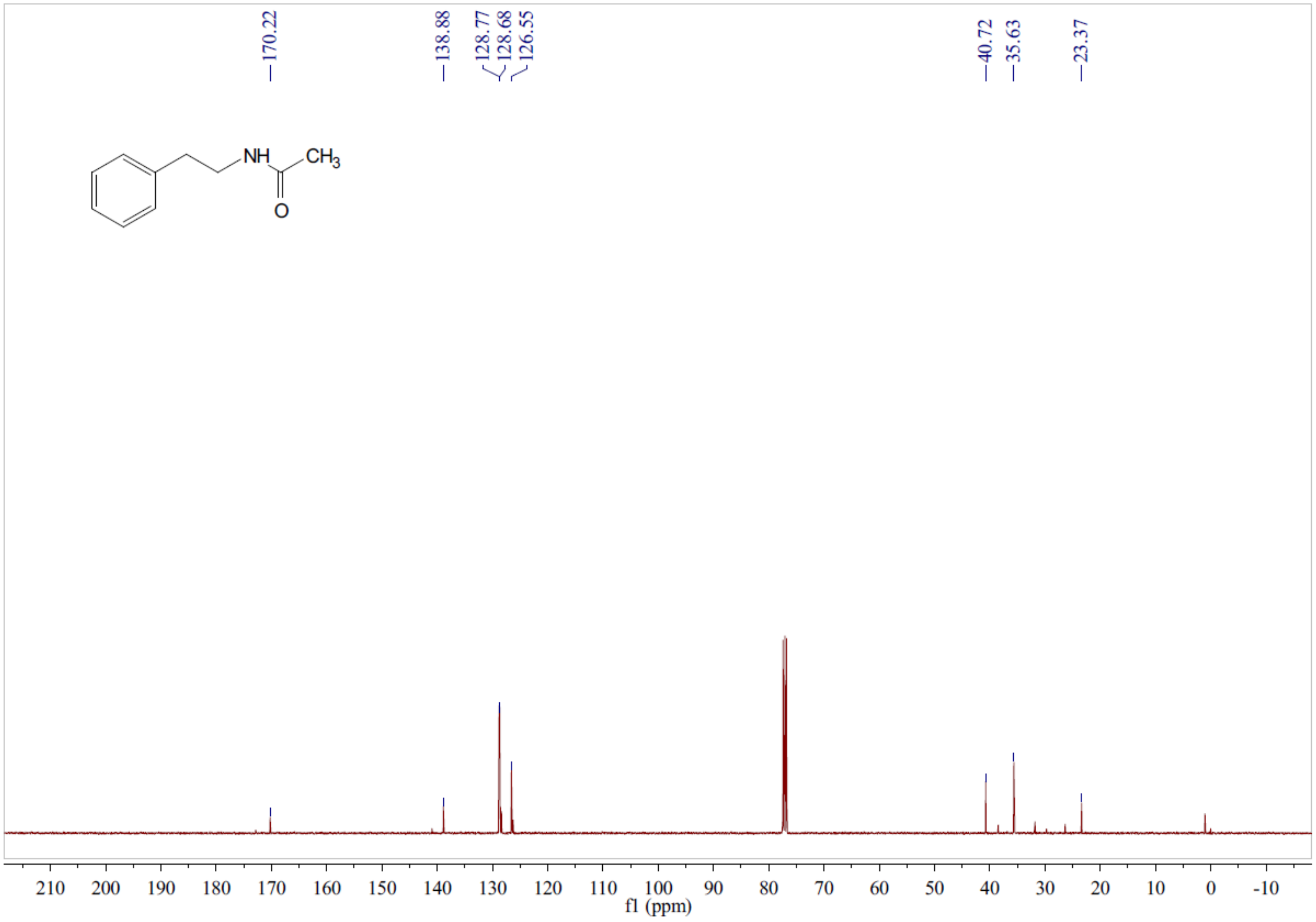

^{1}H NMR Spectra of 4s

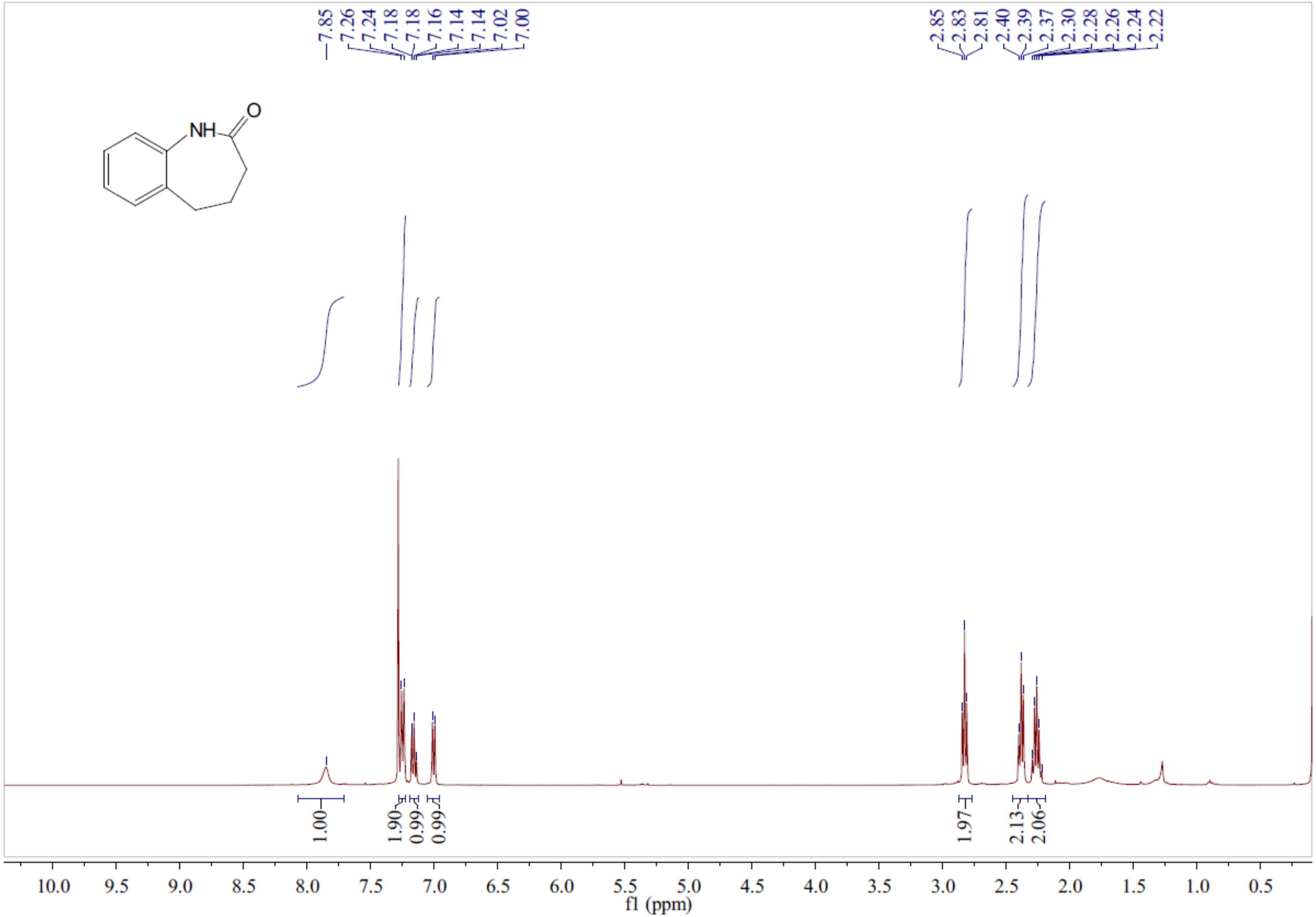

^{13}C NMR Spectra of 4s

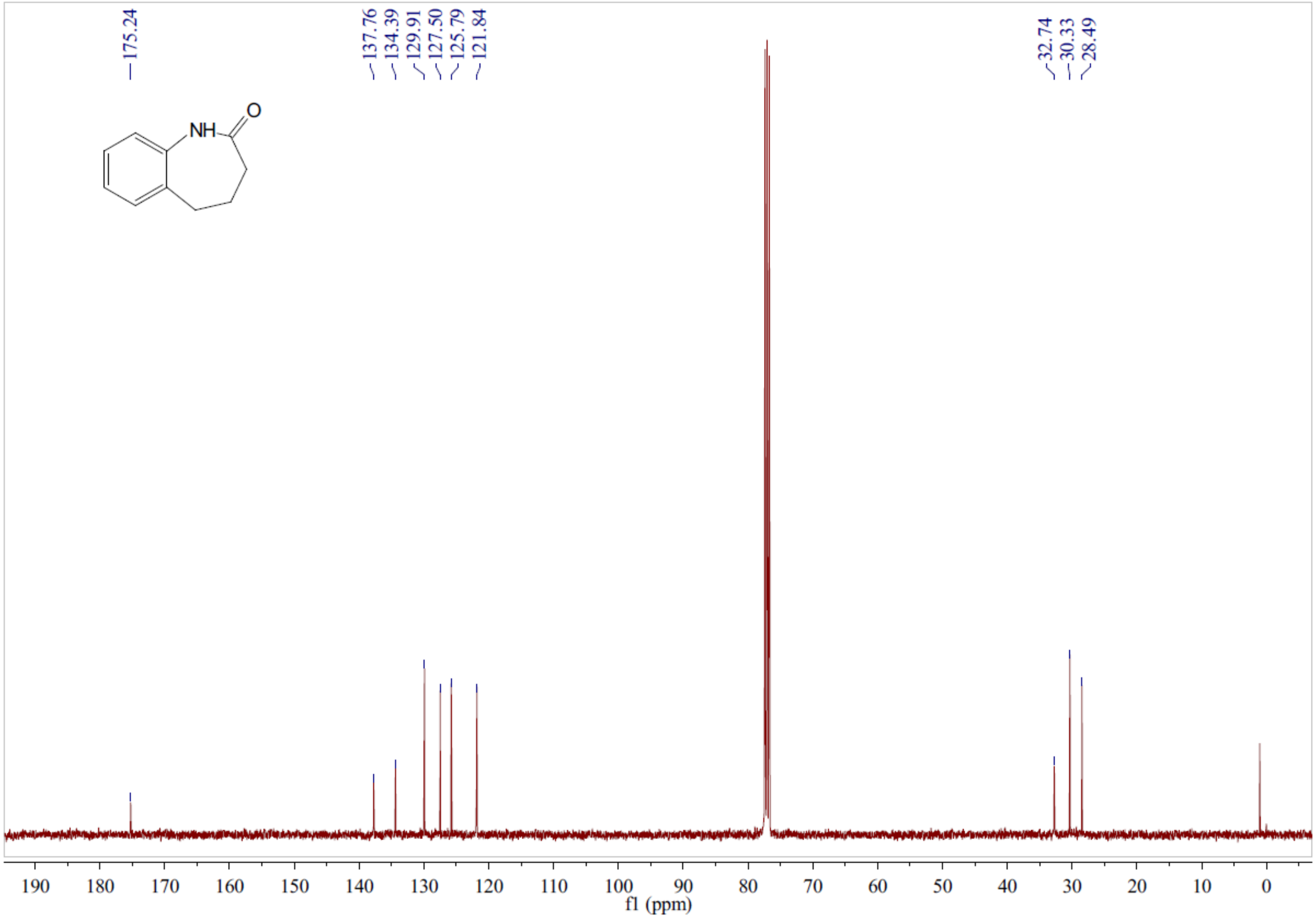

^{1}H NMR Spectra of 4t

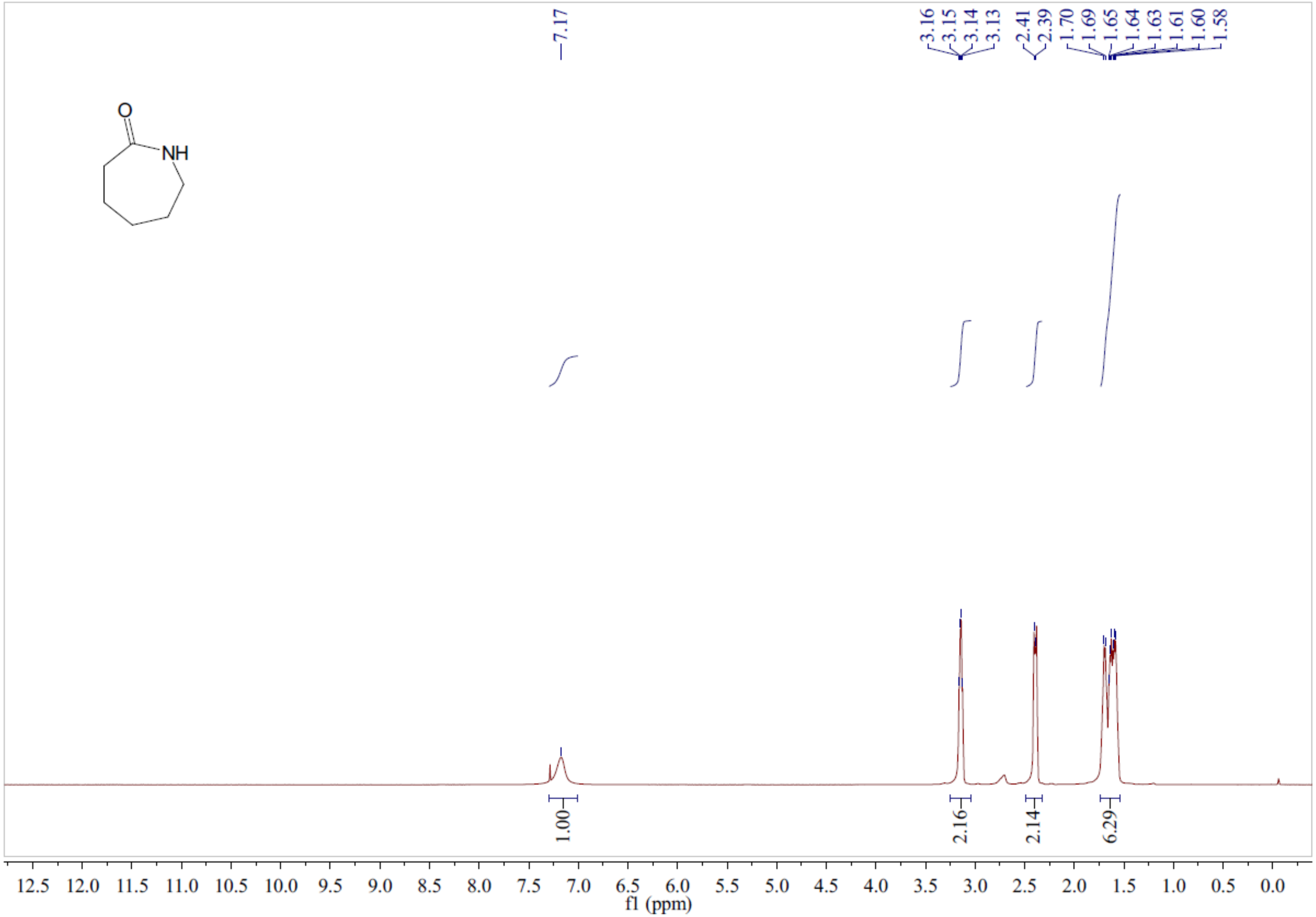

^{13}C NMR Spectra of 4t

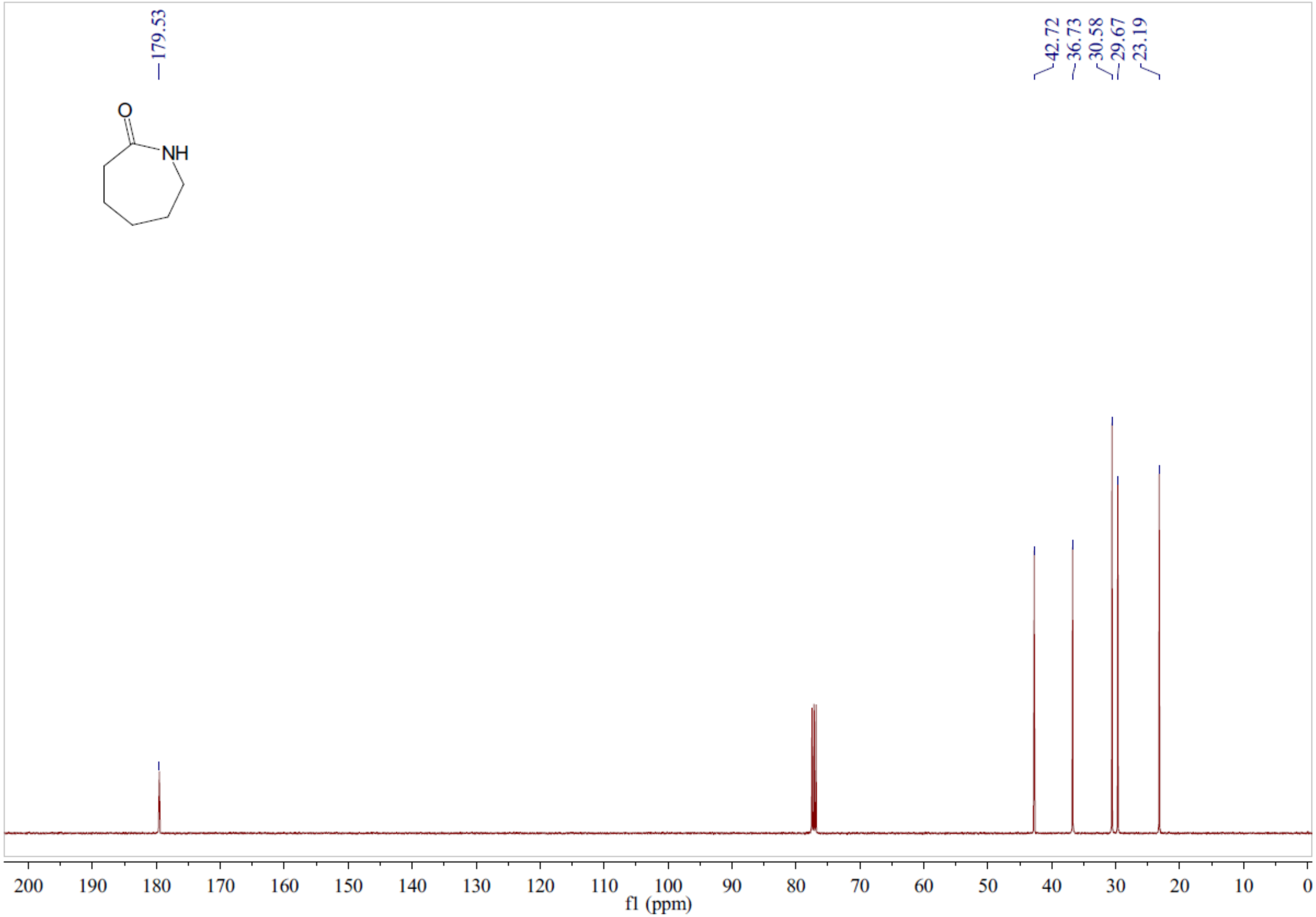

^{1}H NMR Spectra of E

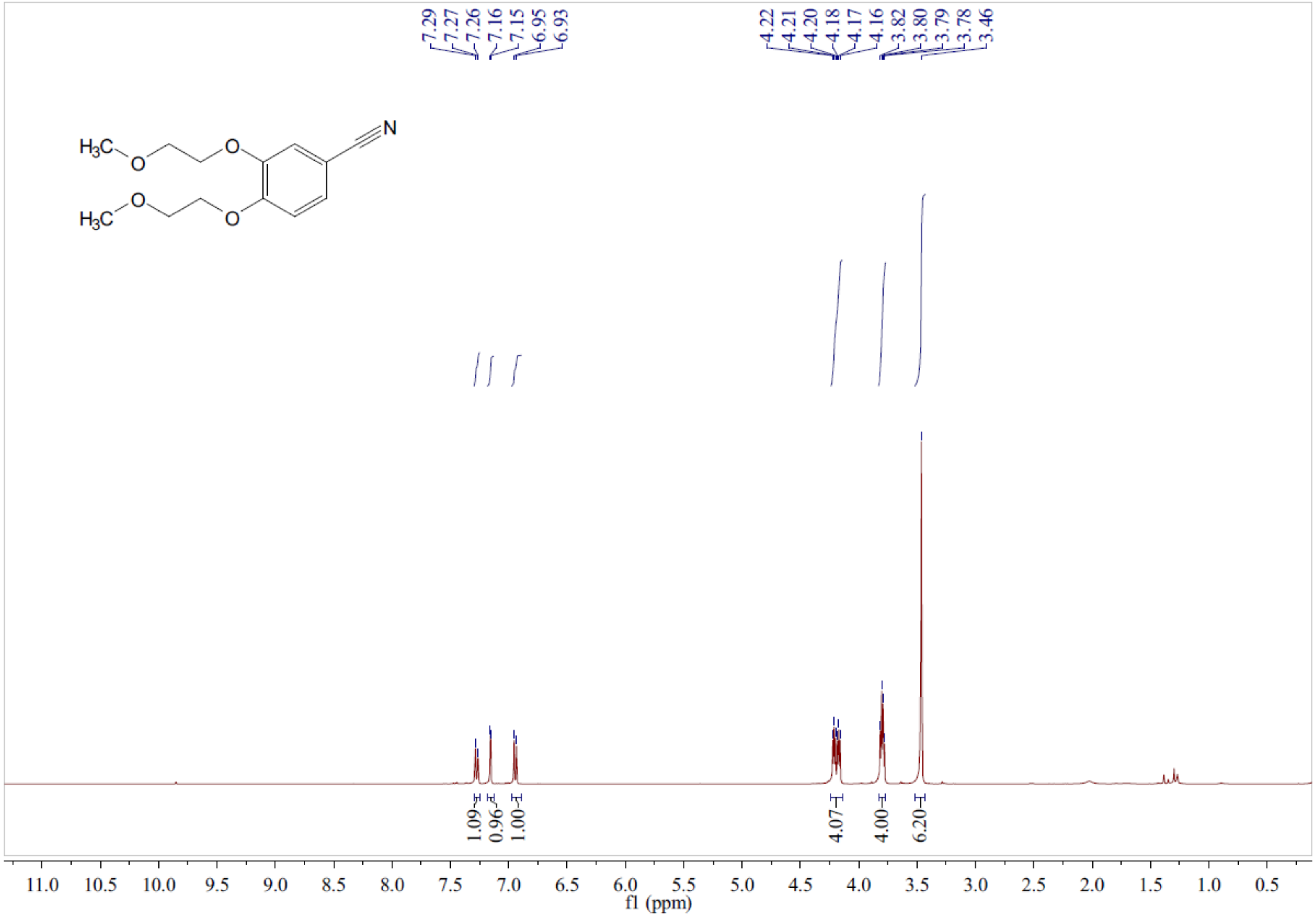

^{13}C NMR Spectra of E

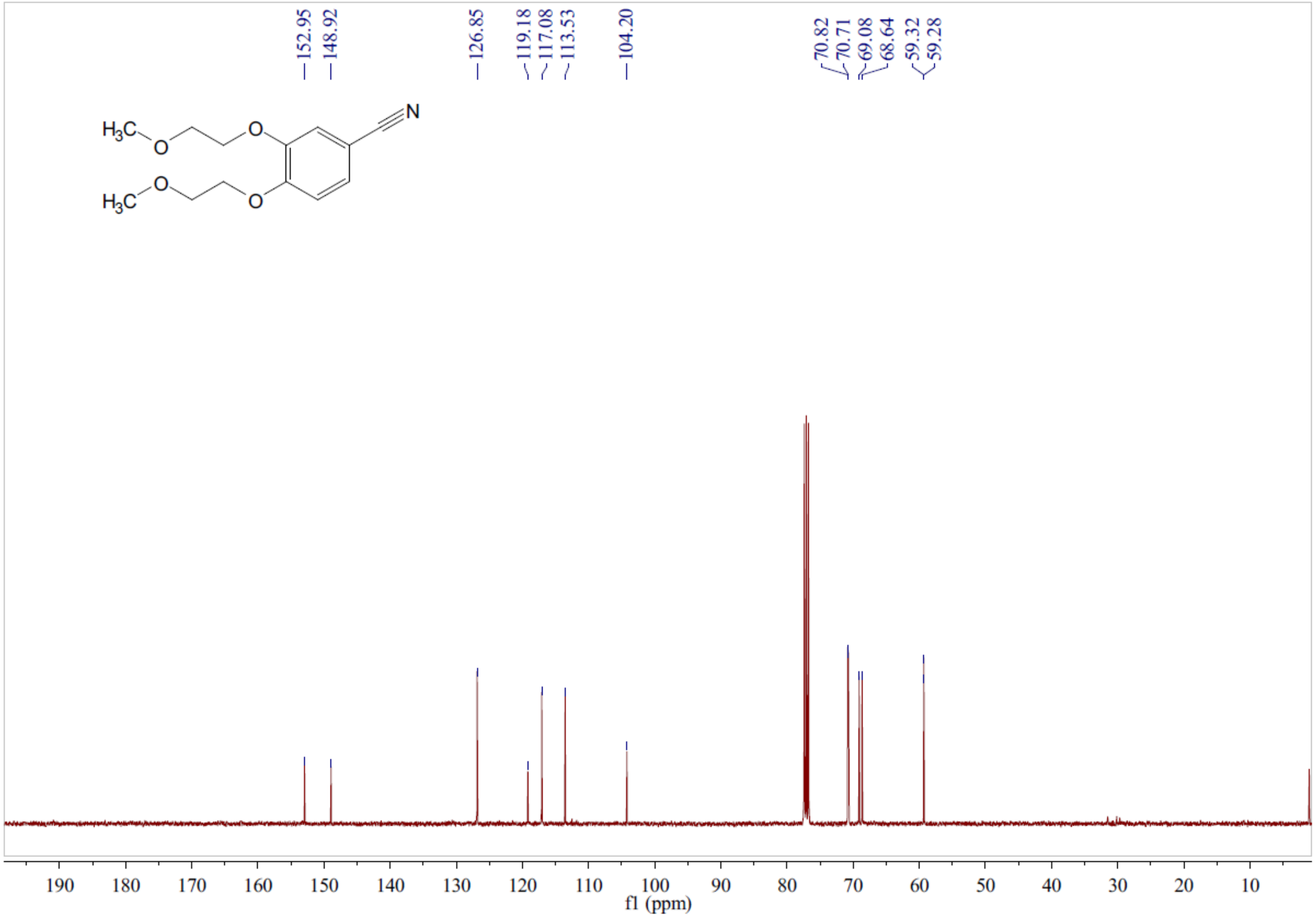

^{1}H NMR Spectra of G

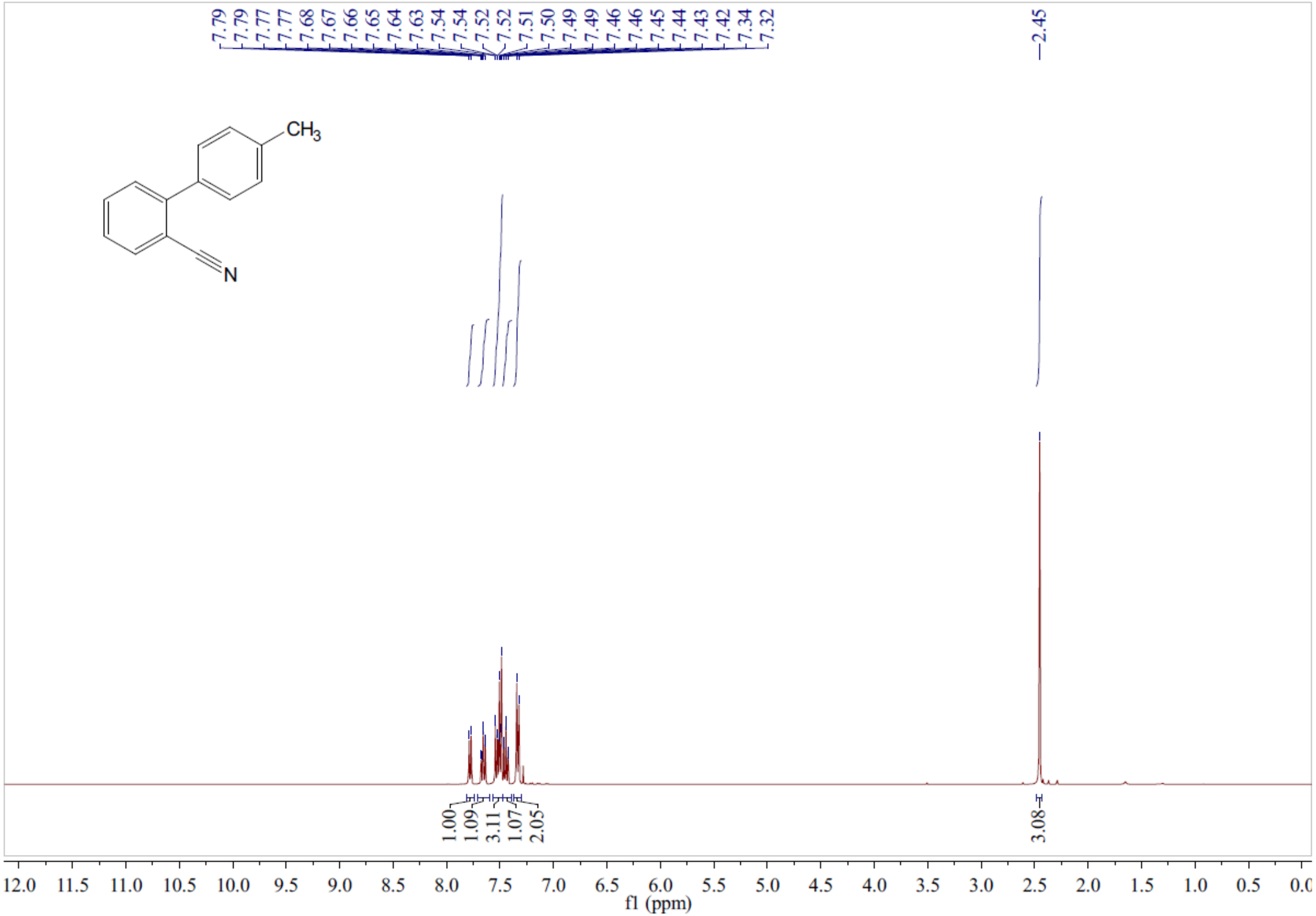

^{13}C NMR Spectra of G

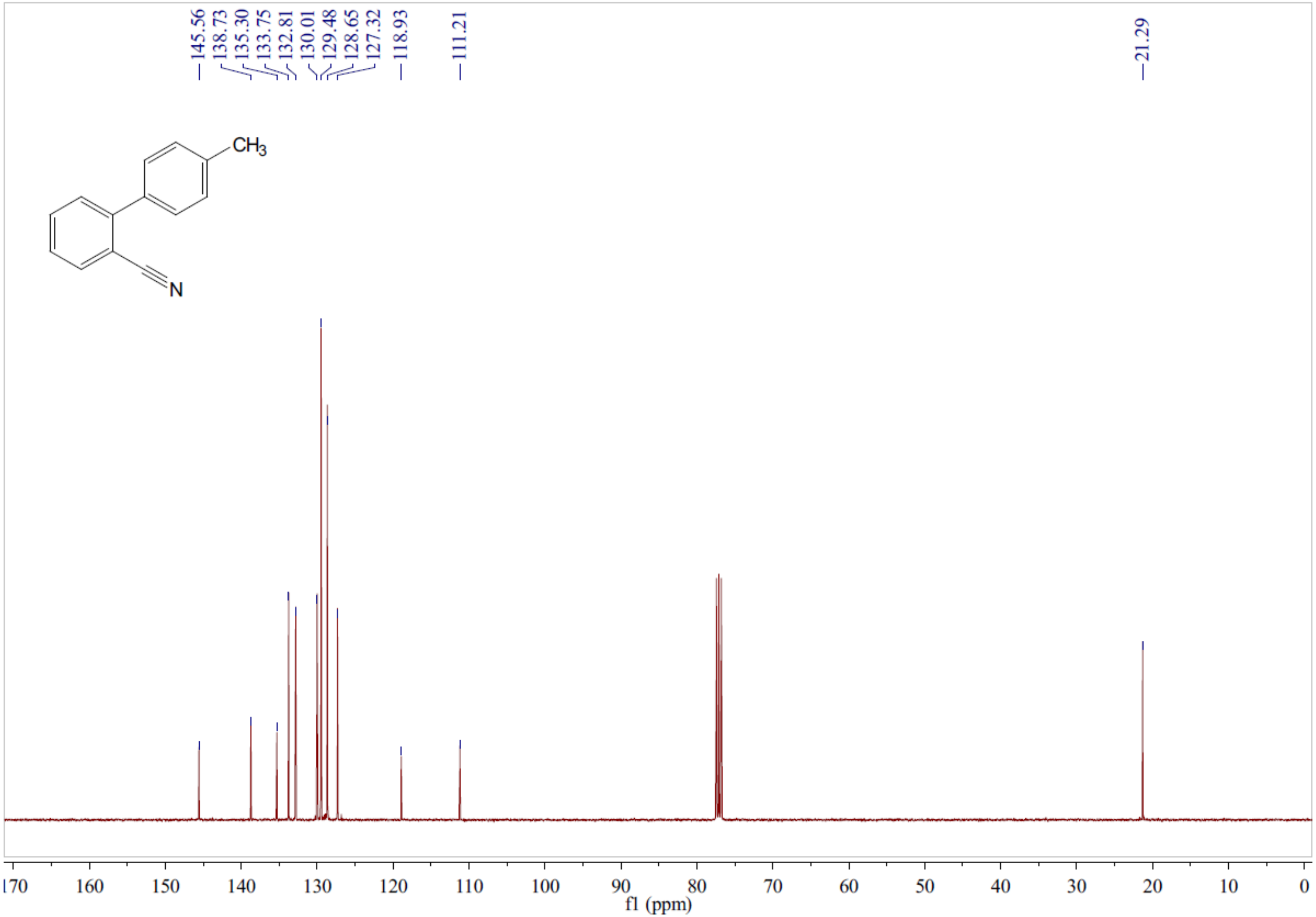

Supplement: RA-012-D2RA05890A-s001 [file RA-012-D2RA05890A-s001.pdf]
